# β-catenin activates TGF-β-induced epithelial–mesenchymal transition in adenomyosis

**DOI:** 10.1038/s12276-020-00514-6

**Published:** 2020-10-15

**Authors:** Jung-Yoon Yoo, Bon Jeong Ku, Tae Hoon Kim, Jong Il Ahn, Ji Yeon Ahn, Woo Sub Yang, Jeong Mook Lim, Maketo M. Taketo, Jung-Ho Shin, Jae-Wook Jeong

**Affiliations:** 1 Department of Biochemistry and Molecular Biology, Brain Korea 21 PLUS Project for Medical Sciences, Yonsei University College of Medicine, Seoul, 03722 Republic of Korea; 2 Department of Obstetrics, Gynecology, & Reproductive Biology, Michigan State University, Grand Rapids, MI 49503 USA; 3 Life Science Instituete, Repure Life Science, Seoul, 03722 Republic of Korea; 4 Department of Internal Medicine, Chungnam National University College of Medicine, Daejeon, 35015 Republic of Korea; 5 Department of Agricultural Biotechnology, Seoul National University, Seoul, 08826 Republic of Korea; 6 Research Institutes of Agriculture and Life Sciences, Seoul National University, Seoul, 08826 Republic of Korea; 7 Division of Experimental Therapeutics, Graduate School of Medicine, Kyoto University, Yoshida-Konoe-cho, Sakyo, Kyoto, 606–8501 Japan; 8 Division of Reproductive Endocrinology, Department of Obstetrics and Gynecology, Guro Hospital, Korea University Medical Center, Seoul, 02841 Republic of Korea

**Keywords:** Urogenital reproductive disorders, Gene regulation

## Abstract

Adenomyosis is defined as the presence of ectopic nests of endometrial glands and stroma within the myometrium. Adenomyosis is a common cause of dysmenorrhea, menorrhagia, and chronic pelvic pain but is often underdiagnosed. Despite its prevalence and severity of symptoms, its pathogenesis and etiology are poorly understood. Our previous study showed that aberrant activation of β-catenin results in adenomyosis through epithelial–mesenchymal transition. Using transcriptomic and ChIP-seq analysis, we identified activation of TGF-β signaling in the uteri of mutant mice that expressed dominant stabilized β-catenin in the uterus. There was a strong positive correlation between β-catenin and TGF-β2 proteins in women with adenomyosis. Furthermore, treatment with pirfenidone, a TGF-β inhibitor, increased E-cadherin expression and reduced cell invasiveness in Ishikawa cells with nuclear β-catenin. Our results suggest that β-catenin activates TGF-β-induced epithelial–mesenchymal transition in adenomyosis. This finding describes the molecular pathogenesis of adenomyosis and the use of TGF-β as a potential therapeutic target for adenomyosis.

## Uterine disease: Regulatory pathways identified

A regulatory link between two proteins involved in the progression of a debilitating uterine condition highlights a potential therapeutic target. Adenomyosis involves the invasion of cells from the inner lining of the uterus (the endometrium) into the uterine muscle wall (the myometrium), resulting in heavy, prolonged periods and chronic pain. The aberrent activation of a protein called β-catenin triggers adenomyosis, but the precise mechanisms are unclear. A team led by Jung-Ho Shin at the Korea University Medical Center, Seoul, South Korea, and Jae-Wook Jeong, Michigan State University, Grand Rapids, USA, used sequencing techniques in mice and human tissue samples to identify the pathways governed by β-catenin in adenomyosis. They found that the *Tgf-β2* gene is directly regulated by β-catenin in the uterus. TGF-β2 levels were elevated in human adenomyosis lesions, suggesting the protein could be a therapeutic target.

## Introduction

Adenomyosis is a common benign heterogeneous gynecological disorder defined by the presence of endometrial glandular and stromal tissue found in the myometrium^1,2^. Adenomyosis is diagnosed in 10 to 66% of women at the time of hysterectomy^3^. It is systematically associated with menorrhagia, dysmenorrhea, chronic pelvic pain and dyspareunia^4,5^, and can interfere with embryo implantation and cause subfertility^6,7^. Currently, adenomyosis is more frequently diagnosed in infertile patients by transvaginal ultrasonography and magnetic resonance imaging^8^. However, early diagnosis of adenomyosis is difficult because of the absence of pathogenomic symptoms and biomarkers. Therefore, most women are not diagnosed until later stages of the disease, and severely symptomatic women who do not respond to pharmacological therapy require invasive surgical intervention (hysterectomy). It has been suggested that adenomyosis is an ovarian steroid hormone-dependent disorder resulting from high estrogen levels unopposed by progesterone, similar to endometriosis, endometrial hyperplasia, and endometrial cancer^9,10^. However, the precise etiology and pathophysiology of adenomyosis is still unknown.

Studies using animal models in many different species, including mice, rats, rabbits, dogs, cats, and nonhuman primates, have provided insight into this disease^11^. Indeed, there have been several studies in mice using hormonal treatment that have shown an increased incidence of adenomyosis^12–14^. Nevertheless, the molecular mechanism for the development and progression of adenomyosis is still unclear. Mice with uterine conditional activation of β-catenin (*Pgr*^*cre/+*^*Ctnnβ*^*f(ex3)/+*^) develop adenomyosis^15^. *Pgr*^*cre/+*^*Ctnnb1*^*f(Ex3)/+*^ mouse has stabilization of β-catenin in *Pgr*-expressing cells and is a useful mouse model to determine the effect of constitutive activation of β-catenin in the mouse uterus^16^. Our previous data showed that *Pgr*^*cre/+*^*Ctnnb1*^*f(ex3)/+*^ mice exhibited infertility, hormone insensitivity, and endometrial glandular hyperplasia^16^. Although the *Pgr*^*Cre*^ mouse shows Cre recombinase activity in the pituitary, ovary, and mammary glands, *Pgr*^*cre/+*^*Ctnnb1*^*f(ex3)/+*^ mice revealed normal ovarian functions and did not develop breast cancer. Therefore, it is a useful model system to investigate the genetic and molecular events involved in the transition from normal uterine structure to adenomyosis.

β-catenin has a dual function, regulating both the coordination of cell–cell adhesion and gene transcription, depending on its localization in the cell. At the plasma membrane, β-catenin is a component of the E-cadherin-catenin unit, where it maintains cell differentiation and normal tissue architecture. β-catenin nuclear translocation depends on Wnt signaling, and nuclear β-catenin can act as a transcriptional activator to regulate the transcription of target genes responsible for cell proliferation and differentiation^17^. The Wnt/β-catenin pathway is important in tissue differentiation during embryonic development and tissue homeostasis and function in adults. In the absence of Wnt signaling, β-catenin is degraded by the β-catenin destruction complex, including adenomatous polyposis coli (APC) and glycogen synthase kinase 3β (GSK-3β)^18–21^. Mutations and excessive activation of β-catenin are associated with many cancers, including hepatocellular carcinoma, colorectal carcinoma, lung cancer, malignant breast tumors, and ovarian and endometrial cancer^22,23^, as well as epithelial–mesenchymal transition (EMT)^24,25^.

EMT is a biological process by which epithelial cells lose cell polarity and cell adhesion and gain migratory and invasive properties to become mesenchymal cells^25^. EMT is important for several developmental processes, including embryogenesis, organ fibrosis, and wound healing^26,27^. EMT is also critical in the initiation of metastasis for cancer progression^28^, as well as in uterine diseases such as adenomyosis, endometriosis, and endometrial cancer^15,29,30^. Serum estrogen levels are negatively correlated with E-cadherin expression levels in the epithelial components of the eutopic endometrium and adenomyotic lesions compared to controls^30^. EMT is initiated by a number of transcription factors, including Snail, Slug, Twist, ZEB1, and SIP1, via the repression of E-cadherin expression^31^. Furthermore, EMT is induced or regulated by growth and differentiation factors such as TGF-β^32^. Crosstalk between TGF-β/Smad signaling and Wnt/β-catenin signaling is important in developmental and pathological events^33,34^. TGF-β signaling influences the Wnt pathway by regulating the activity of β-catenin through the interaction of activated Smad complexes with APC/GSK-3β, β-catenin, and TCF/LEF1^34,35^. In the context of EMT, Smad2 and Smad4 form a complex with LEF1 and suppress the expression of E-cadherin by binding to the E-cadherin promoter. These complexes induce the expression of the mesenchymal markers vimentin and fibronectin^36^.

In this study, we performed transcriptomic and ChIP-seq analyses under in vivo conditions of aberrant β-catenin activation to identify genome-wide β-catenin targets in the mouse uterus. We found that *Tgf-β2* is directly regulated by activated β-catenin in the murine uterus. The levels of TGF-β2 were higher in endometrial epithelial cells of human adenomyosis lesions and the corresponding eutopic endometrium than in control women. Furthermore, we found a positive correlation between β-catenin and TGF-β2 proteins in the endometrium from women with and without adenomyosis. These results suggest that TGF-β2 plays a critical role in adenomyosis development as a direct target of β-catenin.

## Materials and methods

### Animals and tissue collection

Mice were maintained in the designated animal care facility according to Michigan State University’s institutional guidelines. All animal procedures were approved by the Institutional Animal Care and Use Committee of Michigan State University. Uterine tissues were collected from control (*Pgr*^*cre/+*^ and *Ctnnb1*^*f(ex3)/+*^) and mutant (*Pgr*^*cre/+*^*Ctnnb1*^*f(ex3)/+*^) mice^16,37^. Uterine tissues were immediately frozen at the time of dissection and stored at −80 °C for RNA extraction and ChIP analysis or fixed with 4% (v/v) paraformaldehyde for histology and immunostaining analysis.

### Human adenomyosis samples

Human adenomyosis samples were obtained from Korea University Guro Hospital of Biobank with the approval guidelines instituted by the Institutional Review Boards of Korea University. Written informed consent was obtained from all participants. Adenomyosis tissue samples with their corresponding eutopic endometrium were collected from surgical hysterectomy specimens. Controls were regularly cycling premenopausal women undergoing an endometrial hysterectomy for benign conditions with no history or evidence of adenomyosis, who were documented not to be pregnant and who had not been on hormonal therapies for at least 3 months before tissue sampling. Histologic dating of endometrial samples was performed based on the criteria of Noyes^38^. Eutopic endometrium (4 proliferative and 4 secretory phases) and adenomyosis lesion (15 proliferative and 15 secretory phases) samples were from women with adenomyosis, and control endometrium (*n* = 8 proliferative and *n* = 13 secretory) samples were from women without adenomyosis. Samples used for immunostaining were fixed in 10% buffered formalin prior to embedding in paraffin wax.

### RNA isolation and microarray analysis

Total RNA was extracted using the RNeasy Total RNA Isolation Kit (Qiagen, Valencia, CA, USA). RNA was pooled from the uteri of 3 mice per genotype at 1 month of age. All RNA samples were analyzed with a Bioanalyzer 2100 (Agilent Technologies, Wilmington, DE, USA) before microarray hybridization. Microarray data analysis was performed as previously described^39^. Microarray data are deposited in the Gene Expression Omnibus (GEO) database, with accession number GSE88911. Differentially expressed genes were classified according to canonical pathway analysis by Ingenuity System Software (Ingenuity Systems Inc., Redwood City, CA, USA).

### Chromatin immunoprecipitation sequencing (ChIP-seq) analysis

β-catenin and input ChIP were performed by Active Motif, Inc. (Carlsbad, CA, USA) on control and mutant mouse uteri at 1 month of age. ChIP and input DNA were amplified using the Illumina ChIP-Seq DNA Sample Prep Kit (Illumina, San Diego, CA, USA). DNA libraries were sequenced by Illumina’s Hi-Seq Sequencing Service. The sequences were aligned to the mouse genome (NCBI Build 37, July 2007). Alignments were extended in silico (using Active Motif software) at their 3′ ends to a length of 150–250 bp and assigned to 32-nucleotide bins along the genome. Peak locations were determined by applying a threshold of 18 (five consecutive bins containing 18 aligns) and storing the resulting intervals in Browser Extensible Data (BED) files (BED, Affymetrix TAS software). These files were analyzed using Genpathway proprietary software that provides comprehensive information on genomic annotation, peak metrics, and sample comparisons for all peaks (intervals). The model-based analysis of the ChIP-Seq (MACS^40^) peak-finding algorithm was used to normalize ChIP against the input control. Genes associated with intervals were assessed using three increasingly less stringent requirements; if it was within 10, 25, or 50 kb upstream or downstream of a gene, it was counted. Sequence conservation to identify phastCons scores, analysis of enriched motifs, and cis-regulatory element annotation system (CEAS) were performed using the Cistrome Analysis Pipeline software (http://cistrome.org/ap/root) under default settings^41^. Ingenuity Systems Pathway Analysis software (http://www.ingenuity.com/products/ipa) was used for gene functional annotations.

### ChIP assays

ChIP assays were performed in the uteri of control and mutant mice at 4 weeks of age as previously described^39^. Briefly, for each ChIP reaction, 100 μg of chromatin was immunoprecipitated by 4 μg of antibodies against β-catenin (610154; BD Biosciences, San Jose, CA, USA). Eluted DNA was amplified with specific primers using SYBR Green Supermix (Bio-Rad Laboratories, Inc., Hercules, CA, USA). The β-catenin-binding site (CBS) for the ChIP assay was determined by ChIP-seq, and primers for quantitative reverse transcription PCR (RT-qPCR) were designed to query the β-catenin intervals on *Tgf-β2*. One negative control (NC) site on the *Tgf-β2* gene was designed in an area where no enrichment of β-catenin was observed in mutant mouse uteri. The PCR primers were as follows: CBS (forward: 5′-GTTACCGAGGGGCAGAATGC-3′; reverse: 5′-GGGGGTCATCATACAAGGCA-3′) and negative control (NC; forward: 5′-CCGGAGCTCCTCAGATCCAC-3′; reverse: 5′-TGGATCCCTCTCCATCCCAC-3′). The resulting signals were normalized to input activity.

### Quantitative real-time PCR Analysis

Complementary DNA was produced from 1 μg of total RNA using random hexamers and MMLV Reverse Transcriptase (Invitrogen Corp., Carlsbad, CA, USA). Real-time PCR was performed using an RT-PCR SYBR Green detection system (Bio-Rad, Hercules, CA, USA) according to the manufacturer’s instructions (PE Applied Biosystems, Foster City, CA, USA). mRNA quantities were normalized against the housekeeping gene *Rpl7* RNA. The sequences of the primers used for mouse *Tgf-β2* were 5′-TAAAATCGACATGCCGTCCC-3′ and 5′-GAGACATCAAAGCGGACGAT-3′ and for mouse *Rpl7* were 5′-TCAATGGAGTAAGCCCAAAG-3′ and 5′-CAAGAGACCGAGCAATCAAG-3′.

### Immunohistochemistry and immunofluorescence analysis

Immunohistochemistry and immunofluorescence analyses were performed as previously described^42^. Uterine sections from paraffin-embedded tissues were preincubated with 10% normal serum in phosphate-buffered saline (PBS) and incubated with anti-TGF-β2 (ab36495; Abcam, Cambridge, MA, USA), anti-E-cadherin (610181; BD Bioscience, San Jose, CA, USA), anti-F4/80 (nbp2-12506; Novus, Littleton, CO, USA), anti-CD68 (SC-5474; Santa Cruz Biotechnology, Santa Cruz, CA, USA), anti-CD4 (SC-1140; Santa Cruz Biotechnology, Santa Cruz, CA, USA), and anti-vimentin (ab92547; Abcam, Cambridge, MA, USA and SC-6260; Santa Cruz Biotechnology, Santa Cruz, CA, USA) antibodies in 10% normal serum in PBS. On the following day, sections were washed in PBS and incubated with a secondary antibody (Vector Laboratories, Burlingame, CA, USA) for 1 h at room temperature. Immunoreactivity was detected using the Vectastain Elite DAB Kit (Vector Laboratories). Images were captured with a confocal microscope (510 NLO confocal microscope; Carl Zeiss, Thornwood, NY, USA).

### Cell culture and transient transfections

Cells from the Ishikawa uterine endometrial epithelial cell line (endometrial adenocarcinoma) were cultured in DMEM/F12 medium (Gibco, Grand Island, NY, USA) supplemented with 10% fetal bovine serum (FBS; Gibco, Grand Island, NY, USA) and 1% penicillin streptomycin (Gibco, Grand Island, NY, USA). Cells were cultured in monolayers at 37 °C in 5% CO_2_. Transient transfection of the exon 3-deleted β-catenin vector^21^ into Ishikawa cells was performed using Lipofectamine 2000 (Invitrogen Crop., Carlsbad, CA, USA).

### Western blot analysis

Western blot analyses were performed as described previously^43^. Samples, which were lysed in buffer containing 150 mM NaCl, 10 mM Tris-HCl (pH 7.4), 2.5 mM EDTA, 0.125% Nonidet P-40 (v/v), and protease inhibitors, were separated by sodium dodecyl sulfate–polyacrylamide gel electrophoresis and transferred to polyvinylidene difluoride membranes (Millipore, Bedford, MA, USA). Membranes were probed with anti-β-catenin (610154; BD Biosciences, San Jose, CA, USA), anti-TGF-β2 (ab36495; Abcam, Cambridge, MA, USA), anti-E-cadherin (610181; BD Biosciences, San Jose, CA, USA), anti-vimentin (SC-6260; Santa Cruz Biotechnology, Santa Cruz, CA, USA), and anti-actin (SC-1615; Santa Cruz Biotechnology, Santa Cruz, CA, USA) antibodies. Immunoreactivity was visualized by autoradiography.

### Invasion assay

For the transwell invasion assay, post-transfected cells (24 h) were trypsinized and seeded at a density of 2.5 × 10^5^ per 200 μl serum-free culture medium into the insert chamber of a BioCoat (24-well insert; pore size, 8 μm; BD Biosciences, San Jose, CA, USA). The cells were incubated at 37 °C in 5% CO_2_ for 48 h, and noninvading cells were removed with a cotton swab. Invading cells on the lower surface of the membrane were fixed with 100% methanol and stained with 1% crystal violet (Sigma-Aldrich, St. Louis, MO, USA). Stained cells were observed using fluorescence microscopy (Nikon Instruments Inc., Melville, NY, USA) using software from NIS Elements, Inc. (Nikon, Melville, NY, USA).

### Statistical analysis

Statistical analyses were performed using Student’s *t*-test for data with only two groups or analysis of variance (ANOVA) for data containing more than two groups, followed by Tukey’s post hoc test for multiple comparisons using Instat (GraphPad, San Diego, CA, USA). *p* < 0.05 was considered statistically significant.

## Results

### Integrated approach to identify β-catenin-regulated genes for adenomyosis development

Our previous study showed that adenomyosis developed in mice with uterine-specific β-catenin activation^15^. An integrated bioinformatics approach combining chromatin immunoprecipitation sequencing (ChIP-seq) and transcriptomic analysis was applied to identify direct target genes of β-catenin in the uterus of control (*Pgr*^*cre/*+^ and *Ctnnb1* ^*f(ex3)/*+^) and mutant (*Pgr*^*cre/*+^ *Ctnnb1* ^*f(ex3)/*+^) mice at 4 weeks of age. To determine the β-catenin cistrome in the uterus, ChIP-seq results were used to create a genome-wide profile of in vivo β-catenin-binding sites in control and mutant mice. More than 20 million tags of each sample were mapped to unique locations in the mouse genome. A model-based analysis peak-finding algorithm was used to normalize immunoprecipitated chromatin against input with a value of *p* = 1 × 10^−7^ (<1% false-discovery rate). This high-confidence cutoff produced 2092 intervals in the control uterine samples and 3524 intervals in the mutant uterine samples. A total of 606 β-catenin-bound intervals were in common between the control and mutant uteri. This approach identified 2918 binding target sites unique to the mutant uterus that are potential β-catenin target genes in the mutant mice (Supplementary Table S1).

**Fig. 1:**
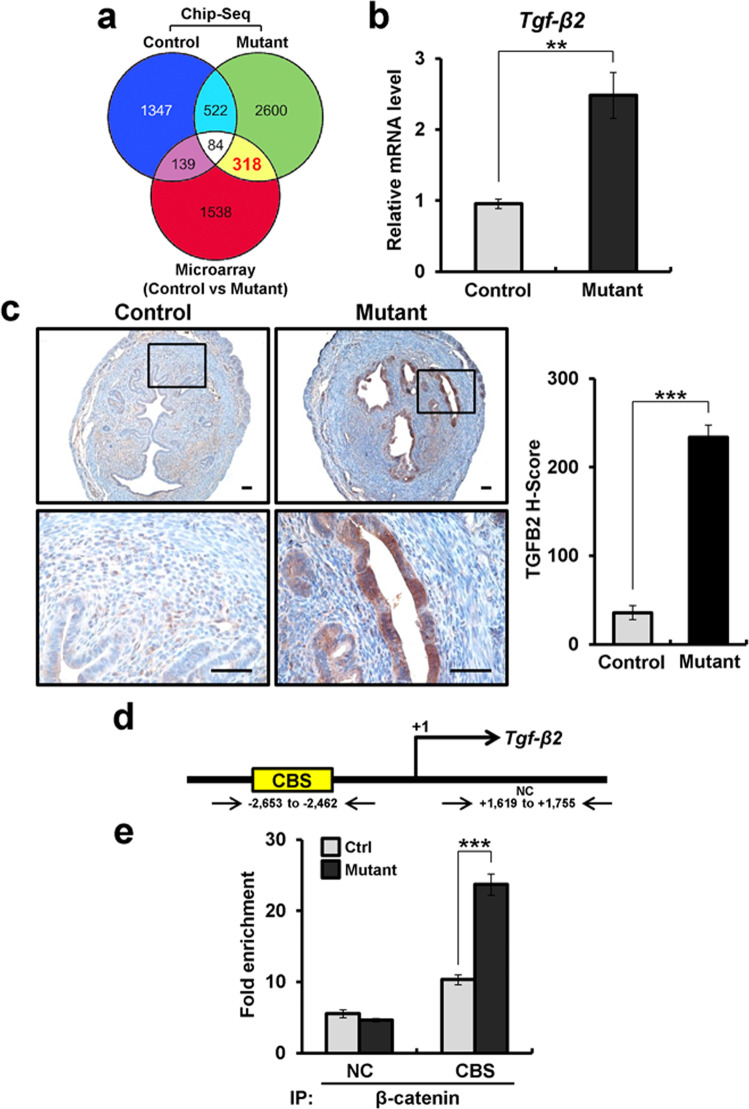
Identification of *Tgf-β2* as a target gene of β-catenin in the murine uterus. **a** Venn diagram illustrating the overlap between nonredundant genes bound by β-catenin as determined by ChIP-seq and those genes regulated by β-catenin by transcriptomic analysis (>1.5-fold) in control and mutant mouse uteri. **b** Quantitative real-time PCR analysis of *Tgf-β2* in the uterus of control and mutant mice at 4 weeks of age (*n* = 3 per genotype). **c** Representative photomicrographs and histological score (H-score) of TGF-β2 immunohistochemical staining in uteri of control and mutant mice at 4 weeks of age (*n* = 5 per genotype). Scale bars represent 50 μm. **d** Map of the β-catenin-binding site (CBS) and negative control (NC) on the *Tgf-β2* promoter. **e** ChIP assay using the anti-β-catenin antibody on the *Tgf-β2* promoter in control and mutant mouse uteri at 4 weeks of age (*n* = 3). The results represent the mean ± SEM. **p < 0.01 and ***p < 0.001.

Next, our transcriptomic analysis identified 2079 genes (fold change >1.5 and *p* < 0.05) as differentially expressed in the uteri of mice with uterine-specific β-catenin activation compared to controls. Of the 2079 genes, 1103 were increased, and 976 genes were decreased (Supplementary Table S2). Functional annotation was performed to determine candidate β-catenin-regulated pathways for adenomyosis development using Ingenuity Systems Pathway Analysis. Candidate β-catenin-activated pathways were involved in Wnt/β-catenin, embryonic stem cell pluripotency, basal cell carcinoma signaling, and TGF-β signaling. From comparison of the ChIP-seq and microarray results, we identified 318 genes that not only contain β-catenin-binding sites but also exhibit altered expression in the uterus of mutant mice compared with controls (Fig. 1a; yellow). Of the 318 genes, 189 were increased, and 129 genes were decreased in mutant mice compared to controls (Supplementary Table S3).

### *Tgf-β2* is a direct target gene of β-catenin in the uterus

Interestingly, the pathway analysis identified dysregulation of the TGF-β signaling pathway, and the ChIP-seq analysis identified β-catenin-binding sites at the promoter region of transforming growth factor beta 2 (*Tgf-β2*). It has been reported that TGF-β1 levels are elevated in uterine washings from patients with adenomyosis^44^, as well as in a mouse model of adenomyosis^45^. However, *Tgf-β1* expression was not altered in our transcriptomic analysis, and *Tgf-β3* was significantly decreased in the mutant mice compared to the controls (Supplementary Fig. S1). TGF-β signaling has been shown to play an important role in EMT^35^. Therefore, we validated the transcriptomic and ChIP-seq analysis results for *Tgf-β2* in uterine tissue of control and mutant mice using RT-qPCR and ChIP analysis. The expression of *Tgf-β2* mRNA was significantly increased in mutant mice compared to control mice at 4 weeks of age (Fig. 1b). Immunohistochemistry analysis showed that the mutant mouse uterus exhibited an increase in TGF-β2 in epithelial cells compared to control mice (Fig. 1c). Furthermore, the recruitment of stabilized β-catenin onto the promoter of *Tgf-β2* was also confirmed in the uterus of mutant mice by ChIP analysis (Fig. 1d, e). These results suggest that *Tgf-β2* is a direct target of β-catenin in the uterus and may play an important role in adenomyosis development.

**Fig. 2:**
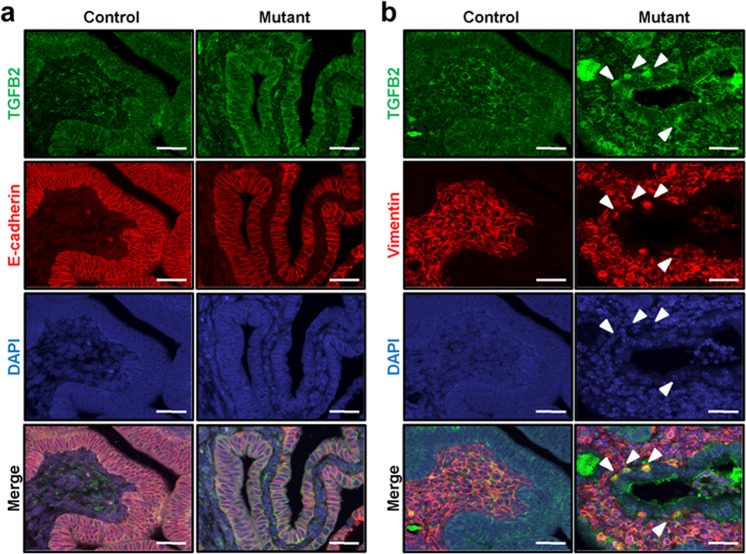
The expression of TGF-β2 and E-cadherin in the mutant mouse uterus. Immunofluorescence analysis of TGF-β2 and E-cadherin (**a**) or vimentin (**b**) in the uteri of control and mutant mice at 4 weeks of age (*n* = 5 per genotype). Nuclei were counterstained with DAPI. Arrowheads indicate TGF-β2- and vimentin-positive epithelial cells. Scale bars represent 50 μm.

To determine whether TGF-β2 expression is associated with EMT in the mutant uterus, we performed double immunofluorescence for TGF-β2 with E-cadherin (as an epithelial cell marker) and vimentin (as a mesenchymal cell marker) in the uteri of control and mutant mice at 4 weeks of age. The levels of TGF-β2 were higher in endometrial epithelial cells of mutant mice compared to the control, while E-cadherin levels were decreased in endometrial epithelial cells of mutant mice (Fig. 2a). The expression of vimentin was observed in some uterine epithelial cells of the mutant mice. The vimentin-positive epithelial cells also expressed TGF-β2 (Fig. 2b). Immunofluorescence with normal IgG was performed as a negative control (Supplementary Fig. S2). To determine whether the vimentin-positive cells were immune cells, we performed immunofluorescence for F4/80 and CD68, macrophage markers, and CD4, a T helper lymphocyte marker, with vimentin. The vimentin-positive epithelial cells were neither F4/80- and CD68- nor CD4-positive in the uterus of mutant mice (Supplementary Fig. S3). These results suggest that β-catenin targets TGF-β2 to mediate EMT for adenomyosis development.

### TGF-β2 overexpression in mice with β-catenin activation during adenomyosis development

While 2-month-old mutant mice do not show adenomyosis, the incidence of adenomyosis in 4- and 6-month-old mutant mice was 40% and 80%, respectively^15^. To determine whether TGF-β2 is dysregulated during adenomyosis development, we examined the level of TGF-β2 in the uteri of control and mutant mice at 2, 4, and 6 months of age using immunohistochemistry analysis. The levels of TGF-β2 were higher in endometrial epithelial cells of mutant mice at 2, 4, and 6 months of age compared to controls (Supplementary Fig. S4).

### Aberrant activation of TGF-β2 in eutopic endometrium and adenomyosis lesions from women with adenomyosis

**Fig. 3:**
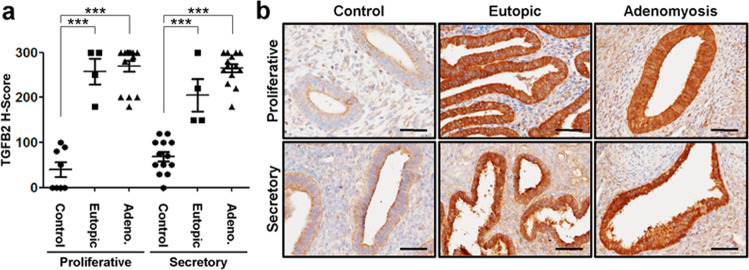
Overexpression of TGF-β2 in eutopic endometrium and adenomyotic lesions from women with adenomyosis. **a** Semiquantitative analysis of TGF-β2 levels in control endometrium from the proliferative (*n* = 8) and secretory (*n* = 13) phases and eutopic endometrium (*n* = 4 per phase) and adenomyotic lesions (*n* = 15 per phase) from the proliferative and secretory phases with adenomyosis analyzed by immunohistochemical H-score. The results represent the mean ± SEM. ***p < 0.001. **b** Representative photomicrographs of immunohistochemical staining of TGF-β2 in women endometrium with (eutopic and adenomyostic lesions) and without adenomyosis during the proliferative and secretory phases. Controls represent the endometrium from women with no history or evidence of adenomyosis. Eutopic refers to the endometrium, and adenomyosis refers to lesions from women with adenomyosis. Scale bars represent 50 μm.

To determine whether TGF-β2 overexpression is related to human adenomyosis, we examined the expression of TGF-β2 proteins in eutopic endometrium and adenomyosis lesions from women with adenomyosis using immunohistochemistry. TGF-β2 proteins were weakly detected throughout the menstrual cycle in human endometrial epithelial and stromal cells. However, the levels of TGF-β2 were significantly higher in epithelial cells of eutopic endometrium and adenomyosis lesions compared to control endometrium without adenomyosis at the proliferative phase as well as the secretory phase (Fig. 3).

**Fig. 4:**
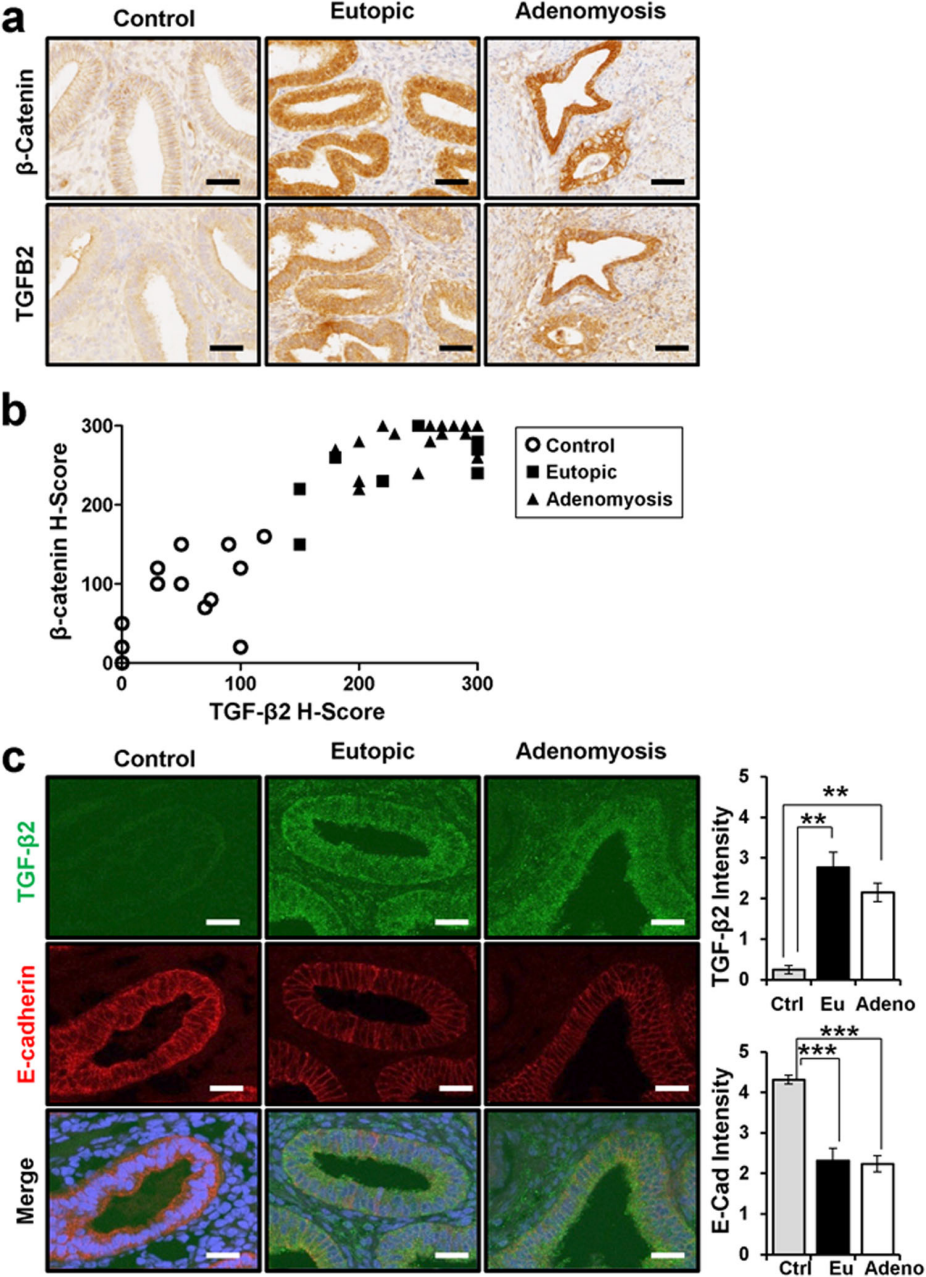
Positive correlation between TGF-β2 and β-catenin in the human endometrium with and without adenomyosis. **a** Representative photomicrographs of immunohistochemical staining of TGF-β2 and β-catenin in the endometrium of women with and without adenomyosis. **b** Correlation analysis of TGF-β2 and β-catenin in control (*n* = 14) and endometrial (*n* = 8) and adenomyotic lesions (*n* = 30) of adenomyosis based on immunohistochemistry results (correlation coefficient = 0.9136, *p* < 0.0001). **c** Immunofluorescence analysis and intensity quantification of TGF-β2 and E-cadherin in women endometrium without adenomyosis (*n* = 14) and endometrium (*n* = 13) and adenomyotic lesions (*n* = 29) of adenomyosis. Nuclei were counterstained with DAPI. Scale bars represent 50 μm. The results represent the mean ± SEM. **p < 0.01 and ***p < 0.001.

Since TGF-β2 was identified as a direct target of β-catenin, we examined the relationship between β-catenin and TGF-β2 proteins in adenomyosis patients. The levels of β-catenin and TGF-β2 proteins were examined and compared in women with and without adenomyosis using immunohistochemistry. Semiquantitative analysis showed a significant positive correlation between β-catenin and TGF-β2 in endometrial epithelial cells (Spearman correlation coefficient *r* = 0.9136, *p* < 0.0001; Fig. 4a, b).

The expression of E-cadherin is lower in epithelial cells of eutopic endometrium and adenomyosis lesions of mutant mice as well as human adenomyosis patients^15^. Therefore, we examined the expression of TGF-β2 and E-cadherin using double immunofluorescence staining. Interestingly, eutopic endometrium and adenomyosis lesions from patients with adenomyosis showed higher levels of TGF-β2 and lower levels of E-cadherin compared to controls, similar to what was observed in the mutant mice (Fig. 4c). These results suggest that β-catenin-induced EMT contributes to adenomyosis development through TGF-β2.

### β-catenin leads to epithelial–mesenchymal transition in adenomyosis development through TGF-β2

**Fig. 5:**
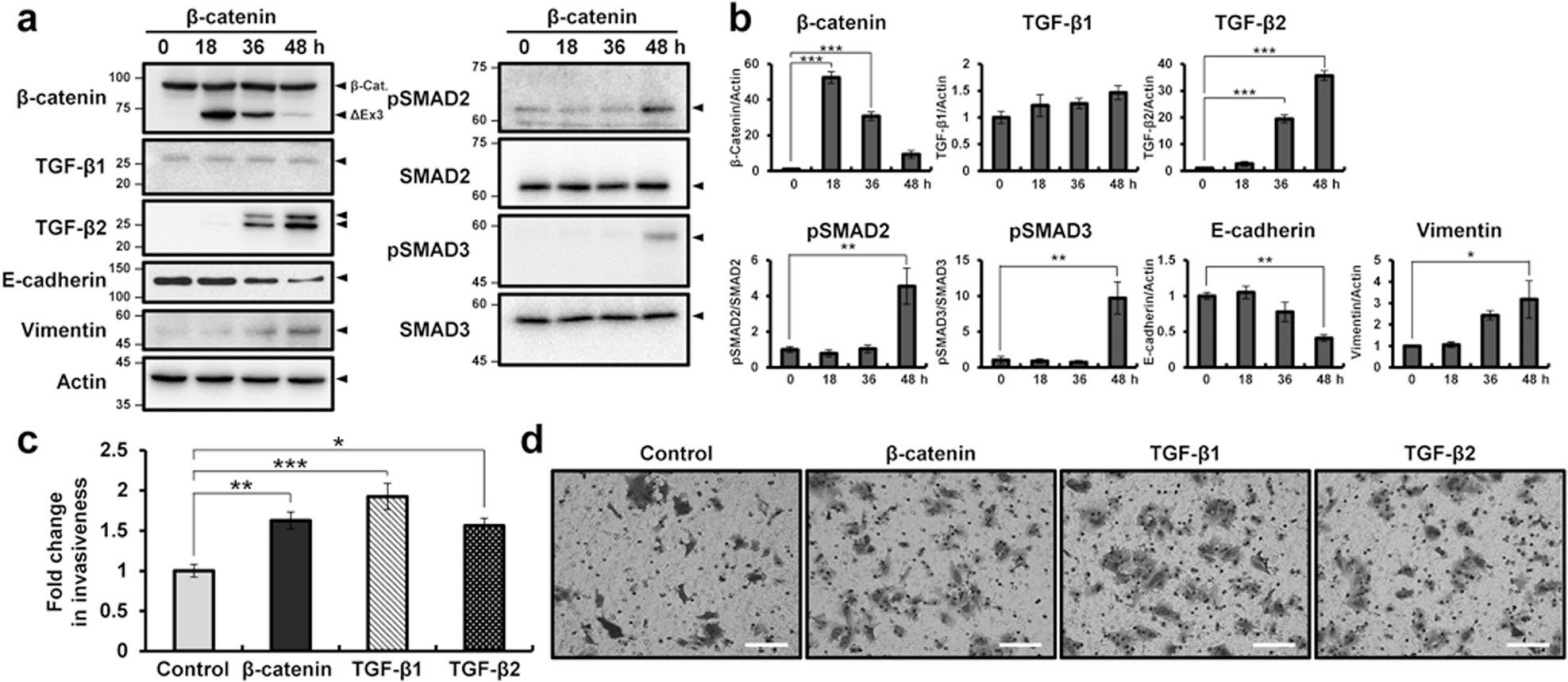
Increased TGF-β2 by β-catenin activation leads to EMT in adenomyosis development. **a** Western blot analysis of β-catenin, TGF-β2, E-cadherin, and vimentin in Ishikawa cells transfected with exon 3-deleted β-catenin vector for 0, 18, 36, and 48 h. Actin was used as a sample loading control. **b** Quantification of western blot results in Ishikawa cells transfected with exon 3-deleted β-catenin vector for 0, 18, 36, and 48 h (*n* = 3). **c** Quantification of invasion through the Matrigel and transwell membrane of Ishikawa cells transfected with control or exon 3-deleted β-catenin vector and treated with TGF-β1 or TGF-β2 (*n* = 5). **d** Representative results of transwell invasion assays of control or exon 3-deleted β-catenin vector-transfected and TGF-β1- or TGF-β2-treated Ishikawa cells. The results represent the mean ± SEM. *p < 0.05, **p < 0.01, and ***p < 0.001. Scale bars represent 100 μm.

To determine the molecular mechanisms of activated-β-catenin in the EMT process, exon 3-deleted β-catenin was expressed in Ishikawa cells using transient transfection. Exon 3-deleted β-catenin is not phosphorylated by GSK-3β and results in consistent stabilization of the protein, nuclear accumulation, and participation in signal transduction and transcriptional activation through complex formation with DNA binding proteins^21^. As expected, TGF-β2 levels gradually increased after exon 3-deleted β-catenin was expressed in Ishikawa cells. However, the expression of TGF-β1 was not altered in Ishikawa cells transfected with activated β-catenin. While the expression levels of SMAD2 and SMAD3 did not differ after β-catenin overexpression, the levels of pSMAD2 and pSMAD3 were increased in Ishikawa cells transfected with activated β-catenin. The expression of E-cadherin was decreased in Ishikawa cells with activated β-catenin. In contrast, the level of vimentin increased over time in Ishikawa cells with activated β-catenin (Fig. 5a, b).

EMT is a process in which epithelial cells acquire the characteristics of invasive mesenchymal cells^46^. Therefore, we examined the invasiveness of Ishikawa cells after β-catenin activation, as well as TGF-β1 and TGF-β2 treatment using a transwell invasion assay. The invasiveness of β-catenin-activated Ishikawa cells was increased by more than 1.7-fold compared to control cells, and the TGF-β1 and TGF-β2 treatments also elicited a higher infiltration rate compared to controls using Ishikawa cells (Fig. 5c, d).

**Fig. 6:**
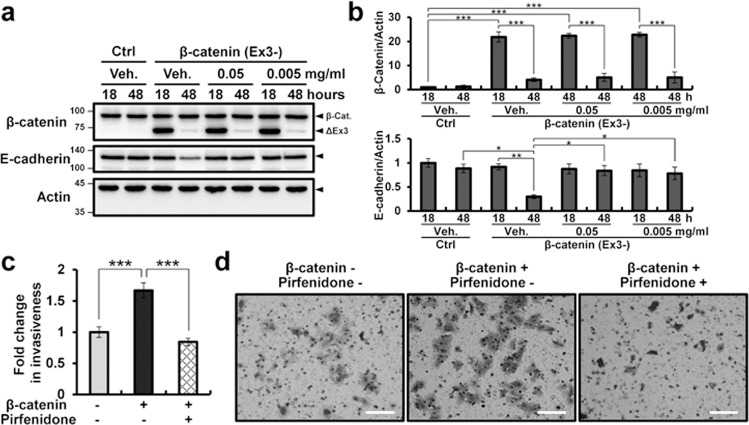
Decrease in the β-catenin-induced EMT process in Ishikawa cells by inhibition of TGF-β. **a** Western blot analysis of β-catenin and E-cadherin in control or exon 3-deleted β-catenin vector-transfected Ishikawa cells treated with or without pirfenidone for 18 and 48 h. Actin was used as a sample loading control. **b** Quantification of western blot results in the control or exon 3-deleted β-catenin vector-transfected Ishikawa cells treated with or without pirfenidone for 18 and 48 h (*n* = 3). **c** Quantification of invasion through the Matrigel and transwell membrane control or exon 3-deleted β-catenin vector-transfected Ishikawa cells treated with or without pirfenidone for 48 h (*n* = 5). **d** Representative results of transwell invasion assays of control or exon 3-deleted β-catenin vector-transfected Ishikawa cells treated with or without pirfenidone for 48 h. The results represent the mean ± SEM. *p < 0.05, **p < 0.01, and ***p < 0.001. Scale bars represent 100 μm.

We next examined whether EMT in β-catenin activation could be interrupted by inhibition of TGF-β using pirfenidone (5-methyl-1-2-[1H]-pyridone). After transfection of exon 3-deleted β-catenin, Ishikawa cells were treated with pirfenidone, and the levels of E-cadherin and invasiveness of Ishikawa cells were examined. The levels of E-cadherin in Ishikawa cells transfected with activated β-catenin were decreased at 48 h. However, pirfenidone treatment abrogated this effect (Fig. 6a, b). Furthermore, the invasiveness of Ishikawa cells following β-catenin activation was inhibited by pirfenidone treatment (Fig. 6c, d). The effect of pirfenidone was confirmed with two other TGF-β inhibitors, GW788388 and R268712 (Supplementary Fig. S5).

## Discussion

This study represents the first comprehensive analysis of the β-catenin cistrome in the uterus, which is important due to β-catenin’s critical role in uterine development and function^16,47–49^, as well its pathological role in adenomyosis development^15^. The integrated uterine transcriptome and ChIP-seq analyses presented here define pathways governed by β-catenin during adenomyosis development. The present study found many candidate genes and pathways that are likely positively regulated by β-catenin in the development of adenomyosis in the mutant uterus. From the pathway analysis using Ingenuity Systems Software, we found altered pathways, including Wnt/β-catenin signaling, TGF-β signaling, embryonic stem cell pluripotency, basal cell carcinoma signaling, ErbB signaling, and ERK/MAPK signaling. Future mechanistic studies will be needed to understand their biological roles in adenomyosis development.

The molecular mechanism for the development and progression of adenomyosis is still unclear, and an appropriate genetically modified mouse model has not yet been developed. Mice with uterine conditional activation of β-catenin were used to explore how the dysregulation of β-catenin signaling can lead to the development of adenomyosis. The major advantage of using such models is spontaneous adenomyosis development and the presence of an intact immune system, which is lost in xenotransplant models. Our animal model constitutes a novel model system that investigates the genetic and molecular events involved in the transition from normal uterine structure to adenomyosis.

As with any mouse model for human disease, the data should be properly interpreted within the boundaries of its context. As such, questions remain as to whether women with activated TGF-β signaling in their eutopic endometrium are more prone to developing adenomyosis. Transgenic models such as the one presented in this study provide evidence that if there is an inherent defect in the eutopic endometrium, this induces the development of adenomyosis. Although there is no perfect model for human adenomyosis aside from the human disease condition, studies such as these provide proof-of-principle evidence to explore and consider potential targets of therapy for adenomyosis.

In this study, we demonstrated that TGF-β2 overexpression plays an important role in the pathogenesis of adenomyosis with aberrant activation of β-catenin. TGF-β superfamily signaling plays a pleiotropic role in fundamental cellular and developmental processes^50^. TGF-β superfamily members are key regulators of female reproduction, including ovulation, uterine decidualization, and embryo development^51–53^. TGF-β signaling has been shown to play an important role in EMT^54^, and it influences Wnt/β-catenin signaling through the interaction of activated Smad complexes with APC/GSK-3β, β-catenin, and TCF/LEF1^34,35^. Liu et al.^55^ reported the increased expression of TGF-β1 in adenomyotic lesions. However, our results revealed that TGF-β1 is not a target of β-catenin. Interestingly, we found a strong positive correlation between β-catenin and TGF-β2 in the endometrial epithelial cells of women with and without adenomyosis. The levels of TGF-β2 were remarkably strong in endometrial epithelial cells of mutant mice compared to controls. Our immunofluorescence results confirmed that vimentin-positive epithelial cells express TGF-β2 proteins in the mutant mice, which suggests that aberrant activation of β-catenin and TGF-β2 plays an important role in the pathogenesis of adenomyosis.

The regulation of EMT is mostly initiated by several signaling pathways, including TGF-β, Wnt/β-catenin, and growth factors^56^. TGF-β signaling is widely known to be associated with multiple signaling pathways, such as cell homeostasis, immunomodulatory functions and EMT formation. TGF-β cooperates with Wnt/β-catenin to forward complete EMT and regulate the mesenchymal phenotype of invasive/metastatic tumor cells^57^. TGF-β can upregulate canonical Wnt signaling^58^. TGF-β1 induced β-catenin nuclear translocation in primary porcine valve interstitial cells through TGF-β receptor I kinase^59^. However, this is the first report that β-catenin induces EMT process through TGF-β2 in adenomyosis development.

Furthermore, Ishikawa cells with nuclear β-catenin expression induced the expression of TGF-β2 and vimentin but decreased the expression of E-cadherin. Overexpression of TGF-β2 in human adenomyosis and the ability of a specific TGF-β inhibitor to decrease cell invasion indicate that the TGF-β pathway is critical for the establishment of adenomyosis. Treatment of Ishikawa cells with the TGF-β inhibitor pirfenidone decreased the EMT process and preferentially inhibited cell invasion, demonstrating the major role TGF-β plays in adenomyosis. One limitation of our study is that Ishikawa human endometrial adenocarcinoma cell line was used to study benign disease. However, it is one of the few endometrial cell lines that expresses functional ERα and PR^60^. This cancer cell line is modestly responsive to estrogen but has lost its inhibitory response to progesterone despite the expression of receptors for both hormones. Another limitation is the lack of β-catenin-mediated TGF-β2 secretion in our study.

Pirfenidone (5-methyl-1-2-[1H]-pyridone) is a small synthetic molecule that inhibits TGF-β production^61,62^ and is approved by the Federal Drug Administration (FDA) for the treatment of idiopathic pulmonary fibrosis^63^. Our results show that pirfenidone treatment suppresses EMT and cell invasiveness in Ishikawa cells with β-catenin activation. Notably, pirfenidone also inhibits TGF-β1 as well as TNFα and IL-1β^64^. Therefore, the effect of pirfenidone was confirmed by using two other TGF-β inhibitors, GW788388 and R268712. Although these TGF-β inhibitors used in the present work are not specific for TGF-β2, our results suggest that TGF-β2 is a potential therapeutic target for adenomyosis-related β-catenin activation and EMT. Therefore, our study improved the understanding of the molecular mechanisms involved in the early pathogenesis of adenomyosis and will help develop strategic therapies for nonsurgical treatment.

In summary, we identified *Tgf-β2* as a direct target gene of β-catenin in the uterus using transcriptomic and ChIP-seq analyses. The levels of TGF-β2 are higher in endometrial epithelial cells of β-catenin-stabilized mice compared to control mice, as well as in epithelial cells of human eutopic endometrium and adenomyosis lesions compared to women without adenomyosis. We demonstrated a strong positive correlation between β-catenin and TGF-β2 protein levels in women with adenomyosis. β-catenin activation leads to EMT in endometrial epithelial cells through TGF-β2, and TGF-β inhibition suppresses EMT-related β-catenin activation effects. These results provide significant insights into our understanding of the pathophysiological function of β-catenin in adenomyosis development and suggest the therapeutic potential of a TGF-β inhibitor for adenomyosis.

## Supplementary information

Supplementary Information File
