# Supplementary material for: β-catenin activates TGF-β-induced epithelial–mesenchymal transition in adenomyosis
Source: Exp Mol Med. 2020 Oct 15;52(10):1754–65. doi: 10.1038/s12276-020-00514-6 (PMC8080580; doi:10.1038/s12276-020-00514-6)
Supplement: Supplementary file 1 — Supplementary Information File [file 12276_2020_514_MOESM1_ESM.pdf]

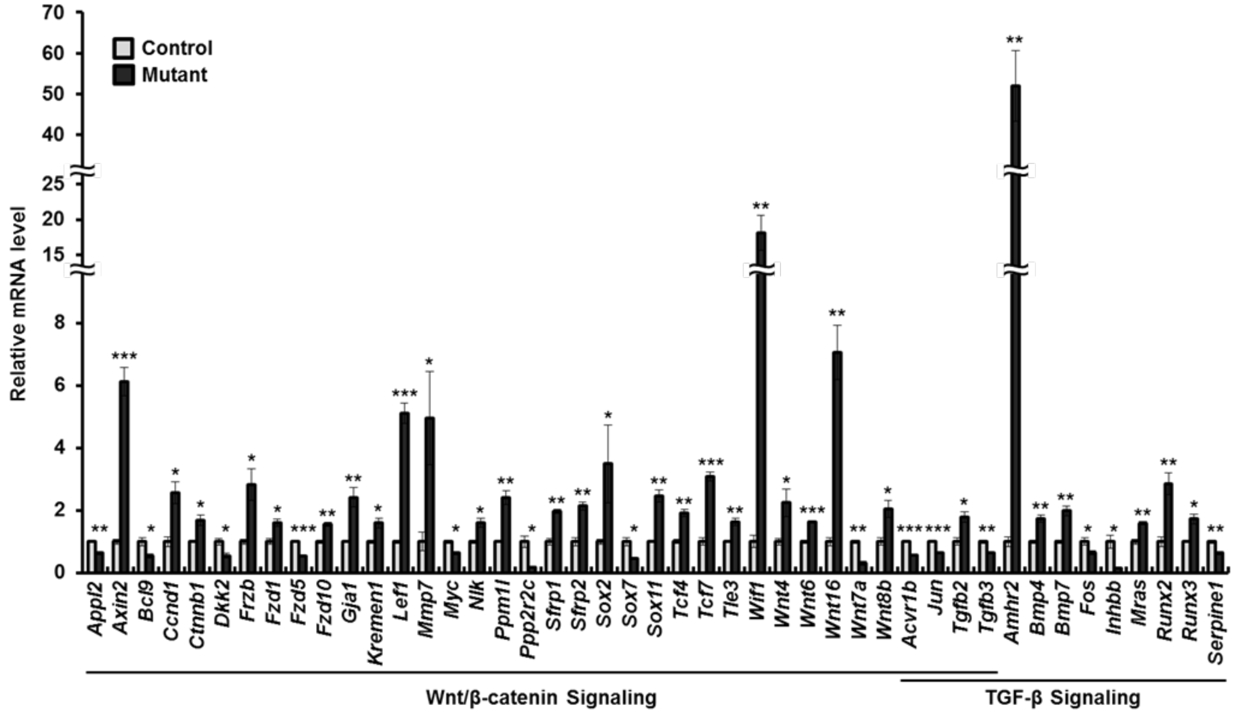

Supplementary Figure S1. Identification of β-catenin-regulated genes for adenomyosis development. Quantitative real-time PCR analysis of genes related to Wnt/β-catenin and TGF-β signaling in uteri of control and mutant mice at 4 weeks of age. The results represent the mean ± 13 SEM. * p<0.05, ** p<0.01 and *** p<0.001.

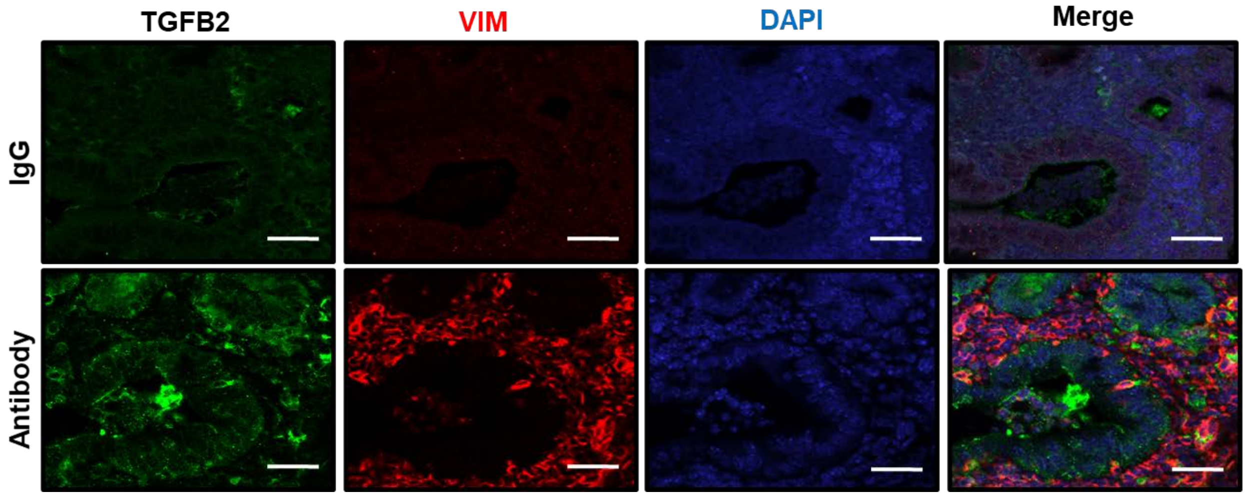

Supplementary Figure S2. Immunofluorescence analysis 24 **of IgG antibody intended for use as a negative control with TGF-β2 or vimentin in the uterus of mutant mice at 4 weeks of age.** Nuclei were counterstained with DAPI. Scale bars represent 50 μm.

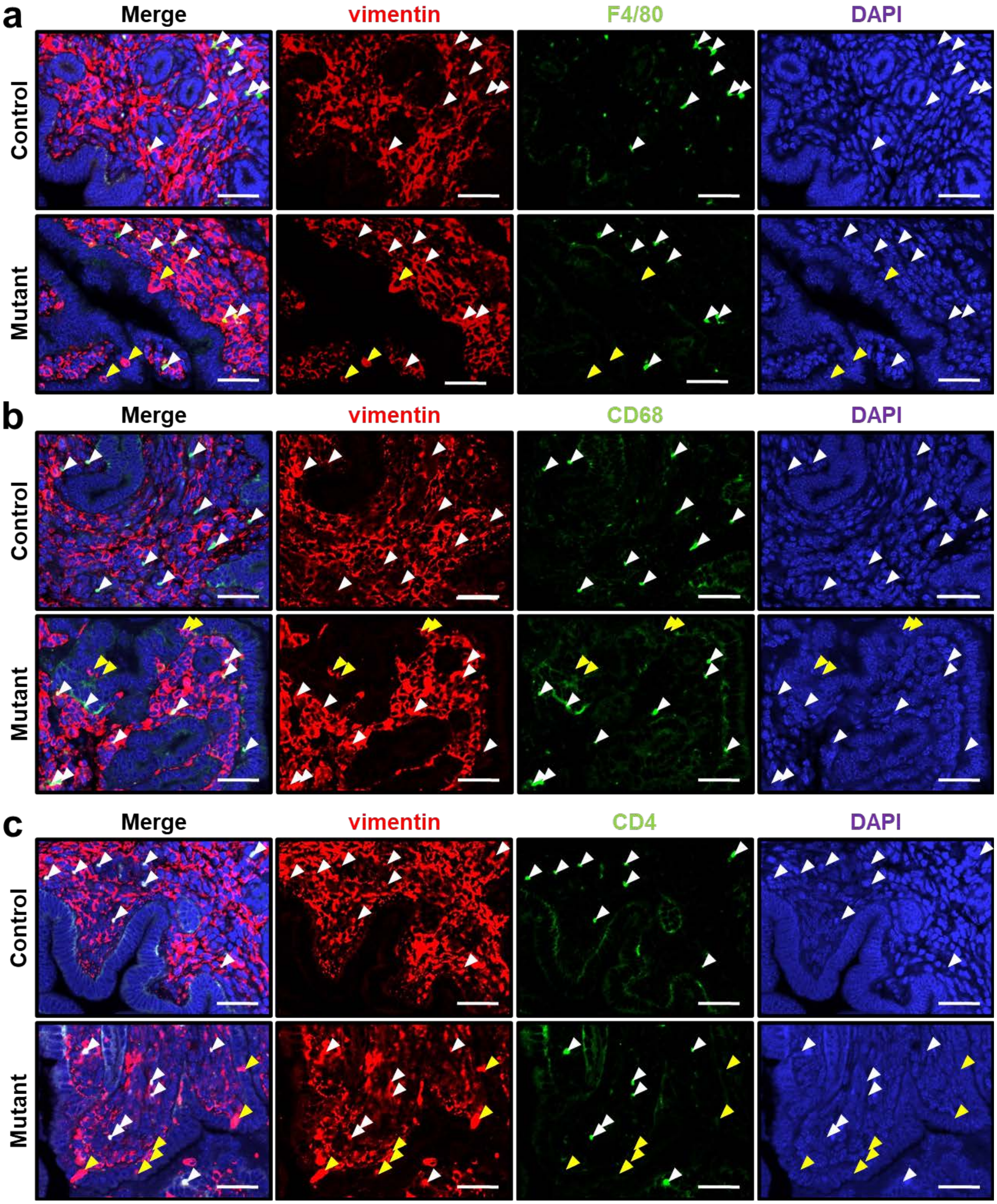

Supplementary Figure S3. Correlation of vimentin expression with F4/80, CD68, or CD4 in mutant mouse uterus. The immunofluorescence analysis of vimentin and F4/80 (a), CD68 (b), or CD4 (c) in the uterus of control and mutant mice at 4 weeks of age. Nuclei were counterstained with DAPI staining. Scale bars represent 50 μm.

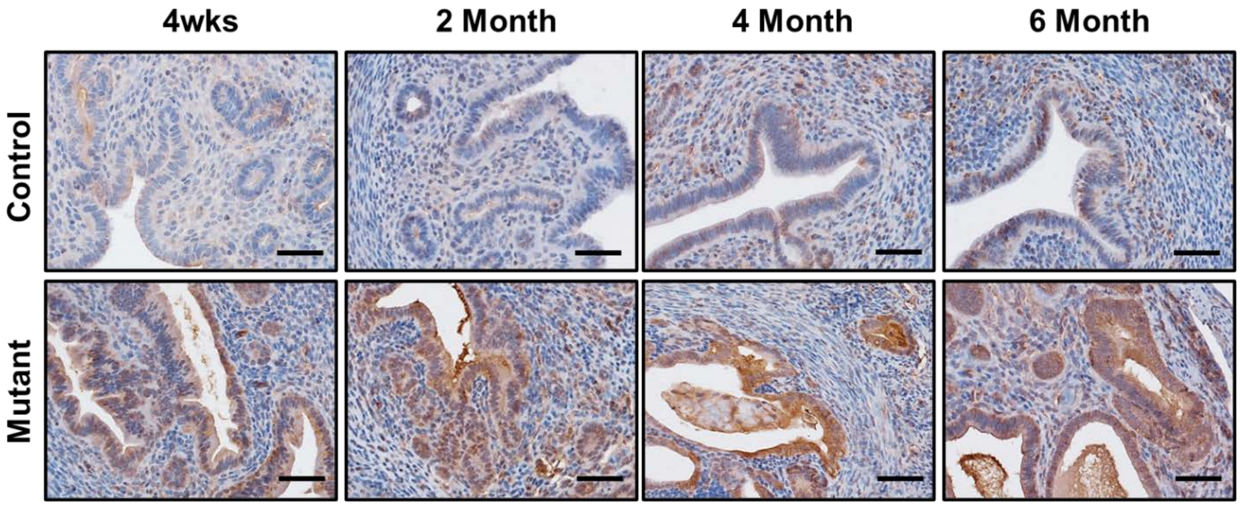

Supplementary Figure S4. Overexpression of TGF-β2 in the murine uterus at 1, 2, 4, and 6 months of age. Immunohistochemical staining of TGF-β2 in uteri of control and mutant mice at 1, 2, 4, and 6 months of age. Scale bars represent 50 μm.

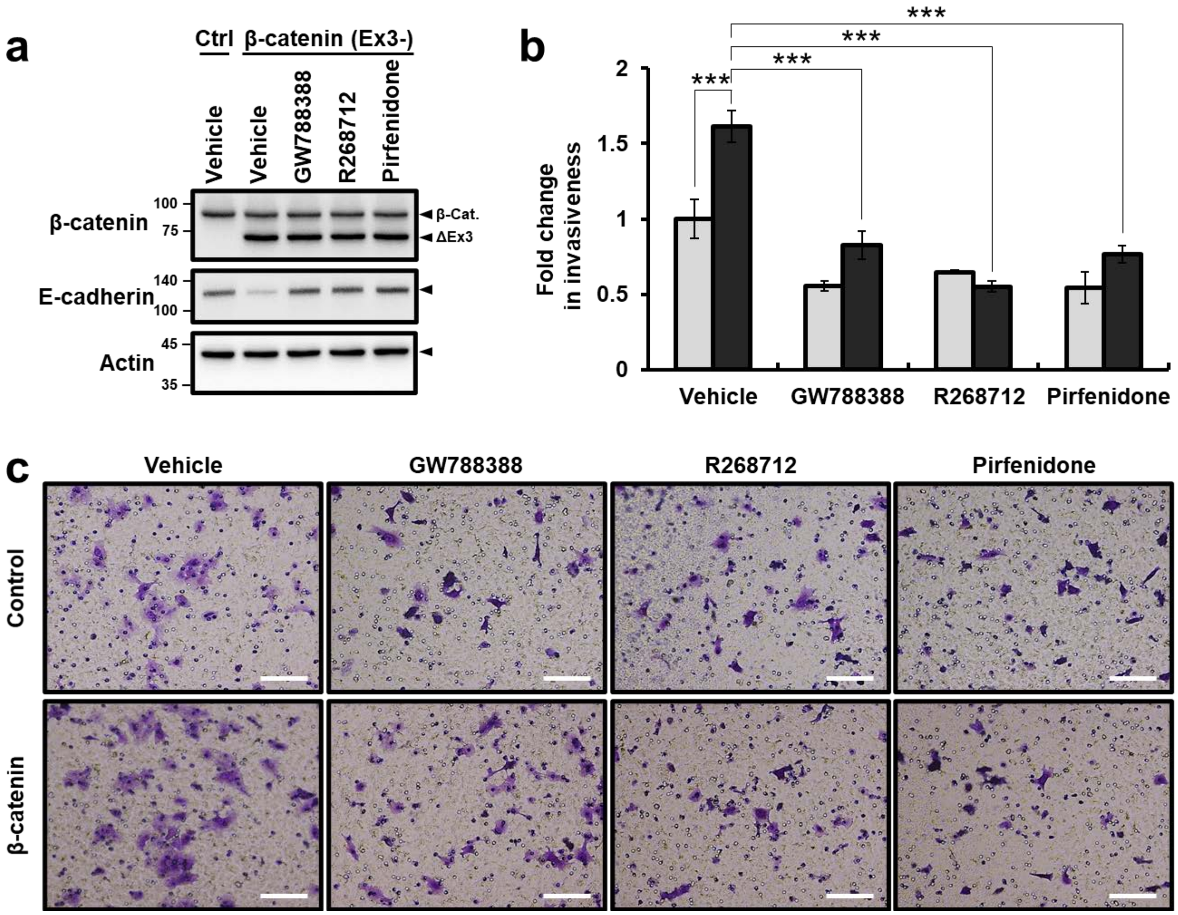

Supplementary Figure S5. Decrease of invasiveness of Ishikawa cells following β-catenin activation by TGF-β inhibitors. (a) The expression of β-catenin and E-cadherin was measured in Ishikawa cells transfected with exon3-deleted β-catenin vector transfected Ishikawa cells treated with vehicle, GW788388, R268712, and pirfenidone over 48 hours using Western blot analysis. (b) Quantification of invasion through the matrigel and transwell membrane control or exon3-deleted β-catenin vector transfected Ishikawa cells treated with vehicle, GW788388, R268712, and pirfenidone over 48 hours. (c) Representative result of transwell invasion assays of control or exon3-deleted β-catenin vector transfected Ishikawa cells treated with vehicle, GW788388, R268712, and pirfenidone over 48 hours. The results represent the mean ± SEM. *** $p<0.001$. Scale bars represent 100 μm.

Table S1. β-catenin target genes in the mutant mice

Accession No.	Symbol	Gene Title	Chr	Gene Start	Gene End	Interval Dists to Start	Interval Pos	Avg Peak
NM_025300	Mrpl15	mitochondrial ribosomal protein L15	1	4,763,290	4,775,791	11311	in gene	13.00
NM_177834	Cpa6	carboxypeptidase A6	1	10,314,808	10,710,024	8808	in gene	6.00
NM_172294	Sulf1	sulfatase 1	1	12,708,626	12,850,453	96078	in gene	5.00
NM_028173	Tram1	translocating chain-associating membrane protein 1	1	13,554,783	13,579,945	-3431	upstream	6.00
NM_026456	Tceb1	transcription elongation factor B (SIII), polypeptide 1	1	16,631,917	16,646,946	-8446	upstream	7.00
NM_026392, NM_027415	Tmem70	transmembrane protein 70	1	16,655,272	16,668,356	120	in gene	7.00
NM_029398	Tmem14a	transmembrane protein 14A	1	21,208,712	21,220,248	-88	upstream	4.00
NM_001012623, NM_001012624, NM_001012625, NM_053270, NM_183018	Rims1	regulating synaptic membrane exocytosis 1	1	22,278,503	22,422,924	147180	downstream	5.00
NM_026719	Lmbrd1	LMBR1 domain containing 1	1	24,685,383	24,823,146	-39, 55625	upstream, in gene	6.50
NM_011200	Ptp4a1	protein tyrosine phosphatase 4a1	1	30,997,148	31,006,600	-568	upstream	8.00
NM_008922	Prim2	DNA primase, p58 subunit	1	33,510,656	33,726,603	-549	upstream	7.00
XR_035294, XR_035301	1700001G17Rik	RIKEN cDNA 1700001G17 gene	1	33,726,669	33,727,559	483	in gene	7.00
NM_011206	Ptpn18	protein tyrosine phosphatase, non-receptor type 18	1	34,516,591	34,530,629	-239	upstream	8.00
XM_982794	EG666290	predicted gene, EG666290	1	36,059,830	36,067,883	11563	downstream	7.00
NM_153408	Lincr	lung-inducible neuralized-related C3HC4 RING domain protein	1	36,321,447	36,330,270	2526	in gene	6.00
NM_026241	Ankrd39	ankyrin repeat domain 39	1	36,595,018	36,604,046	-130, -9362	upstream, upstream	5.00
XM_898566, XM_985534	Sema4c	sema domain, immunoglobulin domain (Ig), transmembrane domain (TM) and short cytoplasmic domain, (semaphorin) 4C	1	36,605,485	36,616,889	12713, 3481	downstream, in gene	5.00
NM_146107	Actr1b	ARP1 actin-related protein 1 homolog B (yeast)	1	36,756,047	36,766,770	-110	upstream	6.00
XM_129743	4933424G06Rik	RIKEN cDNA 4933424G06 gene	1	36,771,383	36,814,805	-4503	upstream	6.00
NM_018872	Tmem131	transmembrane protein 131	1	36,849,034	36,996,372	115284	in gene	6.00
NM_019570	Rev1	REV1 homolog (S. cerevisiae)	1	38,109,631	38,186,507	-677	upstream	5.00
NM_008719	Npas2	neuronal PAS domain protein 2	1	39,251,044	39,419,031	125660, 174225	in gene, downstream	5.00
XM_001477395	LOC100041999	similar to Ribosomal protein L17	1	39,371,721	39,372,339	4983	downstream	5.00
NM_053257	Rpl31	ribosomal protein L31	1	39,424,764	39,428,752	505	in gene	5.00
NM_010553	Il18rap	interleukin 18 receptor accessory protein	1	40,572,208	40,606,150	20165	in gene	5.00
NM_001013025	Tgfbrap1	transforming growth factor, beta receptor associated protein 1	1	43,104,114	43,155,467	10603	in gene	6.00
NM_010212	Fhl2	four and a half LIM domains 2	1	43,179,919	43,220,806	-6010	upstream	6.00
NM_010879	Nck2	non-catalytic region of tyrosine kinase adaptor protein 2	1	43,502,596	43,627,359	56316	in gene	6.00
NM_010863	Myo1b	myosin IB	1	51,806,609	51,972,796	24668	in gene	5.00
NM_001081433	Ankrd44	ankyrin repeat domain 44	1	54,702,184	54,983,202	171618	in gene	7.00
NM_030211	Kctd18	potassium channel tetramerisation domain containing 18	1	58,016,898	58,026,896	272	in gene	7.00
NM_009805, NM_207653	Cflar	CASP8 and FADD-like apoptosis regulator	1	58,770,130	58,815,728	33838	in gene	8.00
NM_018868	Nol5	nucleolar protein 5	1	59,741,850	59,768,354	6	in gene	6.00
XM_001481295, XM_990954	Nbeal1	neurobeachin like 1	1	60,237,443	60,391,923	19901, 162909	in gene, downstream	8.00
NM_030013	Cyp20a1	cytochrome P450, family 20, subfamily A, polypeptide 1	1	60,400,216	60,444,231	136	in gene	10.00
NM_198127	Abi2	abl-interactor 2	1	60,466,642	60,538,002	-253	upstream	7.00
NM_001081050	Pard3b	par-3 partitioning defective 3 homolog B (C. elegans)	1	61,685,817	62,684,497	952743	in gene	5.00
NM_007381	Acadl	acyl-Coenzyme A dehydrogenase, long-chain	1	66,877,427	66,909,841	-79	upstream	9.00
XM_001002143, XM_136682, XM_909965, XM_986918	Erbb4	v-erb-a erythroblastic leukemia viral oncogene homolog 4 (avian)	1	68,086,540	69,154,633	1065401	in gene	5.00
XM_619639, XM_984786	Tns1	tensin 1	1	73,956,805	74,171,021	151677, 29741	in gene, in gene	6.00
NM_001039509, NM_019999, NM_025580	Pnkd	paroxysmal nonkinesiogenic dyskinesia	1	74,331,608	74,400,266	18744	in gene	6.00
NM_027154	Tmbim1	transmembrane BAX inhibitor motif containing 1	1	74,334,828	74,350,734	382	in gene	6.00
XM_001477569, XM_001480060	2410125D13Rik	RIKEN cDNA 2410125D13 gene	1	74,350,254	74,351,143	98	in gene	6.00
NM_013612	Slc11a1	solute carrier family 11 (proton-coupled divalent metal ion transporters), member 1	1	74,421,803	74,432,636	5701	in gene	6.00
NM_153744, NM_153745	Prkag3	protein kinase, AMP-activated, gamma 3 non-catatlytic subunit	1	74,787,214	74,795,436	332	in gene	5.00
NM_194333	Slc23a3	solute carrier family 23 (nucleobase transporters), member 3	1	75,122,115	75,130,464	8672	downstream	7.00
NM_026977	1810031K17Rik	RIKEN cDNA 1810031K17 gene	1	75,131,789	75,138,942	17150	downstream	7.00

Accession No.	Symbol	Gene Title	Chr	Gene Start	Gene End	Interval Dists to Start	Interval Pos	Avg Peak
NM_010043	Des	desmin	1	75,356,919	75,364,291	-151	upstream	6.00
NM_007936	Epha4	Eph receptor A4	1	77,363,760	77,511,663	639	in gene	5.00
XM_001472811	LOC100039330	hypothetical protein LOC100039330	1	77,511,855	77,513,703	-831	upstream	5.00
NM_172849	9430031J16Rik	RIKEN cDNA 9430031J16 gene	1	81,073,892	81,268,239	138108	in gene	7.00
NM_022417	Itm2c	integral membrane protein 2C	1	87,791,140	87,805,267	4812	in gene	7.00
NM_027456, NM_030184	Armc9	armadillo repeat containing 9	1	88,051,355	88,174,859	87989	in gene	7.00
XM_001474704	LOC100040414	similar to ribosomal protein L30	1	88,370,234	88,370,581	-1098	upstream	6.00
NM_008801	Pde6d	phosphodiesterase 6D, cGMP-specific, rod, delta	1	88,439,590	88,479,076	-4604	upstream	5.00
NM_172974	Cops7b	COP9 (constitutive photomorphogenic) homolog, subunit 7b (Arabidopsis thaliana)	1	88,483,675	88,503,075	5	in gene	5.00
NM_153530	4930429A22Rik	RIKEN cDNA 4930429A22 gene	1	88,600,418	88,946,667	147	in gene	5.00
NM_001039169, NM_001039170, NM_023314	Eif4e2	eukaryotic translation initiation factor 4E member 2	1	89,110,489	89,137,063	32199	downstream	5.00
NM_146112	Tnrc15	trinucleotide repeat containing 15	1	89,223,628	89,345,721	5636	in gene	7.00
XM_001475127	LOC100040600	similar to Tnrc15 protein	1	89,223,643	89,224,297	5621	downstream	7.00
NM_029846	Atg16l1	autophagy-related 16-like 1 (yeast)	1	89,652,646	89,688,997	40858	downstream	7.00
NM_009118	Sag	retinal S-antigen	1	89,700,255	89,741,732	-6751, 49857	upstream, downstream	6.50
NM_001033291	Usp40	ubiquitin specific peptidase 40	1	89,841,696	89,905,126	66102	downstream	7.00
XM_001475729	LOC100040766	similar to hCG2012694	1	90,123,595	90,158,864	13317, 17077, 38112	in gene, in gene, downstream	8.67
NM_198652	6430706D22Rik	RIKEN cDNA 6430706D22 gene	1	90,159,684	90,174,095	12388	in gene	7.00
XM_001471840	LOC100038822	hypothetical protein LOC100038822	1	90,161,403	90,174,132	12425	in gene	7.00
XM_001473750, XM_001474136	2410088K16Rik	RIKEN cDNA 2410088K16 gene	1	90,651,471	90,652,310	-3519	upstream	5.00
NM_008515	Lrrfip1	leucine rich repeat (in FLII) interacting protein 1	1	92,950,084	93,025,495	-10148	upstream	6.00
NM_001033292	Espnl	espin-like	1	93,218,652	93,244,880	24932	in gene	5.00
NM_027551	Klhl30	kelch-like 30 (Drosophila)	1	93,247,650	93,258,981	-4066	upstream	5.00
NM_011066	Per2	period homolog 2 (Drosophila)	1	93,312,559	93,355,873	-1183	upstream	6.00
NM_024197	Ndufa10	NADH dehydrogenase (ubiquinone) 1 alpha subcomplex 10	1	94,336,285	94,370,335	-81	upstream	6.00
NM_147038	Olfr1416	olfactory receptor 1416	1	94,376,258	94,377,196	6780	downstream	6.00
NM_011796	Capn10	calpain 10	1	94,830,985	94,844,525	11554	in gene	6.00
NM_172411	2310007B03Rik	RIKEN cDNA 2310007B03 gene	1	95,047,933	95,057,525	1253	in gene	4.00
XM_001474855, XM_977156	E030010N08Rik	RIKEN cDNA E030010N08 gene	1	95,065,302	95,127,651	-9030	upstream	4.00
NM_021537	Stk25	serine/threonine kinase 25 (yeast)	1	95,517,328	95,532,304	-160	upstream	8.00
NM_016778	Bok	Bcl-2-related ovarian killer protein	1	95,582,271	95,592,339	1217, 11889, 19233	in gene, downstream, downstream	6.67
NM_025920	Thap4	THAP domain containing 4	1	95,601,968	95,651,415	57255, 49911	downstream, downstream	7.00
NM_023136	Dtymk	deoxythymidylate kinase	1	95,697,072	95,698,452	-2049	upstream	6.00
NM_013626	Pam	peptidylglycine alpha-amidating monooxygenase	1	99,717,671	99,873,836	61228	in gene	5.00
NM_009257	Serpinb5	serine (or cysteine) peptidase inhibitor, clade B, member 5	1	108,757,757	108,779,925	22723	downstream	6.00
NM_001081276	Clasp1	CLIP associating protein 1	1	120,285,635	120,506,039	66093, 70461	in gene, in gene	6.00
NM_001081125	Gli2	GLI-Kruppel family member GLI2	1	120,730,638	120,950,196	98772, 4	in gene, in gene	6.00
NM_022327	Ralb	v-ral simian leukemia viral oncogene homolog B (ras related)	1	121,366,886	121,401,205	40309	downstream	6.00
NM_133186	Steap3	STEAP family member 3	1	122,123,783	122,161,834	33226, 16778	in gene, in gene	7.00
XM_895892, XM_922706	AI848258	expressed sequence AI848258	1	122,980,884	123,017,009	12892	in gene	4.00
NM_145128	Mgat5	mannoside acetylglucosaminyltransferase 5	1	129,101,563	129,379,549	22960	in gene	6.00
NM_181750	R3hdm1	R3H domain 1 (binds single-stranded nucleic acids)	1	129,999,883	130,134,312	141109	downstream	7.00
NM_026390	Ubxd2	UBX domain containing 2	1	130,140,758	130,175,954	234	in gene	7.00
NM_010016	Cd55	CD55 antigen	1	132,335,606	132,359,317	28341	downstream	5.00
NM_008551	Mapkapk2	MAP kinase-activated protein kinase 2	1	132,950,281	132,994,120	45224, 42920	downstream, in gene	5.50
NM_011924	Avpr1b	arginine vasopressin receptor 1B	1	133,495,691	133,508,577	4021	in gene	5.00
NM_145509	5430435G22Rik	RIKEN cDNA 5430435G22 gene	1	133,585,272	133,610,041	2616, 6568	in gene, in gene	6.00
NM_173865	Slc41a1	solute carrier family 41, member 1	1	133,724,554	133,745,440	14486, 19286	in gene, in gene	6.50
NM_145977	Slc45a3	solute carrier family 45, member 3	1	133,867,186	133,879,541	22254	downstream	5.00
NM_172516	Ripk5	receptor interacting protein kinase 5	1	134,314,030	134,363,536	14594	in gene	6.00
NM_182716	Nfasc	neurofascin	1	134,466,563	134,638,342	38102	in gene	4.00
NM_008575	Mdm4	transformed mouse 3T3 cell double minute 4	1	134,886,422	134,921,925	101	in gene	5.00
XM_001479658, XM_914845	Pik3c2b	phosphoinositide-3-kinase, class 2, beta polypeptide	1	134,962,877	135,004,025	35395	in gene	6.00
NM_133819	Ppp1r15b	protein phosphatase 1, regulatory (inhibitor) subunit 15b	1	135,028,040	135,033,805	14680	downstream	6.00
NM_011439	Sox13	SRY-box containing gene 13	1	135,278,877	135,320,789	1482	in gene	4.00
XM_001479170, XM_990438	EG667414	predicted gene, EG667414	1	136,005,911	136,019,726	-4055	upstream	5.00
NM_009307	Syt2	synaptotagmin II	1	136,543,258	136,645,994	40102, 69670	in gene, in gene	7.00
NM_001081307	Ppp1r12b	protein phosphatase 1, regulatory (inhibitor) subunit 12B	1	136,662,520	136,852,517	182469, 177957	in gene, in gene	7.50

Accession No.	Symbol	Gene Title	Chr	Gene Start	Gene End	Interval Dists to Start	Interval Pos	Avg Peak
NM_173437	Nav1	neuron navigator 1	1	137,335,450	137,482,286	135726, 3182	in gene, in gene	6.00
NM_175447	EG215714	predicted gene, EG215714	1	137,481,350	137,496,527	-2246	upstream	6.00
NM_007791	Csrp1	cysteine and glycine-rich protein 1	1	137,625,774	137,648,806	10418	in gene	13.00
XM_001478282	LOC100042477	similar to general transcription factor II I repeat domain-containing 1	1	137,766,004	137,766,906	-7316	upstream	5.00
NM_019645	Pkp1	plakophilin 1	1	137,767,972	137,815,601	56913	downstream	5.00
XM_001478252, XM_001478294	9830123M21Rik	RIKEN cDNA 9830123M21 gene	1	137,850,155	137,881,876	-9964	upstream	6.00
NM_172643	Zbtb41	zinc finger and BTB domain containing 41 homolog	1	141,318,960	141,345,877	-144	upstream	8.00
NM_009061	Rgs2	regulator of G-protein signaling 2	1	145,847,127	145,851,279	-145	upstream	7.00
NM_001077237, NM_145511	BC003331	cDNA sequence BC003331	1	152,208,441	152,240,185	-23	upstream	7.00
NM_133780	Tpr	translocated promoter region	1	152,239,983	152,297,059	225	in gene	7.00
NM_026876	1190005F20Rik	RIKEN cDNA 1190005F20 gene	1	153,275,778	153,305,289	29134	in gene	5.00
NM_197990	1700025G04Rik	RIKEN cDNA 1700025G04 gene	1	153,739,715	153,937,229	173709	in gene	6.00
NM_010683	Lamc1	laminin, gamma 1	1	155,066,052	155,179,916	69996	in gene	4.00
NM_009230	Soat1	sterol O-acyltransferase 1	1	158,358,239	158,404,459	-117	upstream	5.00
NM_009595	Abl2	v-abl Abelson murine leukemia viral oncogene 2 (arg, Abelson-related gene)	1	158,489,180	158,579,750	10356	in gene	4.00
XM_001472239, XM_917090	LOC433368	similar to HCV NS5A-transactivated protein 9 homolog	1	158,682,411	158,724,518	8117	in gene	4.00
NM_023884	Ralgps2	Ral GEF with PH domain and SH3 binding motif 2	1	158,738,253	158,869,711	751	in gene	5.00
XM_001472276, XM_001479264	4930439D14Rik	RIKEN cDNA 4930439D14 gene	1	158,868,925	158,870,326	35	in gene	5.00
NM_022312	Tnr	tenascin R	1	161,453,900	161,854,606	347348	in gene	8.00
NM_001038621, NM_013862	Rabgap1l	RAB GTPase activating protein 1-like	1	162,149,305	162,723,069	494301	in gene	7.00
NM_001039482	Klhl20	kelch-like 20 (Drosophila)	1	163,018,509	163,061,610	-22	upstream	8.00
XM_001479494, XM_975051	AI848100	expressed sequence AI848100	1	163,746,243	163,807,276	1356	in gene	6.00
NM_008030	Fmo3	flavin containing monooxygenase 3	1	164,883,931	164,914,622	5086	in gene	7.00
NM_010629	Kifap3	kinesin-associated protein 3	1	165,709,810	165,847,231	114350	in gene	6.00
NM_001081126	Gpr161	G protein-coupled receptor 161	1	167,225,897	167,251,982	25991	in gene	10.00
XM_917541, XM_976438	Iqwd1	IQ motif and WD repeats 1	1	167,257,458	167,390,596	138708, 82708, 345	downstream, in gene, in gene	6.67
NM_027430	Brp44	brain protein 44	1	167,391,339	167,411,345	-1088, 18565	upstream, in gene	4.00
NM_173029	Sacy	soluble adenylyl cyclase	1	167,415,314	167,506,904	-5410, 96014	upstream, downstream	5.00
NM_175461	C030014K22Rik	RIKEN cDNA C030014K22 gene	1	168,931,606	169,021,140	1148	in gene	4.00
NM_001039483	Tmco1	transmembrane and coiled-coil domains 1	1	169,238,801	169,264,109	28890	downstream	4.00
NM_008783, NM_183355	Pbx1	pre B-cell leukemia transcription factor 1	1	170,049,495	170,362,389	56773, 15349, -427	in gene, in gene, upstream	7.33
NM_023284	Nuf2	NUF2, NDC80 kinetochore complex component, homolog (S. cerevisiae)	1	171,428,065	171,461,595	37275	downstream	4.00
NM_022563	Ddr2	discoidin domain receptor family, member 2	1	171,907,777	172,040,690	45362	in gene	6.00
XM_001475554	LOC100040571	hypothetical protein LOC100040571	1	172,004,334	172,005,222	-9006	upstream	6.00
NM_023173	Dusp12	dual specificity phosphatase 12	1	172,804,319	172,815,671	3943	in gene	6.00
NM_001029984	Fcrlb	Fc receptor-like B	1	172,837,404	172,843,072	-1205	upstream	4.00
NM_145141	Fcrla	Fc receptor-like A	1	172,848,017	172,857,671	13394	downstream	4.00
XM_484933, XM_917594	Pcp4l1	Purkinje cell protein 4-like 1	1	173,103,390	173,127,166	926	in gene	4.00
NM_020579	B4galt3	UDP-Gal:betaGlcNAc beta 1,4-galactosyltransferase, polypeptide 3	1	173,200,519	173,207,026	-6087	upstream	4.00
NM_018729	Cd244	CD244 natural killer cell receptor 2B4	1	173,489,324	173,515,447	-364	upstream	5.00
NM_033509	Vangl2	vang-like 2 (van gogh, Drosophila)	1	173,934,502	173,957,399	5431	in gene	5.00
XM_001474255	LOC100040199	similar to Ubtf protein	1	173,959,277	173,964,464	-7309	upstream	5.00
NM_019445	Fmn2	formin 2	1	176,419,303	176,752,208	35673	in gene	7.00
	B230369F24Rik	RIKEN cDNA B230369F24 gene	1	180,245,164	180,245,590	-3731	upstream	5.00
XM_001473597, XM_001478186	LOC669454	similar to RIKEN cDNA B230369F24 gene	1	180,247,224	180,249,233	2097	downstream	5.00
NM_025511	2310005N03Rik	RIKEN cDNA 2310005N03 gene	1	180,249,284	180,252,824	37	in gene	5.00
NM_026626	Efcab2	EF-hand calcium binding domain 2	1	180,336,012	180,413,381	45012	in gene	5.00
NM_177724	D230039L06Rik	RIKEN cDNA D230039L06 gene	1	180,459,256	180,646,631	-1560, 111848	upstream, in gene	5.00
NM_027188	Smyd3	SET and MYND domain containing 3	1	180,885,162	181,448,134	538982	in gene	7.00
NM_001081175	Itpkb	inositol 1,4,5-trisphosphate 3-kinase B	1	182,260,607	182,353,790	89649	in gene	7.00
NM_177099	Lefty2	Left-right determination factor 2	1	182,823,250	182,829,234	-3090	upstream	6.00
NM_012058	Srp9	signal recognition particle 9	1	184,054,881	184,062,519	-9409	upstream	5.00
XM_001472215	LOC100039079	similar to Snf5p	1	186,686,428	186,690,074	1647	in gene	5.00
NM_178692	C130074G19Rik	RIKEN cDNA C130074G19 gene	1	186,695,805	186,706,915	18488	downstream	5.00
NM_009367	Tgfb2	transforming growth factor, beta 2	1	188,447,521	188,529,868	-2580	upstream	6.00
NM_028848	Spata17	spermatogenesis associated 17	1	188,872,285	189,039,344	0	in gene	7.00
NM_026367	Gpatch2	G patch domain containing 2	1	189,039,418	189,175,324	-74	upstream	7.00
NM_026796	Smyd2	SET and MYND domain containing 2	1	191,704,371	191,746,167	55	in gene	7.00
NM_008937	Prox1	prospero-related homeobox 1	1	191,945,654	191,994,559	41119, -1473	in gene, upstream	6.00
NM_007498	Atf3	activating transcription factor 3	1	192,994,175	193,007,212	-148	upstream	7.00
XR_035313, XR_035377	A730013G03Rik	RIKEN cDNA A730013G03 gene	1	194,659,313	194,661,407	10639, 5615	downstream, downstream	4.50
NM_198247	Sertad4	SERTA domain containing 4	1	194,670,682	194,681,915	-85	upstream	5.00

Accession No.	Symbol	Gene Title	Chr	Gene Start	Gene End	Interval Dists to Start	Interval Pos	Avg Peak
NM_145415	AA408296	expressed sequence AA408296	1	194,930,597	194,956,445	-67	upstream	11.00
NM_010778	Cd46	CD46 antigen, complement regulatory protein	1	196,868,094	196,918,442	29898	in gene	6.00
NM_172475	Frmd4a	FERM domain containing 4A	2	4,073,918	4,534,988	260738	in gene	4.00
NM_177343	Camk1d	calcium/calmodulin-dependent protein kinase ID	2	5,214,503	5,635,710	314750, 77246	in gene, in gene	5.00
NM_001081131	Dhtkd1	dehydrogenase E1 and transketolase domain containing 1	2	5,817,140	5,863,838	-8418	upstream	11.00
NM_001081132	Upf2	UPF2 regulator of nonsense transcripts homolog (yeast)	2	5,872,515	5,977,749	-259	upstream	11.00
NM_010582	Itih2	inter-alpha trypsin inhibitor, heavy chain 2	2	10,016,218	10,052,290	-9998	upstream	6.00
NM_152824	Rbm17	RNA binding motif protein 17	2	11,507,066	11,524,826	26	in gene	5.00
NM_010067	Trdmt1	tRNA aspartic acid methyltransferase 1	2	13,431,791	13,466,291	291	in gene	7.00
NM_011484	Stam	signal transducing adaptor molecule (SH3 domain and ITAM motif) 1	2	13,995,739	14,069,965	-91	upstream	6.00
NM_008845	Pip5k2a	phosphatidylinositol-4-phosphate 5-kinase, type II, alpha	2	18,763,883	18,919,748	67348, 29620	in gene, in gene	6.00
NM_001042528, NM_007579	Cacna1b	calcium channel, voltage-dependent, N type, alpha 1B subunit	2	24,461,895	24,618,672	1424	in gene	5.00
NM_178408	Arrdc1	arrestin domain containing 1	2	24,780,872	24,790,683	15867	downstream	7.00
NM_028093	Entpd8	ectonucleoside triphosphate diphosphohydrolase 8	2	24,935,843	24,941,239	11341	downstream	5.00
NM_172204	Noxa1	NADPH oxidase activator 1	2	24,941,187	24,950,668	3484	in gene	5.00
NM_009849	Entpd2	ectonucleoside triphosphate diphosphohydrolase 2	2	25,251,478	25,256,838	234	in gene	6.00
NM_008721	Npdc1	neural proliferation, differentiation and control gene 1	2	25,258,603	25,265,014	-6891	upstream	6.00
NM_009422	Traf2	Tnf receptor-associated factor 2	2	25,373,502	25,402,414	9326, -82	in gene, upstream	6.00
NM_021519	Edf1	endothelial differentiation-related factor 1	2	25,413,420	25,417,598	-236	upstream	6.00
XM_358556, XM_913936	C330006A16Rik	RIKEN cDNA C330006A16 gene	2	25,992,331	25,995,853	461, -1779	in gene, upstream	7.00
NM_153559	Qscn6l1	quiescin Q6-like 1	2	26,064,650	26,092,940	5868	in gene	5.00
NM_153410	Gpsm1	G-protein signalling modulator 1 (AGS3-like, C. elegans)	2	26,171,060	26,203,755	36716	downstream	5.00
NM_026828	D2Bwg1335e	DNA segment, Chr 2, Brigham & Women's Genetics 1335 expressed	2	26,203,643	26,207,630	-146	upstream	5.00
XM_285326, XM_914859	Card9	caspase recruitment domain family, member 9	2	26,207,832	26,215,067	7291	downstream	5.00
NM_178444, NM_198724, NM_198725	Egfl7	EGF-like domain 7	2	26,436,603	26,448,202	-9371	upstream	5.00
NM_011512	Surf4	surfeit gene 4	2	26,775,559	26,789,448	616	in gene	7.00
NM_198628	Gm711	gene model 711, (NCBI)	2	26,789,589	26,809,016	-757	upstream	7.00
NM_029981	Adamtsl2	ADAMTS-like 2	2	26,934,901	26,964,133	13723	in gene	5.00
NM_138942	Dbh	dopamine beta hydroxylase	2	27,021,027	27,038,724	-6827	upstream	4.00
NM_009500	Vav2	vav 2 oncogene	2	27,119,155	27,282,347	35387	in gene	5.00
NM_015734	Col5a1	procollagen, type V, alpha 1	2	27,742,205	27,893,044	106819	in gene	6.00
NM_027040	1700007K13Rik	RIKEN cDNA 1700007K13 gene	2	28,317,521	28,321,844	-1980	upstream	5.00
NM_080452	Mrps2	mitochondrial ribosomal protein S2	2	28,323,586	28,326,697	238	in gene	5.00
NM_009058	Ralgds	ral guanine nucleotide dissociation stimulator	2	28,368,851	28,408,590	12504, 38685	in gene, in gene	6.50
NM_009885	Cel	carboxyl ester lipase	2	28,411,339	28,418,882	11346	downstream	7.00
NM_148928	Gtf3c5	general transcription factor IIIC, polypeptide 5	2	28,421,835	28,438,766	302	in gene	5.00
NM_008114	Gfi1b	growth factor independent 1B	2	28,464,977	28,477,464	-8168	upstream	5.00
NM_001033874	1190002A17Rik	RIKEN cDNA 1190002A17 gene	2	28,555,684	28,668,678	99628	in gene	9.00
NM_172977	Gtf3c4	general transcription factor IIIC, polypeptide 4	2	28,681,095	28,695,806	3582	in gene	6.00
NM_001033294	Ddx31	DEAD/H (Asp-Glu-Ala-Asp/His) box polypeptide 31	2	28,696,257	28,761,090	-4033	upstream	6.00
NM_019446	Barhl1	BarH-like 1 (Drosophila)	2	28,763,199	28,771,941	-1291	upstream	6.00
XM_986644, XM_994340	1700101E01Rik	RIKEN cDNA 1700101E01 gene	2	28,810,893	28,910,586	83690, 31034	in gene, in gene	6.50
NM_198033	Setx	senataxin	2	28,980,512	29,037,991	56363	in gene	4.00
NM_026896	Crsp8	cofactor required for Sp1 transcriptional activation, subunit 8	2	29,202,356	29,380,308	76156	in gene	6.00
NM_207298	Ceecam1	cerebral endothelial cell adhesion molecule 1	2	29,725,014	29,738,360	20154, 21354	downstream, downstream	5.00
NM_013615	Odf2	outer dense fiber of sperm tails 2	2	29,745,620	29,778,059	-452, 748	upstream, in gene	5.00
NM_028923	Gle1l	GLE1 RNA export mediator-like (yeast	2	29,790,947	29,814,175	30344	downstream	4.00
XM_977598, XM_990997	LOC665532	hypothetical protein LOC665532	2	29,821,114	29,822,669	177	in gene	4.00
NM_153805	Pkn3	protein kinase N3	2	29,934,286	29,946,539	15090	downstream	8.00
NM_001037762, NM_025428	Zdhhc12	zinc finger, DHHC domain containing 12	2	29,946,464	29,949,155	-221	upstream	8.00
NM_178694	Zer1	zer-1 homolog (C. elegans)	2	29,952,803	29,979,974	30598, -133	downstream, upstream	8.50
XM_001477522	LOC100041114	hypothetical protein LOC100041114	2	29,979,592	29,981,849	515	in gene	9.00
NM_146252	Tbc1d13	TBC1 domain family, member 13	2	29,989,391	30,007,533	-9284	upstream	9.00
NM_007931	Endog	endonuclease G	2	30,027,044	30,029,589	6668	downstream	6.00
NM_172660	D2Wsu81e	DNA segment, Chr 2, Wayne State University 81, expressed	2	30,028,993	30,033,975	263	in gene	6.00
XM_978420	EG665645	predicted gene, EG665645	2	30,628,164	30,638,926	-4620	upstream	5.00
NM_009116	Prrx2	paired related homeobox 2	2	30,700,801	30,736,773	19423	in gene	5.00

Accession No.	Symbol	Gene Title	Chr	Gene Start	Gene End	Interval Dists to Start	Interval Pos	Avg Peak
NM_028846	Usp20	ubiquitin specific peptidase 20	2	30,851,562	30,879,106	8742	in gene	5.00
NM_019681	Freq	frequenin homolog (Drosophila)	2	31,101,443	31,150,992	-2163, 50653	upstream, downstream	5.50
NM_001033389	Fubp3	far upstream element (FUSE) binding protein 3	2	31,428,337	31,473,039	37391	in gene	5.00
NM_011836	Lamc3	laminin gamma 3	2	31,742,801	31,802,055	6111	in gene	5.00
NM_145521	Ppapdc3	phosphatidic acid phosphatase type 2 domain containing 3	2	31,951,171	31,966,340	6965	in gene	4.00
NM_172661	5830434P21Rik	RIKEN cDNA 5830434P21 gene	2	32,037,978	32,090,045	24187	in gene	4.00
NM_145145	Pomt1	protein-O-mannosyltransferase 1	2	32,092,203	32,110,525	10421	in gene	5.00
NM_011675	Uck1	uridine-cytidine kinase 1	2	32,110,522	32,115,581	12957	downstream	5.00
NM_146118	Slc25a25	solute carrier family 25 (mitochondrial carrier, phosphate carrier), member 25	2	32,270,000	32,306,942	28638	in gene	7.00
NM_198191	Pip5kl1	phosphatidylinositol-4-phosphate 5-kinase-like 1	2	32,431,339	32,439,299	14229	downstream	5.00
NM_011373	St6galnac4	ST6 (alpha-N-acetyl-neuraminyl-2,3-beta-galactosyl-1,3)-N-acetylgalactosaminide alpha-2,6-sialyltransferase 4	2	32,443,010	32,456,002	2558	in gene	5.00
NM_001025310, NM_001025311, NM_016973	St6galnac6	ST6 (alpha-N-acetyl-neuraminyl-2,3-beta-galactosyl-1,3)-N-acetylgalactosaminide alpha-2,6-sialyltransferase 6	2	32,455,229	32,476,324	-9661	upstream	5.00
NM_007932	Eng	endoglin	2	32,502,115	32,537,638	333, 9389	in gene, in gene	6.50
NM_010236	Fpgs	folylpolyglutamyl synthetase	2	32,538,129	32,549,652	-700	upstream	6.00
NM_130860	Cdk9	cyclin-dependent kinase 9 (CDC2-related kinase)	2	32,562,958	32,568,305	-2687	upstream	5.00
NM_199302	Lrsam1	leucine rich repeat and sterile alpha motif containing 1	2	32,780,740	32,816,771	-717	upstream	6.00
NR_002898	Snora65	small nucleolar RNA, H/ACA box 65	2	32,818,975	32,819,574	-1487	upstream	6.00
NM_175211	Ralgps1	Ral GEF with PH domain and SH3 binding motif 1	2	32,992,493	33,226,998	240758, 182486, 15793	downstream, in gene, in gene	4.67
NM_010725	Lmx1b	LIM homeobox transcription factor 1 beta	2	33,420,156	33,496,031	82047	downstream	5.00
NM_175184	2610528K11Rik	RIKEN cDNA 2610528K11 gene	2	33,585,476	33,743,466	92090	in gene	9.00
NM_177345	Mapkap1	mitogen-activated protein kinase associated protein 1	2	34,287,545	34,480,470	138503	in gene	6.00
NM_028921	Ttll11	tubulin tyrosine ligase-like family, member 11	2	35,607,467	35,796,326	130598, 128134	in gene, in gene	6.00
NM_026434	Rbm18	RNA binding motif protein 18	2	35,971,601	35,992,131	-157	upstream	5.00
XM_001479253	LOC100042236	hypothetical protein LOC100042236	2	35,991,488	35,994,337	2049	in gene	5.00
NM_026422	Mrrf	mitochondrial ribosome recycling factor	2	35,992,187	36,045,390	101	in gene	5.00
NM_199025	Zbtb26	zinc finger and BTB domain containing 26	2	37,287,688	37,298,641	-90	upstream	5.00
NM_001033960, NM_146121	Rabgap1	RAB GTPase activating protein 1	2	37,298,805	37,421,957	-74	upstream	5.00
NM_010264	Nr6a1	nuclear receptor subfamily 6, group A, member 1	2	38,578,890	38,783,208	167592	in gene	6.00
XM_001475741	LOC100041071	hypothetical protein LOC100041071	2	38,852,257	38,853,805	-6979, -9603	upstream, upstream	6.50
NM_029687	1700123D08Rik	RIKEN cDNA 1700123D08 gene	2	38,852,996	38,861,688	7788, 10412	in gene, downstream	6.50
NM_025592	Rpl35	ribosomal protein L35	2	38,857,101	38,860,651	-133, -2757	upstream, upstream	6.50
NM_028809	Arpc5l	actin related protein 2/3 complex, subunit 5-like	2	38,863,659	38,871,392	-2875, -251	upstream, upstream	6.50
NM_029793	Golga1	golgi autoantigen, golgin subfamily a, 1	2	38,872,023	38,920,926	57518	downstream	5.00
NM_178778	A930041I02Rik	RIKEN cDNA A930041I02 gene	2	38,921,734	39,046,250	16394	in gene	5.00
NM_001025377, NM_153820	Arhgap15	Rho GTPase activating protein 15	2	43,604,344	44,243,143	399336	in gene	9.00
NM_015753	Zeb2	zinc finger E-box binding homeobox 2	2	44,839,154	44,968,799	51007	in gene	5.00
NM_177139	Lypd6	LY6/PLAUR domain containing 6	2	49,983,890	50,049,084	-7986	upstream	6.00
XM_130232, XM_976519	Neb	nebulin	2	51,991,953	52,194,318	100782	in gene	5.00
XM_894739, XM_905830	OTTMUSG00000012638	predicted gene, OTTMUSG00000012638	2	52,475,248	52,476,144	4368	downstream	6.00
NM_018785	Prpf40a	PRP40 pre-mRNA processing factor 40 homolog A (yeast)	2	52,997,509	53,050,221	1093	in gene	4.00
NM_022989	Arl6ip6	ADP-ribosylation factor-like 6 interacting protein 6	2	53,051,145	53,078,060	-2017	upstream	4.00
NM_008867	Pla2r1	phospholipase A2 receptor 1	2	60,257,096	60,391,318	1878	in gene	6.00
NM_020296	Rbms1	RNA binding motif, single stranded interacting protein 1	2	60,590,779	60,800,977	117041, 100961	in gene, in gene	6.50
NM_001081088	Lrp2	low density lipoprotein receptor-related protein 2	2	69,262,392	69,424,124	79377	in gene	5.00
NM_177784	Klhl23	kelch-like 23 (Drosophila)	2	69,660,530	69,674,705	398	in gene	5.00
NM_009278	Ssb	Sjogren syndrome antigen B	2	69,699,685	69,709,861	15739	downstream	5.00
NM_029280	Mettl5	methyltransferase like 5	2	69,715,805	69,723,661	8237	downstream	5.00
XR_002332, XR_005156	4930550G17Rik	RIKEN cDNA 4930550G17 gene	2	69,723,552	69,724,697	9273	downstream	5.00
NM_022435	Sp5	trans-acting transcription factor 5	2	70,312,980	70,315,783	-372, 1756	upstream, in gene	5.50
NM_025744	4933404M02Rik	RIKEN cDNA 4933404M02 gene	2	70,346,876	70,378,941	39724	downstream	4.00
NM_172664	Tlk1	tousled-like kinase 1	2	70,550,464	70,663,537	106257	in gene	7.00
XM_984549, XM_992510	1700109F18Rik	RIKEN cDNA 1700109F18 gene	2	74,548,101	74,550,583	11767, 6551, 2807	downstream, downstream, downstream	6.33
NM_010468	Hoxd3	homeo box D3	2	74,550,050	74,586,328	-6018, -2274, 23561, 41614	upstream, upstream, in gene, downstream	5.75

Accession No.	Symbol	Gene Title	Chr	Gene Start	Gene End	Interval Dists to Start	Interval Pos	Avg Peak
NM_010469	Hoxd4	homeo box D4	2	74,560,035	74,567,217	13576	downstream	5.00
	Mirn10b	microRNA 10b	2	74,564,126	74,564,193	9485	downstream	5.00
XM_984655, XM_992575	LOC667804	hypothetical protein LOC667804	2	74,588,730	74,591,799	2934	in gene	5.00
NM_010467	Hoxd1	homeo box D1	2	74,601,037	74,603,199	-9373	upstream	5.00
NM_010902	Nfe2l2	nuclear factor, erythroid derived 2, like 2	2	75,513,576	75,542,698	1034	in gene	8.00
XM_001475569, XM_001476920	E030042O20Rik	RIKEN cDNA E030042O20 gene	2	75,542,792	75,548,082	-1128	upstream	8.00
NM_001025576	2610301F02Rik	RIKEN cDNA 2610301F02 gene	2	76,883,028	77,008,693	85, -1131	in gene, upstream	7.50
NM_178723	Zfp533	zinc finger protein 533	2	77,249,157	77,654,873	179033	in gene	6.00
NM_026934	Zc3h15	zinc finger CCCH-type containing 15	2	83,484,735	83,504,774	-159	upstream	8.00
NM_008402	Itgav	integrin alpha V	2	83,564,672	83,643,964	55968	in gene	7.00
NM_011576	Tfpi	tissue factor pathway inhibitor	2	84,273,017	84,314,331	-7941	upstream	5.00
NM_133840	Clp1	CLP1, cleavage and polyadenylation factor I subunit, homolog (S. cerevisiae)	2	84,563,279	84,567,425	-7071	upstream	4.00
NM_001005342	Ypel4	yippee-like 4 (Drosophila)	2	84,574,361	84,578,034	135	in gene	4.00
	Mirn130a	microRNA 130a	2	84,581,271	84,581,334	6838	downstream	4.00
NM_182990	Ssrp1	structure specific recognition protein 1	2	84,877,358	84,887,268	10722	downstream	6.00
NM_001081260	Tnks1bp1	tankyrase 1 binding protein 1	2	84,888,928	84,917,286	-848	upstream	6.00
NM_011355	Sfpi1	SFFV proviral integration 1	2	90,936,954	90,955,913	4758	in gene	6.00
NM_013839	Nr1h3	nuclear receptor subfamily 1, group H, member 3	2	91,024,219	91,035,192	-7688	upstream	7.00
NM_007387	Acp2	acid phosphatase 2, lysosomal	2	91,043,087	91,052,300	-207	upstream	7.00
NM_028119	Ddb2	damage specific DNA binding protein 2	2	91,052,013	91,077,135	34255, 223, -5361	downstream, in gene, upstream	5.67
NM_028733, NM_030880	Pacsin3	protein kinase C and casein kinase substrate in neurons 3	2	91,092,758	91,104,835	4810, 8402	in gene, in gene	5.00
NM_023854	Zfp289	zinc finger protein 289	2	91,105,463	91,117,088	-7895, -4303	upstream, upstream	5.00
NM_001080754, NM_172669	D030051N19Rik	RIKEN cDNA D030051N19 gene	2	91,570,295	91,759,006	191545	downstream	6.00
NM_007699	Chrm4	cholinergic receptor, muscarinic 4	2	91,767,406	91,768,845	-5566	upstream	6.00
NM_001012335, NM_001012336, NM_010784	Mdk	midkine	2	91,769,978	91,772,439	10599	downstream	6.00
NM_138306	Dgkz	diacylglycerol kinase zeta	2	91,772,983	91,803,756	3612	in gene	7.00
NM_011957	Creb3l1	cAMP responsive element binding protein 3-like 1	2	91,822,485	91,864,327	11255	in gene	6.00
NM_172670	Gyltl1b	glycosyltransferase-like 1B	2	92,205,209	92,211,168	-9552	upstream	5.00
NM_145122	Pex16	peroxisome biogenesis factor 16	2	92,215,655	92,221,374	5065	in gene	5.00
NM_183112	1700029I15Rik	RIKEN cDNA 1700029I15 gene	2	92,223,075	92,223,758	-2355	upstream	5.00
NM_011162	Mapk8ip1	mitogen activated protein kinase 8 interacting protein 1	2	92,223,837	92,241,420	20700	downstream	5.00
NM_009963	Cry2	cryptochrome 2 (photolyase-like)	2	92,243,803	92,274,185	10089	in gene	7.00
NM_183180	Tspan18	tetraspanin 18	2	93,041,917	93,174,607	50847, 1663, -7473	in gene, in gene, upstream	6.33
XM_001478824	LOC100042789	hypothetical protein LOC100042789	2	93,151,420	93,174,812	1868, -7268	in gene, upstream	6.50
NM_183220	2610203E10Rik	RIKEN cDNA 2610203E10 gene	2	93,675,610	93,689,933	-2763	upstream	4.00
NM_001033452	Gm1967	gene model 1967, (NCBI)	2	93,695,520	93,709,260	16564	downstream	4.00
NM_178886	Ldlrad3	low density lipoprotein receptor class A domain containing 3	2	101,790,358	102,026,534	122198	in gene	5.00
NM_001039150, NM_001039151, NM_009851	Cd44	CD44 antigen	2	102,651,298	102,741,822	2110	in gene	4.00
NM_010125	Elf5	E74-like factor 5	2	103,264,234	103,291,142	12694	in gene	6.00
NM_009804	Cat	catalase	2	103,294,160	103,325,277	2077	in gene	5.00
NM_178890	Abtb2	ankyrin repeat and BTB (POZ) domain containing 2	2	103,406,467	103,558,580	717	in gene	5.00
NM_153126	Nat10	N-acetyltransferase 10	2	103,561,416	103,601,407	-129	upstream	15.00
NM_016739	Caprin1	cell cycle associated protein 1	2	103,605,176	103,637,754	36218, 706, -390	downstream, in gene, upstream	9.00
NM_009037	Rcn1	reticulocalbin 1	2	105,226,105	105,239,476	-236	upstream	6.00
NM_133749	2900064A13Rik	RIKEN cDNA 2900064A13 gene	2	112,295,182	112,307,593	-78	upstream	7.00
NM_033524	Spred1	sprouty protein with EVH-1 domain 1, related sequence	2	116,947,186	117,005,073	-594	upstream	4.00
NM_009273	Srp14	signal recognition particle 14	2	118,301,579	118,305,432	-7400	upstream	7.00
NM_001033254	Pak6	p21 (CDKN1A)-activated kinase 6	2	118,489,039	118,522,972	14737	in gene	5.00
NM_026929	Chac1	ChaC, cation transport regulator-like 1 (E. coli)	2	119,176,992	119,180,117	-288	upstream	5.00
NM_026574	Inoc1	INO80 complex homolog 1 (S. cerevisiae)	2	119,198,778	119,303,365	64085	in gene	6.00
NM_001042653	Oip5	Opa interacting protein 5	2	119,435,268	119,444,241	5169	in gene	5.00
NM_001042652, NM_133851	Nusap1	nucleolar and spindle associated protein 1	2	119,444,034	119,475,896	-4962	upstream	5.00
NM_019392	Tyro3	TYRO3 protein tyrosine kinase 3	2	119,625,250	119,643,840	19182	downstream	6.00
NM_030234	Wdr76	WD repeat domain 76	2	121,332,459	121,370,596	42485	downstream	6.00
NM_172673	Frmd5	FERM domain containing 5	2	121,371,265	121,632,793	257849, 228089	in gene, in gene	6.00
NM_008545	Mageb3	melanoma antigen, family B, 3	2	121,779,507	121,781,828	-188	upstream	4.00
NM_212450	Ctdspl2	CTD (carboxy-terminal domain, RNA polymerase II, polypeptide A) small phosphatase like 2	2	121,782,189	121,839,322	-173	upstream	4.00
NM_026251	4930424G05Rik	RIKEN cDNA 4930424G05 gene	2	121,945,844	122,011,925	34741	in gene	5.00

Accession No.	Symbol	Gene Title	Chr	Gene Start	Gene End	Interval Dists to Start	Interval Pos	Avg Peak
NM_009735	B2m	beta-2 microglobulin	2	121,973,423	121,978,818	3761	in gene	5.00
NM_080510	Trim69	tripartite motif-containing 69	2	121,986,436	122,004,763	-9252	upstream	5.00
NM_177610	Duox2	dual oxidase 2	2	122,106,173	122,123,901	-1635	upstream	5.00
NM_025777	Duoxa2	dual oxidase maturation factor 2	2	122,124,636	122,128,621	900	in gene	5.00
NM_145395	Duoxa1	dual oxidase maturation factor 1	2	122,129,285	122,139,466	13930	downstream	5.00
NM_177608	3110001I20Rik	RIKEN cDNA 3110001I20 gene	2	125,562,722	125,608,606	174	in gene	8.00
NM_009939	Cops2	COP9 (constitutive photomorphogenic) homolog, subunit 2 (Arabidopsis thaliana)	2	125,656,040	125,684,754	146	in gene	7.00
NM_175154	Galk2	galactokinase 2	2	125,684,954	125,810,034	-346	upstream	7.00
NM_001080944	Atp8b4	ATPase, class I, type 8B, member 4	2	126,148,481	126,200,339	2227	in gene	6.00
NM_010249, NM_207669	Gabpb1	GA repeat binding protein, beta 1	2	126,454,649	126,501,223	743	in gene	7.00
NM_001081089	A530057A03Rik	RIKEN cDNA A530057A03 gene	2	127,250,935	127,261,828	3017	in gene	6.00
NM_029629	Fahd2a	fumarylacetoacetate hydrolase domain containing 2A	2	127,261,951	127,270,241	16289	downstream	6.00
NM_019789	Kcnip3	Kv channel interacting protein 3, calsenilin	2	127,282,234	127,347,106	25842, 18194	in gene, in gene	6.50
NM_016902	Nphp1	nephronophthisis 1 (juvenile) homolog (human)	2	127,566,468	127,614,590	-3474	upstream	5.00
XR_035441, XR_035448, XR_035457	1500011K16Rik	RIKEN cDNA 1500011K16 gene	2	127,618,033	127,618,203	139	in gene	5.00
NM_009772	Bub1	budding uninhibited by benzimidazoles 1 homolog (S. cerevisiae)	2	127,626,857	127,657,531	39467	downstream	5.00
NM_028248	Tmem87b	transmembrane protein 87B	2	128,644,039	128,679,997	-55	upstream	8.00
NM_181589	Ckap2l	cytoskeleton associated protein 2-like	2	129,094,617	129,122,900	100	in gene	7.00
NM_010554	Il1a	interleukin 1 alpha	2	129,125,346	129,135,708	12908	downstream	7.00
NM_029432	4930402H24Rik	RIKEN cDNA 4930402H24 gene	2	130,533,567	130,665,846	22	in gene	7.00
XM_001480201, XM_001480601	C030014O09Rik	RIKEN cDNA C030014O09 gene	2	130,665,924	130,666,704	-100	upstream	7.00
NM_011045	Pcna	proliferating cell nuclear antigen	2	132,075,022	132,078,916	-9788	upstream	6.00
NM_138651	Cds2	CDP-diacylglycerol synthase (phosphatidate cytidylyltransferase) 2	2	132,088,993	132,137,777	-289, 1919	upstream, in gene	6.50
NM_144944	Prokr2	prokineticin receptor 2	2	132,196,901	132,211,136	-48	upstream	8.00
NM_007694	Chgb	chromogranin B	2	132,607,014	132,620,808	4463	in gene	6.00
NM_001038641, NM_028201, NM_028834	2210009G21Rik	RIKEN cDNA 2210009G21 gene	2	136,716,954	136,895,514	136646	in gene	5.00
NM_009751	Bfsp1	beaded filament structural protein in lens-CP94	2	143,652,264	143,688,909	33837	in gene	6.00
NM_019771	Dstn	destrin	2	143,741,347	143,769,060	-259	upstream	7.00
XR_035472, XR_035475	EG433481	predicted gene, EG433481	2	143,999,970	144,015,026	-1918	upstream	6.00
NM_025314	Dtd1	D-tyrosyl-tRNA deacylase 1 homolog (S. cerevisiae)	2	144,420,879	144,594,457	39329	in gene	6.00
XM_001481068	LOC100043810	hypothetical protein LOC100043810	2	145,526,931	145,579,408	45245	in gene	5.00
NM_028724	Rin2	Ras and Rab interactor 2	2	145,611,883	145,713,349	373, 60821	in gene, in gene	5.00
NM_001077632, NM_010919	Nkx2-2	NK2 transcription factor related, locus 2 (Drosophila)	2	147,003,282	147,012,138	629	in gene	5.00
XM_001481082	LOC100043816	hypothetical protein LOC100043816	2	147,012,118	147,019,978	8469, -5974	downstream, upstream	5.50
NM_009976	Cst3	cystatin C	2	148,697,468	148,701,204	4756	downstream	5.00
NM_153781	Pygb	brain glycogen phosphorylase	2	150,612,532	150,657,484	-84, 30764	upstream, in gene	5.50
XM_619828, XM_904795	Scrt2	scratch homolog 2, zinc finger protein (Drosophila)	2	151,855,292	151,921,538	-44	upstream	6.00
NM_029688	Srxn1	sulfiredoxin 1 homolog (S. cerevisiae)	2	151,931,466	151,937,089	14966	downstream	5.00
NM_001037247	Defb36	defensin beta 36	2	152,430,109	152,438,464	15945	downstream	4.00
NM_001039122	OTTMUSG00000015862	predicted gene, OTTMUSG00000015862	2	152,448,092	152,448,789	2735	downstream	4.00
NM_009047	Rem1	rad and gem related GTP binding protein 1	2	152,452,744	152,460,927	-6690	upstream	4.00
XM_001004869, XM_001473601	2500004C02Rik	RIKEN cDNA 2500004C02 gene	2	153,168,231	153,171,809	473	in gene	5.00
NM_001039939	Asxl1	additional sex combs like 1 (Drosophila)	2	153,171,875	153,229,743	-539	upstream	5.00
NM_025876	Cdk5rap1	CDK5 regulatory subunit associated protein 1	2	154,161,122	154,198,455	-5385	upstream	7.00
NM_009228	Snta1	syntrophin, acidic 1	2	154,202,064	154,233,817	29977	in gene	7.00
NM_023130	Raly	hnRNP-associated with lethal yellow	2	154,616,893	154,692,980	-333	upstream	7.00
NM_015770	a	nonagouti	2	154,839,306	154,876,748	19670	in gene	6.00
NM_144786	Ggtl3	gamma-glutamyltransferase-like 3	2	155,316,121	155,340,563	-3293	upstream	6.00
NM_019811	Acss2	acyl-CoA synthetase short-chain family member 2	2	155,343,779	155,388,479	77	in gene	6.00
XM_001481279, XM_001481334	Myh7b	myosin, heavy chain 7B, cardiac muscle, beta	2	155,415,661	155,460,043	52003	downstream	8.00
NM_018888	2410003P15Rik	RIKEN cDNA 2410003P15 gene	2	155,672,643	155,755,784	14376	in gene	6.00
NM_025516	Ergic3	ERGIC and golgi 3	2	155,833,861	155,844,015	12627	downstream	4.00
XM_001481280, XM_001481335	Fer1l4	fer-1-like 4 (C. elegans)	2	155,845,578	155,878,376	31888	in gene	4.00
NM_172674	Phf20	PHD finger protein 20	2	156,022,383	156,135,689	107905	in gene	5.00
NM_020255	Scand1	SCAN domain-containing 1	2	156,137,594	156,138,262	7974	downstream	5.00
NM_001042487, NM_001042488, NM_146128	Dlgap4	discs, large homolog-associated protein 4 (Drosophila)	2	156,490,531	156,590,099	22765, 56029	in gene, in gene	6.00
NM_019642	Rpn2	ribophorin II	2	157,104,834	157,152,054	23774	in gene	7.00

Accession No.	Symbol	Gene Title	Chr	Gene Start	Gene End	Interval Dists to Start	Interval Pos	Avg Peak
NM_001025395, NM_009271	Src	Rous sarcoma oncogene	2	157,250,029	157,297,574	-3789	upstream	5.00
NM_175692	A930034L06Rik	RIKEN cDNA A930034L06 gene	2	158,201,374	158,211,881	4130	in gene	7.00
NM_175419	Actr5	ARP5 actin-related protein 5 homolog (yeast)	2	158,450,649	158,464,947	23559	downstream	6.00
NM_177263	Zhx3	zinc fingers and homeoboxes 3	2	160,596,183	160,698,726	-9482	upstream	4.00
NM_022883	Lpin3	lipin 3	2	160,706,469	160,731,528	1739	in gene	4.00
NM_021464	Ptprt	protein tyrosine phosphatase, receptor type, T	2	161,353,359	162,486,883	1090163	in gene	4.00
NM_013731	Sgk2	serum/glucocorticoid regulated kinase 2	2	162,813,282	162,839,863	29518	downstream	8.00
NM_172150	Ift52	intraflagellar transport 52 homolog (Chlamydomonas)	2	162,843,208	162,871,871	-408, 31384	upstream, downstream	6.50
NM_008652	Mybl2	myeloblastosis oncogene-like 2	2	162,880,371	162,910,423	-5779	upstream	5.00
NM_021566	Jph2	junctophilin 2	2	163,163,509	163,223,686	-1530	upstream	5.00
NM_025699	3230401D17Rik	RIKEN cDNA 3230401D17 gene	2	163,231,558	163,245,206	19990	downstream	5.00
NM_013592	Matn4	matrilin 4	2	164,215,134	164,230,660	2132	in gene	5.00
NM_009036	Rbpjl	recombination signal binding protein for immunoglobulin kappa J region-like	2	164,228,694	164,240,948	-166	upstream	5.00
NM_011521	Sdc4	syndecan 4	2	164,249,747	164,268,688	15893, 304	in gene, in gene	5.00
NM_001048227, NM_001048228, NM_001048229, NM_026797	Dbndd2	dysbindin (dystrobrevin binding protein 1) domain containing 2	2	164,311,640	164,318,823	11368	downstream	6.00
NM_133779	Pigt	phosphatidylinositol glycan anchor biosynthesis, class T	2	164,323,025	164,333,801	-17	upstream	6.00
NM_020333	Slc12a5	solute carrier family 12, member 5	2	164,793,819	164,825,231	29829	in gene	4.00
NM_174988	Cdh22	cadherin 22	2	164,937,009	165,060,219	2011	in gene	6.00
NM_029021	4833422F24Rik	RIKEN cDNA 4833422F24 gene	2	165,220,950	165,225,894	-3802	upstream	5.00
NM_054055	Slc13a3	solute carrier family 13 (sodium-dependent dicarboxylate transporter), member 3	2	165,230,795	165,298,697	69001	downstream	5.00
NM_010165	Eya2	eyes absent 2 homolog (Drosophila)	2	165,480,798	165,597,131	-414	upstream	7.00
NM_028072	Sulf2	sulfatase 2	2	165,898,589	165,981,156	24900	in gene	5.00
XM_130646, XM_911012	Arfgef2	ADP-ribosylation factor guanine nucleotide-exchange factor 2 (brefeldin A-inhibited)	2	166,631,064	166,723,553	16152	in gene	4.00
NM_030743	Zfp313	zinc finger protein 313	2	167,318,145	167,341,666	10783	in gene	7.00
NM_009883	Cebpb	CCAAT/enhancer binding protein (C/EBP), beta	2	167,514,415	167,515,918	593	in gene	6.00
NM_009628	Adnp	activity-dependent neuroprotective protein	2	168,006,465	168,032,562	1618	in gene	5.00
NM_010072	Dpm1	dolichol-phosphate (beta-D) mannosyltransferase 1	2	168,034,548	168,055,879	24935	downstream	5.00
NM_001037177, NM_001037178, NM_010899	Nfatc2	nuclear factor of activated T-cells, cytoplasmic, calcineurin-dependent 2	2	168,301,910	168,427,155	-269	upstream	5.00
NM_015731	Atp9a	ATPase, class II, type 9A	2	168,459,938	168,567,300	49236, 13220	in gene, in gene	5.50
NM_029815	Bcas1	breast carcinoma amplified sequence 1	2	170,172,648	170,253,328	26224	in gene	4.00
NM_008561	Mc3r	melanocortin 3 receptor	2	172,073,992	172,076,614	8600	downstream	4.00
NM_025542	2410001C21Rik	RIKEN cDNA 2410001C21 gene	2	172,266,078	172,295,399	35858	downstream	6.00
XM_130728, XM_913316	1700029J11Rik	RIKEN cDNA 1700029J11 gene	2	172,298,028	172,299,831	3908	downstream	6.00
XM_130735, XM_913826	1700030G11Rik	RIKEN cDNA 1700030G11 gene	2	173,394,165	173,403,842	-8462	upstream	6.00
XM_001481221	LOC100043911	hypothetical protein LOC100043911	2	173,422,634	173,434,319	-4929	upstream	7.00
NM_172675	Stx16	syntaxin 16	2	173,901,822	173,923,908	-158	upstream	9.00
NM_001080971	Tubb1	tubulin, beta 1	2	174,276,196	174,283,383	13468	downstream	6.00
NM_025983	Atp5e	ATP synthase, H+ transporting, mitochondrial F1 complex, epsilon subunit	2	174,286,576	174,289,602	-62	upstream	6.00
NM_025531	Slmo2	slowmo homolog 2 (Drosophila)	2	174,290,592	174,298,442	8778	downstream	6.00
NM_001081092	Taf4a	TAF4A RNA polymerase II, TATA box binding protein (TBP)-associated factor	2	179,646,851	179,711,351	40951, 16583	in gene, in gene	6.00
NM_025587	Rps21	ribosomal protein S21	2	179,992,084	179,993,149	524	in gene	5.00
NM_145851	Cables2	Cdk5 and Abl enzyme substrate 2	2	179,993,244	180,008,170	15562	downstream	5.00
NM_145760	Arfgap1	ADP-ribosylation factor GTPase activating protein 1	2	180,701,974	180,717,147	18058	downstream	6.00
XM_001473208, XM_914804	Col20a1	collagen, type XX, alpha 1	2	180,721,240	180,751,033	-1208, 10152	upstream, in gene	6.00
NM_009133	Stmn3	stathmin-like 3	2	181,041,164	181,049,205	277	in gene	7.00
NM_001001882	Rtel1	regulator of telomere elongation helicase 1	2	181,054,490	181,091,321	-5562	upstream	7.00
NM_001048148, NM_144894	Zgpat	zinc finger, CCCH-type with G patch domain	2	181,099,636	181,115,498	15996	downstream	6.00
NM_023684	Lime1	Lck interacting transmembrane adaptor 1	2	181,115,940	181,118,333	-308	upstream	6.00
NM_028125	Btbd4	BTB (POZ) domain containing 4	2	181,145,255	181,194,131	29976	in gene	4.00
NM_183181	BC050777	cDNA sequence BC050777	2	181,227,915	181,229,682	-9355	upstream	6.00
XM_001476491	LOC100041389	hypothetical protein LOC100041389	3	9,053,762	9,056,698	-7762, -2287, 7710	upstream, upstream, downstream	4.67
NM_133218	Zfp704	zinc finger protein 704	3	9,427,023	9,610,085	85429	in gene	6.00
XM_910834, XM_975226	EG635702	predicted gene, EG635702	3	23,703,838	24,682,465	52145	in gene	5.00
NM_173182	Fndc3b	fibronectin type III domain containing 3B	3	27,315,083	27,609,299	172179	in gene	5.00
XM_001473602, XM_001473621, XM_001474897, XM_001474909	Tnik	TRAF2 and NCK interacting kinase	3	28,162,136	28,574,776	101192	in gene	5.00

Accession No.	Symbol	Gene Title	Chr	Gene Start	Gene End	Interval Dists to Start	Interval Pos	Avg Peak
NM_001039090, NM_011386	Skil	SKI-like	3	30,993,983	31,021,499	33	in gene	9.00
XM_001475742	LOC100041072	hypothetical protein LOC100041072	3	33,916,628	33,918,801	-271	upstream	8.00
NM_008053	Fxr1h	fragile X mental retardation gene 1, autosomal homolog	3	33,919,030	33,968,260	42	in gene	8.00
XM_896343	EG621304	predicted gene, EG621304	3	50,336,548	50,432,305	7505	in gene	6.00
NM_023502	Elf2	E74-like factor 2	3	51,059,587	51,129,909	61429	in gene	7.00
NM_025523	Ndufc1	NADH dehydrogenase (ubiquinone) 1, subcomplex unknown, 1	3	51,209,401	51,212,877	349	in gene	8.00
NM_053089	Narg1	NMDA receptor-regulated gene 1	3	51,220,051	51,277,953	-7523	upstream	8.00
NM_080793	Setd7	SET domain containing (lysine methyltransferase) 7	3	51,319,240	51,364,745	649	in gene	5.00
XM_001473103	LOC100039684	hypothetical protein LOC100039684	3	51,368,088	51,371,039	-3992	upstream	5.00
NM_174995	Mgst2	microsomal glutathione S-transferase 2	3	51,465,115	51,486,597	869	in gene	6.00
XM_001473484, XM_001478255	LOC433587	similar to mastermind-like 3	3	51,908,052	51,909,737	-10135	upstream	4.00
NM_172862	Frem2	Fras1 related extracellular matrix protein 2	3	53,317,860	53,461,277	57965	in gene	5.00
NM_019978	Dclk1	doublecortin-like kinase 1	3	55,046,550	55,340,600	153802	in gene	4.00
NM_019410	Pfn2	profilin 2	3	57,645,817	57,651,459	-429	upstream	6.00
NM_001081229	Tsc22d2	TSC22 domain family 2	3	58,219,611	58,270,709	1477	in gene	5.00
NM_032399	Gpr87	G protein-coupled receptor 87	3	58,982,845	58,984,007	9863	downstream	7.00
NM_028808	P2ry13	purinergic receptor P2Y, G-protein coupled 13	3	59,011,828	59,014,804	11828	downstream	7.00
NM_015728	Slc33a1	solute carrier family 33 (acetyl-CoA transporter), member 1	3	63,746,258	63,768,655	1151	in gene	6.00
NM_178892	Tiparp	TCDD-inducible poly(ADP-ribose) polymerase	3	65,332,369	65,359,440	19759	in gene	7.00
NM_008351	Il12a	interleukin 12a	3	68,494,566	68,502,447	-3910	upstream	6.00
NM_178726	Ppm1l	protein phosphatase 1 (formerly 2C)-like	3	69,120,840	69,359,326	22792	in gene	6.00
NM_020026	B3galnt1	UDP-GalNAc:betaGlcNAc beta 1,3-galactosaminyltransferase, polypeptide 1	3	69,378,108	69,402,783	271	in gene	6.00
NM_009250	Serpini1	serine (or cysteine) peptidase inhibitor, clade I, member 1	3	75,361,495	75,446,276	73049	in gene	7.00
NM_181849	Fgb	fibrinogen, B beta polypeptide	3	82,846,225	82,853,712	-5563	upstream	6.00
NM_016784	Plrg1	pleiotropic regulator 1, PRL1 homolog (Arabidopsis)	3	82,859,460	82,876,213	-185	upstream	6.00
NM_144896	Pet112l	PET112-like (yeast)	3	85,378,051	85,458,392	27437	in gene	7.00
NM_001032413, NM_001032414, NM_028460, NM_152799	3110045G13Rik	RIKEN cDNA 3110045G13 gene	3	87,553,019	87,572,875	-1173, -7301	upstream, upstream	4.50
XM_283871, XM_899803, XM_908571, XM_920908	Ntrk1	neurotrophic tyrosine kinase, receptor, type 1	3	87,582,166	87,599,084	25036, 18908, 15372, 11772	downstream, downstream, in gene, in gene	5.25
NM_011832	Insrr	insulin receptor-related receptor	3	87,600,984	87,620,023	9736	in gene	7.00
NM_001025571, NM_021309	Sh2d2a	SH2 domain protein 2A	3	87,650,677	87,659,644	12427, 17483	downstream, downstream	7.50
NM_033573	Prcc	papillary renal cell carcinoma (translocation-associated)	3	87,662,836	87,689,502	26398, 21342	in gene, in gene	7.50
NM_008231	Hdgf	hepatoma-derived growth factor	3	87,710,288	87,720,048	352, 15664	in gene, downstream	5.50
NM_026591	Mrpl24	mitochondrial ribosomal protein L24	3	87,723,466	87,727,586	2486	in gene	5.00
NM_153562	BC023814	cDNA sequence BC023814	3	87,726,827	87,734,068	8116	downstream	5.00
NM_177663	Isg20l2	interferon stimulated exonuclease gene 20-like 2	3	87,734,236	87,744,608	-8284	upstream	5.00
NM_133665	Mef2d	myocyte enhancer factor 2D	3	87,946,317	87,973,089	2291	in gene	8.00
NM_178246	Smg5	Smg-5 homolog, nonsense mediated mRNA decay factor (C. elegans)	3	88,140,182	88,166,259	23807	in gene	6.00
NM_198410	Paqr6	progestin and adipoQ receptor family member VI	3	88,168,511	88,172,377	-4522	upstream	6.00
NM_031368	Bglap-rs1	bone gamma-carboxyglutamate protein, related sequence 1	3	88,172,542	88,173,634	9645	downstream	6.00
NM_031248	Mapbpip	mitogen activated protein binding protein interacting protein	3	88,353,741	88,356,849	8193	downstream	5.00
NM_008487	Arhgef2	rho/rac guanine nucleotide exchange factor (GEF) 2	3	88,425,060	88,450,836	2604	in gene	6.00
NM_022994	Dap3	death associated protein 3	3	88,727,363	88,754,204	8156	in gene	8.00
NM_001077411, NM_008094	Gba	glucosidase, beta, acid	3	89,006,850	89,012,603	1822	in gene	6.00
NM_013604	Mtx1	metaxin 1	3	89,013,011	89,017,827	9155	downstream	6.00
NM_001039466, NM_183037	Trim46	tripartite motif protein 46	3	89,038,099	89,049,819	2155	in gene	4.00
NM_025327	Krtcap2	keratinocyte associated protein 2	3	89,050,360	89,053,644	-2696	upstream	4.00
NM_027315	Ube2q1	ubiquitin-conjugating enzyme E2Q (putative) 1	3	89,577,538	89,587,917	9806	in gene	7.00
NM_010559	Il6ra	interleukin 6 receptor, alpha	3	89,673,246	89,717,084	3356, 1788	in gene, in gene	6.00
NM_026374	Ilf2	interleukin enhancer binding factor 2	3	90,280,123	90,292,301	14725	downstream	5.00
NM_133854	Snapap	SNAP-associated protein	3	90,291,956	90,294,926	78	in gene	5.00
NM_023215	2500003M10Rik	RIKEN cDNA 2500003M10 gene	3	90,302,880	90,313,365	18517	downstream	5.00
NM_026416	S100a16	S100 calcium binding protein A16	3	90,345,145	90,347,073	1239	in gene	6.00
NM_001082484, NM_029721	Snx27	sorting nexin family member 27	3	94,301,466	94,386,624	1088	in gene	5.00

Accession No.	Symbol	Gene Title	Chr	Gene Start	Gene End	Interval Dists to Start	Interval Pos	Avg Peak
NM_175356	Pik4cb	phosphatidylinositol 4-kinase, catalytic, beta polypeptide	3	94,778,658	94,810,511	30462, 33966	in gene, downstream	5.00
NM_030074	Zfp687	zinc finger protein 687	3	94,810,624	94,819,160	10040, 6536	downstream, in gene	5.00
NM_011351	Sema6c	sema domain, transmembrane domain (TM), and cytoplasmic domain, (semaphorin) 6C	3	94,968,296	94,977,238	10040	downstream	5.00
NM_029885, NM_172512	Gabpb2	GA repeat binding protein, beta 2	3	94,985,688	95,021,864	43528	downstream	5.00
NM_173347	Prune	prune homolog (Drosophila)	3	95,057,596	95,085,998	494, -882, -1330	in gene, upstream, upstream	6.67
NM_133858	4930504E06Rik	RIKEN cDNA 4930504E06 gene	3	95,086,866	95,100,083	-1362, 14, 462	upstream, in gene, in gene	6.67
NM_146133	Golph3l	golgi phosphoprotein 3-like	3	95,392,881	95,423,164	36239	downstream	6.00
XM_001001572, XM_906073	EG668457	predicted gene, EG668457	3	95,426,151	95,426,769	2969	downstream	6.00
NM_001026212, NM_019561	Ensa	endosulfine alpha	3	95,428,902	95,436,039	218	in gene	6.00
NM_144899	Adamtsl4	ADAMTS-like 4	3	95,480,127	95,491,781	12325, 10901	downstream, in gene	6.00
NM_153513	BC028528	cDNA sequence BC028528	3	95,687,889	95,695,871	-1889	upstream	7.00
NM_146104	Aph1a	anterior pharynx defective 1a homolog (C. elegans)	3	95,697,919	95,702,226	-159	upstream	7.00
NM_011797	Car14	carbonic anhydrase 14	3	95,701,723	95,708,562	10802	downstream	7.00
NM_027241	Polr3gl	polymerase (RNA) III (DNA directed) polypeptide G like	3	96,381,797	96,398,066	-6926	upstream	8.00
NM_001024851	Ankrd34	ankyrin repeat domain 34	3	96,400,559	96,403,701	4433	downstream	8.00
NM_018812, NM_146135	Pias3	protein inhibitor of activated STAT 3	3	96,500,392	96,509,885	10488	downstream	8.00
NM_019800	Acp6	acid phosphatase 6, lysophosphatidic	3	96,962,714	96,980,491	294	in gene	5.00
NM_008256	Hmgcs2	3-hydroxy-3-methylglutaryl-Coenzyme A synthase 2	3	98,084,358	98,114,655	2682	in gene	7.00
NM_207205	Igsf3	immunoglobulin superfamily, member 3	3	101,182,236	101,264,792	30724	in gene	4.00
NM_172295	BC037703	cDNA sequence BC037703	3	101,616,999	101,639,884	29596	downstream	6.00
XM_001002464, XM_001474615	Slc22a15	solute carrier family 22 (organic anion/cation transporter), member 15	3	101,663,850	101,765,317	37125	in gene	6.00
XM_001479402	LOC100043047	similar to CG2839-PA	3	101,827,526	101,857,240	34138	downstream	6.00
XM_001480691	LOC100043538	hypothetical protein LOC100043538	3	103,526,145	103,538,152	-3832	upstream	8.00
NM_133859	Olfml3	olfactomedin-like 3	3	103,539,317	103,541,924	-60	upstream	8.00
NM_010432	Hipk1	homeodomain interacting protein kinase 1	3	103,547,594	103,595,102	53118, -309	downstream, upstream	7.00
XM_001478142, XM_001480843	A630076J17Rik	RIKEN cDNA A630076J17 gene	3	107,033,532	107,037,641	2100	in gene	7.00
NM_001044382	Prok1	prokineticin 1	3	107,038,449	107,042,625	6993	downstream	7.00
NM_172271	Slc6a17	solute carrier family 6 (neurotransmitter transporter), member 17	3	107,270,466	107,320,936	45720, 1544	in gene, in gene	6.00
NM_026261	Ubl4b	ubiquitin-like 4B	3	107,356,616	107,357,991	-5113	upstream	5.00
NM_007778	Csf1	colony stimulating factor 1 (macrophage)	3	107,543,978	107,563,387	18299	in gene	8.00
NM_181400	Wdr47	WD repeat domain 47	3	108,394,335	108,448,637	61249	downstream	6.00
NM_145543	Clcc1	chloride channel CLIC-like 1	3	108,457,165	108,480,930	-1581	upstream	6.00
NM_030699	Ntng1	netrin G1	3	109,584,024	109,946,146	264130	in gene	5.00
NM_001080818	Cdc14a	CDC14 cell division cycle 14 homolog A (S. cerevisiae)	3	115,975,471	116,126,950	131302	in gene	5.00
NM_023245	Palmd	palmdelphin	3	116,621,180	116,671,870	54238	downstream	6.00
NM_029655	Snx7	sorting nexin 7	3	117,484,796	117,571,712	-32	upstream	6.00
NM_145394	Slc44a3	solute carrier family 44, member 3	3	121,162,446	121,235,256	-8	upstream	6.00
XR_035399, XR_035470	4930432M17Rik	RIKEN cDNA 4930432M17 gene	3	121,373,681	121,385,900	-7473	upstream	6.00
NM_008991	Abcd3	ATP-binding cassette, sub-family D (ALD), member 3	3	121,461,828	121,518,133	-123	upstream	8.00
NM_013867	Bcar3	breast cancer anti-estrogen resistance 3	3	122,122,745	122,233,101	42791	in gene	5.00
XM_001480111	LOC100043415	similar to cyclic nucleotide gated channel beta 1	3	129,085,637	129,094,127	-7537	upstream	5.00
XR_034265, XR_034544	LOC435755	POU domain, class 5, transcription factor 1-like	3	129,129,764	129,137,633	7015	in gene	4.00
NM_130450	Elovl6	ELOVL family member 6, elongation of long chain fatty acids (yeast)	3	129,235,304	129,341,413	696, 84824	in gene, in gene	6.00
NM_008212	Hadh	hydroxyacyl-Coenzyme A dehydrogenase	3	130,936,342	130,974,955	16395	in gene	7.00
NM_028943	Sgms2	sphingomyelin synthase 2	3	131,025,656	131,047,853	24157	downstream	5.00
NM_146141	Ppa2	pyrophosphatase (inorganic) 2	3	132,973,080	133,041,033	24	in gene	5.00
NM_001040400	E130014J05Rik	RIKEN cDNA E130014J05 gene	3	133,126,803	133,207,332	1124	in gene	5.00
XM_884831, XM_915250	EG620439	predicted gene, EG620439	3	134,871,948	134,872,142	3476	downstream	7.00
NM_173762	Cenpe	centromere protein E	3	134,875,527	134,936,504	-103	upstream	7.00
NM_025356	Ube2d3	ubiquitin-conjugating enzyme E2D 3 (UBC4/5 homolog, yeast)	3	135,101,723	135,130,142	53	in gene	4.00
NM_008913	Ppp3ca	protein phosphatase 3, catalytic subunit, alpha isoform	3	136,333,734	136,598,743	22522, 120826	in gene, in gene	5.00
NM_177665	EG229862	predicted gene, EG229862	3	137,213,009	137,215,586	4914	downstream	15.00
NM_019571	Tspan5	tetraspanin 5	3	138,405,159	138,567,388	91193	in gene	6.00
XM_001473646, XM_001479910	LOC620771	similar to ribosomal protein L21	3	138,482,900	138,492,163	-4189	upstream	6.00

Accession No.	Symbol	Gene Title	Chr	Gene Start	Gene End	Interval Dists to Start	Interval Pos	Avg Peak
NM_001040690, NM_145544	Rap1gds1	RAP1, GTP-GDP dissociation stimulator 1	3	138,588,870	138,738,163	-317	upstream	6.00
NM_198659	B930007M17Rik	RIKEN cDNA B930007M17 gene	3	138,868,857	139,373,263	107900	in gene	5.00
NM_153564	Gbp5	guanylate nucleotide binding protein 5	3	142,159,898	142,185,308	33366	downstream	6.00
NM_001083312, NM_145545	Gbp6	guanylate binding protein 6	3	142,193,302	142,213,047	-38	upstream	6.00
NM_010260	Gbp2	guanylate nucleotide binding protein 2	3	142,283,627	142,300,972	16997	in gene	5.00
NM_019464	Sh3glb1	SH3-domain GRB2-like B1 (endophilin)	3	144,351,808	144,383,287	647	in gene	5.00
NM_010516	Cyr61	cysteine rich protein 61	3	145,309,940	145,312,945	2241	in gene	6.00
NM_027617	Spata1	spermatogenesis associated 1	3	146,120,167	146,162,717	205	in gene	8.00
NM_010318	Gng5	guanine nucleotide binding protein (G protein), gamma 5 subunit	3	146,162,800	146,168,507	-288	upstream	8.00
NM_027332, NM_027371	Bxdc5	brix domain containing 5	3	146,169,382	146,184,382	21870	downstream	8.00
XM_001474558	LOC100040368	similar to CG33791-PB	3	148,649,507	148,666,995	1389, 2973	in gene, in gene	6.00
NM_008966	Ptgfr	prostaglandin F receptor	3	151,461,574	151,500,492	-7444	upstream	5.00
NM_025926, NM_027287	Dnajb4	DnaJ (Hsp40) homolog, subfamily B, member 4	3	151,847,060	151,873,047	-201	upstream	7.00
NM_057172	Fubp1	far upstream element (FUSE) binding protein 1	3	151,873,422	151,899,794	-174	upstream	7.00
NM_012028	St6galnac5	ST6 (alpha-N-acetyl-neuraminyl-2,3-beta-galactosyl-1,3)-N-acetylgalactosaminide alpha-2,6-sialyltransferase 5	3	152,482,850	152,645,174	169350	downstream	7.00
NM_007382	Acadm	acyl-Coenzyme A dehydrogenase, medium chain	3	153,585,323	153,607,396	-460	upstream	6.00
NM_017381	Zranb2	zinc finger, RAN-binding domain containing 2	3	157,197,361	157,211,303	-257	upstream	7.00
	Mirn186	microRNA 186	3	157,207,242	157,207,312	-10138	upstream	7.00
NM_001081358	Lrrc7	leucine rich repeat containing 7	3	157,747,403	158,225,185	400321	in gene	5.00
NM_001081183	1110037F02Rik	RIKEN cDNA 1110037F02 gene	4	11,413,105	11,478,290	18927	in gene	5.00
NM_001033453	Ppm2c	protein phosphatase 2C, magnesium dependent, catalytic subunit	4	11,886,958	11,893,567	-33	upstream	5.00
NM_009822	Runx1t1	runt-related transcription factor 1; translocated to, 1 (cyclin D-related)	4	13,711,929	13,818,264	48295	in gene	6.00
NM_172988	Fbxl4	F-box and leucine-rich repeat protein 4	4	22,284,712	22,361,193	19544	in gene	5.00
NM_030733	Gpr63	G protein-coupled receptor 63	4	24,900,566	24,936,380	23509	in gene	6.00
NM_001012450, NM_001012451, NM_080471	Ankrd6	ankyrin repeat domain 6	4	32,891,010	33,037,801	122745, 86169	in gene, in gene	6.00
NM_001033225	Pnrc1	proline-rich nuclear receptor coactivator 1	4	33,332,398	33,335,762	290	in gene	8.00
NM_011884	Rngtt	RNA guanylyltransferase and 5'-phosphatase	4	33,397,286	33,589,585	72954	in gene	7.00
NM_027041	1700003M02Rik	RIKEN cDNA 1700003M02 gene	4	34,658,581	34,677,427	-3581	upstream	5.00
NM_001033255	Gm136	gene model 136, (NCBI)	4	34,691,037	34,703,508	22500	downstream	5.00
NM_178061	Mobkl2b	MOB1, Mps One Binder kinase activator-like 2B (yeast)	4	34,899,953	35,104,715	88491	in gene	9.00
NM_007386	Aco1	aconitase 1	4	40,090,298	40,146,042	102	in gene	6.00
NM_172689	Ddx58	DEAD (Asp-Glu-Ala-Asp) box polypeptide 58	4	40,152,823	40,186,734	-2514	upstream	6.00
NM_001025444, NM_025545	Aptx	aprataxin	4	40,629,111	40,650,220	-196	upstream	8.00
NM_024241	Kif24	kinesin family member 24	4	41,337,781	41,411,873	-5034	upstream	5.00
NM_025539	Nudt2	nudix (nucleoside diphosphate linked moiety X)-type motif 2	4	41,412,181	41,427,959	4726	in gene	5.00
NM_173788	Npr2	natriuretic peptide receptor 2	4	43,644,807	43,664,116	11129	in gene	4.00
NM_001007463	Spag8	sperm associated antigen 8	4	43,664,601	43,666,424	10488	downstream	4.00
NM_015828	Gne	glucosamine	4	44,049,698	44,097,038	11422	in gene	5.00
XM_001474487	LOC100040313	hypothetical protein LOC100040313	4	44,105,508	44,119,716	4268	in gene	6.00
NM_153167	Wdr32	WD repeat domain 32	4	45,354,973	45,392,594	-77	upstream	7.00
NM_028788	1300002K09Rik	RIKEN cDNA 1300002K09 gene	4	45,861,832	45,899,876	-8	upstream	6.00
NM_198664	Tbc1d2	TBC1 domain family, member 2	4	46,617,262	46,663,071	49775	downstream	5.00
NM_009928	Col15a1	procollagen, type XV	4	47,220,884	47,326,037	19980	in gene	7.00
NM_172304	Tex10	testis expressed gene 10	4	48,443,828	48,486,294	-218	upstream	5.00
NM_028137	5730528L13Rik	RIKEN cDNA 5730528L13 gene	4	48,553,371	48,574,791	-347	upstream	4.00
NM_182999	Rnf20	ring finger protein 20	4	49,644,993	49,669,758	-17	upstream	5.00
XM_001476161, XM_001476174, XM_980440	Epb4.1l4b	erythrocyte protein band 4.1-like 4b	4	57,008,606	57,156,520	132856	in gene	5.00
NM_011207	Ptpn3	protein tyrosine phosphatase, non-receptor type 3	4	57,252,396	57,313,215	59775, -1217	in gene, upstream	4.50
NM_172868	Palm2	paralemmin 2	4	57,581,154	57,730,017	121438	in gene	5.00
NM_001037127, NM_001037128, NM_001037129, NM_001037130, NM_010944	Musk	muscle, skeletal, receptor tyrosine kinase	4	58,298,834	58,387,175	78	in gene	6.00
XM_909547, XM_988632	LOC634731	similar to sushi domain containing 1	4	59,327,957	59,451,499	39467	in gene	5.00
NM_024255	Hsdl2	hydroxysteroid dehydrogenase like 2	4	59,594,463	59,631,566	44193	downstream	5.00
NM_001015681, NM_153158	E130308A19Rik	RIKEN cDNA E130308A19 gene	4	59,639,199	59,767,175	-543	upstream	5.00
NM_023597	Wdr31	WD repeat domain 31	4	62,114,768	62,131,906	15810	in gene	4.00
NM_008525	Alad	aminolevulinate, delta-, dehydratase	4	62,170,204	62,181,097	-87	upstream	6.00

Accession No.	Symbol	Gene Title	Chr	Gene Start	Gene End	Interval Dists to Start	Interval Pos	Avg Peak
NM_021498	Pole3	polymerase (DNA directed), epsilon 3 (p17 subunit)	4	62,184,832	62,186,048	4864	downstream	6.00
NM_177607	4933430I17Rik	RIKEN cDNA 4933430I17 gene	4	62,186,403	62,209,027	-5219	upstream	6.00
NM_001081650, NM_019492, NM_134257	Rgs3	regulator of G-protein signaling 3	4	62,220,881	62,363,369	71199, 83706	in gene, in gene	6.00
NM_025685	Col27a1	procollagen, type XXVII, alpha 1	4	62,876,446	62,996,025	32770	in gene	6.00
	Mirn455	microRNA 455	4	62,917,884	62,917,965	-8668	upstream	6.00
NM_008768	Orm1	orosomucoid 1	4	63,005,600	63,009,196	6304	downstream	6.00
NM_013623	Orm3	orosomucoid 3	4	63,017,196	63,020,545	-5292	upstream	6.00
NM_001045514	Akna	AT-hook transcription factor	4	63,028,159	63,064,388	-6636	upstream	5.00
NM_001008791, NM_001008792, NM_001008793, NM_001008794, NM_001008795, NM_001008796, NM_001008797, NM_001008798, NM_028640	Whrn	whirlin	4	63,075,944	63,156,985	85961, 34009	downstream, in gene	5.50
NM_172869	Frmd3	FERM domain containing 3	4	73,659,565	73,835,832	149811	in gene	6.00
NM_144787	Jmjd2c	jumonji domain containing 2C	4	73,897,852	74,051,765	-7	upstream	5.00
NM_027238	1810054D07Rik	RIKEN cDNA 1810054D07 gene	4	82,866,205	82,970,093	525	in gene	5.00
XM_001472567	LOC100039265	hypothetical protein LOC100039265	4	94,698,054	94,716,038	-3322	upstream	8.00
NM_010591	Jun	Jun oncogene	4	94,715,746	94,718,878	-482	upstream	8.00
NM_001033773	Ube2u	ubiquitin-conjugating enzyme E2U (putative)	4	100,151,472	100,222,750	48960	in gene	6.00
NM_183024	Raver2	ribonucleoprotein, PTB-binding 2	4	100,741,643	100,824,975	15701	in gene	6.00
NM_146145	Jak1	Janus kinase 1	4	100,824,579	100,937,887	77599, -33	in gene, upstream	5.50
NM_198412	Dnajc6	DnaJ (Hsp40) homolog, subfamily C, member 6	4	101,223,199	101,315,404	96945	downstream	8.00
NM_175036	Leprot	leptin receptor overlapping transcript	4	101,320,388	101,331,963	-244	upstream	8.00
NM_146254	Wdr78	WD repeat domain 78	4	102,710,674	102,786,904	-5080	upstream	6.00
NM_001039081, NM_027696	Mier1	mesoderm induction early response 1 homolog (Xenopus laevis	4	102,787,070	102,838,354	4914	in gene	6.00
NM_177732	Slc35d1	solute carrier family 35 (UDP-glucuronic acid/UDP-N-acetylgalactosamine dual transporter), member D1	4	102,844,323	102,887,489	11313	in gene	7.00
NM_178143	Prkaa2	protein kinase, AMP-activated, alpha 2 catalytic subunit	4	104,707,595	104,782,474	490	in gene	7.00
NM_080555	Ppap2b	phosphatidic acid phosphatase type 2B	4	104,829,952	104,905,372	71520	in gene	5.00
NM_025617	2210012G02Rik	RIKEN cDNA 2210012G02 gene	4	106,806,767	106,850,971	-133	upstream	8.00
NM_029565	Tmem59	transmembrane protein 59	4	106,851,297	106,873,592	-193	upstream	8.00
NM_147221	Glis1	GLIS family zinc finger 1	4	107,107,324	107,307,664	23204	in gene	6.00
NM_146255	Slc1a7	solute carrier family 1 (glutamate transporter), member 7	4	107,640,937	107,686,134	7879, 30690	in gene, in gene	5.00
NM_175472	Zcchc11	zinc finger, CCHC domain containing 11	4	108,132,031	108,232,020	14065	in gene	7.00
XM_001474862, XM_916360	LOC639774	similar to Butyrophilin-like protein 2 precursor (BTL-II)	4	111,591,997	111,612,262	18915	in gene	5.00
XM_981216	LOC666048	similar to C05G5.5	4	114,079,293	114,283,345	35379, 110467, 115891	in gene, in gene, in gene	6.33
NM_025647	Cmpk	cytidylate kinase	4	114,633,243	114,659,749	293, -155	in gene, upstream	6.50
NM_028142	Nsun4	NOL1/NOP2/Sun domain family, member 4	4	115,705,447	115,726,481	1905	in gene	5.00
XM_905120	LOC433748	similar to C49H3.3	4	115,872,863	115,873,610	8282	downstream	5.00
NM_181585	Pik3r3	phosphatidylinositol 3 kinase, regulatory subunit, polypeptide 3 (p55)	4	115,894,519	115,975,661	15550	in gene	4.00
NM_008958	Ptch2	patched homolog 2	4	116,768,961	116,787,436	8959	in gene	5.00
XR_035186, XR_035205, XR_035280	9530048O09Rik	RIKEN cDNA 9530048O09 gene	4	116,792,467	116,797,186	-5262	upstream	7.00
XM_001477060	LOC100041324	hypothetical protein LOC100041324	4	116,797,227	116,798,330	-4118	upstream	7.00
NM_175030	4833401D15Rik	RIKEN cDNA 4833401D15 gene	4	116,799,418	116,801,335	3030	downstream	7.00
NM_013807	Plk3	polo-like kinase 3 (Drosophila)	4	116,801,260	116,806,557	4109	in gene	7.00
NM_008135	Slc6a9	solute carrier family 6 (neurotransmitter transporter, glycine), member 9	4	117,507,863	117,541,910	3833	in gene	6.00
NM_146152	Ipo13	importin 13	4	117,567,098	117,587,604	1236	in gene	5.00
NM_011213	Ptprf	protein tyrosine phosphatase, receptor type, F	4	117,880,818	117,964,002	81058	in gene	6.00
NM_001004178	AA415398	expressed sequence AA415398	4	119,202,913	119,211,374	-530	upstream	6.00
NM_172699	Foxj3	forkhead box J3	4	119,212,293	119,301,724	-389, 29766, 97771	upstream, in gene, downstream	5.67
NM_008190	Guca2a	guanylate cyclase activator 2a (guanylin)	4	119,310,337	119,312,070	-273	upstream	5.00
NM_013883	Scmh1	sex comb on midleg homolog 1	4	120,119,636	120,202,560	81164	in gene	6.00
NM_177570	Slfnl1	schlafen like 1	4	120,204,836	120,209,266	-4036	upstream	6.00
NM_001048168, NM_008692	Nfyc	nuclear transcription factor-Y gamma	4	120,430,040	120,498,320	384	in gene	5.00
NM_025873	Trit1	tRNA isopentenyltransferase 1	4	122,693,840	122,732,177	-336	upstream	4.00
XM_112246, XM_907862	Nt5c1a	5'-nucleotidase, cytosolic IA	4	122,878,801	122,893,450	24719	downstream	12.00
XM_001472625	LOC100039298	similar to cyclic nucleotide gated channel beta 1	4	125,042,926	125,168,013	87954	in gene	6.00
NM_172701	1810007P19Rik	RIKEN cDNA 1810007P19 gene	4	125,735,809	125,766,578	38063	downstream	7.00

Accession No.	Symbol	Gene Title	Chr	Gene Start	Gene End	Interval Dists to Start	Interval Pos	Avg Peak
NM_138721	Lsm10	U7 snRNP-specific Sm-like protein LSM10	4	125,773,996	125,775,824	-124	upstream	7.00
NM_028800	Stk40	serine/threonine kinase 40	4	125,781,242	125,818,126	-7370, 43622	upstream, downstream	7.00
NM_172145	2610027C15Rik	RIKEN cDNA 2610027C15 gene	4	125,825,247	125,827,118	-383	upstream	7.00
NM_011970	Psmb2	proteasome (prosome, macropain) subunit, beta type 2	4	126,354,910	126,386,950	-1342	upstream	5.00
NM_011986	Ncdn	neurochondrin	4	126,420,999	126,430,603	-341	upstream	6.00
NM_001035525, NM_001035526, NM_133886	AU040320	expressed sequence AU040320	4	126,430,799	126,546,938	145	in gene	6.00
NM_026670	Zmym1	zinc finger, MYM domain containing 1	4	126,724,338	126,738,376	15944	downstream	5.00
NM_205823	Tlr12	toll-like receptor 12	4	128,292,891	128,295,733	8373, -139	downstream, upstream	5.50
NM_018774	Phc2	polyhomeotic-like 2 (Drosophila)	4	128,382,240	128,430,118	42160	in gene	5.00
NM_001009819	A3galt2	alpha 1,3-galactosyltransferase 2 (isoglobotriaosylceramide synthase)	4	128,436,502	128,446,542	18250	downstream	7.00
NM_001081098	BC039093	cDNA sequence BC039093	4	128,450,329	128,483,356	28604	in gene	7.00
NM_172875	Adc	arginine decarboxylase	4	128,609,390	128,639,661	317	in gene	4.00
NM_025452	Tmem54	transmembrane protein 54	4	128,782,860	128,788,829	15460	downstream	8.00
NM_010471	Hpca	hippocalcin	4	128,788,819	128,796,002	-2318	upstream	8.00
NM_029036	S100pbp	S100P binding protein	4	128,828,069	128,866,726	-474	upstream	7.00
NM_134151	Yars	tyrosyl-tRNA synthetase	4	128,867,195	128,896,851	5	in gene	7.00
NM_028603	2410081M15Rik	RIKEN cDNA 2410081M15 gene	4	129,030,876	129,055,272	872	in gene	7.00
NM_010693	Lck	lymphocyte protein tyrosine kinase	4	129,225,602	129,235,616	-10048	upstream	5.00
NM_182783	BC030183	cDNA sequence BC030183	4	129,254,112	129,255,791	10127	downstream	5.00
NM_173071	Bai2	brain-specific angiogenesis inhibitor 2	4	129,669,312	129,699,592	-32	upstream	5.00
NM_010174	Fabp3	fatty acid binding protein 3, muscle and heart	4	129,986,022	129,992,707	7663	downstream	6.00
NM_153160	Zcchc17	zinc finger, CCHC domain containing 17	4	129,993,145	130,037,192	43507	in gene	6.00
NM_030722	Pum1	pumilio 1 (Drosophila)	4	130,219,304	130,337,187	90360	in gene	7.00
NM_025297	Mecr	mitochondrial trans-2-enoyl-CoA reductase	4	131,399,386	131,423,682	-6154	upstream	6.00
NM_020273	Gmeb1	glucocorticoid modulatory element binding protein 1	4	131,780,934	131,807,767	32439	downstream	8.00
NM_025579	Taf12	TAF12 RNA polymerase II, TATA box binding protein (TBP)-associated factor	4	131,830,328	131,849,242	20888	downstream	5.00
NM_175306	Phactr4	phosphatase and actin regulator 4	4	131,911,893	131,978,322	18	in gene	6.00
NM_145553	BC008163	cDNA sequence BC008163	4	132,456,533	132,478,426	74	in gene	6.00
NM_153423	Wasf2	WAS protein family, member 2	4	132,686,548	132,754,245	69791	downstream	5.00
NM_008154	Gpr3	G-protein coupled receptor 3	4	132,765,255	132,768,422	12083	downstream	5.00
XM_001477989	LOC100042018	hypothetical protein LOC100042018	4	132,848,123	132,850,128	1168	in gene	5.00
NM_199306	Wdtc1	WD and tetratricopeptide repeats 1	4	132,848,381	132,895,020	45729	in gene	5.00
NM_172876	Gpatch3	G patch domain containing 3	4	133,130,660	133,140,157	-4	upstream	10.00
NM_133884	Atpbd1b	ATP binding domain 1 family, member B	4	133,140,295	133,147,649	-9639	upstream	10.00
NM_001080819	Arid1a	AT rich interactive domain 1A (Swi1 like)	4	133,234,923	133,309,526	-3434	upstream	8.00
NM_027995	Paqr7	progestin and adipoQ receptor family member VII	4	134,052,893	134,064,725	-368	upstream	5.00
NM_029100	Sepn1	selenoprotein N, 1	4	134,093,807	134,108,081	-7343	upstream	7.00
NM_207237	Man1c1	mannosidase, alpha, class 1C, member 1	4	134,117,605	134,260,205	144781, 34205	downstream, in gene	5.50
NM_019732	Runx3	runt related transcription factor 3	4	134,676,560	134,733,905	43675	in gene	6.00
NM_013885	Clic4	chloride intracellular channel 4 (mitochondrial)	4	134,769,884	134,828,675	28163	in gene	8.00
NM_001080387, NM_010178	Fusip1	FUS interacting protein (serine-arginine rich) 1	4	135,412,007	135,425,823	4121	in gene	5.00
NM_010142	Ephb2	Eph receptor B2	4	136,209,523	136,391,850	104698, 86650	in gene, in gene	5.00
NM_007939	Epha8	Eph receptor A8	4	136,485,334	136,512,733	7261, -483	in gene, upstream	6.50
NM_198248	Zbtb40	zinc finger and BTB domain containing 40	4	136,535,647	136,604,610	67010, 9170	in gene, in gene	5.00
NM_009523	Wnt4	wingless-related MMTV integration site 4	4	136,833,550	136,852,694	3570, 9056	in gene, in gene	4.50
NM_199307	Ece1	endothelin converting enzyme 1	4	137,418,212	137,521,144	51820, 68924	in gene, in gene	6.00
NM_026689	0610009K11Rik	RIKEN cDNA 0610009K11 gene	4	137,990,587	137,998,180	7029	in gene	4.00
NM_001037761, NM_009798	Capzb	capping protein (actin filament) muscle Z-line, beta	4	138,748,846	138,847,727	54194	in gene	6.00
NM_145384	Pqlc2	PQ loop repeat containing 2	4	138,854,445	138,866,593	-159	upstream	5.00
XM_001479428	LOC100042808	hypothetical protein LOC100042808	4	138,865,934	138,866,574	-178	upstream	5.00
NM_025337	Akr7a5	aldo-keto reductase family 7, member A5 (aflatoxin aldehyde reductase)	4	138,866,659	138,874,340	93	in gene	5.00
NM_175438	Aldh4a1	aldehyde dehydrogenase 4 family, member A1	4	139,178,925	139,205,602	4651	in gene	5.00
NM_011039	Pax7	paired box gene 7	4	139,293,996	139,388,883	76291, 8963	in gene, in gene	6.00
NM_198610	Igsf21	immunoglobin superfamily, member 21	4	139,582,767	139,802,699	195851	in gene	4.00
NM_153106	Padi6	peptidyl arginine deiminase, type VI	4	140,283,270	140,298,558	21918, -1890	downstream, upstream	5.00
NM_011061	Padi4	peptidyl arginine deiminase, type IV	4	140,301,780	140,330,100	29652	downstream	5.00
NM_029097	Atp13a2	ATPase type 13A2	4	140,542,843	140,563,245	19125	in gene	4.00
NM_008546	Mfap2	microfibrillar-associated protein 2	4	140,566,618	140,571,888	-4650	upstream	4.00
NM_025383	Necap2	NECAP endocytosis associated 2	4	140,622,432	140,634,249	25	in gene	6.00
NM_177867	Spata21	spermatogenesis associated 21	4	140,644,260	140,668,674	-10036	upstream	6.00
NM_010139	Epha2	Eph receptor A2	4	140,857,155	140,885,293	9805	in gene	5.00
NM_001033374	Gm694	gene model 694, (NCBI)	4	140,988,818	140,992,020	-8940	upstream	7.00
NM_009541	Zbtb17	zinc finger and BTB domain containing 17	4	141,000,599	141,023,144	361, 27033	in gene, downstream	6.00
NM_019763	Spen	SPEN homolog, transcriptional regulator (Drosophila)	4	141,023,805	141,094,512	66880	in gene	5.00
NM_001081408	Agmat	agmatine ureohydrolase (agmatinase)	4	141,302,590	141,315,178	-8462	upstream	4.00

Accession No.	Symbol	Gene Title	Chr	Gene Start	Gene End	Interval Dists to Start	Interval Pos	Avg Peak
NM_025994	Efhd2	EF hand domain containing 2	4	141,414,057	141,430,835	1640, 707	in gene, in gene	5.50
NM_028429	Fhad1	forkhead-associated (FHA) phosphopeptide binding domain 1	4	141,472,204	141,478,341	8165, -4987	downstream, upstream	10.50
NM_001081355	Prdm2	PR domain containing 2, with ZNF domain	4	142,717,372	142,802,612	80564	in gene	6.00
NM_011610	Tnfrsf1b	tumor necrosis factor receptor superfamily, member 1b	4	144,802,271	144,836,773	30597	in gene	6.00
NM_001025365	D4Wsu114e	DNA segment, Chr 4, Wayne State University 114, expressed	4	147,234,900	147,242,656	-608	upstream	7.00
NM_010244	Fv1	Friend virus susceptibility 1	4	147,243,088	147,244,467	176	in gene	7.00
NM_133201	Mfn2	mitofusin 2	4	147,247,704	147,278,928	35664	downstream	7.00
NM_011929	Clcn6	chloride channel 6	4	147,380,593	147,412,876	-116	upstream	9.00
NM_010840	Mthfr	5,10-methylenetetrahydrofolate reductase	4	147,415,331	147,433,658	-2339	upstream	9.00
NM_009642	Agtrap	angiotensin II, type I receptor-associated protein	4	147,451,170	147,462,173	8237	in gene	5.00
NM_173401	Fbxo44	F-box protein 44	4	147,526,910	147,533,989	-6507	upstream	6.00
NM_176848	Fbxo2	F-box protein 2	4	147,534,777	147,540,526	5719	in gene	6.00
NM_001039554	Angptl7	angiopoietin-like 7	4	147,869,389	147,874,571	-7317	upstream	11.00
NM_016699	Exosc10	exosome component 10	4	147,932,567	147,956,506	33673	downstream	4.00
NM_009272	Srm	spermidine synthase	4	147,965,622	147,968,728	618	in gene	4.00
NM_022020	Rbp7	retinol binding protein 7, cellular	4	148,823,810	148,829,077	9541	downstream	6.00
NM_023051	Clstn1	calsyntenin 1	4	148,960,747	149,022,008	63925	downstream	4.00
NM_001029837, NM_008840	Pik3cd	phosphatidylinositol 3-kinase catalytic delta polypeptide	4	149,023,277	149,072,798	48126	in gene	4.00
XM_001480126, XM_906187	LOC435818	novel member of the solute carrier family 2 (facilitated glucose transporter) Slc2 family	4	149,523,081	149,542,591	23575	downstream	6.00
NM_001081557	Camta1	calmodulin binding transcription activator 1	4	150,433,635	151,235,855	571599, 497023, 179055, 122031	in gene, in gene, in gene, in gene	7.00
NM_031867	Tas1r1	taste receptor, type 1, member 1	4	151,402,023	151,412,677	117	in gene	4.00
NM_028727	Nol9	nucleolar protein 9	4	151,413,443	151,435,603	-883	upstream	4.00
XM_001000162, XM_987409	EG666980	predicted gene, EG666980	4	151,450,395	151,453,171	-331	upstream	5.00
NM_001004156	Plekhg5	pleckstrin homology domain containing, family G (with RhoGef domain) member 5	4	151,470,828	151,489,509	15812	in gene	6.00
NM_033042	Tnfrsf25	tumor necrosis factor receptor superfamily, member 25	4	151,490,452	151,494,220	-3812	upstream	6.00
NM_019585, NM_207687, NM_207688, NM_207689, NM_207690, NM_207691	Espn	espin	4	151,494,985	151,526,316	39676	downstream	6.00
NM_201226	Lrrc47	leucine rich repeat containing 47	4	153,385,912	153,395,621	11384	downstream	7.00
XR_035126, XR_035127	1190007F08Rik	RIKEN cDNA 1190007F08 gene	4	153,397,420	153,400,259	2963	downstream	7.00
NM_001033455	Ccdc27	coiled-coil domain containing 27	4	153,400,753	153,416,786	19490	downstream	7.00
XM_620148, XM_909777	Megf6	multiple EGF-like-domains 6	4	153,544,822	153,649,830	4986	in gene	6.00
NM_172990	Pank4	pantothenate kinase 4	4	154,338,242	154,354,982	22462	downstream	7.00
NM_175556	Plch2	phospholipase C, eta 2	4	154,357,482	154,385,093	24389	in gene	7.00
NM_001039079, NM_008860	Prkcz	protein kinase C, zeta	4	154,634,229	154,735,500	81452, 55756	in gene, in gene	5.50
NM_177186	A530082C11Rik	RIKEN cDNA A530082C11 gene	4	154,975,525	154,997,449	11515	in gene	5.00
NM_207223	Centb5	centaurin, beta 5	4	155,265,984	155,281,360	11608	in gene	4.00
NM_026033	Gatad1	GATA zinc finger domain containing 1	5	3,639,969	3,647,917	157	in gene	6.00
NM_029141	4930511M11Rik	RIKEN cDNA 4930511M11 gene	5	3,657,004	3,679,416	-9244	upstream	6.00
NM_001003909	Ankib1	ankyrin repeat and IBR domain containing 1	5	3,690,000	3,802,925	-51	upstream	4.00
NM_030675	Krit1	KRIT1, ankyrin repeat containing	5	3,803,189	3,841,359	-213	upstream	4.00
NM_022890	Cldn12	claudin 12	5	5,505,109	5,514,873	366	in gene	6.00
NM_013726	Dbf4	DBF4 homolog (S. cerevisiae)	5	8,397,011	8,422,606	334	in gene	6.00
NM_178766	Slc25a40	solute carrier family 25, member 40	5	8,422,838	8,459,978	-566	upstream	6.00
NM_011806	Dmtf1	cyclin D binding myb-like transcription factor 1	5	9,118,801	9,161,718	-442	upstream	7.00
NM_011348	Sema3e	sema domain, immunoglobulin domain (Ig), short basic domain, secreted, (semaphorin) 3E	5	14,025,276	14,256,689	148676	in gene	6.00
NM_178403	Pus7	pseudouridylate synthase 7 homolog (S. cerevisiae)	5	23,246,514	23,289,479	14647	in gene	5.00
NM_013853	Abcf2	ATP-binding cassette, sub-family F (GCN20), member 2	5	24,071,163	24,083,177	-9511	upstream	6.00
XM_001478455	LOC100042348	similar to ATP synthase, H+ transporting, mitochondrial F0 complex, subunit G	5	24,087,207	24,087,731	-4957	upstream	6.00
	Mirn671	microRNA 671	5	24,097,931	24,098,028	-5243	upstream	6.00
NM_025891	Smarcd3	SWI/SNF related, matrix associated, actin dependent regulator of chromatin, subfamily d, member 3	5	24,098,440	24,107,820	15132, -2996	downstream, upstream	6.50
NM_016736	Nub1	negative regulator of ubiquitin-like proteins 1	5	24,191,633	24,216,373	12207	in gene	4.00
NM_010608	Kcnk3	potassium channel, subfamily K, member 3	5	30,890,543	30,927,643	18305	in gene	5.00
NM_175675	4930471M23Rik	RIKEN cDNA 4930471M23 gene	5	30,950,336	30,962,102	-7232	upstream	6.00
NM_011773	Slc30a3	solute carrier family 30 (zinc transporter), member 3	5	31,390,322	31,395,871	14943	downstream	5.00

Accession No.	Symbol	Gene Title	Chr	Gene Start	Gene End	Interval Dists to Start	Interval Pos	Avg Peak
NM_009535	Yes1	Yamaguchi sarcoma viral (v-yes) oncogene homolog 1	5	32,913,606	32,989,430	-22	upstream	5.00
NM_177298	Pisd	phosphatidylserine decarboxylase	5	33,078,962	33,128,275	55355	downstream	4.00
XM_001473635	LOC672284	similar to NK1 transcription factor related 2-like,b	5	33,773,383	33,776,625	-4335	upstream	6.00
NM_008010	Fgfr3	fibroblast growth factor receptor 3	5	34,064,373	34,079,712	-5061	upstream	6.00
NM_010753	Mxd4	Max dimerization protein 4	5	34,519,229	34,530,359	215	in gene	5.00
NM_001030306	BC037112	cDNA sequence BC037112	5	34,763,500	34,829,105	45268	in gene	6.00
NM_013587	Lrpap1	low density lipoprotein receptor-related protein associated protein 1	5	35,434,155	35,448,346	106	in gene	5.00
NM_175249	2310020A21Rik	RIKEN cDNA 2310020A21 gene	5	36,546,670	36,549,217	3378	downstream	6.00
NM_025725	Ccdc96	coiled-coil domain containing 96	5	36,827,237	36,830,820	6875	downstream	4.00
NM_133724	Cno	cappuccino	5	37,138,613	37,139,918	11054	downstream	6.00
NM_178394	Jakmip1	janus kinase and microtubule interacting protein 1	5	37,442,155	37,516,534	71573	in gene	5.00
NM_022416	Stk32b	serine/threonine kinase 32B	5	37,838,064	38,108,302	159790	in gene	7.00
XM_001474616	LOC100040397	similar to Ubtf protein	5	38,551,062	38,580,819	18842	in gene	6.00
NM_025480	Tmem128	transmembrane protein 128	5	38,651,614	38,660,857	17938	downstream	5.00
NM_172709	Otop1	otopetrin 1	5	38,668,657	38,695,419	895	in gene	5.00
NM_173764	Tapt1	transmembrane anterior posterior transformation 1	5	44,566,398	44,617,845	341	in gene	7.00
NM_172710	2310045A20Rik	RIKEN cDNA 2310045A20 gene	5	53,498,396	53,604,558	382	in gene	5.00
NM_029554	0610040J01Rik	RIKEN cDNA 0610040J01 gene	5	64,203,603	64,290,858	91149	downstream	7.00
NM_145923	AA536743	expressed sequence AA536743	5	64,300,137	64,360,136	65384	downstream	7.00
NM_026667	9130005N14Rik	RIKEN cDNA 9130005N14 gene	5	65,361,351	65,433,113	39049	in gene	7.00
NM_001081321	9030416H16Rik	RIKEN cDNA 9030416H16 gene	5	66,006,499	66,089,095	-5577	upstream	6.00
NM_001081104	Chrna9	cholinergic receptor, nicotinic, alpha polypeptide 9	5	66,358,363	66,368,725	-3195	upstream	5.00
NM_001001980	3732412D22Rik	RIKEN cDNA 3732412D22 gene	5	67,137,079	67,448,398	63945	in gene	5.00
NM_153389	Atp10d	ATPase, Class V, type 10D	5	72,594,582	72,702,533	-182, 1114	upstream, in gene	6.50
NM_011890	Sgcb	sarcoglycan, beta (dystrophin-associated glycoprotein)	5	74,023,988	74,038,970	314	in gene	8.00
XM_001478951, XM_001479212	Spata18	spermatogenesis associated 18	5	74,042,619	74,070,723	-3963	upstream	8.00
NM_177561	Usp46	ubiquitin specific peptidase 46	5	74,396,062	74,464,436	-12	upstream	5.00
NM_026878	Rasl11b	RAS-like, family 11, member B	5	74,591,351	74,595,502	6409	downstream	5.00
NM_010727	Lnx1	ligand of numb-protein X 1	5	74,988,472	75,073,849	-2023	upstream	5.00
XR_035374, XR_035406	EG619945	predicted gene, EG619945	5	75,345,219	75,362,393	22397	downstream	6.00
NM_021099	Kit	kit oncogene	5	75,971,049	76,052,747	11847	in gene	5.00
NM_011626	Tmem165	transmembrane protein 165	5	76,612,905	76,638,269	599	in gene	6.00
NM_026735	Mobkl1a	MOB1, Mps One Binder kinase activator-like 1A (yeast)	5	89,149,896	89,187,480	-72	upstream	9.00
NM_016912, NM_177270	Cdkl2	cyclin-dependent kinase-like 2 (CDC2-related kinase)	5	92,435,101	92,472,044	348	in gene	5.00
XM_001475024	LOC100040574	hypothetical protein LOC100040574	5	92,471,213	92,472,909	483	in gene	5.00
NM_001080794, NM_001080795, NM_001080796, NM_001080797, NM_011816	G3bp2	GTPase activating protein (SH3 domain) binding protein 2	5	92,481,172	92,512,761	41065	downstream	5.00
NM_007644	Scarb2	scavenger receptor class B, member 2	5	92,872,899	92,934,634	53	in gene	4.00
NM_001033478	Gm1381	gene model 1381, (NCBI)	5	93,000,563	93,019,762	-9363	upstream	6.00
NM_144910, NM_178854	Cnot6l	CCR4-NOT transcription complex, subunit 6-like	5	96,504,756	96,591,009	-783	upstream	6.00
NM_013470	Anxa3	annexin A3	5	97,222,434	97,274,987	12286, 12670	in gene, in gene	4.50
XM_982666	EG666266	predicted gene, EG666266	5	97,350,291	97,363,886	-8211, 9277	upstream, in gene	6.00
NM_029947	Prdm8	PR domain containing 8	5	98,609,888	98,616,467	-4992	upstream	6.00
NM_001081107	Hel308	helicase, mus308-like (Drosophila)	5	101,191,167	101,227,619	-9853	upstream	4.00
NM_026826	Mrps18c	mitochondrial ribosomal protein S18C	5	101,227,778	101,233,486	9694	downstream	4.00
NM_172405	Ccdc98	coiled-coil domain containing 98	5	101,233,821	101,249,954	12482	in gene	4.00
NM_172882	Wdfy3	WD repeat and FYVE domain containing 3	5	102,264,604	102,498,940	181532	in gene	5.00
NM_178741	Klhl8	kelch-like 8 (Drosophila)	5	104,291,069	104,340,248	37960, 16498	in gene, in gene	6.00
NM_010097	Sparcl1	SPARC-like 1 (mast9, hevin)	5	104,508,130	104,542,723	-9885	upstream	7.00
XM_001475877	LOC100041145	hypothetical protein LOC100041145	5	104,668,332	104,700,005	31077, 5253	in gene, in gene	6.00
NM_018759	Zfp326	zinc finger protein 326	5	106,305,621	106,344,831	387	in gene	5.00
NM_026062	2900024C23Rik	RIKEN cDNA 2900024C23 gene	5	108,337,357	108,416,052	42036	in gene	6.00
NM_008325	Idua	iduronidase, alpha-L-	5	109,098,384	109,113,573	5360	in gene	7.00
NM_174870	Slc26a1	solute carrier family 26 (sulfate transporter), member 1	5	109,098,897	109,104,588	844	in gene	7.00
XM_001479076	LOC100042616	hypothetical protein LOC100042616	5	109,112,928	109,123,081	19337	downstream	7.00
NM_008146	Golga3	golgi autoantigen, golgin subfamily a, 3	5	110,605,720	110,652,174	54259	downstream	5.00
NM_027922	D5Ertd585e	DNA segment, Chr 5, ERATO Doi 585, expressed	5	110,660,060	110,685,667	-81	upstream	5.00
NM_153570	Noc4l	nucleolar complex associated 4 homolog (S. cerevisiae)	5	111,077,438	111,082,401	2865	in gene	5.00
NM_027156	Ddx51	DEAD (Asp-Glu-Ala-Asp) box polypeptide 51	5	111,082,493	111,089,515	-2957	upstream	5.00
NM_016681	Chek2	CHK2 checkpoint homolog (S. pombe)	5	111,269,036	111,303,152	29588	in gene	10.00
XM_001476544, XM_001481308	Ttc28	tetratricopeptide repeat domain 28	5	111,308,822	111,718,799	258218, 283706, 312890, 320826	in gene, in gene, in gene, in gene	5.75

Accession No.	Symbol	Gene Title	Chr	Gene Start	Gene End	Interval Dists to Start	Interval Pos	Avg Peak
NM_001081235	Mn1	meningioma 1	5	111,847,186	111,886,045	11566	in gene	6.00
NM_021351	Cryba4	crystallin, beta A4	5	112,675,553	112,681,522	3943	in gene	5.00
NM_023695	Crybb1	crystallin, beta B1	5	112,684,841	112,698,602	-7262	upstream	5.00
NM_018783	Tfip11	tuftelin interacting protein 11	5	112,755,389	112,767,093	99	in gene	5.00
NM_019982	Sez6l	seizure related 6 homolog like	5	112,848,176	113,006,205	55677	in gene	4.00
NM_001035531, NM_177078	Adrbk2	adrenergic receptor kinase, beta 2	5	113,339,498	113,444,534	57078, 8134, 374	in gene, in gene, in gene	5.67
NM_007773	Crybb2	crystallin, beta B2	5	113,487,285	113,497,786	8778	in gene	6.00
XM_001479469	LOC100042920	hypothetical protein LOC100042920	5	113,497,074	113,497,791	8783	downstream	6.00
NM_001033428	Gm854	gene model 854, (NCBI)	5	113,655,929	113,668,283	-169	upstream	6.00
	Mirn469	microRNA 469	5	113,772,105	113,772,178	5527	downstream	5.00
NM_001012726	Aym1	activator of yeast meiotic promoters 1	5	113,786,314	113,786,841	-8682	upstream	5.00
NM_146162	Tmem119	transmembrane protein 119	5	114,243,738	114,250,367	-65	upstream	7.00
NM_009151	Selplg	selectin, platelet (p-selectin) ligand	5	114,268,561	114,280,442	14986	downstream	6.00
NM_198109	Ssh1	slingshot homolog 1 (Drosophila)	5	114,392,228	114,443,766	36566, 24982, 17510, 6966	in gene, in gene, in gene, in gene	6.75
NM_026145	Kctd10	potassium channel tetramerisation domain containing 10	5	114,813,583	114,830,480	-416	upstream	8.00
NM_054093	Ube3b	ubiquitin protein ligase E3B	5	114,830,616	114,871,175	280	in gene	8.00
NM_029956	Mmab	methylmalonic aciduria (cobalamin deficiency) type B homolog (human)	5	114,881,043	114,894,036	9924, -6236	in gene, upstream	6.00
NM_023556	Mvk	mevalonate kinase	5	114,894,315	114,910,600	5957	in gene	5.00
NM_001004180	BC057022	cDNA sequence BC057022	5	115,018,260	115,063,227	41068	in gene	4.00
NM_019821	Gltp	glycolipid transfer protein	5	115,119,499	115,140,944	-80	upstream	6.00
XM_001478355, XM_001478612	1500011B03Rik	RIKEN cDNA 1500011B03 gene	5	115,258,205	115,263,985	9729	downstream	5.00
NM_011107	Pla2g1b	phospholipase A2, group IB, pancreas	5	115,916,275	115,924,726	4717	in gene	6.00
XM_485674, XM_988059	Sirt4	sirtuin 4 (silent mating type information regulation 2 homolog) 4 (S. cerevisiae)	5	115,928,019	115,940,415	19423	downstream	6.00
NM_001080808	Ccdc64	coiled-coil domain containing 64	5	116,099,295	116,181,568	66091, 352	in gene, in gene	5.50
NM_007708	Cit	citron	5	116,295,665	116,456,352	13007, 169039	in gene, downstream	5.50
NM_031869	Prkab1	protein kinase, AMP-activated, beta 1 non-catalytic subunit	5	116,463,599	116,474,437	9733	in gene	5.00
NM_011535, NM_198052	Tbx3	T-box 3	5	120,120,678	120,134,610	-1382, -902, 122	upstream, upstream, in gene	6.00
NM_008499	Lhx5	LIM homeobox protein 5	5	120,881,910	120,891,466	-7427	upstream	4.00
NM_133221	Slc24a6	solute carrier family 24 (sodium/potassium/calcium exchanger), member 6	5	120,961,201	120,984,028	23663, 27887	downstream, downstream	6.00
NM_029096	1110008J03Rik	RIKEN cDNA 1110008J03 gene	5	121,059,072	121,062,578	1234, -318	in gene, upstream	7.00
NM_028041	Ddx54	DEAD (Asp-Glu-Ala-Asp) box polypeptide 54	5	121,063,139	121,078,601	-1795, -243	upstream, upstream	7.00
XM_622379, XM_923515	EG545802	predicted gene, EG545802	5	121,670,208	121,704,753	41792	downstream	6.00
XM_132325, XM_990451	AU042671	expressed sequence AU042671	5	121,708,332	121,818,584	3668	in gene	6.00
NM_172722	C330023M02Rik	RIKEN cDNA C330023M02 gene	5	121,847,991	121,890,122	50745	downstream	5.00
NM_026129	Erp29	endoplasmic reticulum protein 29	5	121,894,762	121,902,483	3747	in gene	5.00
NM_009656	Aldh2	aldehyde dehydrogenase 2, mitochondrial	5	122,017,696	122,043,833	9481	in gene	7.00
NM_028037	Acad10	acyl-Coenzyme A dehydrogenase family, member 10	5	122,071,036	122,110,488	104	in gene	6.00
NM_028227	Brap	BRCA1 associated protein	5	122,110,595	122,137,257	-211	upstream	6.00
NM_008507	Sh2b3	SH2B adaptor protein 3	5	122,267,224	122,286,810	2858, -6278	in gene, upstream	5.50
NM_175474	A230106M15Rik	RIKEN cDNA A230106M15 gene	5	122,299,037	122,304,608	-5949, -957, -157	upstream, upstream, upstream	6.33
NM_007804	Cutl2	cut-like 2 (Drosophila)	5	122,310,225	122,497,834	-2251	upstream	6.00
NM_013636	Ppp1cc	protein phosphatase 1, catalytic subunit, gamma isoform	5	122,608,288	122,625,278	-32	upstream	6.00
NM_001039153	Tect1	tectonic 1	5	122,691,524	122,714,469	37	in gene	5.00
NM_001003953, NM_001005866, NM_013910	Fbxl10	F-box and leucine-rich repeat protein 10	5	123,320,677	123,439,101	-163	upstream	6.00
NM_001039723	BC022593	cDNA sequence BC022593	5	123,526,284	123,567,454	-44	upstream	6.00
XM_485681, XM_915421	Wdr66	WD repeat domain 66	5	123,703,865	123,776,806	21330	in gene	6.00
XM_132344, XM_915437	Il31	interleukin 31	5	123,930,166	123,932,110	-5394	upstream	6.00
NM_001033461	Lrrc43	leucine rich repeat containing 43	5	123,939,334	123,958,213	-1830	upstream	6.00
NM_175520	Gpr81	G protein-coupled receptor 81	5	124,326,987	124,330,012	-8612	upstream	5.00
NM_145070	Hip1r	huntingtin interacting protein 1 related	5	124,423,648	124,453,209	26688, 29376	in gene, in gene	7.00
NM_019875	Abcb9	ATP-binding cassette, sub-family B (MDR/TAP), member 9	5	124,511,866	124,545,807	8815	in gene	5.00
NM_025671	Ogfod2	2-oxoglutarate and iron-dependent oxygenase domain containing 2	5	124,562,347	124,565,485	-459, 9349	upstream, downstream	5.50
NM_144509	Arl6ip4	ADP-ribosylation factor-like 6 interacting protein 4	5	124,566,117	124,568,204	-4229, 5579	upstream, downstream	5.50
NM_011256	Pitpnm2	phosphatidylinositol transfer protein, membrane-associated 2	5	124,568,700	124,666,427	104539, 94731	downstream, in gene	5.50
NM_030241	Setd8	SET domain containing (lysine methyltransferase) 8	5	124,889,939	124,912,316	5405	in gene	8.00
NM_181410	Gtf2h3	general transcription factor IIH, polypeptide 3	5	125,029,180	125,046,850	14836	in gene	5.00

Accession No.	Symbol	Gene Title	Chr	Gene Start	Gene End	Interval Dists to Start	Interval Pos	Avg Peak
NM_026486	4432405B04Rik	RIKEN cDNA 4432405B04 gene	5	125,048,793	125,077,658	-4777	upstream	5.00
XM_001474159, XM_915600	Dnahc10	dynein, axonemal, heavy chain 10	5	125,205,455	125,314,678	96316	in gene	5.00
NM_016741	Scarb1	scavenger receptor class B, member 1	5	125,757,460	125,821,444	-4508	upstream	6.00
NM_029752	Bri3bp	Bri3 binding protein	5	125,921,938	125,941,255	24174	downstream	6.00
NM_030210	Aacs	acetoacetyl-CoA synthetase	5	125,956,243	125,997,773	-10131, 37149	upstream, in gene	5.50
NM_133895	Slc15a4	solute carrier family 15, member 4	5	128,076,036	128,097,762	-22	upstream	4.00
NM_175284	Fzd10	frizzled homolog 10 (Drosophila)	5	129,106,981	129,109,968	-2661	upstream	5.00
NM_001081342	Gpr133	G protein-coupled receptor 133	5	129,602,625	129,710,474	110191	downstream	6.00
XM_001471643	LOC100038887	hypothetical protein LOC100038887	5	130,320,838	130,322,231	9482	downstream	6.00
NM_026445	Sumf2	sulfatase modifying factor 2	5	130,322,866	130,339,827	7454	in gene	6.00
NM_011079	Phkg1	phosphorylase kinase gamma 1	5	130,339,311	130,354,954	24634	downstream	6.00
NM_021371	Caln1	calneuron 1	5	130,924,671	131,316,071	380913	in gene	8.00
NM_033572	Wbscr16	Williams-Beuren syndrome chromosome region 16 homolog (human)	5	134,623,946	134,652,637	22701	in gene	7.00
NM_010876	Ncf1	neutrophil cytosolic factor 1	5	134,696,129	134,705,449	-7975	upstream	7.00
NM_001080746, NM_001080747, NM_001080748, NM_001080749, NM_010365	Gtf2i	general transcription factor II I	5	134,713,704	134,790,616	77192	downstream	7.00
NM_001081462, NM_001081463, NM_001081464, NM_001081465, NM_001081466, NM_001081467, NM_001081468, NM_001081469, NM_001081470, NM_020331	Gtf2ird1	general transcription factor II I repeat domain-containing 1	5	134,833,531	134,932,581	68997	in gene	7.00
NM_001039162, NM_009990	Clip2	CAP-GLY domain containing linker protein 2	5	134,965,256	135,028,304	69936	downstream	7.00
NM_010717	Limk1	LIM-domain containing, protein kinase	5	135,131,909	135,164,460	19516	in gene	6.00
NM_024479	Wbscr27	Williams Beuren syndrome chromosome region 27 (human)	5	135,408,243	135,418,507	2893	in gene	5.00
NM_009903	Cldn4	claudin 4	5	135,420,997	135,422,785	11649	downstream	5.00
NM_025375	Wbscr22	Williams Beuren syndrome chromosome region 22	5	135,528,827	135,539,980	-52	upstream	7.00
NM_025362	Wbscr18	Williams-Beuren syndrome chromosome region 18 homolog (human)	5	135,540,076	135,541,235	-44	upstream	7.00
NM_177574	Vps37d	vacuolar protein sorting 37D (yeast)	5	135,548,771	135,554,136	14104	downstream	7.00
NM_146001	Hip1	huntingtin interacting protein 1	5	135,883,896	136,020,985	55545, 47001	in gene, in gene	7.00
NM_008898	Por	P450 (cytochrome) oxidoreductase	5	136,165,084	136,211,195	36452	in gene	7.00
NM_172541	2010310D06Rik	RIKEN cDNA 2010310D06 gene	5	136,211,360	136,220,042	18506	downstream	7.00
NM_013560	Hspb1	heat shock protein 1	5	136,363,929	136,365,307	-8073, -217	upstream, upstream	5.00
XM_355648, XM_916633	Srcrb4d	scavenger receptor cysteine rich domain containing, group B (4 domains)	5	136,436,093	136,450,346	2570	in gene	6.00
NM_011776	Zp3	zona pellucida glycoprotein 3	5	136,455,975	136,464,494	-8199, 15065	upstream, downstream	5.50
NM_023742	Dtx2	deltex 2 homolog (Drosophila)	5	136,470,747	136,508,734	293	in gene	5.00
NM_009986, NM_198602	Cutl1	cut-like 1 (Drosophila)	5	136,724,005	137,043,301	138197, 133	in gene, in gene	5.50
NM_024474	Emid2	EMI domain containing 2	5	137,217,634	137,358,977	72433, 28513	in gene, in gene	6.00
NM_026073	Rabl5	RAB, member of RAS oncogene family-like 5	5	137,384,020	137,389,114	28	in gene	4.00
NM_031405	Ars2	arsenate resistance protein 2	5	137,737,059	137,748,395	-42, -6821	upstream, upstream	5.00
NM_011639	Trip6	thyroid hormone receptor interactor 6	5	137,751,127	137,755,469	7032, 253	downstream, in gene	5.00
NM_031406	Slc12a9	solute carrier family 12 (potassium/chloride transporters), member 9	5	137,755,787	137,774,730	26293, 19514	downstream, downstream	5.00
NM_028753	Pop7	processing of precursor 7, ribonuclease P family, (S. cerevisiae)	5	137,942,667	137,943,659	-277	upstream	6.00
XM_001474488, XM_001479030, XM_983418	EG666372	predicted gene, EG666372	5	137,950,830	137,958,806	-6894	upstream	6.00
NM_031408	Perq1	PERQ amino acid rich, with GYF domain 1	5	137,960,366	137,966,932	9298	downstream	7.00
NM_010312	Gnb2	guanine nucleotide binding protein, beta 2	5	137,969,357	137,974,457	4793	in gene	7.00
NM_027242	2010007H12Rik	RIKEN cDNA 2010007H12 gene	5	138,220,146	138,221,335	7438	downstream	5.00
NM_144913	Bcdin3	bin3, bicoid-interacting 3, homolog (Drosophila)	5	138,223,134	138,227,929	345	in gene	5.00
NM_001005426	Zcwpw1	zinc finger, CW type with PWWP domain 1	5	138,229,103	138,263,849	-1519	upstream	5.00
NM_001044703, NM_001044704, NM_001044705, NM_011757	Zscan21	zinc finger and SCAN domain containing 21	5	138,558,133	138,575,489	22123	downstream	5.00
NM_019747	Zfp113	zinc finger protein 113	5	138,582,665	138,596,916	16660	downstream	5.00
XM_001000706, XM_357518	EG384244	predicted gene, EG384244	5	139,295,436	139,297,573	1316	in gene	5.00
NM_008923	Prkar1b	protein kinase, cAMP dependent regulatory, type I beta	5	139,493,260	139,605,645	98109, 80253	in gene, in gene	5.50
NM_172723	Centa1	centaurin, alpha 1	5	139,747,830	139,782,273	21697	in gene	6.00

Accession No.	Symbol	Gene Title	Chr	Gene Start	Gene End	Interval Dists to Start	Interval Pos	Avg Peak
NM_013702	Uncx4.1	Unc4.1 homeobox (C. elegans)	5	140,019,863	140,024,133	-3511	upstream	5.00
NM_174850	Micall2	MICAL-like 2	5	140,182,650	140,208,788	31380	downstream	6.00
NM_026748	Ints1	integrator complex subunit 1	5	140,227,237	140,251,607	-2025	upstream	5.00
NM_144914	BC019731	cDNA sequence BC019731	5	140,280,906	140,290,196	-7036	upstream	7.00
NM_025604	1810042K04Rik	RIKEN cDNA 1810042K04 gene	5	140,299,548	140,302,797	5565	downstream	7.00
NM_010752	Mad1l1	mitotic arrest deficient 1-like 1	5	140,484,643	140,797,506	246754	in gene	6.00
XM_144611, XM_917042	EG231836	predicted gene, EG231836	5	140,921,599	140,969,093	50849	downstream	4.00
NM_021528	Chst12	carbohydrate sulfotransferase 12	5	140,981,582	141,001,192	-9134	upstream	4.00
NM_028833	Iqce	IQ motif containing E	5	141,139,459	141,178,332	-6468	upstream	7.00
NM_172724, NM_181066	AA881470	EST AA881470	5	141,180,977	141,195,288	3823	in gene	7.00
NM_178702	D930005D10Rik	RIKEN cDNA D930005D10 gene	5	142,960,795	143,027,031	1047	in gene	7.00
NM_146257	Slc29a4	solute carrier family 29 (nucleoside transporters), member 4	5	143,178,055	143,198,443	-359	upstream	6.00
NM_001033312	Fbxl18	F-box and leucine-rich repeat protein 18	5	143,633,468	143,656,896	29504	downstream	5.00
XR_035132, XR_035135	D430018E03Rik	RIKEN cDNA D430018E03 gene	5	143,635,756	143,640,052	12660	downstream	5.00
XM_001478588, XM_001478596	LOC100042605	hypothetical protein LOC100042605	5	143,926,458	143,927,420	284	in gene	5.00
NM_001045482, NM_021326	Rbak	RB-associated KRAB repressor	5	143,933,867	143,942,422	15286	downstream	5.00
NM_177681	Zfp12	zinc finger protein 12	5	143,996,887	144,009,649	22185	downstream	7.00
NM_017467	Zfp316	zinc finger protein 316	5	144,013,400	144,031,618	12546	in gene	7.00
NM_172726	E130309D02Rik	RIKEN cDNA E130309D02 gene	5	144,062,887	144,077,039	1135	in gene	6.00
NM_028379	Zdhhc4	zinc finger, DHHC domain containing 4	5	144,078,170	144,090,905	15001	downstream	6.00
NM_011182	Pscd3	pleckstrin homology, Sec7 and coiled-coil domains 3	5	144,383,403	144,471,115	9077, 50965	in gene, in gene	6.00
NM_029749	Usp42	ubiquitin specific peptidase 42	5	144,471,194	144,493,149	-419	upstream	4.00
NM_133735	Ptcd1	pentatricopeptide repeat domain 1	5	145,908,400	145,927,891	1651	in gene	4.00
NM_178576	Cpsf4	cleavage and polyadenylation specific factor 4	5	145,928,093	145,942,909	-1853	upstream	4.00
NM_028774	Rnf6	ring finger protein (C3H2C3 type) 6	5	147,020,780	147,033,013	-9547	upstream	5.00
NM_153599	Cdk8	cyclin-dependent kinase 8	5	147,043,251	147,114,450	-691, 29581	upstream, in gene	13.00
NM_080795	Lnx2	ligand of numb-protein X 2	5	147,828,679	147,888,105	-12	upstream	8.00
XM_001473557	LOC100039805	hypothetical protein LOC100039805	5	148,222,674	148,234,199	19982	downstream	8.00
NM_028291	Pan3	PAN3 polyA specific ribonuclease subunit homolog (S. cerevisiae)	5	148,242,465	148,360,072	191	in gene	8.00
NM_010228	Flt1	FMS-like tyrosine kinase 1	5	148,373,772	148,537,564	160364	in gene	6.00
NM_007513	Slc7a1	solute carrier family 7 (cationic amino acid transporter, y+ system), member 1	5	149,139,902	149,211,455	38335	in gene	5.00
NM_011908	Ubl3	ubiquitin-like 3	5	149,316,207	149,364,364	-84	upstream	6.00
NM_153572	Katnal1	katanin p60 subunit A-like 1	5	149,687,642	149,740,194	11938, -110	in gene, upstream	8.00
NM_027519	6330406I15Rik	RIKEN cDNA 6330406I15 gene	5	150,214,381	150,234,278	16675	in gene	5.00
NM_080468	Rxfp2	relaxin/insulin-like family peptide receptor 2	5	150,821,250	150,883,911	15358	in gene	5.00
NM_133898	B230342M21Rik	RIKEN cDNA B230342M21 gene	5	151,374,219	151,397,075	-2093	upstream	6.00
NM_011134	Pon1	paraoxonase 1	6	5,118,105	5,143,824	4208	in gene	5.00
XM_287555, XM_983072	Thsd7a	thrombospondin, type I, domain containing 7A	6	12,265,952	12,699,188	146788	in gene	6.00
NM_023626	Ing3	inhibitor of growth family, member 3	6	21,899,615	21,926,037	609, 24353	in gene, in gene	5.50
NM_053116	Wnt16	wingless-related MMTV integration site 16	6	22,238,230	22,248,522	1162	in gene	5.00
NM_028900	Gcc1	golgi coiled coil 1	6	28,366,691	28,371,724	-2532	upstream	7.00
NM_007480	Arf5	ADP-ribosylation factor 5	6	28,373,640	28,376,499	616	in gene	7.00
NM_019569	Fscn3	fascin homolog 3, actin-bundling protein, testicular (Strongylocentrotus purpuratus)	6	28,377,901	28,388,622	-3645	upstream	7.00
NM_001081185	Flnc	filamin C, gamma (actin binding protein 280)	6	29,383,153	29,411,888	34991	downstream	6.00
NM_025381	Atp6v1f	ATPase, H+ transporting, lysosomal V1 subunit F	6	29,417,783	29,420,509	361	in gene	6.00
XM_001000046, XM_988597	EG668210	predicted gene, EG668210	6	29,421,527	29,423,465	-3383	upstream	6.00
NM_001029985	Crim2	cysteine rich BMP regulator 2 (chordin like)	6	29,432,036	29,457,937	21777	in gene	6.00
NM_009459	Ube2h	ubiquitin-conjugating enzyme E2H	6	30,162,965	30,254,345	68601	in gene	6.00
XR_035177, XR_035288	AB041803	cDNA sequence AB041803	6	31,115,508	31,168,433	35089	in gene	6.00
XM_001472135, XM_001477679	LOC620104	hypothetical protein LOC620104	6	31,652,245	31,796,531	37315	in gene	6.00
NM_175750	Plxna4	plexin A4	6	32,100,122	32,538,192	408320, 91936	in gene, in gene	7.00
NM_009148	Exoc4	exocyst complex component 4	6	33,199,150	33,922,943	618338	in gene	5.00
NM_001025573	2010107G12Rik	RIKEN cDNA 2010107G12 gene	6	34,895,294	34,927,999	25567	in gene	6.00
NM_178661	Creb3l2	cAMP responsive element binding protein 3-like 2	6	37,281,021	37,392,139	87179	in gene	9.00
NM_177200	Svopl	SV2 related protein homolog (rat)-like	6	37,965,273	37,996,996	10500	in gene	5.00
NM_080467	Atp6v0a4	ATPase, H+ transporting, lysosomal V0 subunit A4	6	37,998,483	38,074,574	-7442	upstream	5.00
NM_172477	Dennd2a	DENN/MADD domain containing 2A	6	39,412,377	39,507,833	101113, 74777	downstream, in gene	6.00
NM_001010930, NM_010270	Mrps33	mitochondrial ribosomal protein S33	6	39,751,803	39,760,935	55	in gene	6.00
NM_001004182	EG434008	predicted gene, EG434008	6	40,060,252	40,198,352	-908	upstream	6.00
NM_175408	Tmem139	transmembrane protein 139	6	42,211,958	42,214,552	2826	downstream	5.00
NM_007610	Casp2	caspase 2	6	42,215,038	42,232,495	-254	upstream	5.00
NM_013861	Tpk1	thiamine pyrophosphokinase	6	43,295,006	43,616,174	82798, -2	in gene, upstream	6.50

Accession No.	Symbol	Gene Title	Chr	Gene Start	Gene End	Interval Dists to Start	Interval Pos	Avg Peak
NM_001004357, NM_025771	Cntnap2	contactin associated protein-like 2	6	45,010,087	47,251,368	2240593	in gene	4.00
XR_030568, XR_031849	A230106D06Rik	RIKEN cDNA A230106D06 gene	6	47,893,170	47,903,153	9998	downstream	7.00
NM_178898	AI894139	expressed sequence AI894139	6	47,903,446	47,915,296	-278	upstream	7.00
XM_001474507, XM_904306, XM_973126	Zfp746	zinc finger protein 746	6	48,012,394	48,070,426	34266	in gene	8.00
NM_020589	Zfp467	zinc finger protein 467	6	48,387,585	48,395,606	1590	in gene	5.00
NM_173428	Sspo	SCO-spondin	6	48,398,228	48,451,234	-4212	upstream	5.00
NM_001081273	1600015I10Rik	RIKEN cDNA 1600015I10 gene	6	48,879,892	48,883,686	10620	downstream	4.00
NM_026629	2410066E13Rik	RIKEN cDNA 2410066E13 gene	6	54,631,766	54,650,400	23738	downstream	7.00
NM_172729	Nod1	nucleotide-binding oligomerization domain containing 1	6	54,873,943	54,922,405	-59	upstream	7.00
XM_132538, XM_907872	5830411G16Rik	RIKEN cDNA 5830411G16 gene	6	56,664,832	56,711,910	544	in gene	6.00
NM_146168	AW146242	expressed sequence AW146242	6	57,702,258	57,775,119	28575	in gene	5.00
NM_009075	Rpia	ribose 5-phosphate isomerase A	6	70,715,714	70,742,169	2761	in gene	6.00
NM_024288	Rmnd5a	required for meiotic nuclear division 5 homolog A (S. cerevisiae)	6	71,341,245	71,390,482	-142	upstream	9.00
NM_009088	Rpo1-4	RNA polymerase 1-4	6	71,859,067	71,929,354	61661	in gene	4.00
NM_147779	Sftpb	surfactant associated protein B	6	72,253,239	72,264,364	4329	in gene	6.00
NM_019802	Ggcx	gamma-glutamyl carboxylase	6	72,364,327	72,380,701	-87, 24761	upstream, downstream	6.50
NM_145569	Mat2a	methionine adenosyltransferase II, alpha	6	72,382,793	72,389,552	464	in gene	7.00
XM_355785, XM_911211	Sh2d6	SH2 domain containing 6	6	72,463,646	72,470,621	7981	downstream	5.00
NM_013586	Loxl3	lysyl oxidase-like 3	6	82,984,218	83,002,558	19116	downstream	5.00
NM_019752	Htra2	HtrA serine peptidase 2	6	83,001,276	83,004,565	1231	in gene	5.00
NM_007517	Aup1	ancient ubiquitous protein	6	83,004,647	83,007,676	-1313	upstream	5.00
NM_010692	Lbx2	ladybird homeobox homolog 2 (Drosophila)	6	83,036,359	83,038,235	-7623	upstream	6.00
NR_002871	Vax2os2	Vax2 opposite strand transcript 2	6	83,642,800	83,662,195	3315	in gene	6.00
NR_002873	Vax2os1	Vax2 opposite strand transcript 1	6	83,652,120	83,660,926	2046	in gene	6.00
NM_011912	Vax2	ventral anterior homeobox containing gene 2	6	83,661,258	83,688,298	-2378	upstream	6.00
XM_001479937, XM_989266	LOC676366	hypothetical protein LOC676366	6	83,864,352	83,865,890	-6110	upstream	6.00
NM_008717	Zfml	zinc finger, matrin-like	6	83,864,361	83,936,855	7639	in gene	6.00
NM_001003955, NM_177466	Rab11fip5	RAB11 family interacting protein 5 (class I)	6	85,284,956	85,324,628	5348	in gene	7.00
NM_053096	Cml2	camello-like 2	6	85,815,416	85,819,131	12080	downstream	5.00
NM_031199	Tgfa	transforming growth factor alpha	6	86,145,410	86,225,151	-114	upstream	6.00
NM_025823	Pcyox1	prenylcysteine oxidase 1	6	86,336,851	86,347,114	-7238	upstream	5.00
NM_011585	Tia1	cytotoxic granule-associated RNA binding protein 1	6	86,354,308	86,383,399	44	in gene	5.00
NM_011865	Pcbp1	poly(rC) binding protein 1	6	86,474,488	86,476,161	-543	upstream	8.00
NM_026414	Asprv1	aspartic peptidase, retroviral-like 1	6	86,578,168	86,579,704	8584	downstream	5.00
NM_001037727, NM_175476	Arhgap25	Rho GTPase activating protein 25	6	87,409,379	87,483,229	60989, 58109	in gene, in gene	6.00
NM_024251	2010301N04Rik	RIKEN cDNA 2010301N04 gene	6	87,578,423	87,622,141	35581	in gene	6.00
NM_133933	Rpn1	ribophorin I	6	88,034,510	88,055,298	16226, 28210	in gene, downstream	6.00
NM_023060	Eefsec	eukaryotic elongation factor, selenocysteine-tRNA-specific	6	88,207,328	88,396,533	62021	in gene	5.00
NM_153162	Txnrd3	thioredoxin reductase 3	6	89,593,982	89,625,523	10754	in gene	6.00
NM_023184	Klf15	Kruppel-like factor 15	6	90,412,620	90,425,203	1060, 4772	in gene, in gene	5.00
NM_001081146	Prickle2	prickle-like 2 (Drosophila)	6	92,325,940	92,656,178	291554	in gene	10.00
NM_008377	Lrig1	leucine-rich repeats and immunoglobulin-like domains 1	6	94,554,523	94,650,139	72179	in gene	5.00
NM_011507	Suclg2	succinate-Coenzyme A ligase, GDP-forming, beta subunit	6	95,424,140	95,668,782	236814	in gene	5.00
NM_177233	C130034I18Rik	RIKEN cDNA C130034I18 gene	6	96,781,203	97,010,405	33210	in gene	5.00
NM_001081111	Tmf1	TATA element modulatory factor 1	6	97,105,260	97,129,118	-690	upstream	5.00
NM_011666	Ube1c	ubiquitin-activating enzyme E1C	6	97,134,022	97,155,337	25529	downstream	5.00
XM_001480618	LOC100043692	similar to metallopanstimulin	6	97,840,202	97,840,567	8455	downstream	13.00
XM_284224, XM_916337	Gm765	gene model 765, (NCBI)	6	98,188,071	98,292,666	68453	in gene	4.00
NM_053202	Foxp1	forkhead box P1	6	98,880,084	99,113,012	233204	downstream	6.00
NM_018884	Pdzrn3	PDZ domain containing RING finger 3	6	101,099,608	101,327,891	96291, -2717	in gene, upstream	6.50
NM_011498	Bhlhb2	basic helix-loop-helix domain containing, class B2	6	108,610,623	108,616,919	433	in gene	5.00
NM_133923	Ttll3	tubulin tyrosine ligase-like family, member 3	6	113,347,752	113,364,558	20237	downstream	6.00
XM_132830, XM_924644	Rpusd3	RNA pseudouridylate synthase domain containing 3	6	113,365,313	113,369,334	1345	in gene	6.00
NM_178373	Cidec	cell death-inducing DFFA-like effector c	6	113,374,630	113,385,749	17760	downstream	6.00
NM_172161	Irak2	interleukin-1 receptor-associated kinase 2	6	113,588,497	113,644,745	58687	downstream	6.00
NM_001033463	Tatdn2	TatD DNase domain containing 2	6	113,647,493	113,661,062	-309	upstream	6.00
NM_028835	Atg7	autophagy-related 7 (yeast)	6	114,593,143	114,809,954	81833	in gene	6.00
NM_177683	Vgll4	vestigial like 4 (Drosophila)	6	114,812,108	114,871,770	47290	in gene	6.00
NM_138311	H1foo	H1 histone family, member O, oocyte-specific	6	115,894,957	115,900,251	10659	downstream	6.00
NM_026376	Plxnd1	plexin D1	6	115,905,758	115,944,982	39366	downstream	6.00

Accession No.	Symbol	Gene Title	Chr	Gene Start	Gene End	Interval Dists to Start	Interval Pos	Avg Peak
NM_033648	Fxyd4	FXYD domain-containing ion transport regulator 4	6	117,883,577	117,887,353	-1975	upstream	6.00
XM_001479962, XM_986257	Rasgef1a	RasGEF domain family, member 1A	6	117,960,827	118,041,565	66693	in gene	5.00
NM_194339	Bms1	BMS1 homolog, ribosome assembly protein (yeast)	6	118,333,399	118,369,435	-133	upstream	9.00
NM_028335	Zfp248	zinc finger protein 248	6	118,377,337	118,405,524	35956	downstream	9.00
NM_053204, NM_178085	Erc1	ELKS/RAB6-interacting/CAST family member 1	6	119,520,814	119,798,168	-72	upstream	10.00
NM_016970	Klrg1	killer cell lectin-like receptor subfamily G, member 1	6	122,220,614	122,232,851	2979	in gene	6.00
NM_008479	Lag3	lymphocyte-activation gene 3	6	124,854,379	124,861,723	-6629	upstream	6.00
NM_026988	Ptms	parathymosin	6	124,863,699	124,867,949	-403	upstream	6.00
NM_010736	Ltbr	lymphotoxin B receptor	6	125,256,594	125,263,872	4416	in gene	5.00
NM_011324	Scnn1a	sodium channel, nonvoltage-gated, type I, alpha	6	125,271,871	125,294,961	6817	in gene	5.00
NM_011609	Tnfrsf1a	tumor necrosis factor receptor superfamily, member 1a	6	125,299,773	125,312,503	10291	in gene	7.00
NM_198604	Plekhg6	pleckstrin homology domain containing, family G (with RhoGef domain) member 6	6	125,312,669	125,330,522	20458	downstream	7.00
NM_007657	Cd9	CD9 antigen	6	125,410,284	125,444,773	25813	in gene	6.00
NM_011708	Vwf	Von Willebrand factor homolog	6	125,502,981	125,636,695	62491, 128091	in gene, in gene	6.50
NM_010595	Kcna1	potassium voltage-gated channel, shaker-related subfamily, member 1	6	126,586,481	126,595,819	-1541	upstream	5.00
XM_917767, XM_990939	EG640703	predicted gene, EG640703	6	127,231,839	127,287,473	1537	in gene	6.00
NM_007376	Pzp	pregnancy zone protein	6	128,433,585	128,476,738	24418	in gene	6.00
NM_153590	Klre1	killer cell lectin-like receptor family E member 1	6	129,527,911	129,535,845	-7127	upstream	5.00
XM_001480602	2700089E24Rik	RIKEN cDNA 2700089E24 gene	6	133,054,046	133,060,882	1682	in gene	5.00
NM_026345	Mansc1	MANSC domain containing 1	6	134,559,228	134,582,506	-8806	upstream	4.00
NM_026371	Loh12cr1	loss of heterozygosity, 12, chromosomal region 1 homolog (human)	6	134,590,999	134,661,188	313	in gene	4.00
XM_355823, XM_905672	Apold1	apolipoprotein L domain containing 1	6	134,932,019	134,936,854	-435	upstream	5.00
NM_010128	Emp1	epithelial membrane protein 1	6	135,312,949	135,333,191	6907	in gene	5.00
XM_001481171	LOC100043893	similar to ribosomal protein L36a	6	136,437,249	136,437,727	9503	downstream	4.00
NM_145067	Gucy2c	guanylate cyclase 2c	6	136,645,806	136,730,263	62359	in gene	5.00
NM_011216	Ptpro	protein tyrosine phosphatase, receptor type, O	6	137,200,986	137,411,754	110174	in gene	4.00
XM_908334, XM_979562	Etnk1	ethanolamine kinase 1	6	143,084,110	143,157,066	40114	in gene	6.00
XM_001479070	LOC100042879	hypothetical protein LOC100042879	6	145,491,811	145,562,839	20311	in gene	5.00
NM_027760	Rassf8	Ras association (RalGDS/AF-6) domain family 8	6	145,756,903	145,766,104	-3927	upstream	7.00
NM_024469	Bhlhb3	basic helix-loop-helix domain containing, class B3	6	145,811,257	145,813,860	-396	upstream	7.00
NM_025620	2210417D09Rik	RIKEN cDNA 2210417D09 gene	6	146,981,059	146,982,040	-5155	upstream	7.00
NM_198967	Tmtc1	transmembrane and tetratricopeptide repeat containing 1	6	148,185,194	148,392,874	196085	in gene	5.00
NM_019643	Tera	teratocarcinoma expressed, serine rich	6	148,869,579	148,894,954	21514	in gene	5.00
XM_132975, XM_901291, XM_921354, XM_921360	C730024G19Rik	RIKEN cDNA C730024G19 gene	6	149,106,099	149,137,234	-1054	upstream	6.00
NM_011102	Prkcc	protein kinase C, gamma	7	3,303,658	3,331,005	28710	downstream	5.00
NM_133189	Cacng7	calcium channel, voltage-dependent, gamma subunit 7	7	3,333,055	3,367,810	-687	upstream	5.00
NM_001001454, NM_021324	Ttyh1	tweety homolog 1 (Drosophila)	7	4,071,188	4,086,947	8012	in gene	5.00
NM_172736	Leng8	leukocyte receptor cluster (LRC) member 8	7	4,088,658	4,099,775	-9458	upstream	5.00
NM_001003920	Brsk1	BR serine/threonine kinase 1	7	4,642,530	4,667,599	14302	in gene	6.00
NM_177887	BC022651	cDNA sequence BC022651	7	4,658,434	4,676,853	20021	downstream	6.00
NM_170759	Zfp628	zinc finger protein 628	7	4,870,378	4,873,600	7078	downstream	5.00
NM_201355	Nat14	N-acetyltransferase 14	7	4,874,217	4,876,604	3239	downstream	5.00
NM_173008	A430110N23Rik	RIKEN cDNA A430110N23 gene	7	4,877,446	4,896,399	10	in gene	5.00
NM_133204	Zfp371	zinc finger protein 371	7	6,173,880	6,191,014	22312	downstream	5.00
XM_892523, XM_912420	EG627821	predicted gene, EG627821	7	6,204,312	6,233,463	-8120	upstream	5.00
NM_001025163, NM_177888	Zfp78	zinc finger protein 78	7	6,316,015	6,335,315	20001	downstream	6.00
NM_175247	Zfp28	zinc finger protein 28	7	6,336,037	6,348,462	-21	upstream	6.00
NM_021323	Usp29	ubiquitin specific peptidase 29	7	6,683,452	6,919,931	120708	in gene	5.00
NM_011334	Clcn4-2	chloride channel 4-2	7	7,235,027	7,252,222	2510	in gene	5.00
NM_007770	Crx	cone-rod homeobox containing gene	7	16,451,298	16,465,243	11430	in gene	4.00
NM_009156	Sepw1	selenoprotein W, muscle 1	7	16,502,557	16,507,720	2568, 136	in gene, in gene	5.50
NM_148946	Slc8a2	solute carrier family 8 (sodium/calcium exchanger), member 2	7	16,715,649	16,745,860	23759	in gene	7.00
NM_008627	Mrg2	myeloid ecotropic viral integration site-related gene 2	7	16,760,729	16,771,853	15783	downstream	4.00

Accession No.	Symbol	Gene Title	Chr	Gene Start	Gene End	Interval Dists to Start	Interval Pos	Avg Peak
NM_027883	Dhx34	DEAH (Asp-Glu-Ala-His) box polypeptide 34	7	16,782,572	16,807,342	30830	downstream	4.00
NM_133234	Bbc3	Bcl-2 binding component 3	7	16,894,987	16,903,683	7669	in gene	5.00
NM_019748	Sae1	SUMO1 activating enzyme subunit 1	7	16,912,403	16,973,134	70478	downstream	5.00
NM_009201	Slc1a5	solute carrier family 1 (neutral amino acid transporter), member 5	7	17,366,695	17,383,623	17273	downstream	6.00
NM_178900	Prkd2	protein kinase D2	7	17,428,414	17,455,810	32130	downstream	10.00
NM_009499	Vasp	vasodilator-stimulated phosphoprotein	7	19,843,066	19,858,496	2848	in gene	7.00
NM_177691	C79127	expressed sequence C79127	7	19,862,156	19,865,398	9750	downstream	7.00
NM_001025364, NM_013648	Rtn2	reticulon 2 (Z-band associated protein)	7	19,868,016	19,881,509	13360	in gene	6.00
	Trsp	tRNA phosphoserine	7	19,886,593	19,886,678	-5217, 8991	upstream, downstream	6.00
NM_008036	Fosb	FBJ osteosarcoma oncogene B	7	19,888,045	19,895,394	14018, -190	downstream, upstream	6.00
XM_987344	D830036C21Rik	RIKEN cDNA D830036C21 gene	7	19,905,466	19,927,574	31990	downstream	6.00
NM_172279	Mark4	MAP/microtubule affinity-regulating kinase 4	7	20,011,424	20,043,843	3555	in gene	6.00
NM_008990	Pvrl2	poliovirus receptor-related 2	7	20,302,010	20,334,818	-942	upstream	6.00
NM_020486	Bcam	basal cell adhesion molecule	7	20,341,487	20,355,881	20121	downstream	6.00
NM_033601	Bcl3	B-cell leukemia/lymphoma 3	7	20,393,811	20,408,064	32	in gene	5.00
NM_001045486	Zfp180	zinc finger protein 180	7	24,866,963	24,892,234	-115, 18237	upstream, in gene	6.50
NM_027732	Dmrtc2	doublesex and mab-3 related transcription factor like family C2	7	25,655,079	25,662,666	13961	downstream	6.00
NM_010155	Erf	Ets2 repressor factor	7	26,027,579	26,035,684	2164	in gene	6.00
NM_177102	A830041P22Rik	RIKEN cDNA A830041P22 gene	7	26,454,161	26,460,185	-8903	upstream	5.00
NM_172148	BC028440	cDNA sequence BC028440	7	26,466,177	26,471,577	2911	in gene	5.00
NM_011577	Tgfb1	transforming growth factor, beta 1	7	26,472,021	26,490,015	-2933	upstream	5.00
NM_009465	Axl	AXL receptor tyrosine kinase	7	26,542,292	26,573,560	17896	in gene	5.00
NM_007434	Akt2	thymoma viral proto-oncogene 2	7	28,390,877	28,424,473	26904	in gene	5.00
NM_030562	Lrfn1	leucine rich repeat and fibronectin type III domain containing 1	7	29,237,057	29,247,151	15215	downstream	4.00
NM_022432	Sirt2	sirtuin 2 (silent mating type information regulation 2, homolog) 2 (S. cerevisiae)	7	29,551,802	29,573,679	18854	in gene	5.00
NM_021894	Capn12	calpain 12	7	29,666,676	29,678,604	5612	in gene	5.00
NM_021895	Actn4	actinin alpha 4	7	29,678,273	29,747,299	75011, 49059	downstream, in gene	5.50
NM_001033525	Kcnk6	potassium inwardly-rectifying channel, subfamily K, member 6	7	30,006,947	30,017,512	2760	in gene	5.00
NM_029887	Yif1b	Yip1 interacting factor homolog B (S. cerevisiae)	7	30,023,372	30,032,548	-8620	upstream	5.00
NM_001081028	Sipa1l3	signal-induced proliferation-associated 1 like [illegible]	7	30,105,397	30,290,479	97039	in gene	5.00
NM_023750	Zfp84	zinc finger protein 84	7	30,553,571	30,566,438	2557, 10365	in gene, in gene	5.50
XM_001481066	LOC100043794	hypothetical protein LOC100043794	7	30,569,017	30,569,823	5887	downstream	6.00
NM_013705	Zfp30	zinc finger protein 30	7	30,569,809	30,579,559	-5873	upstream	6.00
NM_152814	Zfp566	zinc finger protein 566	7	30,862,356	30,875,529	-8215	upstream	5.00
NM_011981	Zfp260	zinc finger protein 260	7	30,880,095	30,892,633	3649	in gene	5.00
NM_029274	Wbp7	WW domain binding protein 7	7	31,353,874	31,373,745	-271	upstream	6.00
NM_021397	Zbtb32	zinc finger and BTB domain containing 32	7	31,374,700	31,377,961	3945	downstream	6.00
NM_026815	Upk1a	uroplakin 1A	7	31,388,111	31,397,753	-663	upstream	6.00
NM_025628	Cox6b1	cytochrome c oxidase, subunit VIb polypeptide 1	7	31,401,993	31,411,170	12754	downstream	6.00
NM_020563	Apbh	androgen-binding protein eta	7	32,075,539	32,076,835	3	in gene	5.00
NM_025948	Lsm14a	LSM14 homolog A (SCD6, S. cerevisiae)	7	35,129,738	35,174,559	-273	upstream	7.00
NM_008820	Pepd	peptidase D	7	35,691,920	35,829,727	32528	in gene	5.00
NM_178704	Dpy19l3	dpy-19-like 3 (C. elegans)	7	36,473,853	36,539,417	-10055	upstream	4.00
XM_001002219, XM_145521	zfp507	zinc finger protein 507	7	36,557,362	36,623,898	74426	downstream	4.00
XM_001480192	LOC100043453	hypothetical protein LOC100043453	7	38,556,976	38,562,610	-1401	upstream	4.00
NM_028166	1600014C10Rik	RIKEN cDNA 1600014C10 gene	7	38,968,835	38,982,581	93, 2093, 5917	in gene, in gene, in gene	6.00
NM_177693	Lim2	lens intrinsic membrane protein 2	7	50,685,471	50,691,361	13998	downstream	4.00
NM_024253	Nkg7	natural killer cell group 7 sequence	7	50,692,508	50,693,616	6961	downstream	4.00
NM_028849	Cldnd2	claudin domain containing 2	7	50,696,186	50,698,690	3283	downstream	4.00
NM_026695	Etfb	electron transferring flavoprotein, beta polypeptide	7	50,699,513	50,713,152	-44, 22471	upstream, downstream	5.50
XM_355890, XM_914186	2210412E05Rik	RIKEN cDNA 2210412E05 gene	7	50,718,615	50,726,787	3369	in gene	7.00
XM_001474467, XM_357746	A230106M20Rik	RIKEN cDNA A230106M20 gene	7	50,728,277	50,745,393	23409	downstream	7.00
NM_001008549	BC043301	cDNA sequence BC043301	7	50,814,176	50,830,831	3568	in gene	6.00
NM_010215	Il4i1	interleukin 4 induced 1	7	52,091,659	52,096,179	12293	downstream	9.00
NM_001042655	Tbc1d17	TBC1 domain family, member 17	7	52,096,146	52,104,449	497	in gene	9.00
NM_026270	Akt1s1	AKT1 substrate 1 (proline-rich)	7	52,104,597	52,110,780	-645	upstream	9.00
	Mirn707	microRNA 707	7	52,105,068	52,105,140	-1116	upstream	9.00
NM_029410	Bcl2l12	BCL2-like 12 (proline rich)	7	52,246,595	52,252,949	-5035	upstream	9.00
NM_016849	Irf3	interferon regulatory factor 3	7	52,253,030	52,258,218	4954	in gene	9.00
XM_001003082, XM_914632	Prr12	proline rich 12	7	52,282,933	52,308,251	-677	upstream	6.00
NM_022999	Prrg2	proline-rich Gla (G-carboxyglutamic acid) polypeptide 2	7	52,308,974	52,317,022	8094	downstream	6.00
NM_025533	Nosip	nitric oxide synthase interacting protein	7	52,317,863	52,332,786	-8935	upstream	6.00

Accession No.	Symbol	Gene Title	Chr	Gene Start	Gene End	Interval Dists to Start	Interval Pos	Avg Peak
NM_026555	Rcn3	reticulocalbin 3, EF-hand calcium binding domain	7	52,338,283	52,347,533	-3475	upstream	5.00
NM_010189	Fcgrt	Fc receptor, IgG, alpha chain transporter	7	52,348,434	52,358,462	7454, -1154	in gene, upstream	6.00
NM_175130	Trpm4	transient receptor potential cation channel, subfamily M, member 4	7	52,558,002	52,589,112	33581	downstream	4.00
NM_001033243	BC013491	cDNA sequence BC013491	7	53,183,768	53,204,326	-10024, -5304	upstream, upstream	6.00
XM_001476814, XM_001477482	Otog	otogelin	7	53,496,373	53,565,539	14795	in gene	4.00
NM_010866	Myod1	myogenic differentiation 1	7	53,631,852	53,634,465	-780	upstream	5.00
NM_013643	Ptpn5	protein tyrosine phosphatase, non-receptor type 5	7	54,333,172	54,389,054	47422	in gene	6.00
NM_013808	Csrp3	cysteine and glycine-rich protein 3	7	56,085,768	56,099,021	2813	in gene	5.00
NM_146190	Tubgcp5	tubulin, gamma complex associated protein 5	7	63,049,518	63,086,817	-30	upstream	6.00
NM_001038701, NM_008071	Gabrb3	gamma-aminobutyric acid (GABA-A) receptor, subunit beta 3	7	64,845,904	65,084,172	592	in gene	6.00
NM_001039104, NM_018752	Trpm1	transient receptor potential cation channel, subfamily M, member 1	7	71,298,814	71,414,658	55938	in gene	7.00
	Mirn211	microRNA 211	7	71,350,691	71,350,796	4061	downstream	7.00
NM_172742	BB128963	expressed sequence BB128963	7	71,432,556	71,485,692	61129	downstream	5.00
XM_885802, XM_976717	Mtmr15	myotubularin related protein 15	7	71,491,644	71,518,978	25293	in gene	5.00
NM_026483	Mphosph10	M-phase phosphoprotein 10 (U3 small nucleolar ribonucleoprotein)	7	71,521,427	71,537,122	66, -366	in gene, upstream	5.50
NM_028626	Mcee	methylmalonyl CoA epimerase	7	71,537,657	71,557,005	-601, -169	upstream, upstream	5.50
XM_975528	EG665234	predicted gene, EG665234	7	72,724,884	72,789,503	351	in gene	5.00
NM_172310	Tarsl2	threonyl-tRNA synthetase-like 2	7	72,789,800	72,836,977	-648	upstream	5.00
XM_355911, XM_886136, XM_905687, XM_919493	Pcsk6	proprotein convertase subtilisin/kexin type 6	7	73,007,022	73,195,272	110738	in gene	6.00
NM_021336	Snrpa1	small nuclear ribonucleoprotein polypeptide A'	7	73,205,455	73,219,470	11569	in gene	7.00
NM_024439	H47	histocompatibility 47	7	73,224,535	73,234,291	-7511	upstream	7.00
NM_146191	Lrrk1	leucine-rich repeat kinase 1	7	73,403,631	73,533,227	78219, 77835	in gene, in gene	7.00
XM_620510, XM_907730	Lass3	longevity assurance homolog 3 (S. cerevisiae)	7	73,888,390	73,966,728	14602	in gene	5.00
NM_025905	Ttc23	tetratricopeptide repeat domain 23	7	74,792,327	74,871,460	57668, 68121	in gene, in gene	5.00
NM_009697, NM_183261	Nr2f2	nuclear receptor subfamily 2, group F, member 2	7	77,496,836	77,511,632	9072, 4864	in gene, in gene	6.50
XM_001478880	LOC100042833	hypothetical protein LOC100042833	7	77,514,395	77,516,324	9556	downstream	6.00
XM_978127, XM_980382	EG665610	predicted gene, EG665610	7	80,762,744	80,763,274	-262	upstream	8.00
XM_133543, XM_907140	Akap13	A kinase (PRKA) anchor protein 13	7	82,600,185	82,899,495	178727	in gene	5.00
NM_182782	Klhl25	kelch-like 25 (Drosophila)	7	82,993,283	83,012,377	11349	in gene	4.00
NM_018811	Abhd2	abhydrolase domain containing 2	7	86,418,152	86,506,487	24, 3112	in gene, in gene	6.50
NM_008486	Anpep	alanyl (membrane) aminopeptidase	7	86,966,689	86,987,170	4930, -5854	in gene, upstream	6.50
NM_133952	Unc45a	unc-45 homolog A (C. elegans)	7	87,470,178	87,485,094	-3618	upstream	6.00
NM_026812	Hddc3	HD domain containing 3	7	87,488,023	87,490,983	689	in gene	6.00
NM_001081454, NM_011046	Furin	furin (paired basic amino acid cleaving enzyme)	7	87,534,080	87,550,317	3096, 2301	in gene, in gene	6.00
XM_001479473, XM_001479496	Wdr73	WD repeat domain 73	7	88,035,609	88,046,155	13819	downstream	5.00
NM_054085	Alpk3	alpha-kinase 3	7	88,202,486	88,250,498	35914	in gene	4.00
NM_025997	2610204K14Rik	RIKEN cDNA 2610204K14 gene	7	88,907,839	88,914,376	577	in gene	6.00
NM_177894	1700129I04Rik	RIKEN cDNA 1700129I04 gene	7	89,781,470	89,797,038	-98	upstream	6.00
NM_175317	Eftud1	elongation factor Tu GTP binding domain containing 1	7	89,797,151	89,926,362	-15, 67745	upstream, in gene	6.00
NM_177695	Tmc3	transmembrane channel-like gene family 3	7	90,733,441	90,772,219	21391	in gene	6.00
NM_030705	Mesdc1	mesoderm development candidate 1	7	91,029,005	91,032,796	-164	upstream	7.00
NM_023403	Mesdc2	mesoderm development candiate 2	7	91,040,767	91,050,042	-7807	upstream	7.00
NM_030728	9930013L23Rik	RIKEN cDNA 9930013L23 gene	7	91,083,293	91,235,012	68068, -220	in gene, upstream	7.00
NM_009982	Ctsc	cathepsin C	7	95,426,645	95,459,398	-229	upstream	5.00
XM_357814	EG384719	predicted gene, EG384719	7	97,255,987	97,271,485	-6691	upstream	6.00
XM_896611, XM_922997	2310010J17Rik	RIKEN cDNA 2310010J17 gene	7	97,273,368	97,278,775	599	in gene	6.00
NM_146194	Picalm	phosphatidylinositol binding clathrin assembly protein	7	97,278,742	97,357,442	-566	upstream	6.00
XR_035233, XR_035450	4632427E13Rik	RIKEN cDNA 4632427E13 gene	7	99,888,634	99,889,977	457	in gene	5.00
NM_029494	Rab30	RAB30, member RAS oncogene family	7	99,890,224	99,985,627	-704	upstream	5.00
NM_001080995	4632434I11Rik	RIKEN cDNA 4632434I11 gene	7	100,006,036	100,022,742	-1210	upstream	5.00
NM_028243	Prcp	prolylcarboxypeptidase (angiotensinase C)	7	100,023,807	100,082,509	145	in gene	5.00
NM_011858	Odz4	odd Oz/ten-m homolog 4 (Drosophila)	7	103,359,147	104,057,064	97157	in gene	5.00
NM_010248	Gab2	growth factor receptor bound protein 2-associated protein 2	7	104,230,261	104,453,546	1163	in gene	9.00
NM_001010826, NM_001012434	Kctd14	potassium channel tetramerisation domain containing 14	7	104,601,714	104,607,003	-6498	upstream	6.00
NM_001081267	Rsf1	remodeling and spacing factor 1	7	104,728,406	104,835,302	102570	in gene	6.00
NM_007602	Capn5	calpain 5	7	105,270,075	105,326,703	6799	in gene	6.00

Accession No.	Symbol	Gene Title	Chr	Gene Start	Gene End	Interval Dists to Start	Interval Pos	Avg Peak
XR_035196, XR_035257	A630091E08Rik	RIKEN cDNA A630091E08 gene	7	105,691,830	105,711,500	-9620	upstream	8.00
NM_009519	Wnt11	wingless-related MMTV integration site 11	7	105,987,355	106,002,258	12581	in gene	12.00
NM_001043355, NM_001048167, NM_010837	Mtap6	microtubule-associated protein 6	7	106,415,957	106,485,647	23659	in gene	5.00
NM_009825	Serpinh1	serine (or cysteine) peptidase inhibitor, clade H, member 1	7	106,493,886	106,501,606	-346	upstream	8.00
NM_133692	Pold3	polymerase (DNA-directed), delta 3, accessory subunit	7	107,230,623	107,270,010	-38	upstream	10.00
NM_001081116	Arhgef17	Rho guanine nucleotide exchange factor (GEF) 17	7	108,018,260	108,080,675	55915, 17635	in gene, in gene	5.00
NM_199012	Fchsd2	FCH and double SH3 domains 2	7	108,257,327	108,432,919	-639	upstream	6.00
NM_001040111, NM_001040112, NM_027180, NM_198096	Centd2	centaurin, delta 2	7	108,496,583	108,561,100	13892, 71561	in gene, downstream	4.50
	Mirn139	microRNA 139	7	108,623,889	108,623,956	-8673	upstream	7.00
NM_010567	Inppl1	inositol polyphosphate phosphatase-like 1	7	108,971,175	108,986,590	718	in gene	6.00
NM_008035	Folr2	folate receptor 2 (fetal)	7	108,988,502	109,012,042	26170, -5974	downstream, upstream	5.50
NM_008034	Folr1	folate receptor 1 (adult)	7	109,006,845	109,019,197	1181	in gene	5.00
NM_022979	Nup98	nucleoporin 98	7	109,282,717	109,358,634	234	in gene	6.00
NM_018880	Trim3	tripartite motif protein 3	7	112,758,984	112,782,013	-51	upstream	5.00
NM_029802	Arfip2	ADP-ribosylation factor interacting protein 2	7	112,784,135	112,788,861	6797	downstream	5.00
NM_019502	Fxc1	fractured callus expressed transcript 1	7	112,789,054	112,790,360	-6990	upstream	5.00
NM_021889	Syt9	synaptotagmin IX	7	114,514,304	114,692,169	16	in gene	6.00
NM_008905	Ppfibp2	protein tyrosine phosphatase, receptor-type, F interacting protein, binding protein 2	7	114,738,780	114,888,399	79876	in gene	5.00
NM_011975	Rpl27a	ribosomal protein L27a	7	116,662,709	116,665,883	395	in gene	6.00
NM_001001326, NM_029811	St5	suppression of tumorigenicity 5	7	116,667,425	116,760,661	97557	downstream	6.00
NM_053247	Xlkd1	extra cellular link domain-containing 1	7	117,994,121	118,006,467	-5261	upstream	6.00
NM_194464	Mrvi1	MRV integration site 1	7	118,011,781	118,125,609	113881	downstream	6.00
XM_001479276	LOC100043221	hypothetical protein LOC100043221	7	118,226,578	118,266,051	-109	upstream	7.00
XM_001478600	LOC100042658	similar to Ubtf protein	7	118,266,282	118,386,337	120177, 86209	downstream, in gene	6.00
NM_173739	Galntl4	UDP-N-acetyl-alpha-D-galactosamine:polypeptide N-acetylgalactosaminyltransferase-like 4	7	118,615,175	118,923,491	112867	in gene	6.00
NM_015814	Dkk3	dickkopf homolog 3 (Xenopus laevis)	7	119,259,533	119,302,571	9963	in gene	5.00
NM_009346	Tead1	TEA domain family member 1	7	119,823,107	120,043,504	146877	in gene	10.00
XM_001479452	LOC100043295	similar to ribosomal protein L5	7	121,848,312	121,849,763	-7325	upstream	7.00
NM_054084	Calcb	calcitonin-related polypeptide, beta	7	121,862,151	121,866,737	-5063	upstream	7.00
NM_175645	Xylt1	xylosyltransferase 1	7	124,524,493	124,811,144	20659, 124595	in gene, in gene	6.00
NM_170669	Rps15a	ribosomal protein S15a	7	125,247,888	125,259,661	-24	upstream	4.00
NM_019419	Arl6ip1	ADP-ribosylation factor-like 6 interacting protein 1	7	125,262,414	125,273,077	13392	downstream	4.00
NM_138649	Syt17	synaptotagmin XVII	7	125,525,370	125,587,066	-7670	upstream	6.00
XM_001478924	LOC100042845	hypothetical protein LOC100042845	7	125,589,777	125,591,866	4959	downstream	6.00
NM_022420	Gprc5b	G protein-coupled receptor, family C, group 5, member B	7	126,115,561	126,138,669	-611	upstream	5.00
NM_001024138	Gpr139	G protein-coupled receptor 139	7	126,287,837	126,327,888	38464	in gene	5.00
NM_173408	Dcun1d3	DCN1, defective in cullin neddylation 1, domain containing 3 (S. cerevisiae)	7	126,999,327	127,038,917	69, -699	in gene, upstream	5.50
XM_001479781	LOC100043396	hypothetical protein LOC100043396	7	127,037,858	127,039,812	964, 196	in gene, in gene	5.50
NM_029610	Lyrm1	LYR motif containing 1	7	127,039,806	127,060,264	-958, -190	upstream, upstream	5.50
NM_025298	Polr3e	polymerase (RNA) III (DNA directed) polypeptide E	7	128,061,331	128,089,763	4893	in gene	6.00
XM_001478471, XM_001479210	4930413G21Rik	RIKEN cDNA 4930413G21 gene	7	130,112,572	130,113,564	332	in gene	8.00
NM_175023	Rbbp6	retinoblastoma binding protein 6	7	130,114,333	130,123,695	-1101	upstream	8.00
NM_144529	Arhgap17	Rho GTPase activating protein 17	7	130,422,785	130,513,396	75052	in gene	4.00
NM_025304	Lcmt1	leucine carboxyl methyltransferase 1	7	130,521,496	130,573,872	39000	in gene	6.00
NM_001081022	D430042O09Rik	RIKEN cDNA D430042O09 gene	7	132,851,390	133,018,311	19426, 76130	in gene, in gene	5.50
NM_029420	Giyd2	GIY-YIG domain containing 2	7	133,834,650	133,839,297	353	in gene	7.00
NM_175103	Bola2	bolA-like 2 (E. coli)	7	133,839,514	133,840,207	-570	upstream	7.00
NM_009898	Coro1a	coronin, actin binding protein 1A	7	133,843,288	133,848,268	9324	downstream	7.00
NM_024228	Gdpd3	glycerophosphodiester phosphodiesterase domain containing 3	7	133,909,928	133,919,159	10552	downstream	7.00
NM_025347, NM_026875	Ypel3	yippee-like 3 (Drosophila)	7	133,920,489	133,924,028	-9	upstream	7.00
NM_011538	Tbx6	T-box 6	7	133,924,997	133,929,062	-4517	upstream	7.00
NM_019674	Ppp4c	protein phosphatase 4, catalytic subunit	7	133,929,382	133,935,985	15505	downstream	7.00
NM_010069	Doc2a	double C2, alpha	7	133,991,067	133,996,219	53	in gene	5.00
NM_153580	Ccdc95	coiled-coil domain containing 95	7	133,995,947	134,004,977	13857	downstream	5.00
NM_008400	Itgal	integrin alpha L	7	134,439,862	134,477,412	10522, 26314	in gene, in gene	5.50
NM_001040684, NM_133943	Hsd3b7	hydroxy-delta-5-steroid dehydrogenase, 3 beta- and steroid delta-isomerase 7	7	134,944,123	134,947,316	37	in gene	8.00
NM_024414	Stx1b2	syntaxin 1B2	7	134,950,358	134,968,045	23885	downstream	8.00
NM_146259	Zfp668	zinc finger protein 668	7	135,008,873	135,020,337	401	in gene	7.00

Accession No.	Symbol	Gene Title	Chr	Gene Start	Gene End	Interval Dists to Start	Interval Pos	Avg Peak
NM_172749	Zfp646	zinc finger protein 646	7	135,021,215	135,029,510	-1279	upstream	7.00
NM_001081268	BC039632	cDNA sequence BC039632	7	135,028,958	135,034,484	14548	downstream	7.00
NM_139149	Fus	fusion, derived from t(12;16) malignant liposarcoma (human)	7	135,110,993	135,125,546	-161	upstream	6.00
XM_001479568	LOC100043133	similar to crooked legs CG14938-PB	7	135,379,715	135,380,933	69	in gene	9.00
NM_146205	Armc5	armadillo repeat containing 5	7	135,380,871	135,388,614	-7	upstream	9.00
NM_009365	Tgfb1i1	transforming growth factor beta 1 induced transcript 1	7	135,390,385	135,397,226	-9521	upstream	9.00
XM_902363, XM_924870	EG628781	predicted gene, EG628781	7	135,458,298	135,484,624	3942, 14982	in gene, in gene	5.50
XM_001003100, XM_915289	EG639030	predicted gene, EG639030	7	135,739,430	135,739,792	4547	downstream	4.00
NM_178641	Inpp5f	inositol polyphosphate-5-phosphatase F	7	135,754,879	135,839,950	27121	in gene	11.00
NM_019564	Htra1	HtrA serine peptidase 1	7	138,079,749	138,129,167	40283	in gene	8.00
NM_029609	2310007H09Rik	RIKEN cDNA 2310007H09 gene	7	139,802,326	139,898,102	9461, 51082	in gene, in gene	5.00
NM_175268, NM_212473	A930008G19Rik	RIKEN cDNA A930008G19 gene	7	139,903,767	140,004,879	32783, 18431	in gene, in gene	6.00
NM_009980	Ctbp2	C-terminal binding protein 2	7	140,178,694	140,315,166	69294	in gene	7.00
XM_001479821	LOC100043248	similar to chromosome 10 open reading frame 90	7	141,616,800	141,690,746	56122	in gene	7.00
NM_001033420	Dock1	dedicator of cyto-kinesis 1	7	141,862,370	142,365,330	267118, 426334, 441246	in gene, in gene, in gene	5.67
XM_001004753	EG668693	predicted gene, EG668693	7	142,127,566	142,129,961	1922	in gene	6.00
XM_133909, XM_925223	9430038I01Rik	RIKEN cDNA 9430038I01 gene	7	144,527,469	144,602,439	-590	upstream	4.00
NM_023140	Txnl2	thioredoxin-like 2	7	144,629,331	144,660,277	-7667	upstream	4.00
NM_009760	Bnip3	BCL2/adenovirus E1B interacting protein 1, NIP3	7	146,082,519	146,101,189	-139, -9835	upstream, upstream	6.00
XM_001472165, XM_919084	9330101J02Rik	RIKEN cDNA 9330101J02 gene	7	146,814,255	146,869,716	-588	upstream	6.00
NM_177261	Kndc1	kinase non-catalytic C-lobe domain (KIND) containing 1	7	147,080,737	147,127,438	20687	in gene	5.00
XM_887149, XM_916730	Zfp511	zinc finger protein 511	7	147,222,306	147,226,504	-2	upstream	6.00
NM_010836	Msx3	homeo box, msh-like 3	7	147,232,161	147,234,985	12681	downstream	6.00
NM_026820	Ifitm1	interferon induced transmembrane protein 1	7	148,141,967	148,155,717	15121	downstream	6.00
NM_177897	B4galnt4	beta-1,4-N-acetyl-galactosaminyl transferase 4	7	148,247,173	148,258,018	3579	in gene	6.00
NM_027797	Tmem80	transmembrane protein 80	7	148,514,029	148,522,710	10691, 11891	downstream, downstream	5.00
NM_133191	Eps8l2	EPS8-like 2	7	148,524,901	148,548,915	-181, 1019	upstream, in gene	5.00
NM_011528	Taldo1	transaldolase 1	7	148,578,110	148,588,867	1826, 10306	in gene, in gene	4.50
XM_001480697, XM_001480699	LOC100043585	similar to Ran-interacting protein MOG1	7	148,592,410	148,593,254	-3994	upstream	4.00
NM_172116	Pddc1	Parkinson disease 7 domain containing 1	7	148,594,083	148,599,996	11580	downstream	4.00
NM_021316	Cend1	cell cycle exit and neuronal differentiation 1	7	148,612,348	148,615,319	-8393	upstream	6.00
NM_026646	Slc25a22	solute carrier family 25 (mitochondrial carrier, glutamate), member 22	7	148,615,651	148,623,517	-195	upstream	6.00
NM_022654	Lrdd	leucine-rich and death domain containing	7	148,624,414	148,629,254	5542	downstream	6.00
NM_026020	Rplp2	ribosomal protein, large P2	7	148,633,555	148,637,484	-9843	upstream	6.00
NM_001025103	Efcab4a	EF-hand calcium binding domain 4A	7	148,647,042	148,652,512	15518	downstream	7.00
NM_009842	Cd151	CD151 antigen	7	148,653,292	148,657,372	9268	downstream	7.00
XM_888022, XM_916953	Polr2l	polymerase (RNA) II (DNA directed) polypeptide L	7	148,657,758	148,666,947	4387	in gene	7.00
NM_053082	Tspan4	tetraspanin 4	7	148,661,139	148,679,321	1421	in gene	7.00
NM_007459	Ap2a2	adaptor protein complex AP-2, alpha 2 subunit	7	148,748,137	148,818,910	-41	upstream	5.00
NM_008748	Dusp8	dual specificity phosphatase 8	7	149,267,401	149,276,175	-7740	upstream	7.00
NR_001592	H19	H19 fetal liver mRNA	7	149,761,437	149,764,051	-3789	upstream	5.00
	Mirn675	microRNA 675	7	149,762,968	149,763,051	-4789	upstream	5.00
NM_020286	Tspan32	tetraspanin 32	7	150,191,596	150,205,549	19796	downstream	5.00
NM_133790	R74862	expressed sequence R74862	7	150,219,494	150,239,001	27609	downstream	5.00
NM_013742	Cars	cysteinyl-tRNA synthetase	7	150,743,133	150,785,961	13433	in gene	4.00
NM_203492	Mrgprg	MAS-related GPR, member G	7	150,949,615	150,952,898	9794	downstream	5.00
NM_007803	Cttn	cortactin	7	151,621,629	151,656,646	-5258	upstream	5.00
XM_133979, XM_918243	Ppfia1	protein tyrosine phosphatase, receptor type, f polypeptide (PTPRF), interacting protein, alpha 1	7	151,662,670	151,739,634	77730	downstream	5.00
NM_145379	Mrgprf	MAS-related GPR, member F	7	152,486,814	152,495,462	1943	in gene	5.00
NM_080461	Zfp358	zinc finger protein 358	8	3,493,138	3,497,208	8750	downstream	7.00
NM_053177	Mcoln1	mucolipin 1	8	3,500,519	3,515,232	1369	in gene	7.00
NM_173446	Tmem28	transmembrane protein 28	8	9,206,010	9,771,023	569631	downstream	6.00
NM_176953	Lig4	ligase IV, DNA, ATP-dependent	8	9,970,020	9,976,323	-1485	upstream	8.00
NM_001081119	Abhd13	abhydrolase domain containing 13	8	9,977,717	9,992,155	91	in gene	8.00
NM_001081397	Myo16	myosin XVI	8	10,153,923	10,633,950	203485, 460797	in gene, in gene	6.00
NM_009931	Col4a1	procollagen, type IV, alpha 1	8	11,198,431	11,312,730	109018	in gene	5.00
XM_001474115, XM_906739	Cars2	cysteinyl-tRNA synthetase 2 (mitochondrial)(putative)	8	11,513,975	11,555,691	-2480	upstream	5.00
NM_011919	Ing1	inhibitor of growth family, member 1	8	11,556,066	11,563,250	2105	in gene	5.00

Accession No.	Symbol	Gene Title	Chr	Gene Start	Gene End	Interval Dists to Start	Interval Pos	Avg Peak
NM_017402	Arhgef7	Rho guanine nucleotide exchange factor (GEF7)	8	11,758,347	11,835,219	469	in gene	6.00
NM_001013812	EG434280	predicted gene, EG434280	8	12,385,771	12,436,732	11018	in gene	4.00
XM_489223, XM_920462	EG434279	predicted gene, EG434279	8	12,390,168	12,391,639	-5150	upstream	4.00
NM_009233	Sox1	SRY-box containing gene 1	8	12,396,353	12,397,562	436	in gene	4.00
NM_015804	Atp11a	ATPase, class VI, type 11A	8	12,757,224	12,865,173	15848	in gene	5.00
NM_178076	Mcf2l	mcf.2 transforming sequence-like	8	12,915,958	13,020,502	39402	in gene	6.00
NM_001024504, NM_001042649, NM_001042650, NM_001042651	Dcun1d2	DCN1, defective in cullin neddylation 1, domain containing 2 (S. cerevisiae)	8	13,255,963	13,288,126	926	in gene	7.00
NM_172282	Tmco3	transmembrane and coiled-coil domains 3	8	13,288,013	13,322,924	-813	upstream	7.00
NM_011881	Grk1	G protein-coupled receptor kinase 1	8	13,405,081	13,417,580	4455	in gene	5.00
NM_001037736, NM_172751	Arhgef10	Rho guanine nucleotide exchange factor (GEF) 10	8	14,911,717	15,001,085	29691	in gene	5.00
NM_026792	Agpat5	1-acylglycerol-3-phosphate O-acyltransferase 5 (lysophosphatidic acid acyltransferase, epsilon)	8	18,846,279	18,884,413	6473	in gene	5.00
XM_001476645	LOC100041567	similar to p47 protein	8	19,783,773	19,792,069	883	in gene	9.00
NM_008872	Plat	plasminogen activator, tissue	8	23,868,240	23,893,316	8144	in gene	8.00
NM_153397	Adam32	a disintegrin and metallopeptidase domain 32	8	25,946,616	26,059,259	2011	in gene	5.00
NM_001079908, NM_001079909, NM_010206	Fgfr1	fibroblast growth factor receptor 1	8	26,629,244	26,686,186	10132, 18596	in gene, in gene	6.00
NM_001080793, NM_011791	Ash2l	ash2 (absent, small, or homeotic)-like (Drosophila)	8	26,927,342	26,958,142	18862, 6782	in gene, in gene	5.50
NM_008432	Kcnu1	potassium channel, subfamily U, member 1	8	26,960,095	27,048,406	-8735	upstream	6.00
NM_207659	Hook3	hook homolog 3 (Drosophila)	8	27,142,362	27,229,486	-610	upstream	5.00
NM_029965	Rnf170	ring finger protein 170	8	27,229,852	27,254,343	244	in gene	5.00
NM_026067	Thex1	three prime histone mRNA exonuclease 1	8	36,528,307	36,558,535	22140	in gene	6.00
XM_001476601	LOC100041572	hypothetical protein LOC100041572	8	36,762,110	36,830,423	57069	in gene	5.00
NM_001040699	Mtmr7	myotubularin related protein 7	8	41,635,513	41,720,146	-4483	upstream	6.00
NM_023662	Pcm1	pericentriolar material 1	8	42,325,127	42,417,698	169	in gene	6.00
XM_001473026	LOC100039508	similar to cyclic nucleotide gated channel beta 1	8	45,806,143	46,020,493	215153	downstream	6.00
NM_001081286	Fat1	FAT tumor suppressor homolog 1 (Drosophila)	8	46,035,568	46,137,611	48656, 100752, 110752	in gene, in gene, downstream	6.00
NM_008639	Mtnr1a	melatonin receptor 1A	8	46,154,564	46,173,860	-8244	upstream	7.00
NM_172752	Sorbs2	sorbin and SH3 domain containing 2	8	46,593,149	46,913,258	138211	in gene	6.00
NM_027973	Mlf1ip	myeloid leukemia factor 1 interacting protein	8	47,637,423	47,664,938	-1807	upstream	6.00
XM_001474568, XM_358744	4930448N21Rik	RIKEN cDNA 4930448N21 gene	8	48,379,626	48,531,716	157161	downstream	4.00
NM_133791	Wwc2	WW, C2 and coiled-coil domain containing 2	8	48,912,949	49,075,905	123393	in gene	5.00
NM_011857	Odz3	odd Oz/ten-m homolog 3 (Drosophila)	8	49,313,038	49,760,044	431436	in gene	6.00
NM_144731	Galnt7	UDP-N-acetyl-alpha-D-galactosamine: polypeptide N-acetylgalactosaminyltransferase 7	8	60,003,366	60,131,800	6834	in gene	4.00
NM_021506	Sh3rf1	SH3 domain containing ring finger 1	8	63,702,968	63,874,869	6008, 23272, 166952	in gene, in gene, in gene	5.67
NM_001045553, NM_172754	AI449175	expressed sequence AI449175	8	72,134,553	72,149,447	471	in gene	5.00
NM_133224	Atp13a1	ATPase type 13A1	8	72,315,133	72,331,644	14648	in gene	6.00
NM_198101	Gmip	Gem-interacting protein	8	72,332,586	72,345,769	-2805	upstream	6.00
NM_145597	Tmem161a	transmembrane protein 161A	8	72,696,308	72,707,562	19548	downstream	6.00
NM_001007570	Slc25a42	solute carrier family 25, member 42	8	72,708,239	72,736,155	20299, -101	in gene, upstream	6.00
NM_016685	Comp	cartilage oligomeric matrix protein	8	72,897,457	72,905,965	5455	in gene	6.00
NM_001004062	Crtc1	CREB regulated transcription coactivator 1	8	72,906,257	72,963,472	60560, 18912	downstream, in gene	5.00
NM_018827	Crlf1	cytokine receptor-like factor 1	8	73,017,055	73,027,980	13569	downstream	9.00
NM_025577	2810428I15Rik	RIKEN cDNA 2810428I15 gene	8	73,028,195	73,030,638	14	in gene	9.00
NM_019883	Uba52	ubiquitin A-52 residue ribosomal protein fusion product 1	8	73,032,165	73,034,266	3642	downstream	9.00
XM_001477857	LOC100042011	hypothetical protein LOC100042011	8	73,387,026	73,389,327	-8401, -9201	upstream, upstream	5.50
NM_026964	Ccdc124	coiled-coil domain containing 124	8	73,392,126	73,397,389	-339, -1139	upstream, upstream	5.50
NM_053248	Slc5a5	solute carrier family 5 (sodium iodide symporter), member 5	8	73,407,401	73,416,623	18895, 18095	downstream, downstream	5.50
NM_015742	Myo9b	myosin IXb	8	73,796,613	73,884,611	87051	in gene	5.00
NM_025917, NM_029768	2010315L10Rik	RIKEN cDNA 2010315L10 gene	8	73,891,125	73,893,628	-7461	upstream	5.00
NM_022419	Abhd8	abhydrolase domain containing 8	8	73,980,604	73,987,545	-1191	upstream	6.00
NM_053162	Mrpl34	mitochondrial ribosomal protein L34	8	73,988,825	73,989,652	-89	upstream	6.00
XM_001476732, XM_912953	1500034J01Rik	RIKEN cDNA 1500034J01 gene	8	73,993,086	74,000,326	-4350	upstream	6.00
XM_889480	Tmem16h	transmembrane protein 16H	8	73,995,493	74,009,966	21230	downstream	6.00
XM_001478555	LOC100042405	similar to high mobility group nucleosomal binding domain 2	8	74,827,959	74,829,212	-1796	upstream	5.00
NM_010687	Large	like-glycosyltransferase	8	75,338,498	75,876,455	-1065	upstream	7.00
XM_001472068	LOC100039001	hypothetical protein LOC100039001	8	75,875,064	75,877,621	101	in gene	7.00
NM_010442	Hmox1	heme oxygenase (decycling) 1	8	77,617,521	77,624,484	-113	upstream	4.00

Accession No.	Symbol	Gene Title	Chr	Gene Start	Gene End	Interval Dists to Start	Interval Pos	Avg Peak
NM_008566	Mcm5	minichromosome maintenance deficient 5, cell division cycle 46 (S. cerevisiae)	8	77,633,427	77,652,338	15181	in gene	5.00
XM_001472461	LOC100039211	hypothetical protein LOC100039211	8	79,273,850	79,426,092	-84	upstream	7.00
NM_001083906	Nr3c2	nuclear receptor subfamily 3, group C, member 2	8	79,426,407	79,767,538	-231	upstream	7.00
NM_030113	Arhgap10	Rho GTPase activating protein 10	8	79,774,265	80,041,806	73566, 73006, 4782	in gene, in gene, in gene	5.67
XM_001472504	LOC100039235	hypothetical protein LOC100039235	8	80,041,781	80,047,934	-4757	upstream	6.00
NM_009033	Rbmxrt	RNA binding motif protein, X chromosome retrogene	8	81,029,168	81,032,827	-37	upstream	7.00
NM_029736	Slc10a7	solute carrier family 10 (sodium/bile acid cotransporter family), member 7	8	81,033,234	81,257,904	-370, 225054	upstream, downstream	7.00
XM_906331, XM_984073	Frem3	Fras1 related extracellular matrix protein 3	8	83,134,938	83,219,456	79670	in gene	6.00
NM_053124	Smarca5	SWI/SNF related, matrix associated, actin dependent regulator of chromatin, subfamily a, member 5	8	83,223,842	83,263,358	48750	downstream	6.00
NM_177262	Pkn1	protein kinase N1	8	86,193,995	86,223,025	-15	upstream	5.00
NM_181039	Lphn1	latrophilin 1	8	86,424,612	86,463,876	-580	upstream	6.00
	Mirn23a	microRNA 23a	8	86,732,416	86,732,490	-6736	upstream	9.00
	Mirn27a	microRNA 27a	8	86,732,570	86,732,656	-6890	upstream	9.00
	Mirn24-2	microRNA 24-2	8	86,732,713	86,732,819	-7033	upstream	9.00
NM_172503	Zswim4	zinc finger, SWIM domain containing 4	8	86,734,841	86,760,941	35261	downstream	9.00
NM_010499	Ier2	immediate early response 2	8	87,183,450	87,186,747	-6181	upstream	7.00
NM_025648	Farsa	phenylalanyl-tRNA synthetase, alpha subunit	8	87,380,923	87,390,925	165	in gene	7.00
XM_894155, XM_988455	BC004022	cDNA sequence BC004022	8	89,365,037	89,427,214	10862	in gene	6.00
XM_001474508	LOC100040328	hypothetical protein LOC100040328	8	90,568,802	90,589,523	-6253	upstream	5.00
NM_012047	Brd7	bromodomain containing 7	8	90,856,210	90,886,055	-7977	upstream	6.00
XM_001474559	LOC100040369	similar to retinitis pigmentosa GTPase regulator interacting protein 1	8	90,900,368	90,959,165	-6336, 52432	upstream, in gene	5.50
NM_027280	Nkd1	naked cuticle 1 homolog (Drosophila)	8	91,045,269	91,116,225	16939, 25179, 36747, 57899	in gene, in gene, in gene, in gene	8.00
NM_021390	Sall1	sal-like 1 (Drosophila)	8	91,552,142	91,566,338	-318	upstream	6.00
XM_001476646	LOC100041089	hypothetical protein LOC100041089	8	91,562,636	91,565,980	4020	downstream	6.00
NM_177224	Chd9	chromodomain helicase DNA binding protein [illegible]	8	93,352,736	93,578,407	79552, 80128	in gene, in gene	5.00
XM_890814, XM_909999	EG626231	predicted gene, EG626231	8	93,430,430	93,432,614	326, -250	in gene, upstream	5.00
NM_011936	AJ237917	cDNA sequence AJ237917	8	93,837,438	94,192,329	82, 193218	in gene, in gene	6.50
NM_008393	Irx3	Iroquois related homeobox 3 (Drosophila)	8	94,322,424	94,325,273	-1639	upstream	6.00
NM_177767	Ogfod1	2-oxoglutarate and iron-dependent oxygenase domain containing 1	8	96,561,191	96,591,822	21801	in gene	6.00
NM_008631	Mt4	metallothionein 4	8	96,661,104	96,662,931	-9280	upstream	5.00
NM_022331	Herpud1	homocysteine-inducible, endoplasmic reticulum stress-inducible, ubiquitin-like domain member 1	8	96,910,400	96,919,258	-6688, 224	upstream, in gene	8.50
NM_001033207	AI451557	expressed sequence AI451557	8	97,048,967	97,051,164	7801	downstream	5.00
NM_153507	Cpne2	copine II	8	97,056,928	97,094,429	-160	upstream	5.00
NM_026385	Pllp	plasma membrane proteolipid	8	97,198,795	97,220,142	782	in gene	6.00
NM_024467	Zfp319	zinc finger protein 319	8	97,850,036	97,855,850	-246	upstream	6.00
NM_008187	Gtl3	gene trap locus 3	8	97,944,152	97,958,692	52	in gene	7.00
NM_009974	Csnk2a2	casein kinase 2, alpha prime polypeptide	8	97,971,872	98,012,690	-302	upstream	6.00
XM_001477792, XM_001477935	4933406B17Rik	RIKEN cDNA 4933406B17 gene	8	98,020,347	98,024,848	-7355	upstream	6.00
NM_009868	Cdh5	cadherin 5	8	106,625,599	106,668,401	11969	in gene	6.00
NM_001013380	Dync1li2	dynein, cytoplasmic 1 light intermediate chain 2	8	106,943,024	106,966,905	13604, 5193	in gene, in gene	5.00
NM_180958	Ccdc79	coiled-coil domain containing 79	8	106,970,619	107,033,787	72075	downstream	5.00
NM_001024606	4833426J09Rik	RIKEN cDNA 4833426J09 gene	8	107,115,368	107,120,749	-8360, -125	upstream, upstream	6.00
NM_007663	Cdh16	cadherin 16	8	107,138,047	107,148,161	12833, -6431	downstream, upstream	5.50
NM_019662	Rrad	Ras-related associated with diabetes	8	107,151,966	107,155,221	629	in gene	6.00
NM_026753	1110019N10Rik	RIKEN cDNA 1110019N10 gene	8	107,163,739	107,165,628	11036	downstream	6.00
NM_022309	Cbfb	core binding factor beta	8	107,694,574	107,741,886	54642	downstream	6.00
NM_145604	D230025D16Rik	RIKEN cDNA D230025D16 gene	8	107,749,205	107,776,953	11	in gene	6.00
NM_178879	C76566	expressed sequence C76566	8	107,776,538	107,779,051	-8309	upstream	6.00
NM_001033161	Tradd	TNFRSF1A-associated via death domain	8	107,782,475	107,788,494	1134	in gene	6.00
NM_015821	Fbxl8	F-box and leucine-rich repeat protein 8	8	107,788,640	107,793,225	-1280	upstream	6.00
NM_011939	Hsf4	heat shock transcription factor 4	8	107,793,774	107,799,745	-6414	upstream	6.00
NM_177699	Fhod1	formin homology 2 domain containing 1	8	107,853,065	107,871,831	-217	upstream	5.00
NM_001081332	Slc9a5	solute carrier family 9 (sodium/hydrogen exchanger), member 5	8	107,872,158	107,893,781	-110	upstream	5.00
NM_177150	Cenpt	centromere protein T	8	108,368,578	108,375,908	-3900	upstream	7.00
XM_001476693	LOC100041617	hypothetical protein LOC100041617	8	108,375,935	108,378,950	-858	upstream	7.00
NM_021513	Thap11	THAP domain containing 11	8	108,379,043	108,380,850	765	in gene	7.00
NM_026532	Nutf2	nuclear transport factor 2	8	108,384,534	108,404,302	-4726	upstream	7.00
NM_133792	Lypla3	lysophospholipase 3	8	108,674,299	108,688,615	53, 18629	in gene, downstream	5.00
NM_178798	Slc7a6	solute carrier family 7 (cationic amino acid transporter, y+ system), member 6	8	108,692,775	108,722,604	153	in gene	5.00
NM_009864	Cdh1	cadherin 1	8	109,127,268	109,194,146	43372	in gene	5.00

Accession No.	Symbol	Gene Title	Chr	Gene Start	Gene End	Interval Dists to Start	Interval Pos	Avg Peak
NM_173037	Tmco7	transmembrane and coiled-coil domains 7	8	109,206,968	109,375,339	104, 4232	in gene, in gene	7.50
XM_134291	EG234703	predicted gene, EG234703	8	109,451,587	109,452,355	-7293	upstream	7.00
NM_009229	Sntb2	syntrophin, basic 2	8	109,459,650	109,538,092	-2	upstream	7.00
XM_001479484, XM_001479493	LOC100042791	similar to ribosomal protein	8	109,786,616	109,799,353	11449	in gene	7.00
NM_018823, NM_133957	Nfat5	nuclear factor of activated T-cells 5	8	109,817,762	109,903,417	-482	upstream	5.00
NM_010817	Psmd7	proteasome (prosome, macropain) 26S subunit, non-ATPase, 7	8	110,104,281	110,112,382	222	in gene	5.00
XM_001476934	LOC100041744	hypothetical protein LOC100041744	8	110,196,656	110,390,197	12896	in gene	6.00
XM_001479588	LOC100042829	hypothetical protein LOC100042829	8	111,901,750	111,964,490	9370	in gene	4.00
NM_019938	Pmfbp1	polyamine modulated factor 1 binding protein 1	8	112,017,927	112,066,540	49337	downstream	7.00
NM_178380	Dhx38	DEAH (Asp-Glu-Ala-His) box polypeptide 38	8	112,071,924	112,089,501	22237, 5325	downstream, in gene	6.00
NM_175646	Txnl4b	thioredoxin-like 4B	8	112,089,905	112,097,939	-5729	upstream	5.00
NM_028018	2400003C14Rik	RIKEN cDNA 2400003C14 gene	8	112,195,229	112,217,164	-116	upstream	7.00
NM_029468	4930566A11Rik	RIKEN cDNA 4930566A11 gene	8	112,229,988	112,248,832	20908	downstream	8.00
NM_001080930	D8Ertd587e	DNA segment, Chr 8, ERATO Doi 587, expressed	8	112,250,351	112,261,639	10743	in gene	8.00
XM_146511, XM_916673	Phlppl	PH domain and leucine rich repeat protein phosphatase-like	8	112,392,447	112,464,808	625	in gene	4.00
NM_146216	Vac14	Vac14 homolog (S. cerevisiae)	8	113,142,538	113,244,298	69334, 87318	in gene, in gene	6.00
NM_009179, NM_178048	St3gal2	ST3 beta-galactoside alpha-2,3-sialyltransferase 2	8	113,443,822	113,495,457	-398	upstream	6.00
NM_181549	Mrcl	mannose receptor-like precursor	8	113,582,820	113,605,988	436	in gene	6.00
NM_133206	Znrf1	zinc and ring finger 1	8	114,060,742	114,147,678	73610	in gene	5.00
NM_009954	Bcar1	breast cancer anti-estrogen resistance 1	8	114,234,374	114,267,709	15421	in gene	9.00
NM_011801	Cfdp1	craniofacial development protein 1	8	114,292,373	114,378,210	83106	in gene	6.00
NM_173016	AI427515	expressed sequence AI427515	8	116,729,540	116,897,970	5004	in gene	6.00
NM_026844	2310061C15Rik	RIKEN cDNA 2310061C15 gene	8	119,412,585	119,445,336	27816	in gene	6.00
NM_001081151	Gan	giant axonal neuropathy	8	119,682,035	119,729,086	-243	upstream	8.00
XM_001004724, XM_924798	4933407C03Rik	RIKEN cDNA 4933407C03 gene	8	119,780,993	119,985,406	75455, 132447, 177320	in gene, in gene, in gene	6.00
NM_054095	Efcbp2	EF hand calcium binding protein 2	8	121,970,619	121,996,535	14789	in gene	8.00
NM_001009950	Gm587	gene model 587, (NCBI)	8	122,003,502	122,025,598	7134	in gene	4.00
NM_026648	Lrrc50	leucine rich repeat containing 50	8	122,099,135	122,122,354	26369	downstream	7.00
NM_021441	Taf1c	TATA box binding protein (Tbp)-associated factor, RNA polymerase I, C	8	122,121,874	122,129,140	3636	in gene	7.00
XM_895061	EG628061	predicted gene, EG628061	8	122,126,000	122,126,551	1047	downstream	7.00
NM_023395	Wfdc1	WAP four-disulfide core domain 1	8	122,190,265	122,211,920	31063	downstream	6.00
XM_486167, XM_917337	Atp2c2	ATPase, Ca++ transporting, type 2C, member 2	8	122,223,909	122,281,618	-2581	upstream	6.00
NM_028071	Cotl1	coactosin-like 1 (Dictyostelium)	8	122,333,125	122,364,440	41448	downstream	7.00
NM_009462	Usp10	ubiquitin specific peptidase 10	8	122,434,752	122,481,457	-8720	upstream	6.00
NM_198671	Gse1	genetic suppressor element 1	8	123,012,766	123,105,277	64626	in gene	5.00
XM_001477969	LOC100042333	similar to Ubtf protein	8	123,563,570	123,589,605	-8939	upstream	5.00
NM_172761	Mthfsd	methenyltetrahydrofolate synthetase domain containing	8	123,621,840	123,632,218	-9894	upstream	8.00
NM_013519	Foxc2	forkhead box C2	8	123,640,483	123,642,795	1629	in gene	8.00
NM_008024	Foxl1	forkhead box L1	8	123,651,585	123,654,544	-9473	upstream	8.00
NM_080855	Zcchc14	zinc finger, CCHC domain containing 14	8	124,123,368	124,175,833	14329	in gene	6.00
NM_145605	Klhdc4	kelch domain containing 4	8	124,320,230	124,353,469	23261, -67	in gene, upstream	4.50
NM_011404	Slc7a5	solute carrier family 7 (cationic amino acid transporter, y+ system), member 5	8	124,405,050	124,431,571	34963, 32467	downstream, downstream	8.00
XM_001001798, XM_111398	Gm22	gene model 22, (NCBI)	8	124,783,967	124,796,550	209	in gene	5.00
NM_026014	Cdt1	chromatin licensing and DNA replication factor 1	8	125,091,915	125,097,030	3989	in gene	7.00
NM_009698	Aprt	adenine phosphoribosyl transferase	8	125,098,537	125,100,807	4903	downstream	7.00
NM_016722	Galns	galactosamine (N-acetyl)-6-sulfate sulfatase	8	125,102,141	125,135,241	39337	downstream	7.00
NM_054046	Def8	differentially expressed in FDCP 8	8	125,966,886	125,987,799	-390	upstream	7.00
NM_009606	Acta1	actin, alpha 1, skeletal muscle	8	126,415,667	126,418,636	2348	in gene	5.00
NM_172288	Nup133	nucleoporin 133	8	126,423,200	126,482,325	66037	downstream	5.00
NM_133966	Taf5l	TAF5-like RNA polymerase II, p300/CBP-associated factor (PCAF)-associated factor	8	126,520,210	126,545,209	-71	upstream	7.00
NM_001029876	AK122209	cDNA sequence AK122209	8	126,547,373	126,572,404	-2093	upstream	7.00
NM_139272	Galnt2	UDP-N-acetyl-alpha-D-galactosamine:polypeptide N-acetylgalactosaminyltransferase 2	8	126,755,294	126,869,622	19570	in gene	6.00
NM_171824	Pgbd5	piggyBac transposable element derived 5	8	126,892,949	126,957,836	63500	in gene	5.00
NM_145607	Ttc13	tetratricopeptide repeat domain 13	8	127,195,227	127,245,875	-2205	upstream	5.00
NM_026855	Arv1	ARV1 homolog (yeast)	8	127,246,039	127,258,023	2041	in gene	5.00
NM_001081120	2310031A18Rik	RIKEN cDNA 2310031A18 gene	8	127,264,157	127,275,709	-1955	upstream	6.00
XM_001480316, XM_001480318, XM_001480431, XM_001480436, XM_001480440	Trim67	tripartite motif-containing 67	8	127,317,652	127,353,278	-420	upstream	7.00
NM_008737	Nrp1	neuropilin 1	8	130,883,140	131,027,247	3388	in gene	5.00
NM_007465	Birc2	baculoviral IAP repeat-containing 2	9	7,818,227	7,835,255	-1785	upstream	8.00

Accession No.	Symbol	Gene Title	Chr	Gene Start	Gene End	Interval Dists to Start	Interval Pos	Avg Peak
NM_008829	Pgr	progesterone receptor	9	8,899,833	8,968,611	70567	downstream	8.00
XM_146632, XM_984736	9030420J04Rik	RIKEN cDNA 9030420J04 gene	9	8,994,328	9,239,101	95629	in gene	6.00
XM_001473154, XM_001481278	Maml2	mastermind like 2 (Drosophila)	9	13,415,221	13,513,977	89603	in gene	4.00
NM_028013	Endod1	endonuclease domain containing 1	9	14,158,450	14,185,436	-340	upstream	4.00
NM_010242	Fut4	fucosyltransferase 4	9	14,552,903	14,556,566	86	in gene	6.00
NM_029306	1700012B09Rik	RIKEN cDNA 1700012B09 gene	9	14,562,644	14,575,474	18994	downstream	6.00
XM_001474439	LOC100040375	similar to KIAA1731 protein	9	15,150,527	15,162,232	-8280	upstream	4.00
NM_001002846, NM_013911	Fbxl12	F-box and leucine-rich repeat protein 12	9	20,442,230	20,449,211	-7621	upstream	5.00
NM_025401	Ubl5	ubiquitin-like 5	9	20,447,762	20,451,233	9070	downstream	5.00
NM_023371	Pin1	protein (peptidyl-prolyl cis/trans isomerase) NIMA-interacting 1	9	20,456,574	20,471,028	258, 5842	in gene, in gene	5.50
NM_173777	Olfm2	olfactomedin 2	9	20,472,144	20,532,673	70257	downstream	6.00
NM_018793	Tyk2	tyrosine kinase 2	9	20,908,512	20,935,719	16295	in gene	5.00
NM_016679	Keap1	kelch-like ECH-associated protein 1	9	21,034,511	21,043,257	57, -5127	in gene, upstream	4.50
NM_053190	Edg8	endothelial differentiation, sphingolipid G-protein-coupled receptor, 8	9	21,047,361	21,050,653	7453, 2269	downstream, in gene	4.50
NM_001039520	Dnm2	dynamin 2	9	21,229,389	21,311,568	-45	upstream	8.00
NM_011417	Smarca4	SWI/SNF related, matrix associated, actin dependent regulator of chromatin, subfamily a, member 4	9	21,420,613	21,507,811	59	in gene	6.00
NM_010700	Ldlr	low density lipoprotein receptor	9	21,528,038	21,554,363	-198	upstream	5.00
NM_010487	Elavl3	ELAV (embryonic lethal, abnormal vision, Drosophila)-like 3 (Hu antigen C)	9	21,822,220	21,856,454	-4106	upstream	5.00
NM_177318	Zfp653	zinc finger protein 653	9	21,859,904	21,875,789	15229	in gene	5.00
NM_181316	Bbs9	Bardet-Biedl syndrome 9	9	22,280,159	22,692,724	302129	in gene	5.00
NM_028472	Bmper	BMP-binding endothelial regulator	9	23,027,520	23,289,646	177008	in gene	7.00
NM_026189	2310005P05Rik	RIKEN cDNA 2310005P05 gene	9	25,289,182	25,411,695	45714	in gene	7.00
NM_172290	Hnt	neurotrimin	9	28,803,549	29,770,714	168234, 113034	in gene, in gene	5.50
NM_001080817	Prdm10	PR domain containing 10	9	31,122,692	31,186,128	71420	downstream	4.00
NM_172766	Nfrkb	nuclear factor related to kappa B binding protein	9	31,193,798	31,228,918	314	in gene	4.00
NM_013800	Barx2	BarH-like homeobox 2	9	31,653,865	31,720,658	63618	in gene	6.00
NM_183171	Fez1	fasciculation and elongation protein zeta 1 (zygin I)	9	36,640,394	36,686,507	10678	in gene	7.00
NM_175189	Hepacam	hepatocyte cell adhesion molecule	9	37,175,191	37,194,157	17129	in gene	7.00
NM_133733	9030425E11Rik	RIKEN cDNA 9030425E11 gene	9	40,494,047	40,592,129	45105	in gene	7.00
NM_173038	Tbcel	tubulin folding cofactor E-like	9	42,220,400	42,280,309	13941	in gene	6.00
NM_175481	Grik4	glutamate receptor, ionotropic, kainate 4	9	42,328,517	42,752,454	411550	in gene	4.00
NM_027144	Arhgef12	Rho guanine nucleotide exchange factor (GEF) 12	9	42,771,925	42,913,801	102386	in gene	4.00
XM_001001707, XM_001479244	D630033O11Rik	RIKEN cDNA D630033O11 gene	9	43,067,962	43,088,149	4310	in gene	6.00
NM_023655	Trim29	tripartite motif protein 29	9	43,119,614	43,144,208	17986	in gene	6.00
NM_016808, NM_198091, NM_198092	Usp2	ubiquitin specific peptidase 2	9	43,875,104	43,903,710	-64, 1221, 9936, 16704	upstream, in gene, in gene, in gene	6.00
NM_030256	Bcl9l	B-cell CLL/lymphoma 9-like	9	44,307,219	44,318,506	-19, 5869	upstream, in gene	6.50
NM_007551	Blr1	Burkitt lymphoma receptor 1	9	44,319,870	44,334,504	21416	downstream	6.00
NM_007841, NM_181324	Ddx6	DEAD (Asp-Glu-Ala-Asp) box polypeptide 6	9	44,412,981	44,445,189	-1173	upstream	7.00
NM_153537	Phldb1	pleckstrin homology-like domain, family B, member 1	9	44,494,391	44,543,277	-2131	upstream	8.00
NM_145985	Arcn1	archain 1	9	44,549,651	44,575,880	30472, -56	downstream, upstream	6.50
NM_023831	1500035H01Rik	RIKEN cDNA 1500035H01 gene	9	44,581,092	44,600,797	-5156	upstream	5.00
NM_001014761	Scn2b	sodium channel, voltage-gated, type II, beta	9	44,925,959	44,938,153	18201	downstream	6.00
NM_001013390	Scn4b	sodium channel, type IV, beta	9	44,947,125	44,962,144	-2965	upstream	6.00
NM_007503, NM_052823	Fxyd2	FXYD domain-containing ion transport regulator 2	9	45,211,221	45,218,361	4795	in gene	5.00
NM_001033324	Zbtb16	zinc finger and BTB domain containing 16	9	48,462,402	48,644,050	98290, -2529	in gene, upstream	5.50
XM_196479, XM_924862	Gm684	gene model 684, (NCBI)	9	51,078,363	51,086,470	-3322	upstream	5.00
XM_134800, XM_917087	1810046K07Rik	RIKEN cDNA 1810046K07 gene	9	51,097,789	51,137,022	47230	downstream	5.00
NM_175535	Arhgap20	Rho GTPase activating protein 20	9	51,573,457	51,661,164	7343	in gene	4.00
XM_001481328, XM_284494	Ddx10	DEAD (Asp-Glu-Ala-Asp) box polypeptide 10	9	52,906,559	53,064,488	14696	in gene	5.00
NM_028060	Slc35f2	solute carrier family 35, member F2	9	53,619,345	53,665,963	14703	in gene	5.00
XM_358382, XM_917435	Dmxl2	Dmx-like 2	9	54,212,965	54,349,886	134990	in gene	6.00
NM_172924	C230081A13Rik	RIKEN cDNA C230081A13 gene	9	56,075,516	56,265,845	-32	upstream	9.00
NM_025812	Hmg20a	high mobility group 20A	9	56,266,653	56,344,743	-776	upstream	9.00
NM_028636	Man2c1	mannosidase, alpha, class 2C, member 1	9	56,978,584	56,990,017	9400	in gene	5.00
NM_028347	Neil1	nei endonuclease VIII-like 1 (E. coli)	9	56,991,063	56,994,841	6857	downstream	5.00
NM_007713	Clk3	CDC-like kinase 3	9	57,598,518	57,613,667	-829	upstream	5.00
NM_012043	Islr	immunoglobulin superfamily containing leucine-rich repeat	9	58,004,076	58,006,949	-5899	upstream	6.00

Accession No.	Symbol	Gene Title	Chr	Gene Start	Gene End	Interval Dists to Start	Interval Pos	Avg Peak
NM_001081192	Hcn4	hyperpolarization-activated, cyclic nucleotide-gated K+ 4	9	58,671,319	58,708,762	35854	in gene	5.00
NM_001040426, NM_172444	Thsd4	thrombospondin, type I, domain containing 4	9	59,817,516	60,358,842	471898, 423578, 378474, 276618	in gene, in gene, in gene, in gene	6.50
XM_001475306	LOC100040481	hypothetical protein LOC100040481	9	61,218,604	61,220,000	3444	downstream	6.00
NM_009389	Tle3	transducin-like enhancer of split 3, homolog of Drosophila E(spl)	9	61,221,479	61,264,791	569	in gene	6.00
XM_001473665	LOC100039851	similar to Hmgb1 protein	9	61,320,398	61,321,719	5735	downstream	5.00
NM_009672	Anp32a	acidic (leucine-rich) nuclear phosphoprotein 32 family, member A	9	62,189,235	62,226,609	2221	in gene	6.00
NM_176922	Itga11	integrin, alpha 11	9	62,525,668	62,631,767	66732	in gene	8.00
NM_010193	Fem1b	feminization 1 homolog b (C. elegans)	9	62,639,628	62,659,409	-9919	upstream	6.00
NM_138304	Calml4	calmodulin-like 4	9	62,705,911	62,723,725	-215, 3449	upstream, in gene	5.00
NM_011840	Map2k5	mitogen activated protein kinase kinase 5	9	63,011,577	63,225,659	40987	in gene	6.00
XM_001475908, XM_134960, XM_917939, XM_925370	Iqch	IQ motif containing H	9	63,269,256	63,450,300	176892	in gene	5.00
NM_017382	Rab11a	RAB11a, member RAS oncogene family	9	64,563,107	64,585,563	987, 59	in gene, in gene	6.00
NM_028906	Dpp8	dipeptidylpeptidase 8	9	64,880,265	64,930,458	58295	downstream	4.00
NM_028030	Rbpms2	RNA binding protein with multiple splicing 2	9	65,478,389	65,508,325	18363	in gene	6.00
NM_010019	Dapk2	death-associated kinase 2	9	66,006,072	66,120,049	-88	upstream	6.00
XM_001477062, XM_918072	Herc1	hect (homologous to the E6-AP (UBE3A) carboxyl terminus) domain and RCC1 (CHC1)-like domain (RLD) 1	9	66,198,331	66,358,856	116901	in gene	5.00
NM_024427	Tpm1	tropomyosin 1, alpha	9	66,870,400	66,897,020	5180	in gene	5.00
NM_001081242	Tln2	talin 2	9	67,065,943	67,387,422	-9741	upstream	6.00
XM_001474360	LOC100040253	hypothetical protein LOC100040253	9	67,407,144	67,547,969	150806	downstream	6.00
XM_001004247, XM_001004252, XM_146937	EG244911	predicted gene, EG244911	9	67,678,296	67,679,635	51	in gene	6.00
NM_177184	Vps13c	vacuolar protein sorting 13C (yeast)	9	67,688,203	67,843,441	-8619	upstream	6.00
NM_013646	Rora	RAR-related orphan receptor alpha	9	68,501,609	69,226,838	461735	in gene	6.00
NM_007585	Anxa2	annexin A2	9	69,301,490	69,339,592	4430	in gene	6.00
NM_172772	B230380D07Rik	RIKEN cDNA B230380D07 gene	9	70,446,821	70,504,981	-27	upstream	5.00
XM_001477924	LOC100041622	similar to RIKEN cDNA 1110014J01 gene	9	70,700,355	70,702,290	10888	downstream	6.00
NM_026599	Cgnl1	cingulin-like 1	9	71,474,316	71,619,409	124337, 123089, 95585, 46193	in gene, in gene, in gene, in gene	6.75
NM_008613	Mns1	meiosis-specific nuclear structural protein 1	9	72,266,685	72,306,381	19443	in gene	6.00
NM_175485	Prtg	protogenin homolog (Gallus gallus)	9	72,655,020	72,762,834	308, 75316	in gene, in gene	5.50
NM_010864	Myo5a	myosin Va	9	74,919,013	75,071,495	126107	in gene	6.00
NM_205844	Gfral	GDNF family receptor alpha like	9	76,011,909	76,061,464	40504	in gene	6.00
NM_172528	Lrrc1	leucine rich repeat containing 1	9	77,278,896	77,391,866	-870	upstream	5.00
NM_001081490, NM_023605	Fbxo9	f-box protein 9	9	77,929,306	77,956,860	-4308	upstream	6.00
NM_019987	Ick	intestinal cell kinase	9	77,961,216	78,015,749	-48	upstream	6.00
NM_133718	Tmem30a	transmembrane protein 30A	9	79,616,756	79,641,237	21	in gene	7.00
XM_001476043	LOC100041249	hypothetical protein LOC100041249	9	79,996,241	80,015,835	25263	downstream	5.00
NM_001039546	Myo6	myosin VI	9	80,012,847	80,159,536	8657	in gene	5.00
NM_022986	Irak1bp1	interleukin-1 receptor-associated kinase 1 binding protein 1	9	82,723,606	82,741,293	8010	in gene	6.00
NM_001081216	Phip	pleckstrin homology domain interacting protein	9	82,759,766	82,869,096	26440, 1064	in gene, in gene	5.00
NM_172926	Snx14	sorting nexin 14	9	88,271,613	88,333,767	103	in gene	7.00
NM_019666, NM_019796	Syncrip	synaptotagmin binding, cytoplasmic RNA interacting protein	9	88,344,576	88,377,235	1283	in gene	6.00
NM_007801	Ctsh	cathepsin H	9	89,949,347	89,970,927	28605	downstream	6.00
NM_001039147, NM_024431	Morf4l1	mortality factor 4 like 1	9	89,986,507	90,009,600	31648	downstream	6.00
NM_009576	Zic4	zinc finger protein of the cerebellum 4	9	91,263,810	91,284,186	13182	in gene	6.00
NM_177909	Slc9a9	solute carrier family 9 (sodium/hydrogen exchanger), isoform 9	9	94,570,337	95,130,864	-10089, 199359	upstream, in gene	5.50
NM_018763	Chst2	carbohydrate sulfotransferase 2	9	95,304,920	95,307,141	48	in gene	5.00
NM_011916	Xrn1	5'-3' exoribonuclease 1	9	95,855,179	95,953,453	21	in gene	7.00
NM_007502	Atp1b3	ATPase, Na+/K+ transporting, beta 3 polypeptide	9	96,233,092	96,264,718	-178	upstream	6.00
NM_177770	BC043934	cDNA sequence BC043934	9	96,336,223	96,338,557	29	in gene	8.00
NM_053268	Rasa2	RAS p21 protein activator 2	9	96,439,719	96,531,922	530	in gene	6.00
NM_138756	Slc25a36	solute carrier family 25, member 36	9	96,977,430	97,011,460	228	in gene	7.00
XM_001474396, XM_001475836	3222402P14Rik	RIKEN cDNA 3222402P14 gene	9	101,004,294	101,154,125	-51	upstream	10.00
NM_173447	Ephb1	Eph receptor B1	9	101,824,458	102,256,963	350915	in gene	6.00
NM_001042607, NM_013649	Ryk	receptor-like tyrosine kinase	9	102,737,250	102,810,637	23678	in gene	4.00
NM_033314	Slco2a1	solute carrier organic anion transporter family, member 2a1	9	102,910,819	102,990,179	70125	in gene	6.00
NM_001002896	Bfsp2	beaded filament structural protein 2, phakinin	9	103,327,254	103,382,658	8274	in gene	6.00
NM_172461	Nek11	NIMA (never in mitosis gene a)-related expressed kinase 11	9	105,064,797	105,297,573	43381	in gene	5.00
NM_172927	E330026B02Rik	RIKEN cDNA E330026B02 gene	9	105,676,442	105,711,978	40149	downstream	4.00

Accession No.	Symbol	Gene Title	Chr	Gene Start	Gene End	Interval Dists to Start	Interval Pos	Avg Peak
NM_026763	1110001D15Rik	RIKEN cDNA 1110001D15 gene	9	105,891,785	105,974,567	-5881	upstream	8.00
XM_135109, XM_920961	Wdr82	WD repeat domain containing 82	9	106,073,257	106,095,321	6967	in gene	5.00
	Mirnlet7g	microRNA let7g	9	106,081,170	106,081,257	-946	upstream	5.00
NM_027354	Wdr51a	WD repeat domain 51A	9	106,183,732	106,252,208	72316	downstream	6.00
NM_153459	Dusp7	dual specificity phosphatase 7	9	106,270,963	106,278,052	13165	downstream	5.00
NM_145619	Parp3	poly (ADP-ribose) polymerase family, member 3	9	106,372,684	106,378,982	-431	upstream	5.00
NM_145620	Rrp9	RRP9, small subunit (SSU) processome component, homolog (yeast)	9	106,379,640	106,387,746	-227	upstream	5.00
XM_001475846	LOC100041128	similar to TAF4A RNA polymerase II, TATA box binding protein (TBP)-associated factor	9	106,689,090	106,725,106	17218, 130	in gene, in gene	6.00
NM_020263	Cacna2d2	calcium channel, voltage-dependent, alpha 2/delta subunit 2	9	107,301,943	107,431,389	9225, 126985	in gene, in gene	6.00
NM_019704	Tmem115	transmembrane protein 115	9	107,436,276	107,440,987	-7348	upstream	7.00
NM_018879	Tusc4	tumor suppressor candidate 4	9	107,444,557	107,448,026	12723	downstream	7.00
NM_053253	Zmynd10	zinc finger, MYND domain containing 10	9	107,449,641	107,453,650	7639	downstream	7.00
NM_019713	Rassf1	Ras association (RalGDS/AF-6) domain family 1	9	107,456,987	107,464,591	293, 14133	in gene, downstream	7.00
NM_019742	Tusc2	tumor suppressor candidate 2	9	107,465,586	107,468,439	-8306, 5534	upstream, downstream	7.00
NM_010489	Hyal2	hyaluronoglucosaminidase 2	9	107,471,494	107,475,109	-374	upstream	7.00
NM_008317	Hyal1	hyaluronoglucosaminidase 1	9	107,479,283	107,482,468	-8163, 10605	upstream, downstream	6.00
NM_019750	Nat6	N-acetyltransferase 6	9	107,482,501	107,486,379	7387	downstream	5.00
NM_178020	Hyal3	hyaluronoglucosaminidase 3	9	107,483,638	107,489,690	6250	downstream	5.00
NM_025903	Ifrd2	interferon-related developmental regulator 2	9	107,490,049	107,495,369	-161	upstream	5.00
NM_001081244	BY080835	cDNA sequence BY080835	9	107,495,398	107,499,406	9518	downstream	5.00
NM_011349	Sema3f	sema domain, immunoglobulin domain (Ig), short basic domain, secreted, (semaphorin) 3 F	9	107,583,833	107,612,806	7878, -6378	in gene, upstream	5.00
NM_028944	4933406E20Rik	RIKEN cDNA 4933406E20 gene	9	108,481,157	108,499,931	5115	in gene	7.00
NM_173019	Pfkfb4	6-phosphofructo-2-kinase/fructose-2,6-biphosphatase 4	9	108,893,947	108,934,739	46789	downstream	7.00
NM_025858, NM_026381	Scotin	scotin gene	9	108,941,141	108,960,280	-405	upstream	7.00
XM_001481067, XM_915629	Lba1	lupus brain antigen 1	9	111,214,243	111,298,279	80477	in gene	5.00
NM_009752	Glb1	galactosidase, beta 1	9	114,310,237	114,383,495	30627	in gene	7.00
NM_001042503	Trim71	tripartite motif-containing 71	9	114,420,386	114,473,487	45295	in gene	8.00
NM_153585	Cnot10	CCR4-NOT transcription complex, subunit 10	9	114,494,996	114,549,192	-152	upstream	8.00
NM_148958	Osbpl10	oxysterol binding protein-like 10	9	114,976,397	115,141,341	21235	in gene	7.00
NM_133710	Ctdspl	CTD (carboxy-terminal domain, RNA polymerase II, polypeptide A) small phosphatase-like	9	118,835,571	118,953,116	49453, 84813	in gene, in gene	5.00
XM_486268, XM_916519	Dlec1	deleted in lung and esophageal cancer 1	9	119,011,596	119,056,812	24660	in gene	6.00
NM_021544	Scn5a	sodium channel, voltage-gated, type V, alpha	9	119,392,529	119,488,134	88726	in gene	6.00
NM_028976	Gorasp1	golgi reassembly stacking protein 1	9	119,834,791	119,846,676	-1388	upstream	4.00
NM_028735	Ttc21a	tetratricopeptide repeat domain 21A	9	119,846,724	119,876,911	1340, 19724	in gene, in gene	4.50
NM_001081339	Cmya1	cardiomyopathy associated 1	9	119,923,383	119,928,933	8485	downstream	6.00
NM_144557	Myrip	myosin VIIA and Rab interacting protein	9	120,213,191	120,383,952	84617, 139929	in gene, in gene	5.50
NM_026892	Eif1b	eukaryotic translation initiation factor 1B	9	120,401,759	120,404,445	-175	upstream	6.00
NM_178676	Entpd3	ectonucleoside triphosphate diphosphohydrolase 3	9	120,448,961	120,477,435	34687	downstream	5.00
NM_025974	Rpl14	ribosomal protein L14	9	120,480,634	120,483,770	3014	in gene	5.00
XM_001473897, XM_001477043	5830454E08Rik	RIKEN cDNA 5830454E08 gene	9	120,486,316	120,487,190	-2668	upstream	5.00
NM_007614	Ctnnb1	catenin (cadherin associated protein), beta 1	9	120,842,593	120,869,625	-49, 19679	upstream, in gene	7.00
XM_001479566, XM_001481114	Ulk4	unc-51-like kinase 4 (C. elegans)	9	120,982,789	121,186,592	210688	downstream	6.00
NM_010918	Nktr	natural killer tumor recognition sequence	9	121,628,299	121,665,959	42453	downstream	5.00
NM_026179	Abhd5	abhydrolase domain containing 5	9	122,260,734	122,290,641	2	in gene	6.00
NM_001002267	Tmem158	transmembrane protein 158	9	123,168,175	123,169,907	8275	downstream	6.00
NM_019958	Rgs17	regulator of G-protein signaling 17	10	4,424,183	4,514,266	98249	downstream	4.00
NM_175374	Mtrf1l	mitochondrial translational release factor 1-like	10	4,522,598	4,534,654	-166	upstream	4.00
XM_136914	Gm221	gene model 221, (NCBI)	10	5,785,430	5,836,669	44237	in gene	6.00
NM_026141	Ppil4	peptidylprolyl isomerase (cyclophilin)-like 4	10	7,512,719	7,542,933	-111	upstream	5.00
NM_175155	Sash1	SAM and SH3 domain containing 1	10	8,442,017	8,605,868	100444, 66988, 65036	in gene, in gene, in gene	7.00
NM_025799	Fuca2	fucosidase, alpha-L- 2, plasma	10	13,220,834	13,237,081	13662	in gene	5.00
NM_019961	Pex3	peroxisomal biogenesis factor 3	10	13,243,922	13,272,899	38403	downstream	5.00
NM_001033432	Heca	headcase homolog (Drosophila)	10	17,620,272	17,667,873	17361	in gene	4.00
NM_009397	Tnfaip3	tumor necrosis factor, alpha-induced protein 3	10	18,720,722	18,735,173	117	in gene	8.00
NM_008580	Map3k5	mitogen activated protein kinase kinase kinase 5	10	19,654,278	19,861,529	191578	in gene	5.00
NM_026203	Ahi1	Abelson helper integration site	10	20,672,470	20,800,235	-22	upstream	7.00
XM_001473813	LOC100039936	hypothetical protein LOC100039936	10	21,463,673	21,466,812	-3641	upstream	4.00
NM_011361	Sgk	serum/glucocorticoid regulated kinase	10	21,714,502	21,719,698	-214	upstream	6.00

Accession No.	Symbol	Gene Title	Chr	Gene Start	Gene End	Interval Dists to Start	Interval Pos	Avg Peak
NM_178934	Slc2a12	solute carrier family 2 (facilitated glucose transporter), member 12	10	22,364,937	22,424,091	46764	in gene	5.00
NM_027347	Crsp3	cofactor required for Sp1 transcriptional activation, subunit 3	10	24,589,853	24,633,266	-189	upstream	5.00
NM_008481	Lama2	laminin, alpha 2	10	26,701,091	27,336,748	594348	in gene	5.00
NM_025614	Rwdd1	RWD domain containing 1	10	33,716,361	33,739,422	-2	upstream	6.00
NM_008538	Marcks	myristoylated alanine rich protein kinase C substrate	10	36,853,049	36,858,732	1796	in gene	7.00
XM_001475382	LOC100040846	hypothetical protein LOC100040846	10	36,860,890	36,871,119	-3954	upstream	7.00
NM_145743	Lace1	lactation elevated 1	10	42,032,391	42,198,371	51	in gene	7.00
NM_172416	Ostm1	osteopetrosis associated transmembrane protein 1	10	42,398,723	42,422,261	31725	downstream	5.00
NM_153055	Sec63	SEC63-like (S. cerevisiae)	10	42,481,532	42,549,093	132	in gene	5.00
XM_001476352	LOC100041418	hypothetical protein LOC100041418	10	43,426,704	43,428,098	6333	downstream	4.00
NM_053069	Atg5	autophagy-related 5 (yeast)	10	43,988,164	44,084,097	93444	in gene	4.00
XM_001476478	LOC100041506	hypothetical protein LOC100041506	10	44,139,763	44,151,019	-9253	upstream	4.00
NM_007548	Prdm1	PR domain containing 1, with ZNF domain	10	44,156,983	44,178,554	18282	in gene	4.00
NM_011156	Prep	prolyl endopeptidase	10	44,787,020	44,878,801	15412	in gene	5.00
NM_177306	Rfxdc1	regulatory factor X domain containing 1	10	51,405,324	51,450,235	21108	in gene	6.00
NM_001081428	4930589M24Rik	RIKEN cDNA 4930589M24 gene	10	53,352,951	53,470,715	121499	downstream	8.00
NM_026148	Lims1	LIM and senescent cell antigen-like domains 1	10	57,786,295	57,887,437	96233	in gene	6.00
NM_010100	Edar	ectodysplasin-A receptor	10	58,063,537	58,138,444	3884	in gene	7.00
NM_026937	Ascc1	activating signal cointegrator 1 complex subunit 1	10	59,465,587	59,562,734	75773, 96125	in gene, in gene	5.50
NM_052994	Spock2	sparc/osteonectin, cwcv and kazal-like domains proteoglycan 2	10	59,569,005	59,596,662	-7293	upstream	5.00
NM_016803	Chst3	carbohydrate (chondroitin 6/keratan) sulfotransferase 3	10	59,644,282	59,651,455	383	in gene	5.00
NM_011179	Psap	prosaposin	10	59,740,486	59,765,348	1914	in gene	5.00
NM_023596	Slc29a3	solute carrier family 29 (nucleoside transporters), member 3	10	60,174,820	60,215,530	21578	in gene	5.00
NM_025273	Pcbd1	pterin 4 alpha carbinolamine dehydratase/dimerization cofactor of hepatocyte nuclear factor 1 alpha (TCF1) 1	10	60,552,107	60,557,067	-6347	upstream	6.00
NM_001081127	Adamts14	a disintegrin-like and metallopeptidase (reprolysin type) with thrombospondin type 1 motif, 14	10	60,659,860	60,736,186	22394	in gene	4.00
NM_013753	X99384	cDNA sequence X99384	10	60,782,410	60,818,784	9248, 6944	in gene, in gene	6.00
NM_026438	Ppa1	pyrophosphatase (inorganic) 1	10	61,111,369	61,136,913	11031	in gene	5.00
NM_009314	Tacr2	tachykinin receptor 2	10	61,715,504	61,728,738	8747	in gene	4.00
NM_010438	Hk1	hexokinase 1	10	61,732,610	61,842,631	118380	downstream	4.00
NM_181423	Supv3l1	suppressor of var1, 3-like 1 (S. cerevisiae)	10	61,892,126	61,912,441	3257	in gene	5.00
NM_133672	Vps26a	vacuolar protein sorting 26 homolog A (yeast)	10	61,918,020	61,949,500	40316	downstream	5.00
NM_053183	Ddx50	DEAD (Asp-Glu-Ala-Asp) box polypeptide 50	10	62,078,771	62,113,946	-214	upstream	6.00
XM_125685, XM_905594	Stox1	storkhead box 1	10	62,121,791	62,188,876	74716	downstream	6.00
XM_483911, XM_905765	Mypn	myopalladin	10	62,578,528	62,671,678	49118	in gene	5.00
XM_907708, XM_975833	Jmjd1c	jumonji domain containing 1C	10	66,590,006	66,719,074	-150	upstream	6.00
NM_001081346	Plekhk1	pleckstrin homology domain containing, family K member 1	10	67,442,346	67,506,612	18966	in gene	5.00
NM_001081347	Rhobtb1	Rho-related BTB domain containing 1	10	68,675,405	68,754,539	65971	in gene	7.00
NM_009670, NM_146005, NM_170687, NM_170688, NM_170689, NM_170690, NM_170728, NM_170729, NM_170730	Ank3	ankyrin 3, epithelial	10	68,996,456	69,490,184	262536	in gene	5.00
NM_025807	Slc16a9	solute carrier family 16 (monocarboxylic acid transporters), member 9	10	69,708,024	69,748,699	14056	in gene	7.00
NM_001081412	Bcr	breakpoint cluster region homolog	10	74,523,641	74,645,695	97831	in gene	6.00
NM_172549	Cabin1	calcineurin binding protein 1	10	75,108,855	75,227,102	-9266	upstream	6.00
NM_010027	Ddt	D-dopachrome tautomerase	10	75,233,978	75,236,119	-249	upstream	6.00
NM_133994	Gstt3	glutathione S-transferase, theta 3	10	75,236,867	75,244,159	7791	downstream	6.00
NM_008185	Gstt1	glutathione S-transferase, theta 1	10	75,246,560	75,261,329	24961	downstream	6.00
NM_011418	Smarcb1	SWI/SNF related, matrix associated, actin dependent regulator of chromatin, subfamily b, member 1	10	75,359,520	75,384,333	8381	in gene	5.00
NM_008606	Mmp11	matrix metallopeptidase 11	10	75,385,969	75,395,208	19256	downstream	5.00
NM_001081419	Dip2a	DIP2 disco-interacting protein 2 homolog A (Drosophila)	10	75,725,794	75,808,007	45399	in gene	5.00
NM_146007	Col6a2	procollagen, type VI, alpha 2	10	76,058,507	76,086,059	-149	upstream	6.00
NM_021568	Pcbp3	poly(rC) binding protein 3	10	76,224,602	76,424,533	48949, -139	in gene, upstream	6.00
NM_009929	Col18a1	procollagen, type XVIII, alpha 1	10	76,514,924	76,629,246	34302, 4478	in gene, in gene	8.00
NM_026431	1810043G02Rik	RIKEN cDNA 1810043G02 gene	10	77,441,395	77,448,183	-131	upstream	8.00

Accession No.	Symbol	Gene Title	Chr	Gene Start	Gene End	Interval Dists to Start	Interval Pos	Avg Peak
NM_008826	Pfkl	phosphofructokinase, liver, B-type	10	77,450,016	77,472,494	31230	downstream	8.00
NM_172134	Pdxk	pyridoxal (pyridoxine, vitamin B6) kinase	10	77,901,715	77,927,720	4200	in gene	6.00
NM_173751	Ilvbl	ilvB (bacterial acetolactate synthase)-like	10	78,037,287	78,047,238	-3447	upstream	6.00
NM_027422	Mier2	mesoderm induction early response 1, family member 2	10	79,002,990	79,017,836	6604	in gene	5.00
NM_013591	Madcam1	mucosal vascular addressin cell adhesion molecule 1	10	79,127,328	79,131,281	7840	downstream	5.00
NM_148934	Gtrgeo22	gene trap ROSA b-geo 22	10	79,132,155	79,138,871	3013, 12293	in gene, downstream	5.50
NM_177613	Cdc34	cell division cycle 34 homolog (S. cerevisiae)	10	79,144,940	79,151,143	-9772, -492	upstream, upstream	5.50
NM_008504	Gzmm	granzyme M (lymphocyte met-ase 1)	10	79,151,765	79,158,005	-7317	upstream	6.00
NM_023304	Fgf22	fibroblast growth factor 22	10	79,217,864	79,219,706	11256	downstream	7.00
NM_144528	Rnf126	ring finger protein 126	10	79,221,284	79,229,669	549	in gene	7.00
NM_011492	Stk11	serine/threonine kinase 11	10	79,578,947	79,593,424	15325	downstream	5.00
NM_007705	Cirbp	cold inducible RNA binding protein	10	79,630,586	79,634,398	-2714	upstream	9.00
NM_027207	1600002K03Rik	RIKEN cDNA 1600002K03 gene	10	79,635,689	79,637,864	-7817	upstream	9.00
NM_007909	Efna2	ephrin A2	10	79,642,227	79,652,390	8461	in gene	6.00
NM_025650	Uqcr	ubiquinol-cytochrome c reductase (6.4kD) subunit	10	79,865,742	79,869,566	6910	downstream	9.00
NM_011548	Tcfe2a	transcription factor E2a	10	79,872,339	79,896,352	33696, 1136	downstream, in gene	7.50
NM_026094	Atp8b3	ATPase, Class I, type 8B, member 3	10	79,982,330	80,001,869	4509	in gene	5.00
NM_025852	Rexo1	REX1, RNA exonuclease 1 homolog (S. cerevisiae)	10	80,003,671	80,024,305	26945	downstream	5.00
NM_019575	Scamp4	secretory carrier membrane protein 4	10	80,065,627	80,078,528	4517	in gene	5.00
NM_007460	Ap3d1	adaptor-related protein complex 3, delta 1 subunit	10	80,169,723	80,204,956	-628	upstream	5.00
NM_008753	Oaz1	ornithine decarboxylase antizyme	10	80,289,401	80,292,035	9671	downstream	6.00
NM_001013758	BC072620	cDNA sequence BC072620	10	80,295,546	80,306,775	7703, -6105	in gene, upstream	5.50
NM_025349	Lsm7	LSM7 homolog, U6 small nuclear RNA associated (S. cerevisiae)	10	80,315,570	80,317,954	5074	downstream	5.00
NM_175195	3110056O03Rik	RIKEN cDNA 3110056O03 gene	10	80,318,020	80,331,453	-5140	upstream	5.00
NM_021501	Pias4	protein inhibitor of activated STAT 4	10	80,616,033	80,630,559	14383	in gene	4.00
NM_010768	Matk	megakaryocyte-associated tyrosine kinase	10	80,720,290	80,725,726	10782	downstream	9.00
NM_025317	Mrpl54	mitochondrial ribosomal protein L54	10	80,727,467	80,729,671	-1401	upstream	9.00
NM_018758	Apba3	amyloid beta (A4) precursor protein-binding, family A, member 3	10	80,730,985	80,735,973	87	in gene	9.00
NM_027381	2510012J08Rik	RIKEN cDNA 2510012J08 gene	10	80,783,942	80,788,987	8954	downstream	5.00
NM_009325	Tbxa2r	thromboxane A2 receptor	10	80,791,476	80,797,917	1420	in gene	5.00
NM_148951	Gipc3	GIPC PDZ domain containing family, member 3	10	80,800,507	80,806,011	13115, -8725	downstream, upstream	5.50
NM_010440	Hmg20b	high mobility group 20 B	10	80,808,797	80,813,155	-1581	upstream	6.00
NM_028657	F630110N24Rik	RIKEN cDNA F630110N24 gene	10	80,820,236	80,828,967	-5500, 7332	upstream, in gene	5.50
NM_001014836	4930404N11Rik	RIKEN cDNA 4930404N11 gene	10	80,826,794	80,828,535	967	in gene	5.00
NM_019757	Fzr1	fizzy/cell division cycle 20 related 1 (Drosophila)	10	80,829,624	80,841,115	13547, -21	downstream, upstream	6.00
NM_133964	Dohh	deoxyhypusine hydroxylase/monooxygenase	10	80,847,256	80,851,097	-6120	upstream	7.00
NM_008688, NM_026756	Nfic	nuclear factor I/C	10	80,858,936	80,889,918	18174, 13182, -6914	in gene, in gene, upstream	7.67
XM_618789, XM_985040	Brunol5	bruno-like 5, RNA binding protein (Drosophila)	10	80,924,959	80,945,429	5509	in gene	7.00
NM_134009	Ncln	nicalin homolog (zebrafish)	10	80,948,994	80,959,101	19181	downstream	7.00
NM_001042513, NM_001042514, NM_001042523, NM_015762	Txnrd1	thioredoxin reductase 1	10	82,296,696	82,360,469	63864, 69789	downstream, downstream	5.00
NM_001024918, NM_027689	Rfx4	regulatory factor X, 4 (influences HLA class II expression)	10	84,218,793	84,369,283	81591	in gene	7.00
NM_001017525, NM_028709	Btbd11	BTB (POZ) domain containing 11	10	84,850,564	85,123,037	61756, 113836, 147036	in gene, in gene, in gene	5.33
NM_181650	Prdm4	PR domain containing 4	10	85,354,711	85,379,690	3834	in gene	5.00
NM_175418	8030451F13Rik	RIKEN cDNA 8030451F13 gene	10	87,981,027	88,067,759	6959	in gene	7.00
NM_053072	Fgd6	FYVE, RhoGEF and PH domain containing 6	10	93,498,746	93,604,749	33094, 112294	in gene, downstream	6.00
NM_011629	Nr2c1	nuclear receptor subfamily 2, group C, member 1	10	93,619,031	93,658,096	-7991, 42585	upstream, downstream	6.50
NM_025551	Ndufa12	NADH dehydrogenase (ubiquinone) 1 alpha subcomplex, 12	10	93,661,754	93,683,693	-138	upstream	5.00
NM_172051	Tmcc3	transmembrane and coiled coil domains 3	10	93,977,624	94,053,699	312	in gene	5.00
NM_009950	Cradd	CASP2 and RIPK1 domain containing adaptor with death domain	10	94,637,380	94,786,731	96075	in gene	5.00
NM_027740	Wdr51b	WD repeat domain 51B	10	98,569,805	98,660,622	65171	in gene	7.00
NM_026268	Dusp6	dual specificity phosphatase 6	10	98,725,865	98,730,118	-1337	upstream	6.00
NM_001033332	Tmtc3	transmembrane and tetratricopeptide repeat containing 3	10	99,910,818	99,949,981	285	in gene	5.00
NM_207522	BC067068	cDNA sequence BC067068	10	105,200,303	105,278,410	10	in gene	7.00
NM_025602	Ccdc59	coiled-coil domain containing 59	10	105,278,535	105,284,566	-135	upstream	7.00
NM_027892	Ppp1r12a	protein phosphatase 1, regulatory (inhibitor) subunit 12A	10	107,599,456	107,714,631	-768	upstream	6.00
NM_007792	Csrp2	cysteine and glycine-rich protein 2	10	110,357,235	110,376,678	-115	upstream	8.00
NM_001033261	Ccdc131	coiled-coil domain containing 131	10	114,822,015	114,869,827	43553	in gene	7.00
NM_029928	Ptprb	protein tyrosine phosphatase, receptor type, B	10	115,738,562	115,820,989	83422	downstream	6.00

Accession No.	Symbol	Gene Title	Chr	Gene Start	Gene End	Interval Dists to Start	Interval Pos	Avg Peak
XM_001000571, XM_137270	Kifc5c	kinesin family member C5C	10	117,027,299	117,032,998	685	in gene	9.00
NM_029875	Slc35e3	solute carrier family 35, member E3	10	117,170,734	117,183,414	14438	downstream	6.00
NM_024457	Rap1b	RAS related protein 1b	10	117,251,653	117,283,030	-170	upstream	7.00
XM_125901, XM_904108	Cand1	cullin associated and neddylation disassociated 1	10	118,635,868	118,735,810	59426	in gene	7.00
NM_011915	Wif1	Wnt inhibitory factor 1	10	120,471,286	120,537,698	-326	upstream	4.00
XM_001481269, XM_137322	4930505D03Rik	RIKEN cDNA 4930505D03 gene	10	120,700,475	120,789,326	36590	in gene	6.00
NM_173022	BC048403	cDNA sequence BC048403	10	121,176,993	121,189,915	18751	downstream	6.00
NM_001033262	March9	membrane-associated ring finger (C3HC4) 9	10	126,493,106	126,497,240	-3144	upstream	7.00
NM_009870	Cdk4	cyclin-dependent kinase 4	10	126,500,671	126,504,451	-287	upstream	7.00
NM_025982	Tspan31	tetraspanin 31	10	126,504,346	126,507,317	6933	downstream	7.00
NM_028027	D10Ertd610e	DNA segment, Chr 10, ERATO Doi 610, expressed	10	126,619,585	126,627,075	-2701	upstream	5.00
NM_030714	Dtx3	deltex 3 homolog (Drosophila)	10	126,627,434	126,632,765	2989	in gene	5.00
NM_054097	Pip5k2c	phosphatidylinositol-4-phosphate 5-kinase, type II, gamma	10	126,634,123	126,648,602	18826	downstream	5.00
NM_027151	Dctn2	dynactin 2	10	126,703,455	126,718,863	24225	downstream	7.00
NM_033072	Mbd6	methyl-CpG binding domain protein 6	10	126,719,012	126,725,827	-1853	upstream	7.00
XM_001479400	LOC100043045	hypothetical protein LOC100043045	10	126,726,751	126,727,983	303	in gene	7.00
NM_007837	Ddit3	DNA-damage inducible transcript 3	10	126,727,866	126,733,342	-186	upstream	7.00
NM_001003913	Mars	methionine-tRNA synthetase	10	126,733,282	126,748,695	21015	downstream	7.00
NM_010296	Gli1	GLI-Kruppel family member GLI1	10	126,766,938	126,778,635	-9749	upstream	5.00
NM_008382	Inhbe	inhibin beta E	10	126,786,458	126,788,828	444, -2900	in gene, upstream	5.00
NM_010565	Inhbc	inhibin beta-C	10	126,793,378	126,807,486	19102, 15758	downstream, downstream	5.00
NM_008512	Lrp1	low density lipoprotein receptor-related protein 1	10	126,975,213	127,058,204	69948, 62236	in gene, in gene	7.50
NM_007412	Admr	adrenomedullin receptor	10	127,186,658	127,188,854	-7322	upstream	5.00
NM_080436	Rdh1	retinol dehydrogenase 1 (all trans)	10	127,196,866	127,205,352	-690	upstream	5.00
NM_013608	Naca	nascent polypeptide-associated complex alpha polypeptide	10	127,472,629	127,485,689	23387	downstream	6.00
NM_019766	Ptges3	prostaglandin E synthase 3 (cytosolic)	10	127,496,038	127,514,310	-22, 762, 27194	upstream, in gene, downstream	8.67
NM_016774	Atp5b	ATP synthase, H+ transporting mitochondrial F1 complex, beta subunit	10	127,520,363	127,527,444	2869, 8965, 13909	in gene, downstream, downstream	6.00
	Mirn677	microRNA 677	10	127,522,341	127,522,449	891, 6987	downstream, downstream	6.00
NM_054078	Baz2a	bromodomain adjacent to zinc finger domain, 2A	10	127,529,839	127,566,359	-6607, -511, 4433, 32465	upstream, upstream, in gene, in gene	6.25
XM_001479461	LOC100043085	similar to proline-rich protein BstNI subfamily 2	10	127,529,903	127,534,876	11644, 5548, 604	downstream, downstream, in gene	6.00
NM_001039080, NM_019711	Rbms2	RNA binding motif, single stranded interacting protein 2	10	127,566,526	127,617,353	55049, 32713	downstream, in gene	6.50
NM_133992	Usp52	ubiquitin specific peptidase 52	10	127,740,391	127,758,403	19465	downstream	4.00
NM_019953	Tmem4	transmembrane protein 4	10	127,759,515	127,764,243	341	in gene	4.00
NM_026444	Cs	citrate synthase	10	127,774,888	127,799,535	30696	downstream	9.00
NM_198160	Smarcc2	SWI/SNF related, matrix associated, actin dependent regulator of chromatin, subfamily c, member 2	10	127,896,350	127,927,228	39506	downstream	6.00
NM_010860	Myl6	myosin, light polypeptide 6, alkali, smooth muscle and non-muscle	10	127,927,917	127,930,881	-4975	upstream	6.00
NM_172259	Myl6b	myosin, light polypeptide 6B	10	127,931,213	127,935,741	-115	upstream	6.00
NM_134003	Zc3h10	zinc finger CCCH type containing 10	10	127,980,619	127,984,800	-928	upstream	6.00
NM_018860	Rpl41	ribosomal protein L41	10	127,985,173	127,986,172	444	in gene	6.00
NM_011119	Pa2g4	proliferation-associated 2G4	10	127,994,822	128,002,990	17262, -386	downstream, upstream	6.50
NM_010153	Erbb3	v-erb-b2 erythroblastic leukemia viral oncogene homolog 3 (avian)	10	128,006,424	128,026,557	23181	downstream	7.00
NM_016811	Dgka	diacylglycerol kinase, alpha	10	128,157,190	128,181,112	-4632	upstream	6.00
XM_001480976	LOC100043669	hypothetical protein LOC100043669	10	128,180,402	128,181,937	-3807	upstream	6.00
NM_030100	Wibg	within bgcn homolog (Drosophila)	10	128,185,018	128,203,146	726	in gene	6.00
NM_030207	Sfi1	Sfi1 homolog, spindle assembly associated (yeast)	11	3,031,853	3,093,466	69626	downstream	13.00
NM_029458	Hormad2	HORMA domain containing 2	11	4,246,386	4,341,085	4605	in gene	5.00
NM_023816	Ankrd36	ankyrin repeat domain 36	11	5,469,687	5,589,340	72121	in gene	5.00
XM_979372	LOC665181	similar to Endothelial differentiation-related factor 1 (EDF-1) (Multiprotein bridging factor 1) (MBF1) (Calmodulin-associated peptide 19) (CAP-19)	11	5,534,063	5,534,774	7745	downstream	5.00
NM_009636	Aebp1	AE binding protein 1	11	5,761,950	5,772,053	1010	in gene	6.00
NM_008894	Pold2	polymerase (DNA directed), delta 2, regulatory subunit	11	5,772,191	5,778,220	15260	downstream	6.00
NM_010292	Gck	glucokinase	11	5,800,826	5,849,602	29618	in gene	7.00
NM_007595	Camk2b	calcium/calmodulin-dependent protein kinase II, beta	11	5,869,675	5,965,751	45863	in gene	4.00
NM_008907	Ppia	peptidylprolyl isomerase A	11	6,315,873	6,319,813	671	in gene	8.00
XM_001476068, XM_907680	H2afv	H2A histone family, member V	11	6,327,229	6,344,442	1226	in gene	7.00

Accession No.	Symbol	Gene Title	Chr	Gene Start	Gene End	Interval Dists to Start	Interval Pos	Avg Peak
XM_975835	LOC665283	similar to SMT3 suppressor of mif two 3 homolog 2	11	6,353,134	6,354,045	-9918	upstream	7.00
NM_019511	Ramp3	receptor (calcitonin) activity modifying protein 3	11	6,558,536	6,577,478	2232	in gene	5.00
NM_001083587	Tns3	tensin 3	11	8,331,655	8,564,538	122410, 39578	in gene, in gene	5.50
NM_010789	Meis1	myeloid ecotropic viral integration site 1	11	18,780,431	18,918,683	48259, -1877	in gene, upstream	8.00
XM_001477234	LOC100041937	hypothetical protein LOC100041937	11	19,819,254	19,820,410	1162, 5898	downstream, downstream	6.00
NM_033523	Spred2	sprouty-related, EVH1 domain containing 2	11	19,824,445	19,922,600	-4029, 707	upstream, in gene	6.00
NM_001038625, NM_021372	Sertad2	SERTA domain containing 2	11	20,443,256	20,553,026	89064	in gene	5.00
NM_139297	Ugp2	UDP-glucose pyrophosphorylase 2	11	21,221,147	21,270,478	-786	upstream	6.00
NM_144514	Commd1	COMM domain containing 1	11	22,799,728	22,882,284	-8393	upstream	6.00
NM_009837	Cct4	chaperonin subunit 4 (delta)	11	22,890,593	22,903,336	84	in gene	6.00
NM_176841	A430106J12Rik	RIKEN cDNA A430106J12 gene	11	29,273,775	29,410,812	86321, 141249	in gene, downstream	5.50
NM_026465	2010316F05Rik	RIKEN cDNA 2010316F05 gene	11	29,413,055	29,415,033	9	in gene	5.00
XM_001479750	LOC100042990	hypothetical protein LOC100042990	11	29,415,086	29,417,355	-62, 11378	upstream, downstream	6.00
NM_133767	Mtif2	mitochondrial translational initiation factor 2	11	29,426,457	29,445,255	7	in gene	7.00
NM_026262	4930524B15Rik	RIKEN cDNA 4930524B15 gene	11	31,865,633	31,879,651	191	in gene	5.00
NM_177364	Sh3pxd2b	SH3 and PX domains 2B	11	32,247,811	32,328,183	77317, 78205	in gene, in gene	5.00
NM_173784	Ubtd2	ubiquitin domain containing 2	11	32,355,372	32,418,709	15	in gene	5.00
NM_134015	Fbxw11	F-box and WD-40 domain protein 11	11	32,542,875	32,646,816	-155	upstream	5.00
NM_008005	Fgf18	fibroblast growth factor 18	11	33,017,430	33,047,400	24312	in gene	5.00
XM_001474176	LOC100039945	similar to putative oral cancer suppressor	11	48,638,620	48,640,157	-4451	upstream	8.00
NM_053166	Trim7	tripartite motif protein 7	11	48,643,941	48,654,919	667	in gene	8.00
NM_021510	Hnrph1	heterogeneous nuclear ribonucleoprotein H1	11	50,191,221	50,200,030	-298	upstream	6.00
NM_153393	Col23a1	procollagen, type XXIII, alpha 1	11	51,103,422	51,394,612	132930, 139746	in gene, in gene	6.50
NM_028398	Agxt2l2	alanine-glyoxylate aminotransferase 2-like 2	11	51,398,305	51,416,539	21279	downstream	7.00
NM_010448	Hnrpab	heterogeneous nuclear ribonucleoprotein A/B	11	51,413,602	51,420,383	799	in gene	7.00
NM_029976	D11Ertd497e	DNA segment, Chr 11, ERATO Doi 497, expressed	11	51,781,165	51,790,838	-4493	upstream	6.00
NM_008300	Hspa4	heat shock protein 4	11	53,073,316	53,113,981	48685	downstream	5.00
NM_011031	P4ha2	procollagen-proline, 2-oxoglutarate 4-dioxygenase (proline 4-hydroxylase), alpha II polypeptide	11	53,915,018	53,945,150	38614	downstream	10.00
NM_178626	Cdc42se2	CDC42 small effector 2	11	54,531,004	54,601,138	50	in gene	8.00
NM_001029988	Fat2	FAT tumor suppressor homolog 2 (Drosophila)	11	55,064,112	55,125,759	55535	in gene	5.00
NM_020492	Glra1	glycine receptor, alpha 1 subunit	11	55,328,315	55,421,292	72668	in gene	6.00
NM_134189	Galnt10	UDP-N-acetyl-alpha-D-galactosamine:polypeptide N-acetylgalactosaminyltransferase 10	11	57,458,944	57,601,003	-96, 123264, 141440	upstream, in gene, in gene	6.00
NM_001045521	Gm249	gene model 249, (NCBI)	11	59,186,171	59,189,155	-9021	upstream	8.00
NM_144521	1110031B06Rik	RIKEN cDNA 1110031B06 gene	11	59,220,652	59,263,458	26530	in gene	4.00
NM_178417	BC050078	cDNA sequence BC050078	11	59,274,699	59,285,976	-5176	upstream	5.00
NM_012027, NM_201245	AA536749	expressed sequence AA536749	11	59,476,034	59,589,724	61502, 81454	in gene, in gene	6.00
NM_001039092, NM_001039093, NM_153080	Tom1l2	target of myb1-like 2 (chicken)	11	60,040,216	60,166,407	73223, 56615	in gene, in gene	5.50
NM_145427	Atpaf2	ATP synthase mitochondrial F1 complex assembly factor 2	11	60,214,126	60,230,507	-1045	upstream	4.00
NM_025757	4933439F18Rik	RIKEN cDNA 4933439F18 gene	11	60,230,805	60,258,779	747	in gene	4.00
NM_010862, NM_182698	Myo15	myosin XV	11	60,282,841	60,341,871	52423	in gene	4.00
NM_025294	Gtlf3b	gene trap locus F3b	11	60,715,748	60,727,292	-1700	upstream	5.00
NM_010603	Kcnj12	potassium inwardly-rectifying channel, subfamily J, member 12	11	60,879,306	60,884,631	3942, 5366	in gene, downstream	6.50
NM_013881	Ulk2	Unc-51 like kinase 2 (C. elegans)	11	61,589,162	61,668,119	4967	in gene	5.00
XM_001479316	LOC100042766	hypothetical protein LOC100042766	11	62,563,799	62,564,849	7433	downstream	6.00
NM_145136, NM_146386	Myocd	myocardin	11	64,990,063	65,083,482	-38	upstream	5.00
XM_110968, XM_917040	Dnahc9	dynein, axonemal, heavy chain 9	11	65,644,826	65,982,053	322725, 308805	in gene, in gene	5.50
NM_008744	Ntn1	netrin 1	11	68,022,866	68,200,328	84648, 1608	in gene, in gene	7.00
NM_023668	Ndel1	nuclear distribution gene E-like homolog 1 (A. nidulans)	11	68,634,936	68,666,564	14196	in gene	7.00
NM_177060	9930039A11Rik	RIKEN cDNA 9930039A11 gene	11	68,702,055	68,708,517	12985	downstream	5.00
NM_009080	Rpl26	ribosomal protein L26	11	68,715,068	68,718,036	-28	upstream	5.00
NM_011786	Aloxe3	arachidonate lipoxygenase 3	11	68,939,879	68,962,616	30649	downstream	5.00
NM_009659	Alox12b	arachidonate 12-lipoxygenase, 12R type	11	68,970,574	68,983,293	-46, 5074	upstream, in gene	5.50
NM_001045525	Cyb5d1	cytochrome b5 domain containing 1	11	69,207,114	69,208,848	-1304	upstream	4.00
NM_030083	Lsmd1	LSM domain containing 1	11	69,209,293	69,210,173	859	in gene	4.00
NM_025915	Tmem88	transmembrane protein 88	11	69,210,021	69,211,736	1584	in gene	4.00
NM_007911	Efnb3	ephrin B3	11	69,367,627	69,373,680	12376, -5216	downstream, upstream	6.00
NM_144824	Wdr79	WD repeat domain 79	11	69,375,256	69,392,826	13930	in gene	7.00
XM_001479913	LOC100043030	similar to ribosomal protein S27 (metallopanstimulin 1)	11	69,387,322	69,387,686	-8426	upstream	7.00
NM_029348	Zbtb4	zinc finger and BTB domain containing 4	11	69,588,908	69,593,175	1540	in gene	6.00

Accession No.	Symbol	Gene Title	Chr	Gene Start	Gene End	Interval Dists to Start	Interval Pos	Avg Peak
NM_009601	Chrnb1	cholinergic receptor, nicotinic, beta polypeptide 1 (muscle)	11	69,597,539	69,609,445	18997	downstream	6.00
XM_484042, XM_978002	LOC432576	hypothetical LOC432576	11	69,690,186	69,691,607	-2777	upstream	7.00
NM_153143	Kctd11	potassium channel tetramerisation domain containing 11	11	69,691,179	69,694,908	524	in gene	7.00
NM_153788	Centb1	centaurin, beta 1	11	69,695,069	69,709,041	14657	downstream	7.00
NM_001013414	0610025P10Rik	RIKEN cDNA 0610025P10 gene	11	69,715,393	69,727,322	18943	downstream	6.00
NM_019726	Gps2	G protein pathway suppressor 2	11	69,727,694	69,730,093	6642	downstream	6.00
NM_181582	Eif5a	eukaryotic translation initiation factor 5A	11	69,730,224	69,734,843	507	in gene	6.00
NM_009204	Slc2a4	solute carrier family 2 (facilitated glucose transporter), member 4	11	69,755,788	69,761,692	-5188	upstream	6.00
NM_007864	Dlg4	discs, large homolog 4 (Drosophila)	11	69,832,366	69,858,270	7591	in gene	5.00
NM_007440	Alox12	arachidonate 12-lipoxygenase	11	70,054,957	70,068,843	747	in gene	5.00
NM_145429	Arrb2	arrestin, beta 2	11	70,246,155	70,254,176	6981	in gene	6.00
NM_025397	Med11	mediator of RNA polymerase II transcription, subunit 11 homolog (S. cerevisiae)	11	70,265,433	70,267,229	8231	downstream	7.00
NM_023158	Cxcl16	chemokine (C-X-C motif) ligand 16	11	70,267,736	70,273,486	-178	upstream	7.00
NM_001029929	Zmynd15	zinc finger, MYND-type containing 15	11	70,273,124	70,279,701	540	in gene	7.00
NM_178367	Dhx33	DEAH (Asp-Glu-Ala-His) box polypeptide 33	11	70,797,593	70,817,934	382	in gene	6.00
NM_033562	Derl2	Der1-like domain family, member 2	11	70,820,947	70,832,765	15213	downstream	6.00
NM_144526	6720460F02Rik	RIKEN cDNA 6720460F02 gene	11	71,856,004	71,860,872	10364	downstream	5.00
NM_001024927, NM_001081641	Pitpnm3	PITPNM family member 3	11	71,861,030	71,949,391	83023	in gene	5.00
NM_001004148	Slc13a5	solute carrier family 13 (sodium-dependent citrate transporter), member 5	11	72,055,585	72,080,106	12922	in gene	4.00
NM_001037713	Fbxo39	F-box protein 39	11	72,115,167	72,122,270	7569	downstream	10.00
XM_892822	LOC628100	similar to F-box only protein 39	11	72,127,946	72,132,921	-5210	upstream	10.00
NM_011569	Tekt1	tektin 1	11	72,158,222	72,175,382	23414	downstream	6.00
NM_177776	D130058I21Rik	RIKEN cDNA D130058I21 gene	11	72,203,616	72,225,215	927	in gene	6.00
NM_027819	Ggt6	gamma-glutamyltransferase 6	11	72,249,028	72,251,909	5788	downstream	7.00
NM_016776	Mybbp1a	MYB binding protein (P160) 1a	11	72,254,880	72,265,052	-64	upstream	7.00
NM_018883	Camkk1	calcium/calmodulin-dependent protein kinase kinase 1, alpha	11	72,832,510	72,855,575	18386	in gene	6.00
NM_145099	Trpv3	transient receptor potential cation channel, subfamily V, member 3	11	73,081,122	73,110,702	34334	downstream	5.00
NM_023113	Aspa	aspartoacylase (aminoacylase) 2	11	73,118,490	73,138,136	22680	downstream	5.00
NM_001015046	Garnl4	GTPase activating RANGAP domain-like 4	11	74,196,985	74,403,660	63548	in gene	8.00
NM_197943	Rutbc1	RUN and TBC1 domain containing 1	11	74,662,766	74,710,582	518	in gene	6.00
NM_177325	Tsr1	TSR1, 20S rRNA accumulation, homolog (yeast)	11	74,711,582	74,722,842	-1518	upstream	6.00
NM_013761	Srr	serine racemase	11	74,720,298	74,739,205	29141	downstream	6.00
NM_177708	Rtn4rl1	reticulon 4 receptor-like 1	11	75,007,495	75,081,264	69593	in gene	7.00
NM_001004157	Scarf1	scavenger receptor class F, member 1	11	75,327,043	75,340,082	16973	downstream	6.00
NM_173388	Slc43a2	solute carrier family 43, member 2	11	75,345,210	75,388,543	-1194	upstream	6.00
NM_008916	Pps	putative phosphatase	11	75,444,522	75,462,367	17286, 20278, 25382	in gene, downstream, downstream	7.00
NM_001080774, NM_001080775, NM_008659	Myo1c	myosin IC	11	75,465,011	75,488,145	-3203, -211, 4893, 19853	upstream, upstream, in gene, in gene	6.50
NM_133656	Crk	v-crk sarcoma virus CT10 oncogene homolog (avian)	11	75,492,812	75,519,596	-7948	upstream	5.00
NM_026664	Vps53	vacuolar protein sorting 53 (yeast)	11	75,859,728	75,993,132	71841	in gene	4.00
NM_008750	Nxn	nucleoredoxin	11	76,070,738	76,212,626	136191	in gene	4.00
NM_177710	Ssh2	slingshot homolog 2 (Drosophila)	11	77,029,927	77,269,050	-247	upstream	9.00
NM_144825	Taok1	TAO kinase 1	11	77,342,664	77,421,317	-91	upstream	7.00
NM_001024205	Nufip2	nuclear fragile X mental retardation protein interacting protein 2	11	77,499,657	77,531,468	32519	downstream	5.00
NM_009965	Cryba1	crystallin, beta A1	11	77,532,234	77,538,795	6619	downstream	5.00
NM_001040403, NM_008028	Flot2	flotillin 2	11	77,851,443	77,873,934	21053	in gene	6.00
NM_009143	Sdf2	stromal cell derived factor 2	11	78,059,248	78,068,988	16032	downstream	6.00
NM_001002004	2610507B11Rik	RIKEN cDNA 2610507B11 gene	11	78,075,258	78,104,125	22	in gene	6.00
NM_172795	A830091I15Rik	RIKEN cDNA A830091I15 gene	11	78,286,793	78,311,205	-3131	upstream	4.00
NM_011707	Vtn	vitronectin	11	78,312,788	78,315,826	1548	in gene	4.00
NM_008759	Og9x	OG9 homeobox gene	11	78,317,015	78,318,583	-2679	upstream	4.00
NM_199199	AI316787	expressed sequence AI316787	11	78,320,560	78,325,670	11334	downstream	4.00
NM_026389	Poldip2	polymerase (DNA-directed), delta interacting protein 2	11	78,325,798	78,336,238	13258, 14490	downstream, downstream	5.00
NM_009395	Tnfaip1	tumor necrosis factor, alpha-induced protein 1 (endothelial)	11	78,336,350	78,349,696	10640, 9408	in gene, in gene	5.00
NM_018854	Ift20	intraflagellar transport 20 homolog (Chlamydomonas)	11	78,349,938	78,354,975	-9650	upstream	5.00
NM_175543	Rab11fip4	RAB11 family interacting protein 4 (class II)	11	79,404,714	79,507,514	70251	in gene	5.00
	Mirn365-2	microRNA 365-2	11	79,539,901	79,540,012	-3677	upstream	6.00
NM_018776	Crlf3	cytokine receptor-like factor 3	11	79,860,001	79,894,459	28262	in gene	5.00
NM_139228	Rhbdl3	rhomboid, veinlet-like 3 (Drosophila)	11	80,114,578	80,169,457	-8994	upstream	6.00
NM_019816	Aatf	apoptosis antagonizing transcription factor	11	84,236,358	84,327,003	5419	in gene	4.00
NM_153144	Ggnbp2	gametogenetin binding protein 2	11	84,645,863	84,684,240	-9568	upstream	8.00

Accession No.	Symbol	Gene Title	Chr	Gene Start	Gene End	Interval Dists to Start	Interval Pos	Avg Peak
NM_001077636, NM_027388	Pigw	phosphatidylinositol glycan anchor biosynthesis, class W	11	84,689,815	84,693,787	-21, -3245	upstream, upstream	6.50
NM_001003908	Cltc	clathrin, heavy polypeptide (Hc)	11	86,508,155	86,570,994	-590	upstream	8.00
NM_025638	Gdpd1	glycerophosphodiester phosphodiesterase domain containing 1	11	86,847,390	86,887,550	42574	downstream	5.00
NM_031386	Tex14	testis expressed gene 14	11	87,299,788	87,369,325	53780	in gene	6.00
NM_133215	Mtmr4	myotubularin related protein 4	11	87,405,719	87,429,798	19177	in gene	6.00
NM_001045527	Hsf5	heat shock transcription factor family member 5	11	87,430,666	87,473,044	-5770	upstream	6.00
NM_026556	Dynll2	dynein light chain LC8-type 2	11	87,793,035	87,801,007	-145	upstream	7.00
NM_016686	Vezf1	vascular endothelial zinc finger 1	11	87,881,843	87,898,231	-179	upstream	6.00
NM_054043	Msi2	Musashi homolog 2 (Drosophila)	11	88,152,884	88,403,651	55747, 49667	in gene, in gene	6.00
NM_001042541, NM_009648	Akap1	A kinase (PRKA) anchor protein 1	11	88,692,106	88,725,900	76	in gene	7.00
NM_008796	Pctp	phosphatidylcholine transfer protein	11	89,844,731	89,864,208	22400	downstream	4.00
NM_172563	Hlf	hepatic leukemia factor	11	90,197,849	90,252,231	10823, -132	in gene, upstream	5.50
NM_028011	Tom1l1	target of myb1-like 1 (chicken)	11	90,507,005	90,548,915	23619	in gene	6.00
NM_001025428, NM_001025429, NM_001025430, NM_027569	Spag9	sperm associated antigen 9	11	93,857,405	93,987,397	803	in gene	5.00
XM_001473216	LOC100039440	hypothetical protein LOC100039440	11	93,999,844	94,017,090	18764	downstream	5.00
NM_181819	Wfikkn2	WAP, follistatin/kazal, immunoglobulin, kunitz and netrin domain containing 2	11	94,098,912	94,103,863	6135, -361	downstream, upstream	8.50
NM_009783	Cacna1g	calcium channel, voltage-dependent, T type, alpha 1G subunit	11	94,269,830	94,335,039	41087, 12543	in gene, in gene	6.00
NM_001013381	Rsad1	radical S-adenosyl methionine domain containing 1	11	94,401,112	94,410,513	-15	upstream	6.00
NM_007742	Col1a1	procollagen, type I, alpha 1	11	94,797,584	94,813,181	688	in gene	6.00
NM_007867	Dlx4	distal-less homeobox 4	11	95,001,770	95,007,577	153	in gene	6.00
NM_172543	5730593F17Rik	RIKEN cDNA 5730593F17 gene	11	95,198,332	95,243,184	356, 33028	in gene, in gene	7.00
XM_112540, XM_923886	Gm53	gene model 53, (NCBI)	11	96,112,908	96,125,798	1380, 20500	in gene, downstream	5.00
	Mirn196a-1	microRNA 196a-1	11	96,126,477	96,126,578	6931	downstream	5.00
NM_008270	Hoxb9	homeo box B9	11	96,132,644	96,137,909	764	in gene	5.00
NM_010461	Hoxb8	homeo box B8	11	96,143,219	96,146,639	-9811	upstream	5.00
NR_003293	D030028A08Rik	RIKEN cDNA D030028A08 gene	11	96,805,504	96,826,374	12656	in gene	8.00
NM_001080964, NM_030220	Sp2	Sp2 transcription factor	11	96,814,651	96,839,002	20842	in gene	8.00
NM_026154	Mrpl10	mitochondrial ribosomal protein L10	11	96,902,900	96,910,527	10820	downstream	4.00
NM_001081434	Osbpl7	oxysterol binding protein-like 7	11	96,912,134	96,930,218	1586	in gene	4.00
NM_198100	Tbkbp1	TBK1 binding protein 1	11	96,997,487	97,011,026	-2990	upstream	7.00
NM_021493	4933428G20Rik	RIKEN cDNA 4933428G20 gene	11	97,311,474	97,363,714	286	in gene	8.00
NM_001081435	Fbxo47	F-box protein 47	11	97,715,621	97,745,468	-4	upstream	5.00
NM_028199	Plxdc1	plexin domain containing 1	11	97,784,552	97,847,694	-8178	upstream	5.00
NM_207231	Arl5c	ADP-ribosylation factor-like 5C	11	97,850,892	97,857,487	1615	in gene	5.00
NM_031173, NM_145121	Cacnb1	calcium channel, voltage-dependent, beta 1 subunit	11	97,864,215	97,884,337	28465	downstream	5.00
NM_001080118, NM_013634, NM_134027	Pparbp	peroxisome proliferator activated receptor binding protein	11	98,013,468	98,054,607	175	in gene	6.00
XM_001473891	LOC100039982	hypothetical protein LOC100039982	11	98,064,101	98,064,612	10180	downstream	6.00
NM_001003817	Erbb2	v-erb-b2 erythroblastic leukemia viral oncogene homolog 2, neuro/glioblastoma derived oncogene homolog (avian)	11	98,273,798	98,299,030	22442	in gene	5.00
NM_025559	1810046J19Rik	RIKEN cDNA 1810046J19 gene	11	98,299,022	98,300,302	4062	downstream	5.00
NM_178060	Thra	thyroid hormone receptor alpha	11	98,603,187	98,626,425	15789	in gene	6.00
NM_028722	4121402D02Rik	RIKEN cDNA 4121402D02 gene	11	98,656,830	98,667,741	-318	upstream	6.00
NM_138660	Casc3	cancer susceptibility candidate 3	11	98,666,219	98,695,121	-9707	upstream	6.00
NM_009024	Rara	retinoic acid receptor, alpha	11	98,799,010	98,836,256	17310	in gene	5.00
NM_172564	Tns4	tensin 4	11	98,926,993	98,950,616	17048	in gene	7.00
NM_176830	1110036O03Rik	RIKEN cDNA 1110036O03 gene	11	100,270,062	100,276,133	213	in gene	7.00
NM_010221	Fkbp10	FK506 binding protein 10	11	100,277,011	100,286,139	-1091, 17901	upstream, downstream	6.50
NM_026561	Nt5c3l	5'-nucleotidase, cytosolic III-like	11	100,283,635	100,302,388	26468, 7476	downstream, in gene	6.50
NM_025727	Klhl10	kelch-like 10 (Drosophila)	11	100,303,238	100,318,338	-8326	upstream	6.00
NM_028918	Ttc25	tetratricopeptide repeat domain 25	11	100,406,946	100,433,880	34286	downstream	4.00
NM_009923	Cnp1	cyclic nucleotide phosphodiesterase 1	11	100,436,289	100,443,039	4943	in gene	4.00
NM_019795	Dnajc7	DnaJ (Hsp40) homolog, subfamily C, member 7	11	100,444,150	100,481,482	40250	downstream	4.00
NM_016920	Atp6v0a1	ATPase, H+ transporting, lysosomal V0 subunit A1	11	100,870,769	100,925,028	9935, 38927	in gene, in gene	4.50
NM_134028	Tubg2	tubulin, gamma 2	11	101,017,235	101,023,101	11853	downstream	8.00
NM_146030	Plekhh3	pleckstrin homology domain containing, family H (with MyTH4 domain) member 3	11	101,023,993	101,032,616	3528	in gene	8.00
NM_007721	Ccr10	chemokine (C-C motif) receptor 10	11	101,034,312	101,036,757	7669	downstream	8.00
NM_016782	Cntnap1	contactin associated protein-like 1	11	101,037,431	101,052,034	-8343	upstream	8.00
NM_007970	Ezh1	enhancer of zeste homolog 1 (Drosophila)	11	101,052,430	101,089,262	-2962	upstream	8.00
NM_019584	Becn1	beclin 1 (coiled-coil, myosin-like BCL2-interacting protein)	11	101,149,581	101,163,581	-67	upstream	5.00

Accession No.	Symbol	Gene Title	Chr	Gene Start	Gene End	Interval Dists to Start	Interval Pos	Avg Peak
NM_008815	Etv4	ets variant gene 4 (E1A enhancer binding protein, E1AF)	11	101,631,056	101,646,624	-2096, -6005	upstream, upstream	5.50
NM_001044383, NM_011551	Ubtf	upstream binding transcription factor, RNA polymerase I	11	102,165,874	102,180,410	11514	in gene	6.00
NM_016759	Rap2ip	Rap2 interacting protein	11	102,254,990	102,263,867	13714	downstream	9.00
NM_026542	Slc25a39	solute carrier family 25, member 39	11	102,264,461	102,268,786	82	in gene	9.00
NM_008175	Grn	granulin	11	102,291,821	102,298,119	3667	in gene	5.00
NM_199200	BC025575	cDNA sequence BC025575	11	102,298,295	102,308,977	13489	downstream	5.00
XM_001475276	LOC100040792	hypothetical protein LOC100040792	11	102,437,709	102,442,716	2828, 1948, 1372	in gene, in gene, in gene	5.00
NM_020510	Fzd2	frizzled homolog 2 (Drosophila)	11	102,465,745	102,469,372	-9660, 47	upstream, in gene	4.50
NM_080846	Higd1b	HIG1 domain family, member 1B	11	102,697,610	102,699,354	5158	downstream	5.00
NM_011431	Eftud2	elongation factor Tu GTP binding domain containing 2	11	102,699,791	102,742,231	39463, -5161	in gene, upstream	5.00
NM_028492	Ccdc103	coiled-coil domain containing 103	11	102,742,558	102,746,529	4834	downstream	5.00
XM_001479358	LOC100042500	similar to Coiled-coil domain containing 103	11	102,745,870	102,748,045	1522	in gene	5.00
NM_010277	Gfap	glial fibrillary acidic protein	11	102,749,991	102,758,445	11053	downstream	5.00
NM_026551	Dcakd	dephospho-CoA kinase domain containing	11	102,855,370	102,878,461	15597	in gene	5.00
NM_001038609, NM_010838	Mapt	microtubule-associated protein tau	11	104,092,750	104,193,410	76914	in gene	4.00
NM_001081045	1700081L11Rik	RIKEN cDNA 1700081L11 gene	11	104,194,543	104,303,605	84458	in gene	6.00
XM_110936, XM_907217	EG217246	predicted gene, EG217246	11	105,856,045	105,866,757	17827	downstream	5.00
NM_001037712	Kcnh6	potassium voltage-gated channel, subfamily H (eag-related), member 6	11	105,869,517	105,895,378	4355, 10483	in gene, in gene	6.00
NM_027946	Wdr68	WD repeat domain 68	11	105,898,186	105,920,638	12630	in gene	5.00
NM_011947	Map3k3	mitogen activated protein kinase kinase kinase 3	11	105,946,216	106,016,760	27832	in gene	5.00
NM_028074	Ddx42	DEAD (Asp-Glu-Ala-Asp) box polypeptide 42	11	106,078,240	106,110,453	42112	downstream	7.00
NM_025310	Ftsj3	FtsJ homolog 3 (E. coli)	11	106,110,458	106,117,116	-3236	upstream	7.00
XM_001476085	LOC100041277	hypothetical protein LOC100041277	11	106,360,284	106,362,528	2052	in gene	7.00
NM_198292	Tex2	testis expressed gene 2	11	106,363,453	106,474,244	111908	downstream	7.00
NM_001033435	Gm885	gene model 885, (NCBI)	11	106,612,582	106,628,882	5562	in gene	5.00
NM_015810	Polg2	polymerase (DNA directed), gamma 2, accessory subunit	11	106,629,620	106,640,756	-9068	upstream	9.00
NM_007840	Ddx5	DEAD (Asp-Glu-Ala-Asp) box polypeptide 5	11	106,641,670	106,649,808	-16	upstream	9.00
NM_177088	Ccdc45	coiled-coil domain containing 45	11	106,650,589	106,680,143	-765	upstream	9.00
NM_010655	Kpna2	karyopherin (importin) alpha 2	11	106,849,943	106,860,839	-137	upstream	9.00
NM_145823	Pitpnc1	phosphatidylinositol transfer protein, cytoplasmic 1	11	107,069,206	107,332,034	154818, 76386, -94, -8686	in gene, in gene, upstream, upstream	5.75
NM_025894	Psmd12	proteasome (prosome, macropain) 26S subunit, non-ATPase, 12	11	107,340,842	107,359,350	-8714, -122	upstream, upstream	6.00
NM_011101	Prkca	protein kinase C, alpha	11	107,799,562	108,205,242	72154, 18938	in gene, in gene	6.00
NM_015732	Axin2	axin2	11	108,781,680	108,812,097	776	in gene	5.00
XM_196056, XM_920629	E030025P04Rik	RIKEN cDNA E030025P04 gene	11	109,000,603	109,005,685	4341, -43	in gene, upstream	7.50
XM_001477505, XM_001480042	1700096J18Rik	RIKEN cDNA 1700096J18 gene	11	109,208,181	109,214,965	15707	downstream	7.00
NM_010303	Gna13	guanine nucleotide binding protein, alpha 13	11	109,224,108	109,262,683	-220	upstream	7.00
NM_011943	Map2k6	mitogen activated protein kinase kinase 6	11	110,260,436	110,374,951	-3124	upstream	5.00
NM_027216	Slc39a11	solute carrier family 39 (metal ion transporter), member 11	11	113,106,169	113,427,075	84195	in gene	7.00
NM_172800	Sdk2	sidekick homolog 2 (chicken)	11	113,642,104	113,927,265	119249, 88865, 1	in gene, in gene, in gene	6.00
XM_001476930	LOC100041739	hypothetical protein LOC100041739	11	113,931,224	113,956,640	-3960	upstream	6.00
NM_173048	Gga3	golgi associated, gamma adaptin ear containing, ARF binding protein 3	11	115,445,569	115,465,220	-5020	upstream	6.00
NM_025305	Mrps7	mitchondrial ribosomal protein S7	11	115,465,465	115,468,938	4775	downstream	6.00
NM_027162	Mif4gd	MIF4G domain containing	11	115,469,232	115,473,817	3577	in gene	6.00
NM_026071	Slc25a19	solute carrier family 25 (mitochondrial thiamine pyrophosphate carrier), member 19	11	115,476,297	115,489,453	19213	downstream	6.00
NM_020483	Sap30bp	SAP30 binding protein	11	115,794,973	115,826,848	13027	in gene	6.00
NM_016905	Galk1	galactokinase 1	11	115,869,671	115,874,033	-8063	upstream	5.00
NM_008211	H3f3b	H3 histone, family 3B	11	115,883,275	115,885,818	3722	downstream	5.00
NM_172569	Unk	unkempt homolog (Drosophila)	11	115,891,636	115,922,508	-9540	upstream	5.00
NM_024177	Mrpl38	mitochondrial ribosomal protein L38	11	115,993,131	116,000,182	-10165	upstream	5.00
NM_172571	Fbf1	Fas (TNFRSF6) binding factor 1	11	116,003,599	116,029,492	19145, 5124	in gene, in gene	5.00
NM_015729	Acox1	acyl-Coenzyme A oxidase 1, palmitoyl	11	116,033,202	116,060,359	35991	downstream	5.00
XM_181318, XM_910489	2310004N24Rik	RIKEN cDNA 2310004N24 gene	11	116,060,169	116,076,634	22679	downstream	5.00
NM_027165	Cdk3	cyclin-dependent kinase 3	11	116,077,854	116,081,599	4994	downstream	5.00
NM_025276	Evpl	envoplakin	11	116,081,873	116,099,405	16557, -5299	in gene, upstream	5.00
NM_146032	Srp68	signal recognition particle 68	11	116,106,480	116,135,531	30827	downstream	5.00
NM_010254	Galr2	galanin receptor 2	11	116,142,253	116,145,229	8019	downstream	6.00
NM_172948	Mgat5b	mannoside acetylglucosaminyltransferase 5, isoenzyme B	11	116,780,177	116,848,258	35455	in gene	6.00
XM_001477442	LOC100042036	hypothetical protein LOC100042036	11	116,899,603	116,976,757	50517	in gene	5.00
NM_017380	Sept9	septin 9	11	117,193,034	117,223,621	26438	in gene	4.00
NM_001012273, NM_009689	Birc5	baculoviral IAP repeat-containing 5	11	117,710,551	117,717,057	16057	downstream	6.00

Accession No.	Symbol	Gene Title	Chr	Gene Start	Gene End	Interval Dists to Start	Interval Pos	Avg Peak
XM_621267, XM_911377	LOC546519	hypothetical LOC546519	11	117,722,066	117,725,673	4542	downstream	6.00
NM_027919	Tha1	threonine aldolase 1	11	117,729,265	117,734,795	8187	downstream	6.00
NM_007707	Socs3	suppressor of cytokine signaling 3	11	117,827,393	117,830,476	5676	downstream	6.00
XM_001481301, XM_126677	Dnahc17	dynein, axonemal, heavy chain 17	11	117,883,037	117,990,533	36597	in gene	4.00
NM_011180	Pscd1	pleckstrin homology, Sec7 and coiled-coil domains 1	11	118,025,489	118,109,874	63474	in gene	7.00
NM_019959	C1qtnf1	C1q and tumor necrosis factor related protein 1	11	118,295,167	118,311,277	-455, 2977	upstream, in gene	5.50
NM_001030291	Enpp7	ectonucleotide pyrophosphatase/phosphodiesterase 7	11	118,849,502	118,854,155	11282	downstream	7.00
NM_013926	Cbx8	chromobox homolog 8 (Drosophila Pc class)	11	118,899,750	118,902,227	-3277	upstream	4.00
NM_175430	Ccdc40	coiled-coil domain containing 40	11	119,089,886	119,126,526	21010	in gene	7.00
NM_130886	Card14	caspase recruitment domain family, member 14	11	119,176,101	119,206,689	38523	downstream	5.00
NM_018822	Sgsh	N-sulfoglucosamine sulfohydrolase (sulfamidase)	11	119,204,803	119,216,824	2200	in gene	5.00
NM_178743	Slc26a11	solute carrier family 26, member 11	11	119,216,871	119,242,390	-2247	upstream	5.00
XM_126613, XM_917071	Chmp6	chromatin modifying protein 6	11	119,775,124	119,780,866	-3199	upstream	5.00
NM_008244	Hgs	HGF-regulated tyrosine kinase substrate	11	120,329,005	120,345,293	22075	downstream	5.00
NM_027204	Mrpl12	mitochondrial ribosomal protein L12	11	120,345,983	120,350,068	5097	downstream	5.00
NM_013770	Slc25a10	solute carrier family 25 (mitochondrial carrier, dicarboxylate transporter), member 10	11	120,353,186	120,360,480	-2106	upstream	5.00
NM_008101	Gcgr	glucagon receptor	11	120,390,586	120,400,402	12902	downstream	5.00
NM_001033231	BC003940	cDNA sequence BC003940	11	120,404,202	120,411,041	7553	downstream	5.00
NM_026814	Dysfip1	dysferlin interacting protein 1	11	120,411,289	120,412,446	8958	downstream	5.00
NM_001038230, NM_025389	Anapc11	anaphase promoting complex subunit 11 homolog (yeast)	11	120,459,751	120,469,512	11001, 14505	downstream, downstream	4.00
NM_153288	Npb	neuropeptide B	11	120,469,791	120,470,414	961, 4465	downstream, downstream	4.00
NM_144795	Pycr1	pyrroline-5-carboxylate reductase 1	11	120,497,026	120,504,984	9992	downstream	6.00
NM_145370	Gps1	G protein pathway suppressor 1	11	120,645,829	120,650,416	11947	downstream	9.00
NM_026824	Dus1l	dihydrouridine synthase 1-like (S. cerevisiae)	11	120,650,516	120,657,709	-67	upstream	9.00
NM_007988	Fasn	fatty acid synthase	11	120,667,272	120,685,861	28085, 485	downstream, in gene	7.50
NM_027745	Ccdc57	coiled-coil domain containing 57	11	120,687,856	120,794,186	108810, 58810	downstream, in gene	6.50
NM_027874, NM_139059	Csnk1d	casein kinase 1, delta	11	120,823,055	120,852,647	1223	in gene	5.00
NM_025402	1110031I02Rik	RIKEN cDNA 1110031I02 gene	11	121,038,907	121,065,962	5210	in gene	7.00
NM_001001333	Hexdc	hexosaminidase (glycosyl hydrolase family 20, catalytic domain) containing	11	121,069,017	121,083,961	-8265	upstream	7.00
NM_026272	Narf	nuclear prelamin A recognition factor	11	121,098,567	121,117,169	22489	downstream	7.00
NM_001080932	Foxk2	forkhead box K2	11	121,116,808	121,171,210	4248	in gene	7.00
NM_144797	Metrnl	meteorin, glial cell differentiation regulator-like	11	121,563,741	121,578,703	5059	in gene	5.00
XM_001477246	LOC100041946	similar to 1700012B15Rik protein	12	3,235,100	3,236,468	644	in gene	6.00
XR_035176, XR_035415	1700012B15Rik	RIKEN cDNA 1700012B15 gene	12	3,235,511	3,238,286	233	in gene	6.00
XM_001472910, XM_001480129	Atad2b	ATPase family, AAA domain containing 2B	12	4,924,159	5,054,216	641	in gene	5.00
XM_906219, XM_978355	Klhl29	kelch-like 29 (Drosophila)	12	5,084,275	5,382,806	256150, 183958	in gene, in gene	6.00
NM_008640	Laptm4a	lysosomal-associated protein transmembrane 4A	12	8,928,113	8,945,548	-4049	upstream	5.00
NM_029007	AW125753	expressed sequence AW125753	12	14,154,404	14,158,844	1964	in gene	7.00
NM_015763, NM_172950	Lpin1	lipin 1	12	16,543,442	16,596,576	56299	downstream	5.00
XM_001001921	EG668514	predicted gene, EG668514	12	17,072,201	17,076,244	4999	downstream	5.00
NM_001081977, NM_080563	Rnf144	ring finger protein 144	12	26,991,662	27,100,121	106985	in gene	6.00
NM_181395	Pxdn	peroxidasin homolog (Drosophila)	12	30,622,901	30,700,724	4603	in gene	5.00
NM_008482	Lamb1-1	laminin B1 subunit 1	12	31,950,159	32,014,504	22193	in gene	6.00
NM_134048	Cbll1	Casitas B-lineage lymphoma-like 1	12	32,171,761	32,184,405	-891	upstream	4.00
NM_011658	Twist1	twist gene homolog 1 (Drosophila)	12	34,642,536	34,644,696	9272	downstream	6.00
NM_013464	Ahr	aryl-hydrocarbon receptor	12	36,182,651	36,219,661	-19	upstream	7.00
NM_007960	Etv1	ets variant gene 1	12	39,506,897	39,594,790	-1569	upstream	6.00
NM_053122	Immp2l	IMP2 inner mitochondrial membrane peptidase-like (S. cerevisiae)	12	42,729,751	43,056,573	137865	in gene	6.00
NM_007728	Coch	coagulation factor C homolog (Limulus polyphemus)	12	52,694,442	52,706,760	374	in gene	5.00
NM_052973	Strn3	striatin, calmodulin binding protein 3	12	52,710,619	52,792,873	137	in gene	6.00
NM_021710	Ap4s1	adaptor-related protein complex AP-4, sigma 1	12	52,792,024	52,839,918	712	in gene	6.00
NM_207010	Mdga2	MAM domain containing glycosylphosphatidylinositol anchor 2	12	67,572,169	68,322,190	161742	in gene	13.00
NM_027269	1110034A24Rik	RIKEN cDNA 1110034A24 gene	12	70,290,825	70,299,416	1416	in gene	6.00
XR_035259, XR_035390	9330151L19Rik	RIKEN cDNA 9330151L19 gene	12	70,300,357	70,300,855	-2357	upstream	6.00
NM_011133	Pole2	polymerase (DNA directed), epsilon 2 (p59 subunit)	12	70,302,766	70,329,177	31177	downstream	6.00

Accession No.	Symbol	Gene Title	Chr	Gene Start	Gene End	Interval Dists to Start	Interval Pos	Avg Peak
NM_028127	Frmd6	FERM domain containing 6	12	71,926,501	72,003,221	-464	upstream	6.00
NM_011184	Psma3	proteasome (prosome, macropain) subunit, alpha type 3	12	72,075,680	72,095,908	5216	in gene	5.00
NM_021532	Dact1	dapper homolog 1, antagonist of beta-catenin (xenopus)	12	72,410,971	72,419,881	-171	upstream	5.00
NM_026102, NM_172464	Daam1	dishevelled associated activator of morphogenesis 1	12	72,932,065	73,093,354	-321, 119218	upstream, in gene	4.50
XM_001001604, XM_983512	EG666385	predicted gene, EG666385	12	74,894,470	74,909,398	3930	in gene	5.00
XM_001001795, XM_001476702, XM_001476717, XM_001479426	Wdr89	WD repeat domain 89	12	76,731,581	76,770,524	-324	upstream	6.00
NM_028356	Zbtb25	zinc finger and BTB domain containing 25	12	77,449,931	77,469,986	19570	in gene	4.00
NM_013675	Spnb1	spectrin beta 1	12	77,681,475	77,811,534	65006	in gene	5.00
NM_206534	Churc1	churchill domain containing 1	12	77,866,560	77,884,167	-80	upstream	5.00
NM_134050	Rab15	RAB15, member RAS oncogene family	12	77,898,949	77,923,503	1679	in gene	5.00
NM_013738	Plek2	pleckstrin 2	12	79,989,681	80,007,937	-4959	upstream	7.00
XM_001477600	LOC100041968	hypothetical protein LOC100041968	12	80,932,096	80,932,838	2358	downstream	7.00
NM_134156	Actn1	actinin, alpha 1	12	81,268,534	81,361,303	-409	upstream	5.00
NM_177267	Wdr22	WD repeat domain 22	12	81,437,669	81,537,526	94006, 58886	in gene, in gene	4.00
NM_001079694, NM_001079695, NM_009159	Sfrs5	splicing factor, arginine/serine-rich 5 (SRp40, HRS)	12	82,046,491	82,051,494	-219	upstream	4.00
NM_022316	Smoc1	SPARC related modular calcium binding 1	12	82,127,818	82,287,387	110694	in gene	6.00
NM_177395	Map3k9	mitogen-activated protein kinase kinase kinase 9	12	82,829,401	82,881,994	14746	in gene	5.00
NM_015812	Rgs6	regulator of G-protein signaling 6	12	83,718,025	84,259,799	135079, 147927	in gene, in gene	7.00
NM_130887	Papln	papilin, proteoglycan-like sulfated glycoprotein	12	85,104,611	85,133,302	4765	in gene	5.00
NM_172582	Coq6	coenzyme Q6 homolog (yeast)	12	85,702,997	85,714,740	20795	downstream	5.00
NM_001026214, NM_007647	Entpd5	ectonucleoside triphosphate diphosphohydrolase 5	12	85,714,807	85,749,979	26187	in gene	5.00
NM_013589	Ltbp2	latent transforming growth factor beta binding protein 2	12	86,124,162	86,217,445	68949	in gene	5.00
NM_030225	Dlst	dihydrolipoamide S-succinyltransferase (E2 component of 2-oxo-glutarate complex)	12	86,451,846	86,475,036	11882	in gene	5.00
NM_145138	Nek9	NIMA (never in mitosis gene a)-related expressed kinase 9	12	86,640,463	86,680,287	239	in gene	6.00
NM_026775	Tmed10	transmembrane emp24-like trafficking protein 10 (yeast)	12	86,681,564	86,715,667	35619	downstream	6.00
NM_001081423	Ttll5	tubulin tyrosine ligase-like family, member 5	12	87,165,660	87,394,852	144908	in gene	4.00
XM_892281, XM_906877	EG627607	predicted gene, EG627607	12	88,075,319	88,104,745	27737	in gene	7.00
NM_145836	6430527G18Rik	RIKEN cDNA 6430527G18 gene	12	88,221,651	88,225,761	-63	upstream	8.00
NM_001080943	Zdhhc22	zinc finger, DHHC-type containing 22	12	88,324,331	88,329,626	-5462	upstream	7.00
NM_181815	4930534B04Rik	RIKEN cDNA 4930534B04 gene	12	92,236,932	92,622,818	365506	in gene	7.00
XM_980161, XM_991883	LOC667688	similar to 60S ribosomal protein L17 (L23) (Amino acid starvation-induced protein) (ASI)	12	96,913,467	97,016,760	19813	in gene	6.00
NM_201518	Flrt2	fibronectin leucine rich transmembrane protein 2	12	96,930,464	97,019,235	2816	in gene	6.00
NM_011877	Ptpn21	protein tyrosine phosphatase, non-receptor type 21	12	99,914,952	99,972,804	36	in gene	7.00
NM_183186	Ches1	checkpoint suppressor 1	12	100,433,304	100,688,284	136428, 56988	in gene, in gene	6.50
XM_001473733	LOC100039892	hypothetical protein LOC100039892	12	100,686,807	100,802,177	-3311	upstream	6.00
XM_484171, XM_912077	BC002230	cDNA sequence BC002230	12	101,363,662	101,397,863	15239	in gene	7.00
NM_009790	Calm1	calmodulin 1	12	101,437,751	101,448,016	-135	upstream	6.00
XM_127105, XM_985528	Ttc7b	tetratricopeptide repeat domain 7B	12	101,538,602	101,759,042	215922	in gene	4.00
NM_011812	Fbln5	fibulin 5	12	102,984,775	103,065,261	14461	in gene	6.00
NM_023049	Asb2	ankyrin repeat and SOCS box-containing protein 2	12	104,559,352	104,594,211	14723	in gene	6.00
XM_001478583, XM_994648	EG668158	predicted gene, EG668158	12	109,444,555	109,513,627	57227, 52091	in gene, in gene	5.00
NM_001044380	1600002O04Rik	RIKEN cDNA 1600002O04 gene	12	109,544,480	109,566,510	27936	downstream	7.00
NM_010010	Cyp46a1	cytochrome P450, family 46, subfamily a, polypeptide 1	12	109,572,591	109,600,444	-175, 14385, 31217, 34465	upstream, in gene, downstream, downstream	7.00
NM_001043335, NM_001043336	Eml1	echinoderm microtubule associated protein like 1	12	109,648,865	109,777,774	33103, 136351	in gene, downstream	5.50
NM_007965	Evl	Ena-vasodilator stimulated phosphoprotein	12	109,792,930	109,926,726	-7714	upstream	6.00
NM_011710	Wars	tryptophanyl-tRNA synthetase	12	110,098,244	110,131,407	5239	in gene	5.00
XM_001474974, XM_001476637	Wdr25	WD repeat domain 25	12	110,132,513	110,266,663	-6345	upstream	5.00
NM_026048	2810452K22Rik	RIKEN cDNA 2810452K22 gene	12	112,110,832	112,127,324	-4772	upstream	5.00
NM_001081057	4930573I19Rik	RIKEN cDNA 4930573I19 gene	12	112,127,522	112,210,604	4574	in gene	5.00
NM_183016	Cdc42bpb	Cdc42 binding protein kinase beta	12	112,531,190	112,615,570	354	in gene	6.00
NM_009652	Akt1	thymoma viral proto-oncogene 1	12	113,892,032	113,912,401	4401	in gene	6.00

Accession No.	Symbol	Gene Title	Chr	Gene Start	Gene End	Interval Dists to Start	Interval Pos	Avg Peak
XM_001477883, XM_356600, XM_914337	EG382639	predicted gene, EG382639	12	113,917,051	113,920,958	-9051	upstream	6.00
XM_001475946	LOC100041194	similar to KIAA2019 protein	12	114,014,007	114,046,697	6489	in gene	5.00
NM_145450	BC022687	cDNA sequence BC022687	12	114,047,186	114,054,456	-6978	upstream	5.00
XM_001472404	Igh	immunoglobulin heavy chain complex	12	114,496,979	117,248,165	2177813	in gene	5.00
	Ighv5-15	immunoglobulin heavy variable V5-15	12	115,064,857	115,065,152	-5200	upstream	5.00
	Ighv5-16	immunoglobulin heavy variable V5-16	12	115,076,739	115,077,032	6680	downstream	5.00
NM_011215	Ptprn2	protein tyrosine phosphatase, receptor type, N polypeptide 2	12	117,724,306	118,515,266	542142, 732014	in gene, in gene	5.50
NM_145131	Pitrm1	pitrilysin metallepetidase 1	13	6,547,403	6,579,404	28597	in gene	5.00
NM_019703	Pfkp	phosphofructokinase, platelet	13	6,579,120	6,647,970	71970	downstream	5.00
NM_033268	Actn2	actinin alpha 2	13	12,361,693	12,432,999	61735	in gene	8.00
NM_138654	5033411D12Rik	RIKEN cDNA 5033411D12 gene	13	16,949,306	17,786,573	-819	upstream	4.00
NM_025479	2810021B07Rik	RIKEN cDNA 2810021B07 gene	13	17,787,247	17,790,939	145	in gene	4.00
NM_175006	Pou6f2	POU domain, class 6, transcription factor 2	13	18,216,794	18,473,873	257617	downstream	5.00
NM_080288, NM_198093	Elmo1	engulfment and cell motility 1, ced-12 homolog (C. elegans)	13	20,277,294	20,698,394	69202	in gene	6.00
NM_178210	Hist1h4j	histone cluster 1, H4j	13	21,826,965	21,827,276	-693	upstream	5.00
NM_178211	Hist1h4k	histone cluster 1, H4k	13	21,842,063	21,842,374	-2058	upstream	5.00
NM_178183	Hist1h2ak	histone cluster 1, H2ak	13	21,845,304	21,845,696	1264	downstream	5.00
NM_178201	Hist1h2bn	histone cluster 1, H2bn	13	21,845,992	21,846,372	-1560	upstream	5.00
NM_007717	Cmah	cytidine monophospho-N-acetylneuraminic acid hydroxylase	13	24,506,991	24,562,880	64561	downstream	5.00
NM_001080381, NM_029679, NM_178658	6330500D04Rik	RIKEN cDNA 6330500D04 gene	13	24,706,479	24,825,675	24337	in gene	6.00
NM_144536	Cdkal1	CDK5 regulatory subunit associated protein 1-like 1	13	29,417,175	29,947,457	450081, 83681	in gene, in gene	6.50
NM_001013788	EG432743	predicted gene, EG432743	13	31,066,033	31,068,117	12031	downstream	6.00
NM_146041	Gmds	GDP-mannose 4, 6-dehydratase	13	31,911,455	32,430,413	69357	in gene	6.00
	4930482L21Rik	RIKEN cDNA 4930482L21 gene	13	34,088,999	34,089,565	-4675	upstream	5.00
NM_009068	Ripk1	receptor (TNFRSF)-interacting serine-threonine kinase 1	13	34,094,743	34,127,039	-503	upstream	5.00
XM_884663, XM_910732	2310047M15Rik	RIKEN cDNA 2310047M15 gene	13	34,254,833	34,270,041	-6577	upstream	5.00
NM_013830	Prpf4b	PRP4 pre-mRNA processing factor 4 homolog B (yeast)	13	34,967,365	34,994,901	27	in gene	5.00
NM_145367	Txndc5	thioredoxin domain containing 5	13	38,592,144	38,620,329	9	in gene	5.00
XM_001472442, XM_001473940	BC024659	cDNA sequence BC024659	13	41,345,213	41,371,946	499	in gene	5.00
NM_025935	Tbc1d7	TBC1 domain family, member 7	13	43,247,113	43,266,728	29469, -56	downstream, upstream	7.00
NM_021878	Jarid2	jumonji, AT rich interactive domain 2	13	44,826,272	45,016,350	108997	in gene	4.00
XM_001473014	LOC100039502	hypothetical protein LOC100039502	13	45,185,233	45,254,612	75396	downstream	5.00
NM_025508	Gmpr	guanosine monophosphate reductase	13	45,602,838	45,641,750	15914	in gene	7.00
NM_009124	Atxn1	ataxin 1	13	45,650,262	46,060,345	382478, 215129, 178041, 1097	in gene, in gene, in gene, in gene	5.25
XM_001473131, XM_001474547	5033430I15Rik	RIKEN cDNA 5033430I15 gene	13	46,060,622	46,061,876	-1374	upstream	5.00
NM_025900	Dek	DEK oncogene (DNA binding)	13	47,180,154	47,201,479	71	in gene	7.00
NM_146042	Ibrdc2	IBR domain containing 2	13	47,218,089	47,341,845	120407	in gene	6.00
NM_031166	Id4	inhibitor of DNA binding 4	13	48,356,796	48,359,405	-348	upstream	5.00
NM_029361	Wnk2	WNK lysine deficient protein kinase 2	13	49,131,679	49,243,383	29895, 15351	in gene, in gene	4.50
NM_013601	Msx2	homeo box, msh-like 2	13	53,562,250	53,568,149	5333	in gene	6.00
NM_028597	Thoc3	THO complex 3	13	54,560,879	54,570,180	436	in gene	6.00
NM_008011	Fgfr4	fibroblast growth factor receptor 4	13	55,254,179	55,270,120	18861	downstream	6.00
NM_145382	BC021381	cDNA sequence BC021381	13	55,640,680	55,672,481	8801	in gene	5.00
NM_181278	B230219D22Rik	RIKEN cDNA B230219D22 gene	13	55,794,485	55,804,861	14779	downstream	5.00
NM_175150	2310047H23Rik	RIKEN cDNA 2310047H23 gene	13	55,816,011	55,827,588	-6747	upstream	5.00
NM_019568	Cxcl14	chemokine (C-X-C motif) ligand 14	13	56,390,004	56,397,912	8872	downstream	6.00
NM_009369	Tgfbi	transforming growth factor, beta induced	13	56,710,964	56,740,700	-9236, 22956	upstream, in gene	7.50
NM_019832	Gkap1	G kinase anchoring protein 1	13	58,334,712	58,375,549	-611	upstream	5.00
NM_030153	Mak10	MAK10 homolog, amino-acid N-acetyltransferase subunit, (S. cerevisiae)	13	59,686,772	59,736,159	-244	upstream	7.00
NM_177711	4932411G14Rik	RIKEN cDNA 4932411G14 gene	13	59,827,286	59,833,113	-10055	upstream	7.00
NM_025547	Mterfd1	MTERF domain containing 1	13	67,013,062	67,034,003	211	in gene	7.00
NM_008959	Ptdss1	phosphatidylserine synthase 1	13	67,033,766	67,099,337	26	in gene	7.00
NM_198600	Pols	polymerase (DNA directed) sigma	13	69,636,837	69,672,717	-1059	upstream	8.00
XR_035386	A530095I07Rik	RIKEN cDNA A530095I07 gene	13	69,681,924	69,682,901	9125	downstream	8.00
XM_127466, XM_906878	3110006E14Rik	RIKEN cDNA 3110006E14 gene	13	69,841,712	69,900,877	22173	in gene	5.00
NM_146047	Clptm1l	CLPTM1-like	13	73,741,749	73,758,053	-309	upstream	6.00
NM_028186	Nkd2	naked cuticle 2 homolog (Drosophila)	13	73,957,581	73,985,060	2660	in gene	6.00
NM_182839	2900041A09Rik	RIKEN cDNA 2900041A09 gene	13	74,146,926	74,173,201	34994	downstream	6.00
NM_028959	Cep72	centrosomal protein 72	13	74,175,283	74,199,722	17802	in gene	6.00
NM_009644	Ahrr	aryl-hydrocarbon receptor repressor	13	74,348,566	74,429,757	46557	in gene	8.00
NM_029847	Arsk	arylsulfatase K	13	76,198,330	76,236,059	-21	upstream	5.00
NM_001081352	AK129128	cDNA sequence AK129128	13	76,236,182	76,325,431	-102	upstream	5.00
NM_133905	Papd4	PAP associated domain containing 4	13	93,917,355	93,962,238	-178	upstream	6.00

Accession No.	Symbol	Gene Title	Chr	Gene Start	Gene End	Interval Dists to Start	Interval Pos	Avg Peak
NM_021310	Jmy	junction-mediating and regulatory protein	13	94,204,157	94,269,644	172	in gene	7.00
NM_009680	Ap3b1	adaptor-related protein complex 3, beta 1 subunit	13	95,128,986	95,336,270	49270	in gene	6.00
NM_007974	F2rl1	coagulation factor II (thrombin) receptor-like 1	13	96,281,687	96,304,221	-4371	upstream	4.00
NM_010422	Hexb	hexosaminidase B	13	97,946,362	97,968,225	7729	in gene	7.00
NM_012026	Rgnef	Rho-guanine nucleotide exchange factor	13	98,668,550	98,976,120	190104	in gene	6.00
NM_023472	Ankra2	ankyrin repeat, family A (RFXANK-like), 2	13	99,033,197	99,043,868	67	in gene	4.00
NM_001025606	Tmem171	transmembrane protein 171	13	99,456,193	99,464,786	13010	downstream	5.00
NM_008634	Mtap1b	microtubule-associated protein 1 B	13	100,194,396	100,286,495	4991	in gene	6.00
XM_001476235	LOC100040968	similar to CG33003-PA	13	109,906,430	110,125,257	145474, 214898	in gene, in gene	4.50
NM_008046	Fst	follistatin	13	115,242,470	115,248,938	618	in gene	5.00
NM_025341	Abhd6	abhydrolase domain containing 6	14	8,835,416	8,889,069	26472, 51400	in gene, in gene	6.00
NM_010210	Fhit	fragile histidine triad gene	14	10,382,608	11,994,546	380050	in gene	6.00
NM_001033270	Slc4a7	solute carrier family 4, sodium bicarbonate cotransporter, member 7	14	15,535,539	15,632,457	13997	in gene	7.00
NM_025586	Rpl15	ribosomal protein L15	14	19,100,337	19,103,500	-196	upstream	5.00
NM_023526	Nkiras1	NFKB inhibitor interacting Ras-like protein 1	14	19,103,656	19,116,517	40	in gene	5.00
NM_144839	Ube2e2	ubiquitin-conjugating enzyme E2E 2 (UBC4/5 homolog, yeast)	14	19,406,091	19,726,141	249245	in gene	5.00
NM_001038637, NM_010315	Gng2	guanine nucleotide binding protein (G protein), gamma 2 subunit	14	20,691,781	20,796,471	15383	in gene	5.00
NM_018829	Ap3m1	adaptor-related protein complex 3, mu 1 subunit	14	21,854,032	21,871,407	399	in gene	5.00
NM_134079	Adk	adenosine kinase	14	21,871,855	22,267,769	-847	upstream	5.00
NM_145459	Zfp503	zinc finger protein 503	14	22,803,184	22,808,823	1655, -761	in gene, upstream	6.00
NM_010610	Kcnma1	potassium large conductance calcium-activated channel, subfamily M, alpha member 1	14	24,117,983	24,823,427	168387	in gene	8.00
XM_908180, XM_975187	2310047A01Rik	RIKEN cDNA 2310047A01 gene	14	26,531,126	26,588,342	43606, 31494	in gene, in gene	6.00
XM_354798	4933413J09Rik	RIKEN cDNA 4933413J09 gene	14	27,177,039	27,219,942	34033	in gene	5.00
NM_134437	Il17rd	interleukin 17 receptor D	14	27,852,187	27,920,472	19077	in gene	6.00
NM_027871	Arhgef3	Rho guanine nucleotide exchange factor (GEF) 3	14	28,051,225	28,217,090	-7385, 162311	upstream, in gene	7.50
NM_019979	Selk	selenoprotein K	14	30,781,566	30,788,260	450	in gene	6.00
NM_027493	Actr8	ARP8 actin-related protein 8 homolog (S. cerevisiae)	14	30,791,572	30,806,399	-9556	upstream	6.00
NM_001081221	Ercc6	excision repair cross-complementing rodent repair deficiency, complementation group 6	14	33,326,707	33,394,175	57869	in gene	6.00
XM_358452, XM_922412	1810011H11Rik	RIKEN cDNA 1810011H11 gene	14	33,599,149	33,631,154	15475	in gene	4.00
NM_130878	Pcdh21	protocadherin 21	14	37,891,035	37,911,497	7305	in gene	4.00
XM_354808, XM_986046	6330416L11Rik	RIKEN cDNA 6330416L11 gene	14	45,885,001	45,898,928	12807	in gene	5.00
NM_015774	Ero1l	ERO1-like (S. cerevisiae)	14	45,903,996	45,938,202	40394	downstream	5.00
NM_146054	Plekhc1	pleckstrin homology domain containing, family C (with FERM domain) member 1	14	46,078,467	46,149,740	60716	in gene	4.00
XM_001476890	LOC100041724	hypothetical protein LOC100041724	14	46,999,142	47,003,283	7450	downstream	8.00
NM_007554	Bmp4	bone morphogenetic protein 4	14	47,003,195	47,010,274	3682	in gene	8.00
XM_001476910	LOC100041732	hypothetical protein LOC100041732	14	47,005,954	47,008,957	638	in gene	8.00
NM_001037221, NM_028966	Samd4	sterile alpha motif domain containing 4	14	47,502,642	47,721,877	43230	in gene	5.00
NM_172599	D14Ertd436e	DNA segment, Chr 14, ERATO Doi 436, expressed	14	48,160,569	48,188,089	8937	in gene	6.00
XM_890298, XM_906000	EG625730	predicted gene, EG625730	14	48,188,898	48,189,452	-9746	upstream	6.00
NM_033602	Peli2	pellino 2	14	48,740,544	48,880,558	50752	in gene	4.00
NM_172600	6720456H20Rik	RIKEN cDNA 6720456H20 gene	14	49,066,251	49,134,805	-171	upstream	6.00
NM_001081430	Nat12	N-acetyltransferase 12	14	49,791,902	49,810,706	-190	upstream	6.00
NM_001001808	Olfr221	olfactory receptor 221	14	52,654,849	52,655,784	-6232	upstream	5.00
NM_013768	Prmt5	protein arginine N-methyltransferase 5	14	55,126,296	55,136,252	12	in gene	7.00
NM_011186	Psmb5	proteasome (prosome, macropain) subunit, beta type 5	14	55,232,957	55,236,832	-176	upstream	4.00
	Mirn686	microRNA 686	14	55,235,513	55,235,621	-1387	upstream	4.00
NM_175204	5830406J20Rik	RIKEN cDNA 5830406J20 gene	14	55,244,150	55,247,765	-7142	upstream	4.00
NM_001003829, NM_177049	Jph4	junctophilin 4	14	55,725,663	55,735,115	7894	in gene	5.00
NM_024267	Ipo4	importin 4	14	56,244,466	56,254,515	19	in gene	5.00
NM_028780	Tm9sf1	transmembrane 9 superfamily member 1	14	56,254,803	56,262,643	8147	downstream	5.00
XM_001476299, XM_147850	BC030046	cDNA sequence BC030046	14	56,472,853	56,493,578	555	in gene	5.00
NM_030004	Cryl1	crystallin, lambda 1	14	57,893,871	58,017,320	184	in gene	4.00
NM_145463	Tmem46	transmembrane protein 46	14	60,244,145	60,250,492	-1553	upstream	6.00
NM_170591	Nupl1	nucleoporin like 1	14	60,838,305	60,870,215	-137	upstream	6.00
NM_144843	Mtmr6	myotubularin related protein 6	14	60,884,065	60,921,207	35551	in gene	7.00
NM_013869	Tnfrsf19	tumor necrosis factor receptor superfamily, member 19	14	61,582,873	61,665,306	50175, 33946	in gene, in gene	5.50
NM_008715	Ints6	integrator complex subunit 6	14	63,295,162	63,379,949	-291	upstream	6.00
NM_007549	Blk	B lymphoid kinase	14	63,991,674	64,036,024	47832	downstream	5.00

Accession No.	Symbol	Gene Title	Chr	Gene Start	Gene End	Interval Dists to Start	Interval Pos	Avg Peak
NM_153414	D14Ertd231e	DNA segment, Chr 14, ERATO Doi 231, expressed	14	65,568,882	65,658,663	48334	in gene	5.00
NM_009955	Dpysl2	dihydropyrimidinase-like 2	14	67,421,701	67,487,437	-435	upstream	7.00
NM_001034881	EG432870	predicted gene, EG432870	14	67,487,687	67,489,842	185	in gene	7.00
NM_020275	Tnfrsf10b	tumor necrosis factor receptor superfamily, member 10b	14	70,167,336	70,184,218	13912	in gene	6.00
NM_153514	Rhobtb2	Rho-related BTB domain containing 2	14	70,184,799	70,205,277	24029	downstream	6.00
NM_198642	5031414D18Rik	RIKEN cDNA 5031414D18 gene	14	75,402,640	75,452,224	56128	downstream	5.00
XM_001479710	LOC100043189	hypothetical protein LOC100043189	14	76,243,727	76,244,969	265, -663	in gene, upstream	5.00
XM_001471928	LOC100038852	hypothetical protein LOC100038852	14	76,245,138	76,248,105	3401, 2473	downstream, in gene	5.00
NM_009429	Tpt1	tumor protein, translationally-controlled 1	14	76,245,138	76,248,105	-434, 494	upstream, in gene	5.00
XM_894566, XM_987594	2810032E02Rik	RIKEN cDNA 2810032E02 gene	14	76,932,619	76,956,696	184	in gene	6.00
NM_172488	9030625A04Rik	RIKEN cDNA 9030625A04 gene	14	77,424,008	77,436,424	72	in gene	5.00
NM_175369	Ccdc122	coiled-coil domain containing 122	14	77,436,579	77,512,011	-227	upstream	5.00
NM_172813	D230005D02Rik	RIKEN cDNA D230005D02 gene	14	77,556,623	78,121,214	25185	in gene	5.00
NM_001081336	Dgkh	diacylglycerol kinase, eta	14	78,969,416	79,124,896	93648	in gene	4.00
NM_025427	1190002H23Rik	RIKEN cDNA 1190002H23 gene	14	79,688,557	79,701,442	130	in gene	5.00
NM_018765	Wbp4	WW domain binding protein 4	14	79,859,744	79,881,075	163	in gene	8.00
NM_007920	Elf1	E74-like factor 1	14	79,881,001	79,982,283	-89	upstream	8.00
NM_001013753	Pcdh17	protocadherin 17	14	84,845,481	84,934,008	-1337	upstream	6.00
NM_016723	Uchl3	ubiquitin carboxyl-terminal esterase L3 (ubiquitin thiolesterase)	14	102,053,184	102,095,342	78	in gene	4.00
XM_001472577, XM_001480973	Lmo7	LIM domain only 7	14	102,129,129	102,333,927	140791	in gene	5.00
NM_177715	Kctd12	potassium channel tetramerisation domain containing 12	14	103,375,798	103,381,854	30	in gene	8.00
NM_015822	Fbxl3	F-box and leucine-rich repeat protein 3	14	103,479,456	103,498,726	-234	upstream	6.00
NM_026047	2610206B13Rik	RIKEN cDNA 2610206B13 gene	14	104,876,751	104,921,883	-5	upstream	7.00
NM_011897	Spry2	sprouty homolog 2 (Drosophila)	14	106,291,732	106,296,036	-604	upstream	6.00
NM_001033336	Abcc4	ATP-binding cassette, sub-family C (CFTR/MRP), member 4	14	118,881,914	119,105,427	11635	in gene	7.00
NM_008929	Dnajc3a	DnaJ (Hsp40) homolog, subfamily C, member 3A	14	119,337,209	119,379,121	15159	in gene	7.00
NM_015820	Hs6st3	heparan sulfate 6-O-sulfotransferase 3	14	119,537,524	120,269,037	201708	in gene	5.00
NM_175341, NM_207515	Mbnl2	muscleblind-like 2	14	120,674,891	120,830,920	52373, 129693	in gene, in gene	5.00
NM_029556	Clybl	citrate lyase beta like	14	122,580,943	122,801,450	81180	in gene	4.00
NM_010201, NM_207667	Fgf14	fibroblast growth factor 14	14	124,377,129	125,076,349	389885	in gene	5.00
NM_029341	Capsl	calcyphosine-like	15	9,365,783	9,395,790	30841	downstream	6.00
NM_030888	C1qtnf3	C1q and tumor necrosis factor related protein 3	15	10,882,111	10,909,905	29489	downstream	7.00
NM_008537	Amacr	alpha-methylacyl-CoA racemase	15	10,911,511	10,926,379	89	in gene	7.00
NM_025673	Golph3	golgi phosphoprotein 3	15	12,251,251	12,281,022	1581	in gene	9.00
NM_001008420	Cdh12	cadherin 12	15	21,041,207	21,519,288	250985	in gene	7.00
NM_019472	Myo10	myosin X	15	25,552,305	25,743,426	55583, 161823	in gene, in gene	5.00
NM_020332	Ank	progressive ankylosis	15	27,396,432	27,524,660	123472	in gene	7.00
NM_146057	Dap	death-associated protein	15	31,154,180	31,204,090	28, 23708	in gene, in gene	5.00
NM_176073	Pgcp	plasma glutamate carboxypeptidase	15	33,012,953	33,524,258	359	in gene	5.00
NM_016762	Matn2	matrilin 2	15	34,236,436	34,365,997	193	in gene	5.00
NM_054049	Osr2	odd-skipped related 2 (Drosophila)	15	35,225,867	35,233,060	-107	upstream	8.00
XM_981774, XM_986525	Vps13b	vacuolar protein sorting 13B (yeast)	15	35,301,301	35,860,984	401963, 403451	in gene, in gene	5.50
NM_175134	Ankrd46	ankyrin repeat domain 46	15	36,407,423	36,426,506	26	in gene	7.00
NM_025712	4631426E05Rik	RIKEN cDNA 4631426E05 gene	15	36,433,817	36,485,327	58847	downstream	7.00
NM_080640	Baalc	brain and acute leukemia, cytoplasmic	15	38,765,455	38,782,810	4769	in gene	5.00
NM_026149	Nudcd1	NudC domain containing 1	15	44,206,777	44,259,434	-182	upstream	6.00
NM_175009	Eny2	enhancer of yellow 2 homolog (Drosophila)	15	44,259,657	44,269,231	-41	upstream	6.00
NM_134092	Mtbp	Mdm2, transformed 3T3 cell double minute p53 binding protein	15	55,388,963	55,457,978	32765	in gene	5.00
NM_172378	BC026439	cDNA sequence BC026439	15	57,075,326	57,309,180	218492	in gene	7.00
NM_199449	Zhx2	zinc fingers and homeoboxes protein 2	15	57,526,222	57,671,387	78162, 132733	in gene, in gene	4.50
NM_175226	Rnf139	ring finger protein 139	15	58,720,853	58,732,409	14230	downstream	4.00
NM_175151	Tatdn1	TatD DNase domain containing 1	15	58,721,708	58,765,285	30202	in gene	4.00
NM_144800	Mtss1	metastasis suppressor 1	15	58,774,802	58,913,544	129544	in gene	6.00
NR_003368	Pvt1	plasmacytoma variant translocation 1	15	61,869,542	62,082,530	20522, 174506	in gene, in gene	5.00
XM_001475406	LOC100040584	hypothetical protein LOC100040584	15	62,042,972	62,044,802	754	in gene	5.00
NM_009623	Adcy8	adenylate cyclase 8	15	64,530,597	64,753,858	130642	in gene	6.00
NM_009177	St3gal1	ST3 beta-galactoside alpha-2,3-sialyltransferase 1	15	66,934,437	67,008,444	252	in gene	7.00
XM_907370, XM_981889	Col22a1	collagen, type XXII, alpha 1	15	71,628,906	71,864,639	239	in gene	5.00
NM_001033219	Slc45a4	solute carrier family 45, member 4	15	73,411,987	73,455,174	1350	in gene	5.00
XM_128161, XM_909411	1700016M24Rik	RIKEN cDNA 1700016M24 gene	15	74,436,458	74,466,748	19164	in gene	6.00
NM_008415	Jrk	jerky	15	74,532,842	74,539,727	-1841	upstream	5.00
XR_035258, XR_035306	4933427E11Rik	RIKEN cDNA 4933427E11 gene	15	74,539,591	74,540,804	1977	downstream	5.00
NM_028216	Psca	prostate stem cell antigen	15	74,545,269	74,547,499	-3701	upstream	5.00

Accession No.	Symbol	Gene Title	Chr	Gene Start	Gene End	Interval Dists to Start	Interval Pos	Avg Peak
NM_198607	4930572J05Rik	RIKEN cDNA 4930572J05 gene	15	74,551,664	74,554,803	-10096	upstream	5.00
NM_026730	Gpihbp1	GPI-anchored HDL-binding protein 1	15	75,427,088	75,428,643	2245	downstream	5.00
XM_001475872, XM_001481291	2810039B14Rik	RIKEN cDNA 2810039B14 gene	15	75,450,171	75,477,866	22405	in gene	7.00
NM_008164	Rhpn1	rhophilin, Rho GTPase binding protein 1	15	75,534,824	75,544,655	9416	in gene	7.00
NM_177922	Mapk15	mitogen-activated protein kinase 15	15	75,824,199	75,829,583	345	in gene	5.00
NM_134087	AA409316	expressed sequence AA409316	15	75,831,533	75,844,766	20222	downstream	5.00
XM_001476072	LOC100041269	similar to sphingomyelin phosphodiesterase 3, neutral membrane	15	76,124,697	76,126,431	10039	downstream	5.00
NM_153122	Oplah	5-oxoprolinase (ATP-hydrolysing)	15	76,127,033	76,137,675	2939	in gene	5.00
NM_058214	Recql4	RecQ protein-like 4	15	76,534,171	76,540,906	-4310	upstream	6.00
NM_145916	Zfp7	zinc finger protein 7	15	76,709,689	76,722,825	20615	downstream	7.00
NM_025536	Commd5	COMM domain containing 5	15	76,730,371	76,731,727	-67	upstream	7.00
NM_012053	Rpl8	ribosomal protein L8	15	76,734,529	76,736,744	-4225	upstream	7.00
XM_001478851	LOC100042318	similar to thymosin, beta 10	15	77,200,578	77,200,712	3336	downstream	5.00
NM_013645	Pvalb	parvalbumin	15	78,021,547	78,036,781	19469	downstream	6.00
NM_008595	Mfng	manic fringe homolog (Drosophila)	15	78,586,313	78,603,875	-2301	upstream	6.00
NM_001024716, NM_001039155, NM_001039156, NM_138579	Triobp	TRIO and F-actin binding protein	15	78,778,154	78,836,299	34806	in gene	7.00
NM_008197	H1f0	H1 histone family, member 0	15	78,858,642	78,860,934	-4370	upstream	7.00
NM_013847	Gcat	glycine C-acetyltransferase (2-amino-3-ketobutyrate-coenzyme A ligase)	15	78,861,350	78,868,784	-7078	upstream	7.00
NM_016915	Pla2g6	phospholipase A2, group VI	15	79,116,658	79,158,598	2022	in gene	5.00
NM_010755	Maff	v-maf musculoaponeurotic fibrosarcoma oncogene family, protein F (avian)	15	79,178,108	79,189,506	260	in gene	5.00
NM_013767	Csnk1e	casein kinase 1, epsilon	15	79,248,286	79,272,437	1013	in gene	5.00
NM_001040187, NM_152806, NM_199079, NM_199080	Ddx17	DEAD (Asp-Glu-Ala-Asp) box polypeptide 17	15	79,358,126	79,377,171	67	in gene	5.00
NM_030689	Nptxr	neuronal pentraxin receptor	15	79,616,781	79,635,139	8995	in gene	5.00
NM_030255	Apobec3	apolipoprotein B editing complex 3	15	79,722,888	79,738,516	-9032	upstream	10.00
NM_009303, NM_207708	Syngr1	synaptogyrin 1	15	79,921,764	79,949,931	25516	in gene	6.00
NM_178719	Smcr7l	Smith-Magenis syndrome chromosome region, candidate 7-like (human)	15	80,064,550	80,083,154	20762	downstream	7.00
NM_009716	Atf4	activating transcription factor 4	15	80,085,614	80,087,971	-302	upstream	7.00
NM_175109	Rps19bp1	ribosomal protein S19 binding protein 1	15	80,091,044	80,094,736	9424	downstream	7.00
NM_017376, NM_153484	Tef	thyrotroph embryonic factor	15	81,633,247	81,657,293	8673	in gene	8.00
XM_001474248, XM_001479829	Xrcc6	X-ray repair complementing defective repair in Chinese hamster cells 6	15	81,846,799	81,870,521	30041	downstream	4.00
NM_011482	Nhp2l1	NHP2 non-histone chromosome protein 2-like 1 (S. cerevisiae)	15	81,871,775	81,878,028	1188	in gene	4.00
NM_022723	Scube1	signal peptide, CUB domain, EGF-like 1	15	83,433,013	83,555,451	85611, 73179, 58267, 38075	in gene, in gene, in gene, in gene	7.50
NM_029946	4931407K02Rik	RIKEN cDNA 4931407K02 gene	15	83,697,142	83,895,779	67683	in gene	6.00
NM_013873	Sult4a1	sulfotransferase family 4A, member 1	15	83,906,527	83,936,184	16040	in gene	5.00
XM_128169, XM_901935	1810041L15Rik	RIKEN cDNA 1810041L15 gene	15	84,209,633	84,277,527	36791	in gene	5.00
NM_146061	Arhgap8	Rho GTPase activating protein 8	15	84,511,428	84,534,103	-4324	upstream	7.00
NM_028455	3110043J09Rik	RIKEN cDNA 3110043J09 gene	15	84,550,532	84,602,631	1004, 9703, 37916	in gene, in gene, in gene	5.33
	Mirnlet7c-2	microRNA let7c-2	15	85,537,032	85,537,126	-7624	upstream	5.00
	Mirnlet7b	microRNA let7b	15	85,537,748	85,537,832	-8340	upstream	5.00
NM_011144	Ppara	peroxisome proliferator activated receptor alpha	15	85,566,206	85,633,249	75938	downstream	7.00
NM_197998	2210021J22Rik	RIKEN cDNA 2210021J22 gene	15	85,637,402	85,642,089	-55	upstream	7.00
NM_011105	Pkdrej	polycystic kidney disease (polycystin) and REJ (sperm receptor for egg jelly, sea urchin homolog)-like	15	85,645,106	85,652,163	10019	downstream	7.00
NM_001033337	AW124722	expressed sequence AW124722	15	85,662,774	85,688,163	10666	in gene	5.00
NM_145476	Tbc1d22a	TBC1 domain family, member 22a	15	86,044,889	86,328,933	191543, 259255	in gene, in gene	8.00
NM_027081	1700027J05Rik	RIKEN cDNA 1700027J05 gene	15	89,012,643	89,026,905	345	in gene	6.00
NM_016705	Kif21a	kinesin family member 21A	15	90,764,814	90,880,242	2	in gene	5.00
NM_011994	Abcd2	ATP-binding cassette, sub-family D (ALD), member 2	15	90,976,302	91,022,238	1550	in gene	6.00
NM_026025	Zcrb1	zinc finger CCHC-type and RNA binding motif 1	15	93,216,544	93,228,721	17	in gene	7.00
NM_001083114, NM_146062, NM_175363	Pphln1	periphilin 1	15	93,228,781	93,322,344	-77	upstream	7.00
NM_008971	Twf1	twinfilin, actin-binding protein, homolog 1 (Drosophila)	15	94,408,379	94,420,255	86	in gene	4.00
NM_175344	Tmem16f	transmembrane protein 16F	15	95,621,274	95,805,205	17, 80742	in gene, in gene	6.00
NM_175121	Slc38a2	solute carrier family 38, member 2	15	96,517,823	96,530,129	977	in gene	4.00
NM_172293	BC038822	cDNA sequence BC038822	15	97,077,538	97,216,107	29534	in gene	5.00

Accession No.	Symbol	Gene Title	Chr	Gene Start	Gene End	Interval Dists to Start	Interval Pos	Avg Peak
NM_028003	D15Ertd682e	DNA segment, Chr 15, ERATO Doi 682, expressed	15	97,505,536	97,536,253	25453	in gene	6.00
NM_144850	Rapgef3	Rap guanine nucleotide exchange factor (GEF) 3	15	97,575,384	97,598,103	20695	in gene	5.00
NM_019572	Hdac7a	histone deacetylase 7A	15	97,623,112	97,662,102	21622, 10486	in gene, in gene	5.50
NM_177716	AI836003	expressed sequence AI836003	15	97,998,091	98,000,565	-6203	upstream	5.00
NM_001044741, NM_007581	Cacnb3	calcium channel, voltage-dependent, beta 3 subunit	15	98,462,651	98,474,961	2298, 13829	in gene, downstream	5.50
NM_013639	Prph1	peripherin 1	15	98,885,638	98,889,101	1242	in gene	6.00
NM_028015	Lass5	longevity assurance homolog 5 (S. cerevisiae)	15	99,566,023	99,602,946	162	in gene	7.00
NM_023063	Lima1	LIM domain and actin binding 1	15	99,608,939	99,650,503	47719	downstream	7.00
NM_027011	Krt5	keratin 5	15	101,537,501	101,543,322	-5750	upstream	6.00
NM_031170	Krt8	keratin 8	15	101,827,142	101,834,773	1397	in gene	7.00
NM_008344	Igfbp6	insulin-like growth factor binding protein 6	15	101,974,793	101,979,943	6759	downstream	6.00
NM_146064	Soat2	sterol O-acyltransferase 2	15	101,981,006	101,993,867	546	in gene	6.00
NM_144942	Csad	cysteine sulfinic acid decarboxylase	15	102,007,429	102,019,474	-1582	upstream	5.00
NM_134100	D15Mgi27	DNA Segment, Chr 15, Mouse Genome Informatics 27	15	102,109,885	102,112,175	-13	upstream	6.00
NM_013672	Sp1	trans-acting transcription factor 1	15	102,236,747	102,266,835	8181	in gene	5.00
NM_146065	Atf7	activating transcription factor 7	15	102,367,074	102,455,852	332	in gene	6.00
NM_026192	Calcoco1	calcium binding and coiled coil domain 1	15	102,537,210	102,552,500	5892	in gene	4.00
NM_001024842	Hoxc11	homeo box C11	15	102,784,957	102,787,132	579	in gene	5.00
NM_010462	Hoxc10	homeo box C10	15	102,797,296	102,802,152	2560, 14288, 14896	in gene, downstream, downstream	6.00
	Mirn196a-2	microRNA 196a-2	15	102,803,780	102,803,864	-3924, 7804, 8412	upstream, downstream, downstream	6.00
NM_008272	Hoxc9	homeo box C9	15	102,807,463	102,814,875	-7607, 4121, 4729	upstream, in gene, in gene	6.00
XM_001479425	LOC100043060	similar to Unknown (protein for IMAGE:1366468)	15	102,807,604	102,808,618	8762, -2966, -3574	downstream, upstream, upstream	6.00
NM_010465	Hoxc6	homeo box C6	15	102,839,993	102,842,312	-489	upstream	5.00
NM_175730	Hoxc5	homeo box C5	15	102,844,439	102,847,860	-4935	upstream	5.00
	Mirn615	microRNA 615	15	102,845,340	102,845,431	-5836	upstream	5.00
NM_013866	Zfp385	zinc finger protein 385	15	103,144,326	103,170,517	5861, 613	in gene, in gene	4.50
NM_010577	Itga5	integrin alpha 5 (fibronectin receptor alpha)	15	103,174,717	103,197,194	32538, 27290	downstream, downstream	4.50
NM_029090	1200013P24Rik	RIKEN cDNA 1200013P24 gene	16	3,884,619	3,904,781	133	in gene	8.00
NM_026508	Trap1	TNF receptor-associated protein 1	16	4,039,977	4,077,810	-30	upstream	5.00
NM_009624	Adcy9	adenylate cyclase 9	16	4,287,545	4,419,587	81235	in gene	5.00
XM_906811, XM_974329	4930562C15Rik	RIKEN cDNA 4930562C15 gene	16	4,835,416	4,867,691	31976	in gene	7.00
XM_622593, XM_974374	1500031H01Rik	RIKEN cDNA 1500031H01 gene	16	4,874,777	4,880,315	12923, 411	downstream, in gene	6.00
NM_029657	Mgrn1	mahogunin, ring finger 1	16	4,886,252	4,938,296	-6348, 19268	upstream, in gene	5.00
NM_025821	Carhsp1	calcium regulated heat stable protein 1	16	8,658,680	8,672,246	1814	in gene	8.00
XM_489506, XM_622598	EG436336	predicted gene, EG436336	16	8,672,327	8,684,000	-1895	upstream	8.00
XM_975517	LOC665231	hypothetical protein LOC665231	16	8,829,424	8,830,328	-48	upstream	5.00
XM_001480138, XM_907664	1810013L24Rik	RIKEN cDNA 1810013L24 gene	16	8,830,188	8,859,017	-812	upstream	5.00
NM_007929	Emp2	epithelial membrane protein 2	16	10,281,842	10,314,061	-1219	upstream	6.00
NM_021428	Dexi	dexamethasone-induced transcript	16	10,530,300	10,543,147	-4597	upstream	7.00
NM_177562	4932416N17Rik	RIKEN cDNA 4932416N17 gene	16	10,545,480	10,744,964	2264	in gene	7.00
NM_009223	Snn	stannin	16	11,066,454	11,075,071	42	in gene	11.00
NM_029582, NM_134105	Txndc11	thioredoxin domain containing 11	16	11,075,004	11,134,625	68129, 20513	downstream, in gene	9.50
NM_146068	2310008H04Rik	RIKEN cDNA 2310008H04 gene	16	15,889,320	16,146,926	110	in gene	4.00
NM_001001983	Pik4ca	phosphatidylinositol 4-kinase, catalytic, alpha polypeptide	16	17,280,445	17,406,407	487	in gene	7.00
NM_023348	Snap29	synaptosomal-associated protein	16	17,406,093	17,430,919	-173	upstream	7.00
XM_001478907	LOC100042830	hypothetical protein LOC100042830	16	17,759,358	17,761,950	418	in gene	5.00
NM_145479	Klhl22	kelch-like 22 (Drosophila)	16	17,759,764	17,793,473	12	in gene	5.00
NM_033474	Arvcf	armadillo repeat gene deleted in velo-cardio-facial syndrome	16	18,348,367	18,407,169	33617, 44401	in gene, in gene	6.50
NM_001001999, NM_010327	Gp1bb	glycoprotein Ib, beta polypeptide	16	18,620,412	18,622,496	-6144	upstream	4.00
NM_213614	Sept5	septin 5	16	18,621,904	18,630,031	1391	in gene	4.00
NM_009862	Cdc45l	cell division cycle 45 homolog (S. cerevisiae)-like	16	18,780,545	18,812,065	225	in gene	5.00
NM_011672	Ufd1l	ubiquitin fusion degradation 1 like	16	18,812,419	18,835,348	-579	upstream	5.00
NM_010922	Mrpl40	mitochondrial ribosomal protein L40	16	18,872,310	18,876,730	58	in gene	6.00
NM_010435	Hira	histone cell cycle regulation defective homolog A (S. cerevisiae)	16	18,876,843	18,970,402	-171, 78656	upstream, in gene	5.50
XM_001479111	LOC100042938	hypothetical protein LOC100042938	16	20,097,560	20,099,755	-56, 552	upstream, in gene	7.00
NM_029436	Klhl24	kelch-like 24 (Drosophila)	16	20,097,627	20,127,817	-123, 485	upstream, in gene	7.00
NM_134101	Psmd2	proteasome (prosome, macropain) 26S subunit, non-ATPase, 2	16	20,651,768	20,663,487	6744	in gene	7.00
NM_009379	Thpo	thrombopoietin	16	20,724,527	20,734,584	-5544	upstream	5.00
NM_009893	Chrd	chordin	16	20,733,200	20,742,455	6928	in gene	5.00

Accession No.	Symbol	Gene Title	Chr	Gene Start	Gene End	Interval Dists to Start	Interval Pos	Avg Peak
NM_183029	Igf2bp2	insulin-like growth factor 2 mRNA binding protein 2	16	22,059,128	22,163,092	40788	in gene	4.00
NM_178665	Lpp	LIM domain containing preferred translocation partner in lipoma	16	24,393,576	24,981,116	47944, 258776	in gene, in gene	6.50
NM_001025615, NM_026202	Ccdc50	coiled-coil domain containing 50	16	27,389,069	27,448,909	12483	in gene	6.00
XM_001479023, XM_001480951	1700025H01Rik	RIKEN cDNA 1700025H01 gene	16	30,195,157	30,200,385	-6971	upstream	5.00
XM_001480958, XM_001480961, XM_893953	Atp13a3	ATPase type 13A3	16	30,312,488	30,388,616	-376	upstream	5.00
NM_178069	Lsg1	large subunit GTPase 1 homolog (S. cerevisiae)	16	30,560,580	30,587,669	36341	downstream	6.00
NM_198626	AI480653	expressed sequence AI480653	16	30,955,716	31,081,466	97882	in gene	6.00
XM_001001008, XM_986694	Gm536	gene model 536, (NCBI)	16	31,618,219	31,639,065	-107	upstream	7.00
NM_172822	Pigz	phosphatidylinositol glycan anchor biosynthesis, class Z	16	31,934,040	31,946,132	-856, 14472	upstream, downstream	7.00
XR_035420	0610012G03Rik	RIKEN cDNA 0610012G03 gene	16	31,947,137	31,948,595	83	in gene	9.00
NM_026554	Ncbp2	nuclear cap binding protein subunit 2	16	31,948,725	31,958,267	-213	upstream	9.00
NM_177103	Senp5	SUMO/sentrin specific peptidase 5	16	31,962,639	32,003,257	121	in gene	6.00
XM_619387, XM_914704	Ubxd7	UBX domain containing 7	16	32,330,782	32,387,978	5650	in gene	8.00
NM_025329	0610012D17Rik	RIKEN cDNA 0610012D17 gene	16	32,419,830	32,429,184	11098	downstream	6.00
NM_009981	Pcyt1a	phosphate cytidylyltransferase 1, choline, alpha isoform	16	32,431,082	32,472,101	-154	upstream	6.00
NM_016788	Tnk2	tyrosine kinase, non-receptor, 2	16	32,644,947	32,683,579	1293	in gene	7.00
NM_172615	1700021K19Rik	RIKEN cDNA 1700021K19 gene	16	32,821,822	32,868,425	18153	in gene	4.00
NM_172934	4632417D23Rik	RIKEN cDNA 4632417D23 gene	16	33,684,552	33,722,999	2376	in gene	6.00
NM_010739	Muc13	mucin 13, epithelial transmembrane	16	33,794,123	33,820,013	28517	downstream	6.00
NM_010580	Itgb5	integrin beta 5	16	33,829,845	33,949,232	-7205	upstream	6.00
NM_009471	Umps	uridine monophosphate synthetase	16	33,955,098	33,967,089	-495	upstream	6.00
NM_023587	Ptplb	protein tyrosine phosphatase-like (proline instead of catalytic arginine), member b	16	35,022,507	35,109,261	-59	upstream	7.00
NM_001012765	Adcy5	adenylate cyclase 5	16	35,154,963	35,305,821	56957	in gene	5.00
NM_028295	Pdia5	protein disulfide isomerase associated 5	16	35,397,398	35,490,959	51823	in gene	7.00
NM_153550	Dirc2	disrupted in renal carcinoma 2 (human)	16	35,694,989	35,769,442	18770	in gene	5.00
NM_026439	Ccdc80	coiled-coil domain containing 80	16	45,094,166	45,128,037	-22, 2218	upstream, in gene	5.00
NM_172511	Abhd10	abhydrolase domain containing 10	16	45,730,510	45,743,017	105	in gene	5.00
NM_153412	Phldb2	pleckstrin homology-like domain, family B, member 2	16	45,746,356	45,844,491	101579	downstream	5.00
XM_001481102, XM_489641	Plcxd2	phosphatidylinositol-specific phospholipase C, X domain containing 2	16	45,959,376	46,010,526	-1218	upstream	9.00
NM_001033238	Cblb	Casitas B-lineage lymphoma b	16	52,031,662	52,208,160	151202	in gene	7.00
XM_001003685, XM_925022	Gm1752	gene model 1752, (NCBI)	16	55,843,294	55,895,398	214	in gene	6.00
NM_028815	Lrriq2	leucine-rich repeats and IQ motif containing 2	16	55,902,376	55,934,961	39777	downstream	6.00
XM_001479180, XM_001480210	LOC629242	hypothetical protein LOC629242	16	55,961,428	55,965,167	13212	downstream	5.00
NM_024218	Rpl24	ribosomal protein L24	16	55,966,506	55,971,547	8134	downstream	5.00
XM_001480244, XM_001481121	Zbtb11	zinc finger and BTB domain containing 11	16	55,973,929	56,009,026	711	in gene	5.00
NM_026254	Tbc1d23	TBC1 domain family, member 23	16	57,168,975	57,231,579	14011	in gene	6.00
NM_025599	2610528E23Rik	RIKEN cDNA 2610528E23 gene	16	57,302,113	57,606,956	168588, 150956	in gene, in gene	4.50
NM_001040397	4631422O05Rik	RIKEN cDNA 4631422O05 gene	16	57,353,390	57,515,803	84978, 102610	in gene, in gene	4.50
NM_175549	Robo2	roundabout homolog 2 (Drosophila)	16	73,892,551	74,411,157	449045	in gene	7.00
NM_173440	Nrip1	nuclear receptor interacting protein 1	16	76,291,107	76,373,294	58654	in gene	6.00
NM_001081068	Zfp294	zinc finger protein 294	16	87,376,896	87,432,851	29763	in gene	6.00
NM_015755	Hunk	hormonally upregulated Neu-associated kinase	16	90,386,642	90,499,798	38574	in gene	6.00
NM_027627	4931408A02Rik	RIKEN cDNA 4931408A02 gene	16	90,831,358	90,904,940	51682	in gene	9.00
NM_080456	Mrps6	mitochondrial ribosomal protein S6	16	92,058,581	92,112,472	20107	in gene	5.00
NM_017391	Slc5a3	solute carrier family 5 (inositol transporters), member 3	16	92,077,302	92,079,458	1386	in gene	5.00
NM_001081549, NM_019466	Dscr1	Down syndrome critical region homolog 1 (human)	16	92,392,198	92,466,391	64959, 30012	in gene, in gene	5.00
XM_001473308, XM_001475819	2410124H12Rik	RIKEN cDNA 2410124H12 gene	16	92,478,987	92,497,610	19413, 19829, 22613	downstream, downstream, downstream	5.33
NM_172469	Clic6	chloride intracellular channel 6	16	92,498,392	92,541,486	8, 424, 3208	in gene, in gene, in gene	5.33
NM_026700, NM_027293	Dopey2	dopey family member 2	16	93,712,152	93,810,833	69256, 96248	in gene, in gene	8.00
NM_133659	Erg	avian erythroblastosis virus E-26 (v-ets) oncogene related	16	95,581,811	95,751,972	34324	in gene	6.00
NM_145125	Brwd1	bromodomain and WD repeat domain containing 1	16	96,214,054	96,304,033	63673	in gene	4.00
NM_008251	Hmgn1	high mobility group nucleosomal binding domain 1	16	96,343,195	96,349,332	-620, -8300	upstream, upstream	5.50
NM_207301	Wrb	tryptophan rich basic protein	16	96,367,026	96,379,459	-9394	upstream	5.00

Accession No.	Symbol	Gene Title	Chr	Gene Start	Gene End	Interval Dists to Start	Interval Pos	Avg Peak
NM_015775	Tmprss2	transmembrane protease, serine 2	16	97,786,289	97,832,802	17426	in gene	5.00
XM_619423, XM_918778	Rbm16	RNA binding motif protein 16	17	3,104,693	3,198,859	10379	in gene	6.00
NM_146073	Zdhhc14	zinc finger, DHHC domain containing 14	17	5,492,600	5,753,891	104104, 230488	in gene, in gene	6.00
NM_011523	Synj2	synaptojanin 2	17	5,975,641	6,044,290	1079	in gene	6.00
NM_010515	Igf2r	insulin-like growth factor 2 receptor	17	12,875,272	12,962,572	64940	in gene	6.00
NM_001034891, NM_001039552	9030025P20Rik	RIKEN cDNA 9030025P20 gene	17	15,158,724	15,201,187	34204	in gene	6.00
XM_974092	5830433I10Rik	RIKEN cDNA 5830433I10 gene	17	15,840,976	15,842,227	-589	upstream	5.00
NM_007690	Chd1	chromodomain helicase DNA binding protein 1	17	15,842,254	15,907,328	562	in gene	5.00
NM_172827	Lnpep	leucyl/cystinyl aminopeptidase	17	17,664,687	17,761,453	-211	upstream	8.00
XM_891564	EG626942	predicted gene, EG626942	17	17,847,275	17,964,398	116565	in gene	8.00
	Mirn99b	microRNA 99b	17	17,967,151	17,967,220	-3311, 8113	upstream, downstream	7.50
	Mirnlet7e	microRNA let7e	17	17,967,315	17,967,407	-3475, 7949	upstream, downstream	7.50
	Mirn125a	microRNA 125a	17	17,967,775	17,967,842	-3935, 7489	upstream, downstream	7.50
NM_029205	4930546H06Rik	RIKEN cDNA 4930546H06 gene	17	17,968,028	17,979,973	-4188, 7236	upstream, in gene	7.50
NM_008215	Has1	hyaluronan synthase1	17	17,980,287	17,992,150	16886	downstream	7.00
NM_011747	Zfp13	zinc finger protein 13	17	23,712,819	23,736,454	6614	in gene	7.00
NM_001033425	Zscan10	zinc finger and SCAN domains 10	17	23,737,823	23,747,979	-7983	upstream	7.00
NM_175229	Srrm2	serine/arginine repetitive matrix 2	17	23,940,154	23,961,708	26150	downstream	6.00
NM_026305	Tceb2	transcription elongation factor B (SIII), polypeptide 2	17	23,961,707	23,966,076	-228	upstream	6.00
NM_001081399	Prss33	protease, serine, 33	17	23,970,832	23,973,032	6728	downstream	6.00
NM_027644	4931440B09Rik	RIKEN cDNA 4931440B09 gene	17	23,973,752	23,981,123	14819	downstream	6.00
NM_133731	Prss22	protease, serine, 22	17	24,130,501	24,135,050	7042	downstream	5.00
NM_175440	Prss27	protease, serine 27	17	24,175,210	24,182,916	6742	in gene	5.00
NM_027008	Kctd5	potassium channel tetramerisation domain containing 5	17	24,184,705	24,210,404	28452	downstream	5.00
NM_001080773, NM_011062	Pdpk1	3-phosphoinositide dependent protein kinase-1	17	24,210,647	24,278,556	140	in gene	8.00
NM_013630	Pkd1	polycystic kidney disease 1 homolog	17	24,686,895	24,733,459	326, 38177, 44634, 50697	in gene, in gene, in gene, downstream	5.00
NM_001039363, NM_011647	Tsc2	tuberous sclerosis 2	17	24,732,882	24,769,574	44502, 38045, 31982, -5925	downstream, downstream, in gene, upstream	4.50
NM_008743	Nthl1	nth (endonuclease III)-like 1 (E.coli)	17	24,769,655	24,775,782	5844	in gene	5.00
NM_023055, NM_023449	Slc9a3r2	solute carrier family 9 (sodium/hydrogen exchanger), isoform 3 regulator 2	17	24,776,233	24,787,223	11724, -1449	downstream, upstream	5.00
XM_001478349, XM_993080	Npw	neuropeptide W	17	24,794,275	24,796,344	7672	downstream	5.00
NM_134126	Ift140	intraflagellar transport 140 homolog (Chlamydomonas)	17	25,153,668	25,236,431	61228	in gene	6.00
NM_001001183	BC054438	cDNA sequence BC054438	17	25,194,647	25,218,059	3163	in gene	6.00
NM_027880	Telo2	TEL2, telomere maintenance 2, homolog (S. cerevisiae)	17	25,236,522	25,252,720	-7904	upstream	5.00
XM_128459, XM_914629	1110018H23Rik	RIKEN cDNA 1110018H23 gene	17	25,257,705	25,262,213	2919	in gene	5.00
NM_011930	Clcn7	chloride channel 7	17	25,270,339	25,299,044	-9715	upstream	5.00
NM_028789	Unkl	unkempt-like (Drosophila)	17	25,359,527	25,371,387	12057	downstream	6.00
NM_172529	Gnptg	N-acetylglucosamine-1-phosphotransferase, gamma subunit	17	25,371,262	25,377,061	5477	in gene	6.00
NM_026676	0610007P22Rik	RIKEN cDNA 0610007P22 gene	17	25,377,144	25,379,740	-5560	upstream	6.00
NM_029624	Tmem112	transmembrane protein 112	17	25,716,134	25,799,668	77050	in gene	6.00
NM_173741	Wdr24	WD repeat domain 24	17	25,960,661	25,965,669	9259	downstream	9.00
NM_028101	2610003J06Rik	RIKEN cDNA 2610003J06 gene	17	25,965,992	25,968,711	3928	downstream	9.00
NM_019719	Stub1	STIP1 homology and U-Box containing protein 1	17	25,967,581	25,970,306	386	in gene	9.00
NM_144816	Rhbdl1	rhomboid, veinlet-like 1 (Drosophila)	17	25,971,410	25,974,072	4152	downstream	9.00
NM_145999	Rhot2	ras homolog gene family, member T2	17	25,975,783	25,981,796	11876	downstream	9.00
NM_139154	Rab40c	Rab40c, member RAS oncogene family	17	26,019,059	26,056,647	-169	upstream	9.00
NM_011822	Pigq	phosphatidylinositol glycan anchor biosynthesis, class Q	17	26,063,369	26,078,907	22091	downstream	9.00
NM_021793	Tmem8	transmembrane protein 8 (five membrane-spanning domains)	17	26,250,262	26,260,199	6837	in gene	5.00
NM_024227	Mrpl28	mitochondrial ribosomal protein L28	17	26,260,477	26,263,558	-3378	upstream	5.00
NM_026170	Ergic1	endoplasmic reticulum-golgi intermediate compartment (ERGIC) 1	17	26,698,457	26,793,879	41959	in gene	8.00
NM_008700	Nkx2-5	NK2 transcription factor related, locus 5 (Drosophila)	17	26,975,610	26,978,510	3294	downstream	6.00
NM_011861, NM_178365	Pacsin1	protein kinase C and casein kinase substrate in neurons 1	17	27,792,627	27,848,051	61517	downstream	5.00
NM_013891	Spdef	SAM pointed domain containing ets transcription factor	17	27,851,395	27,865,865	11721	in gene	5.00
NM_001033279, NM_001044719	D17Wsu92e	DNA segment, Chr 17, Wayne State University 92, expressed	17	27,888,180	27,957,487	34463	in gene	7.00
NM_181413	Anks1	ankyrin repeat and SAM domain containing 1	17	28,046,285	28,199,582	59235, 120115	in gene, in gene	6.00
NM_011566	Tead3	TEA domain family member 3	17	28,468,620	28,487,545	-4071	upstream	6.00

Accession No.	Symbol	Gene Title	Chr	Gene Start	Gene End	Interval Dists to Start	Interval Pos	Avg Peak
NM_021478	Tulp1	tubby like protein 1	17	28,488,464	28,502,088	10472	in gene	6.00
XM_001478871	LOC100042815	hypothetical protein LOC100042815	17	29,230,361	29,231,484	-9849	upstream	5.00
NM_007669	Cdkn1a	cyclin-dependent kinase inhibitor 1A (P21)	17	29,230,720	29,237,667	-10208	upstream	5.00
NM_026845	Ppil1	peptidylprolyl isomerase (cyclophilin)-like 1	17	29,387,780	29,400,916	-4924	upstream	7.00
NM_030561	BC004004	cDNA sequence BC004004	17	29,405,924	29,439,826	-84	upstream	7.00
NM_019880	Mtch1	mitochondrial carrier homolog 1 (C. elegans)	17	29,469,021	29,484,849	-7754	upstream	5.00
NM_013710	Fgd2	FYVE, RhoGEF and PH domain containing 2	17	29,497,859	29,516,498	-5256	upstream	5.00
NM_198647	Tbc1d22b	TBC1 domain family, member 22B	17	29,686,747	29,743,753	44997	in gene	5.00
NM_028791	1300018I05Rik	RIKEN cDNA 1300018I05 gene	17	29,797,546	29,840,304	-10	upstream	6.00
NM_001081160	Mdga1	MAM domain containing glycosylphosphatidylinositol anchor 1	17	29,964,903	30,024,827	44955	in gene	5.00
NM_009593	Abcg1	ATP-binding cassette, sub-family G (WHITE), member 1	17	31,187,732	31,252,722	29132	in gene	5.00
XM_987982	EG667056	predicted gene, EG667056	17	31,430,697	31,436,767	-9473	upstream	6.00
NM_153062	Slc37a1	solute carrier family 37 (glycerol-3-phosphate transporter), member 1	17	31,432,428	31,487,654	13812	in gene	6.00
NM_016670	Pknox1	Pbx/knotted 1 homeobox	17	31,720,641	31,744,629	32591	downstream	4.00
NM_144855, NM_178224	Cbs	cystathionine beta-synthase	17	31,749,589	31,774,086	20854, 11942	in gene, in gene	4.00
XM_484622, XM_904809	Hsf2bp	heat shock transcription factor 2 binding protein	17	32,081,714	32,171,453	97373	downstream	6.00
NM_028244	Rrp1b	ribosomal RNA processing 1 homolog B (S. cerevisiae)	17	32,173,107	32,197,550	26765	downstream	8.00
NM_008716	Notch3	Notch gene homolog 3 (Drosophila)	17	32,257,838	32,303,797	51365	downstream	5.00
NM_172458	9030612M13Rik	RIKEN cDNA 9030612M13 gene	17	32,902,441	32,925,232	48	in gene	9.00
NM_183177	Zfp811	zinc finger protein 811	17	32,933,934	32,946,798	21614	downstream	9.00
NM_013585	Psmb9	proteosome (prosome, macropain) subunit, beta type 9 (large multifunctional peptidase 2)	17	34,319,044	34,324,275	-109	upstream	6.00
NM_013683	Tap1	transporter 1, ATP-binding cassette, sub-family B (MDR/TAP)	17	34,324,825	34,333,744	-441	upstream	6.00
NM_019441	Ppt2	palmitoyl-protein thioesterase 2	17	34,753,607	34,764,042	-566, -5382	upstream, upstream	6.50
NM_030890	Prrt1	proline-rich transmembrane protein 1	17	34,766,631	34,769,205	-2023, 2793	upstream, downstream	6.50
NM_013558	Hspa1l	heat shock protein 1-like	17	35,109,648	35,116,173	10048	downstream	7.00
NM_030597	Lsm2	LSM2 homolog, U6 small nuclear RNA associated (S. cerevisiae)	17	35,119,082	35,122,836	614	in gene	7.00
NM_033478	Ly6g6d	lymphocyte antigen 6 complex, locus G6D	17	35,208,293	35,211,409	-5775	upstream	8.00
NM_027366	Ly6g6e	lymphocyte antigen 6 complex, locus G6E	17	35,213,887	35,215,749	3297	downstream	8.00
NM_178592	Bat5	HLA-B associated transcript 5	17	35,226,236	35,239,932	-9052	upstream	8.00
NM_032460	Bat4	HLA-B associated transcript 4	17	35,259,897	35,261,751	11943	downstream	5.00
NM_010909	Nfkbil1	nuclear factor of kappa light polypeptide gene enhancer in B-cells inhibitor-like 1	17	35,357,120	35,372,760	-40	upstream	8.00
NM_023179	Atp6v1g2	ATPase, H+ transporting, lysosomal V1 subunit G2	17	35,373,541	35,375,712	-741	upstream	8.00
NM_019693	Bat1a	HLA-B-associated transcript 1A	17	35,378,815	35,390,628	-6015, 21217	upstream, downstream	7.50
NM_010380	H2-D1	histocompatibility 2, D region locus 1	17	35,400,039	35,404,442	-7	upstream	7.00
NM_175137	Vars2	valyl-tRNA synthetase 2, mitochondrial (putative)	17	35,793,335	35,804,537	3049	in gene	5.00
NM_010364	Gtf2h4	general transcription factor II H, polypeptide 4	17	35,804,684	35,810,627	9139	downstream	5.00
XM_001480927	LOC100043663	hypothetical protein LOC100043663	17	35,840,843	35,845,189	155	in gene	4.00
NM_133662	Ier3	immediate early response 3	17	35,958,658	35,959,856	334, 1726	in gene, downstream	6.00
NM_008027	Flot1	flotillin 1	17	35,960,302	35,969,732	-1310, 82, 14482	upstream, in gene, downstream	5.67
NM_011655	Tubb5	tubulin, beta 5	17	35,970,865	35,975,177	393	in gene	5.00
NM_001010833	Mdc1	mediator of DNA damage checkpoint 1	17	35,978,443	35,996,615	-3659, 26581	upstream, downstream	6.00
NM_134122	Nrm	nurim (nuclear envelope membrane protein)	17	35,998,263	36,002,345	6761	downstream	7.00
XM_001003622, XM_001474189	2310014H01Rik	RIKEN cDNA 2310014H01 gene	17	36,003,079	36,012,541	1945, 13545	in gene, downstream	6.00
NM_026987	Dhx16	DEAH (Asp-Glu-Ala-His) box polypeptide 16	17	36,016,764	36,029,615	-140	upstream	5.00
NM_010814	Mog	myelin oligodendrocyte glycoprotein	17	37,147,685	37,160,343	-809	upstream	4.00
NM_172621	Clic5	chloride intracellular channel 5	17	44,325,521	44,417,117	41327	in gene	5.00
NM_009820	Runx2	runt related transcription factor 2	17	44,740,950	44,951,576	166472, 144696, 37688	in gene, in gene, in gene	5.33
NM_178652	Supt3h	suppressor of Ty 3 homolog (S. cerevisiae)	17	44,914,120	45,256,233	-232	upstream	5.00
NM_013688	Tcte1	t-complex-associated testis expressed 1	17	45,660,383	45,679,628	27777	downstream	6.00
NM_001013749	Gm323	gene model 323, (NCBI)	17	45,678,901	45,686,626	-1534	upstream	6.00
NM_008690	Nfkbie	nuclear factor of kappa light polypeptide gene enhancer in B-cells inhibitor, epsilon	17	45,692,665	45,700,118	-4505	upstream	6.00
NM_028662	Slc35b2	solute carrier family 35, member B2	17	45,701,101	45,704,618	9779	downstream	10.00
NM_008302	Hsp90ab1	heat shock protein 90kDa alpha (cytosolic), class B member 1	17	45,704,727	45,710,210	-670	upstream	10.00
NM_029338	1700027N10Rik	RIKEN cDNA 1700027N10 gene	17	46,266,226	46,281,147	14203	in gene	8.00
NM_019581	Gtpbp2	GTP binding protein 2	17	46,298,020	46,306,317	7836	in gene	6.00
NM_030715	Polh	polymerase (DNA directed), eta (RAD 30 related)	17	46,308,942	46,339,574	33718	downstream	6.00
NM_028751	Tjap1	tight junction associated protein 1	17	46,394,825	46,419,962	7786	in gene	6.00
NM_029091	Klc4	kinesin light chain 4	17	46,767,580	46,782,093	15741, 15021, 9229	downstream, downstream, in gene	8.33

Accession No.	Symbol	Gene Title	Chr	Gene Start	Gene End	Interval Dists to Start	Interval Pos	Avg Peak
NM_028065	Tnrc5	trinucleotide repeat containing 5	17	46,872,657	46,889,149	-67	upstream	5.00
NM_011195	Ptcra	pre T-cell antigen receptor alpha	17	46,892,610	46,900,661	11445	downstream	5.00
XM_140042, XM_911987	EG240110	predicted gene, EG240110	17	47,179,386	47,179,988	3972	downstream	5.00
NM_172622	Trerf1	transcriptional regulating factor 1	17	47,450,898	47,496,398	-5394	upstream	5.00
NM_001081635, NM_001081636, NM_007632	Ccnd3	cyclin D3	17	47,642,000	47,736,638	90341	in gene	5.00
NM_016859	Bysl	bystin-like	17	47,737,029	47,748,433	16092	downstream	5.00
NM_028232	Sgol1	shugoshin-like 1 (S. pombe)	17	53,815,385	53,828,637	5037	in gene	6.00
NM_020568	S3-12	plasma membrane associated protein, S3-12	17	56,240,017	56,249,226	8330, -550	in gene, upstream	6.00
NM_001077348, NM_025874	2310076L09Rik	RIKEN cDNA 2310076L09 gene	17	56,251,024	56,256,971	16075, 7195	downstream, downstream	6.00
NM_029796	Lrg1	leucine-rich alpha-2-glycoprotein 1	17	56,259,101	56,261,369	11593	downstream	6.00
NM_025566	Tnfaip8l1	tumor necrosis factor, alpha-induced protein 8-like 1	17	56,301,997	56,313,376	10643	in gene	5.00
NM_080837	D17Wsu104e	DNA segment, Chr 17, Wayne State University 104, expressed	17	56,315,964	56,323,343	10703	downstream	5.00
XM_001480312, XM_924956	Safb	scaffold attachment factor B	17	56,724,280	56,745,717	28440	downstream	6.00
NM_153152	2410015M20Rik	RIKEN cDNA 2410015M20 gene	17	56,746,875	56,749,194	-3526	upstream	6.00
NM_018730	Rpl36	ribosomal protein L36	17	56,752,857	56,753,670	-137	upstream	6.00
NM_028782	Lonp1	lon peptidase 1, mitochondrial	17	56,753,720	56,766,326	13606	downstream	6.00
XM_884088, XM_914378	LOC619718	hypothetical LOC619718	17	56,769,821	56,787,232	7651	in gene	5.00
NM_027933	Ranbp3	RAN binding protein 3	17	56,812,680	56,851,192	43288	downstream	5.00
NM_178926	AI662250	expressed sequence AI662250	17	56,853,361	56,856,275	307	in gene	5.00
XM_895878, XM_924988	Ndufa11	NADH dehydrogenase (ubiquinone) 1 alpha subcomplex 11	17	56,857,185	56,862,115	-1217	upstream	5.00
NM_144858	Dus3l	dihydrouridine synthase 3-like (S. cerevisiae)	17	56,904,201	56,909,264	13207	downstream	6.00
XM_148801, XM_914449	Gm546	gene model 546, (NCBI)	17	56,909,699	56,911,557	7709	downstream	6.00
NM_010613	Khsrp	KH-type splicing regulatory protein	17	57,160,474	57,170,930	-1102	upstream	5.00
NM_175333	Slc25a41	solute carrier family 25, member 41	17	57,172,195	57,181,077	9045	downstream	5.00
XM_001478480, XM_484656	A930002H24Rik	RIKEN cDNA A930002H24 gene	17	64,211,417	64,216,530	3314	in gene	8.00
NM_013933	Vapa	vesicle-associated membrane protein, associated protein A	17	65,929,395	65,962,881	33, -511	in gene, upstream	7.50
NM_001025572	Ankrd12	ankyrin repeat domain 12	17	66,316,841	66,426,386	119410	downstream	6.00
NM_009372	Tgif1	TG interacting factor 1	17	71,193,555	71,200,538	90	in gene	6.00
NM_010867	Myom1	myomesin 1	17	71,368,861	71,476,195	110627	downstream	7.00
NM_145158	Emilin2	elastin microfibril interfacer 2	17	71,601,511	71,660,806	10630	in gene	7.00
NM_007439	Alk	anaplastic lymphoma kinase	17	72,218,328	72,953,647	358799	in gene	5.00
NM_027166	Ypel5	yippee-like 5 (Drosophila)	17	73,186,044	73,200,535	900	in gene	7.00
NM_027864	Galnt14	UDP-N-acetyl-alpha-D-galactosamine:polypeptide N-acetylgalactosaminyltransferase 14	17	73,842,568	74,059,404	137228	in gene	5.00
NM_133771	Memo1	mediator of cell motility 1	17	74,600,046	74,694,323	30355	in gene	6.00
NM_152817	Ttc27	tetratricopeptide repeat domain 27	17	75,117,090	75,262,910	49302	in gene	7.00
NM_019919, NM_206958	Ltbp1	latent transforming growth factor beta binding protein 1	17	75,404,869	75,791,109	8571, 36347	in gene, in gene	6.50
NM_026514	Cdc42ep3	CDC42 effector protein (Rho GTPase binding) 3	17	79,733,365	79,754,431	9359	in gene	5.00
NM_201361	AW061290	expressed sequence AW061290	17	80,014,240	80,081,492	400	in gene	6.00
NM_009994	Cyp1b1	cytochrome P450, family 1, subfamily b, polypeptide 1	17	80,106,293	80,114,381	-1747	upstream	5.00
NM_146083	Sfrs7	splicing factor, arginine/serine-rich 7	17	80,599,420	80,606,629	3301, 757	in gene, in gene	5.50
XM_283466, XM_916685	4930560E09Rik	RIKEN cDNA 4930560E09 gene	17	80,615,254	80,617,276	-9382	upstream	4.00
NM_009231	Sos1	Son of sevenless homolog 1 (Drosophila)	17	80,793,092	80,879,793	81	in gene	5.00
NM_001081357	Map4k3	mitogen-activated protein kinase kinase kinase kinase 3	17	80,979,852	81,127,433	86249	in gene	6.00
XM_001481201, XM_001481202	LOC100043900	hypothetical protein LOC100043900	17	83,497,542	83,520,081	-5871	upstream	7.00
NM_011104	Prkce	protein kinase C, epsilon	17	86,567,930	87,054,038	338278	in gene	7.00
NM_010137	Epas1	endothelial PAS domain protein 1	17	87,153,242	87,230,922	35254	in gene	6.00
NM_180974	Foxn2	forkhead box N2	17	88,840,098	88,877,411	382	in gene	7.00
NM_001039692, NM_029277	Arhgap12	Rho GTPase activating protein 12	18	6,024,446	6,136,096	160	in gene	6.00
NM_009280	Ss18	synovial sarcoma translocation, Chromosome 18	18	14,784,138	14,841,423	30895	in gene	6.00
XM_128905, XM_911701	Taf4b	TAF4B RNA polymerase II, TATA box binding protein (TBP)-associated factor	18	14,941,754	15,058,868	-258	upstream	5.00
NM_026529	2700062C07Rik	RIKEN cDNA 2700062C07 gene	18	24,629,372	24,636,268	-2236	upstream	6.00
NM_175276	Fhod3	formin homology 2 domain containing 3	18	24,867,946	25,292,002	172950	in gene	5.00
XM_906763, XM_981660	EG666111	predicted gene, EG666111	18	30,618,603	30,636,468	-9980	upstream	6.00
XM_981691	EG666114	predicted gene, EG666114	18	30,654,557	30,655,126	8678	downstream	6.00
NM_028866	Wdr33	WD repeat domain 33	18	31,986,939	32,066,649	83557	downstream	8.00

Accession No.	Symbol	Gene Title	Chr	Gene Start	Gene End	Interval Dists to Start	Interval Pos	Avg Peak
XM_484715, XM_907118	Sft2d3	SFT2 domain containing 3	18	32,068,198	32,071,573	1077	in gene	8.00
NM_007874	Reep5	receptor accessory protein 5	18	34,504,539	34,532,983	-57	upstream	5.00
NM_030147	Brd8	bromodomain containing 8	18	34,758,269	34,784,464	13616	in gene	7.00
NM_144865	Reep2	receptor accessory protein 2	18	35,000,312	35,007,109	9704	downstream	6.00
NM_144866	Etf1	eukaryotic translation termination factor 1	18	35,062,439	35,091,657	-215	upstream	11.00
NM_001033141	1110006O17Rik	RIKEN cDNA 1110006O17 gene	18	35,872,743	35,881,145	-7207	upstream	6.00
NM_001033168	1700066B19Rik	RIKEN cDNA 1700066B19 gene	18	35,886,643	35,890,523	1709	in gene	6.00
XM_128954, XM_892499, XM_900126, XM_910347, XM_921603, XM_921606, XM_986235, XM_989707	2610307O08Rik	RIKEN cDNA 2610307O08 gene	18	35,893,332	35,900,208	11856	downstream	6.00
XM_984294	LOC666505	similar to 40S ribosomal protein S25	18	35,929,972	35,931,242	1100	in gene	8.00
NM_019912	Ube2d2	ubiquitin-conjugating enzyme E2D 2	18	35,931,261	35,966,817	-189	upstream	8.00
NM_001081365	0610010O12Rik	RIKEN cDNA 0610010O12 gene	18	36,508,278	36,553,024	26074, 35295	in gene, in gene	5.00
NM_172830	Slc4a9	solute carrier family 4, sodium bicarbonate cotransporter, member 9	18	36,687,806	36,704,262	1970	in gene	5.00
NM_009841	Cd14	CD14 antigen	18	36,884,721	36,886,308	-8644	upstream	6.00
NM_028036	Tmco6	transmembrane and coiled-coil domains 6	18	36,894,724	36,902,045	228	in gene	6.00
NM_010885	Ndufa2	NADH dehydrogenase (ubiquinone) 1 alpha subcomplex, 2	18	36,902,020	36,904,202	9250	downstream	6.00
NM_011879	Ik	IK cytokine	18	36,904,370	36,917,289	-9418	upstream	6.00
XM_001478852	1700086O06Rik	RIKEN cDNA 1700086O06 gene	18	38,398,688	38,409,849	-5783	upstream	7.00
NM_024179	0610009O20Rik	RIKEN cDNA 0610009O20 gene	18	38,409,913	38,422,280	5719	in gene	7.00
NM_011937	Gnpda1	glucosamine-6-phosphate deaminase 1	18	38,487,209	38,498,605	-35	upstream	5.00
NM_022996	Ndfip1	Nedd4 family interacting protein 1	18	38,578,629	38,624,060	-5	upstream	9.00
NM_011898	Spry4	sprouty homolog 4 (Drosophila)	18	38,745,919	38,760,922	11882, -854	in gene, upstream	5.50
XR_035375	9630014M24Rik	RIKEN cDNA 9630014M24 gene	18	38,761,188	38,805,576	588	in gene	6.00
NM_001033789	EG545253	predicted gene, EG545253	18	39,052,110	39,130,928	58866	in gene	6.00
NM_008173	Nr3c1	nuclear receptor subfamily 3, group C, member 1	18	39,570,199	39,646,899	-4173	upstream	7.00
NM_023311	Yipf5	Yip1 domain family, member 5	18	40,364,519	40,379,053	-51	upstream	5.00
NM_172966	Sh3rf2	SH3 domain containing ring finger 2	18	42,213,364	42,318,105	1404	in gene	5.00
NM_009468	Dpysl3	dihydropyrimidinase-like 3	18	43,483,936	43,552,985	-1415	upstream	6.00
NM_178872	Trim36	tripartite motif-containing 36	18	46,325,499	46,372,179	14131	in gene	5.00
XM_001479433	LOC100042905	hypothetical protein LOC100042905	18	46,358,675	46,359,385	-627	upstream	5.00
NM_173423	Fem1c	fem-1 homolog c (C.elegans)	18	46,664,260	46,685,625	217	in gene	7.00
NM_173394	Ticam2	toll-like receptor adaptor molecule 2	18	46,717,885	46,734,186	346	in gene	6.00
XM_001478219, XM_001479574	A730092B10	hypothetical protein A730092B10	18	47,698,232	47,878,287	-472	upstream	5.00
NM_026408	Sncaip	synuclein, alpha interacting protein (synphilin)	18	52,927,363	53,075,584	38621	in gene	6.00
NM_175751	Zfp608	zinc finger protein 608	18	55,047,702	55,149,567	-865	upstream	5.00
NM_177115	March3	membrane-associated ring finger (C3HC4) 3	18	56,921,370	57,085,202	145010	in gene	6.00
XM_001001192, XM_980728	1700011I03Rik	RIKEN cDNA 1700011I03 gene	18	57,693,434	57,890,721	70374, 197030	in gene, in gene	6.50
XR_035694	9330166H04Rik	RIKEN cDNA 9330166H04 gene	18	58,037,369	58,038,257	1015	downstream	7.00
NM_009194	Slc12a2	solute carrier family 12, member 2	18	58,037,369	58,106,475	1015	in gene	7.00
NM_133363	Myoz3	myozenin 3	18	60,735,943	60,751,442	-3454, -4398	upstream, upstream	6.50
XM_001479956, XM_001479958, XM_981156	Synpo	synaptopodin	18	60,753,627	60,784,227	29331, 28387	in gene, in gene	6.50
NM_009792, NM_177407	Camk2a	calcium/calmodulin-dependent protein kinase II alpha	18	61,085,286	61,147,798	37754	in gene	10.00
NM_201353	Slc6a7	solute carrier family 6 (neurotransmitter transporter, L-proline), member 7	18	61,155,034	61,173,853	-8035	upstream	6.00
NM_009880	Cdx1	caudal type homeo box 1	18	61,178,519	61,195,853	13965	in gene	6.00
NM_133249	Ppargc1b	peroxisome proliferative activated receptor, gamma, coactivator 1 beta	18	61,457,790	61,560,085	885	in gene	5.00
	Mirn378	microRNA 378	18	61,557,488	61,557,553	-1647	upstream	5.00
NM_019508	Il17b	interleukin 17B	18	61,847,589	61,852,191	-5029	upstream	5.00
NM_172628	Sh3tc2	SH3 domain and tetratricopeptide repeats 2	18	62,112,729	62,175,373	-8393, -4249	upstream, upstream	7.00
XM_001002365, XM_984603	2700046A07Rik	RIKEN cDNA 2700046A07 gene	18	62,911,328	62,915,969	321	in gene	5.00
NM_207255	Zfp532	zinc finger protein 532	18	65,739,884	65,848,587	3316, 6916	in gene, in gene	6.00
NM_178793	Ccbe1	collagen and calcium binding EGF domains 1	18	66,216,510	66,451,492	210564	in gene	4.00
NM_010307, NM_177137	Gnal	guanine nucleotide binding protein, alpha stimulating, olfactory type	18	67,247,990	67,386,446	116906	in gene	8.00
NM_024190	Chmp1b	chromatin modifying protein 1B	18	67,365,077	67,366,655	-181	upstream	8.00
NM_176832, NM_194355	Spire1	spire homolog 1 (Drosophila)	18	67,647,863	67,712,375	52151	in gene	8.00
NM_001039088, NM_028112	Seh1l	SEH1-like (S. cerevisiae	18	67,934,530	67,955,141	25694	downstream	7.00
NM_013685	Tcf4	transcription factor 4	18	69,504,175	69,843,998	317841	in gene	6.00
NM_007831	Dcc	deleted in colorectal carcinoma	18	71,413,286	72,510,723	1052915	in gene	5.00
NM_008540	Smad4	MAD homolog 4 (Drosophila)	18	73,798,667	73,863,395	35939, -61	in gene, upstream	7.00
NM_010720	Lipg	lipase, endothelial	18	75,099,017	75,120,760	-9064	upstream	6.00

Accession No.	Symbol	Gene Title	Chr	Gene Start	Gene End	Interval Dists to Start	Interval Pos	Avg Peak
NM_001002239	Rpl17	ribosomal protein L17	18	75,160,176	75,163,034	5264	downstream	8.00
NM_001001181	BC031181	cDNA sequence BC031181	18	75,165,554	75,169,587	-114	upstream	8.00
NM_027727	Dym	dymeclin	18	75,178,426	75,446,620	121094, 197206	in gene, in gene	6.50
NM_001042660	Smad7	MAD homolog 7 (Drosophila)	18	75,527,019	75,555,588	24928	in gene	5.00
NM_201354	Gm672	gene model 672, (NCBI)	18	75,590,859	75,857,208	269976, 142520, 101368	downstream, in gene, in gene	5.67
NM_016791, NM_198429	Nfatc1	nuclear factor of activated T-cells, cytoplasmic, calcineurin-dependent 1	18	80,802,944	80,909,797	88213	in gene	6.00
NM_183033	Zfp516	zinc finger protein 516	18	83,125,011	83,172,115	-1603	upstream	6.00
XM_976217, XM_991500	Zfp407	zinc finger protein 407	18	84,377,093	84,758,896	-240	upstream	4.00
NM_023149	Cndp2	CNDP dipeptidase 2 (metallopeptidase M20 family)	18	84,836,861	84,855,025	-175	upstream	4.00
XM_001473368	LOC100039711	hypothetical protein LOC100039711	18	84,853,350	84,857,083	1883	in gene	4.00
NM_013495	Cpt1a	carnitine palmitoyltransferase 1a, liver	19	3,323,348	3,385,732	172	in gene	6.00
NM_029456	Saps3	SAPS domain family, member 3	19	3,454,928	3,575,720	-504	upstream	7.00
XM_001480817	LOC100043581	hypothetical protein LOC100043581	19	3,573,969	3,575,718	2255	downstream	7.00
NM_144871	Suv420h1	suppressor of variegation 4-20 homolog 1 (Drosophila)	19	3,767,421	3,816,391	1283	in gene	7.00
NM_026316	Aldh3b1	aldehyde dehydrogenase 3 family, member B1	19	3,913,491	3,929,716	-6412	upstream	5.00
NM_019449	Unc93b1	unc-93 homolog B1 (C. elegans)	19	3,935,219	3,949,340	909	in gene	5.00
NM_008851	Pitpnm1	phosphatidylinositol membrane-associated 1	19	4,100,622	4,113,961	13938	downstream	6.00
NM_001078649, NM_025889	Tmem134	transmembrane protein 134	19	4,125,960	4,132,307	88	in gene	4.00
NM_144532	Cabp4	calcium binding protein 4	19	4,135,423	4,139,609	13561, -7543	downstream, upstream	4.00
NM_206973	Gpr152	G protein-coupled receptor 152	19	4,139,799	4,145,741	7353	downstream	4.00
NM_011778	Coro1b	coronin, actin binding protein 1B	19	4,148,663	4,154,035	-1511	upstream	4.00
NM_016933	Ptprcap	protein tyrosine phosphatase, receptor type, C polypeptide-associated protein	19	4,154,646	4,156,710	-7494	upstream	4.00
NM_198113	Ssh3	slingshot homolog 3 (Drosophila)	19	4,261,668	4,269,172	5460	in gene	7.00
NM_026720	Ankrd13d	ankyrin repeat domain 13 family, member D	19	4,270,180	4,283,137	19425	downstream	7.00
NM_008797	Pcx	pyruvate carboxylase	19	4,594,346	4,621,698	20422	in gene	6.00
NM_153388	Lrfn4	leucine rich repeat and fibronectin type III domain containing 4	19	4,611,792	4,615,502	734	in gene	6.00
NM_023131	Rce1	RCE1 homolog, prenyl protein peptidase (S. cerevisiae)	19	4,622,551	4,625,617	10849	downstream	6.00
NM_009032	Rbm4	RNA binding motif protein 4	19	4,784,293	4,793,877	5893, -8619	in gene, upstream	5.00
NM_019869	Rbm14	RNA binding motif protein 14	19	4,800,925	4,811,634	9138	in gene	4.00
NM_016892	Ccs	copper chaperone for superoxide dismutase	19	4,825,366	4,839,322	-70	upstream	6.00
NM_207268	Ccdc87	coiled-coil domain containing 87	19	4,839,415	4,842,163	-23	upstream	6.00
NM_153553	Npas4	neuronal PAS domain protein 4	19	4,984,355	4,989,971	11731	downstream	7.00
NM_016882	Sart1	squamous cell carcinoma antigen recognized by T-cells 1	19	5,377,523	5,388,703	95	in gene	5.00
XR_031843, XR_034927	Tsga10ip	testis specific 10 interacting protein	19	5,390,046	5,402,460	13852	downstream	5.00
NM_021474	Efemp2	epidermal growth factor-containing fibulin-like extracellular matrix protein 2	19	5,474,750	5,481,853	14338	downstream	7.00
NM_027877	Mus81	MUS81 endonuclease homolog (yeast)	19	5,482,851	5,488,341	-747	upstream	7.00
NM_007687	Cfl1	cofilin 1, non-muscle	19	5,490,525	5,495,201	-1437	upstream	7.00
NM_001024560	B930037P14Rik	RIKEN cDNA B930037P14 gene	19	5,495,278	5,510,489	21401, -4359	downstream, upstream	8.00
NM_019935	Ovol1	OVO homolog-like 1 (Drosophila)	19	5,549,137	5,560,575	-7105, -7585	upstream, upstream	6.50
NM_001033448	Gm962	gene model 962, (NCBI)	19	5,568,074	5,571,261	-394, 86	upstream, in gene	6.50
NM_009045	Rela	v-rel reticuloendotheliosis viral oncogene homolog A (avian)	19	5,637,490	5,648,130	9646	in gene	5.00
NM_011379	Sipa1	signal-induced proliferation associated gene 1	19	5,651,194	5,663,620	16484	downstream	5.00
NM_022012	Map3k11	mitogen activated protein kinase kinase kinase 11	19	5,689,131	5,702,865	10213	in gene	5.00
NM_001004138, NM_010609	Kcnk7	potassium channel, subfamily K, member 7	19	5,704,476	5,707,101	-5132	upstream	5.00
NM_053252	Ehbp1l1	EH domain binding protein 1-like 1	19	5,707,891	5,726,317	26973	downstream	5.00
XR_035481, XR_035482	2310043N10Rik	RIKEN cDNA 2310043N10 gene	19	5,796,401	5,845,579	21743	in gene	5.00
XM_886988, XM_906412	LOC622554	hypothetical LOC622554	19	6,195,680	6,223,566	16256, 31360	in gene, downstream	5.00
NM_130453	Gpha2	glycoprotein hormone alpha 2	19	6,226,401	6,227,768	639	in gene	6.00
NM_198168	Ppp2r5b	protein phosphatase 2, regulatory subunit B (B56), beta isoform	19	6,227,767	6,235,840	8800	downstream	6.00
NM_010119	Ehd1	EH-domain containing 1	19	6,276,896	6,300,096	30827	downstream	4.00
XM_140553, XM_906449	Cdc42bpg	CDC42 binding protein kinase gamma (DMPK-like)	19	6,306,448	6,325,177	1275	in gene	4.00
NM_020253	Nrxn2	neurexin II	19	6,428,016	6,533,217	81872	in gene	5.00
NM_019924	Rps6ka4	ribosomal protein S6 kinase, polypeptide 4	19	6,903,574	6,915,091	-269	upstream	5.00
NM_001081291	Ccdc88	coiled-coil domain containing 88	19	6,919,113	6,932,701	17341	downstream	5.00
NM_007953	Esrra	estrogen related receptor, alpha	19	6,985,467	6,996,298	-1142	upstream	7.00
NM_001039494	1700019N12Rik	RIKEN cDNA 1700019N12 gene	19	6,996,916	6,999,870	2430, -8418	in gene, upstream	6.50
NM_008431	Kcnk4	potassium channel, subfamily K, member 4	19	7,000,180	7,009,005	11565, 717	downstream, in gene	6.50
NM_207220	Gpr137	G protein-coupled receptor 137	19	7,012,560	7,015,969	7681	downstream	6.00

Accession No.	Symbol	Gene Title	Chr	Gene Start	Gene End	Interval Dists to Start	Interval Pos	Avg Peak
NM_007522	Bad	Bcl-associated death promoter	19	7,016,345	7,026,383	-8057	upstream	6.00
NM_134147	D930010J01Rik	RIKEN cDNA D930010J01 gene	19	7,131,300	7,272,547	12908	in gene	6.00
NM_146093	D19Ertd721e	DNA segment, Chr 19, ERATO Doi 721, expressed	19	8,946,049	8,950,146	9407	downstream	7.00
XM_887080, XM_907845	AI462493	expressed sequence AI462493	19	8,954,504	8,955,423	-33	upstream	7.00
NM_025463	1810009A15Rik	RIKEN cDNA 1810009A15 gene	19	8,963,393	8,965,230	-7937, 9215	upstream, downstream	7.50
NM_176843	Ints5	integrator complex subunit 5	19	8,967,495	8,972,378	5113	downstream	8.00
NM_008060	Ganab	alpha glucosidase 2 alpha neutral subunit	19	8,972,601	8,991,156	7	in gene	8.00
NM_010239	Fth1	ferritin heavy chain 1	19	10,057,193	10,059,582	172	in gene	7.00
NM_011913	Best1	bestrophin 1	19	10,059,664	10,071,704	14339	downstream	7.00
NM_018801, NM_173067, NM_173068	Syt7	synaptotagmin VII	19	10,463,973	10,522,934	-725	upstream	5.00
NM_001037801, NM_009852	Cd6	CD6 antigen	19	10,863,829	10,904,548	12260	in gene	6.00
XM_888885, XM_908564	AW112010	expressed sequence AW112010	19	11,122,102	11,125,056	5664	downstream	4.00
NM_008139	Gnaq	guanine nucleotide binding protein, alpha q polypeptide	19	16,207,321	16,461,943	261879	downstream	5.00
XM_001477736	LOC100042193	hypothetical protein LOC100042193	19	18,742,735	18,745,272	2481	in gene	6.00
NM_026120	2410127L17Rik	RIKEN cDNA 2410127L17 gene	19	18,745,270	18,779,282	-54	upstream	6.00
NM_001033759, NM_031997	Tmem2	transmembrane protein 2	19	21,852,832	21,932,817	52256	in gene	5.00
NM_010638	Klf9	Kruppel-like factor 9	19	23,215,716	23,241,401	-7012, -6084	upstream, upstream	9.00
NM_198651	4430402I18Rik	RIKEN cDNA 4430402I18 gene	19	28,997,554	29,038,644	-4172	upstream	7.00
NM_021299	Ak3	adenylate kinase 3	19	29,095,322	29,122,392	-5267	upstream	5.00
XR_035347, XR_035355	2700046G09Rik	RIKEN cDNA 2700046G09 gene	19	32,463,697	32,465,674	-1553	upstream	5.00
NM_008960	Pten	phosphatase and tensin homolog	19	32,832,067	32,900,650	-1219	upstream	7.00
NM_198300	Cpeb3	cytoplasmic polyadenylation element binding protein 3	19	37,095,781	37,281,783	175543	in gene	7.00
NM_001034962, NM_001034963, NM_001034964, NM_009166, NM_178362	Sorbs1	sorbin and SH3 domain containing 1	19	40,366,530	40,588,302	108478	in gene	6.00
XM_001000855, XM_994273	E130314M14Rik	RIKEN cDNA E130314M14 gene	19	40,969,177	40,972,881	3825	downstream	5.00
NM_028319	Zfp518	zinc finger protein 518	19	40,969,195	40,992,437	-139	upstream	5.00
NM_015748	Slit1	slit homolog 1 (Drosophila)	19	41,674,749	41,818,346	68058, 52906, 25658	in gene, in gene, in gene	5.67
NM_145501	Pi4k2a	phosphatidylinositol 4-kinase type 2 alpha	19	42,164,925	42,196,708	307	in gene	6.00
XR_035382, XR_035422	Marveld1	MARVEL (membrane-associating) domain containing 1	19	42,221,879	42,224,737	-2695	upstream	8.00
NM_027694	4933417O08Rik	RIKEN cDNA 4933417O08 gene	19	42,330,273	42,344,838	14895	downstream	5.00
NM_145123	Crtac1	cartilage acidic protein 1	19	42,357,527	42,506,273	133153	in gene	5.00
NM_145156	Slc25a28	solute carrier family 25, member 28	19	43,738,291	43,749,371	-149, -549	upstream, upstream	6.00
NM_053197	Sfxn3	sideroflexin 3	19	45,122,164	45,130,872	18172	downstream	6.00
NM_178929	Kazald1	Kazal-type serine peptidase inhibitor domain 1	19	45,150,629	45,153,772	-1114	upstream	5.00
NM_001037758, NM_009771	Btrc	beta-transducin repeat containing protein	19	45,430,387	45,604,503	174093	in gene	5.00
NM_198296	9130011E15Rik	RIKEN cDNA 9130011E15 gene	19	45,892,634	46,072,978	26290, -142	in gene, upstream	5.00
XM_001000740, XM_001001649, XM_973308	4930505N22Rik	RIKEN cDNA 4930505N22 gene	19	46,072,313	46,073,124	4	in gene	6.00
NM_176785	Hps6	Hermansky-Pudlak syndrome 6	19	46,077,998	46,080,643	-4878	upstream	6.00
NM_001025391, NM_015752	Sufu	suppressor of fused homolog (Drosophila)	19	46,471,408	46,561,150	81936	in gene	5.00
NM_146099	D19Wsu162e	DNA segment, Chr 19, Wayne State University 162, expressed	19	46,673,641	46,731,874	33831	in gene	6.00
NM_007809	Cyp17a1	cytochrome P450, family 17, subfamily a, polypeptide 1	19	46,741,670	46,747,464	4328	in gene	6.00
NM_021360	Neurl	neuralized-like homolog (Drosophila)	19	47,253,310	47,333,931	33314, 85698	in gene, downstream	6.00
NM_177833	EG329070	predicted gene, EG329070	19	47,336,657	47,339,548	2351	in gene	6.00
NM_175360	Obfc1	oligonucleotide/oligosaccharide-binding fold containing 1	19	47,575,538	47,611,510	20870	in gene	6.00
NM_007732	Col17a1	procollagen, type XVII, alpha 1	19	47,720,852	47,766,584	14712	in gene	4.00
NM_007790	Smc3	structural maintenace of chromosomes 3	19	53,674,937	53,719,638	49415	downstream	6.00
XM_001002314, XM_001481268	Rbm20	RNA binding motif protein 20	19	53,751,796	53,940,203	176092	in gene	6.00
NM_009348	Tectb	tectorin beta	19	55,255,375	55,270,803	4753	in gene	5.00
NM_001081076	Gucy2g	guanylate cyclase 2g	19	55,272,591	55,315,726	-3906	upstream	5.00
NM_027976	Acsl5	acyl-CoA synthetase long-chain family member 5	19	55,327,859	55,371,118	-8227, -275	upstream, upstream	4.50
NM_016862	Vti1a	vesicle transport through interaction with t-SNAREs homolog 1A (yeast)	19	55,390,841	55,701,051	85959	in gene	5.00
NM_009333	Tcf7l2	transcription factor 7-like 2, T-cell specific, HMG-box	19	55,816,759	56,007,732	2089, 26105	in gene, in gene	4.50

Accession No.	Symbol	Gene Title	Chr	Gene Start	Gene End	Interval Dists to Start	Interval Pos	Avg Peak
NM_018831	Dclre1a	DNA cross-link repair 1A, PSO2 homolog (S. cerevisiae)	19	56,603,651	56,622,516	3460	in gene	5.00
NM_025811	Nhlrc2	NHL repeat containing 2	19	56,622,751	56,673,336	-3695	upstream	5.00
NM_178688	Ablim1	actin-binding LIM protein 1	19	57,111,630	57,290,522	184218, 146330	downstream, in gene	5.50
NR_002863	Emx2os	empty spiracles homolog 2 (Drosophila) opposite strand	19	59,499,594	59,533,125	22517	in gene	7.00
NM_010123	Eif3s10	eukaryotic translation initiation factor 3, subunit 10 (theta)	19	60,837,025	60,866,546	194	in gene	6.00
NM_018869	Gprk5	G protein-coupled receptor kinase 5	19	60,966,379	61,168,245	-603	upstream	6.00
NM_008423	Kcnd1	potassium voltage-gated channel, Shal-related family, member 1	X	7,400,969	7,413,629	17559	downstream	6.00
NM_138604	Otud5	OTU domain containing 5	X	7,418,957	7,451,986	-429	upstream	6.00
NM_016697	Gpc3	glypican 3	X	49,625,603	49,967,098	160370	in gene	4.00
XM_001473214	LOC100039621	hypothetical protein LOC100039621	X	57,736,736	57,811,637	64256	in gene	5.00
XM_205232, XM_993146	Gm715	gene model 715, (NCBI)	X	57,801,194	57,802,392	-202	upstream	5.00
NM_019870	Ard1	N-acetyltransferase ARD1 homolog (S. cerevisiae)	X	71,162,214	71,167,252	-12	upstream	6.00
NM_023132	Renbp	renin binding protein	X	71,167,461	71,176,157	8893	downstream	6.00
NM_001039059, NM_001039060, NM_001039061, NM_153165	Klhl15	kelch-like 15 (Drosophila)	X	91,480,269	91,518,854	-397	upstream	6.00
NM_010099	Eda	ectodysplasin-A	X	97,170,945	97,596,099	165919	in gene	7.00
NM_009910	Cxcr3	chemokine (C-X-C motif) receptor 3	X	98,926,875	98,929,486	-3842	upstream	4.00
NM_008828	Pgk1	phosphoglycerate kinase 1	X	103,382,463	103,399,038	9281	in gene	6.00
NM_008001	Fgd1	FYVE, RhoGEF and PH domain containing 1	X	147,481,776	147,523,642	-240	upstream	4.00
NM_025660	Ribc1	RIB43A domain with coiled-coils 1	X	148,439,125	148,450,838	-607	upstream	4.00
NM_019710	Smc1a	structural maintenance of chromosomes 1A	X	148,450,971	148,496,510	474	in gene	4.00
NM_198409	Rai2	retinoic acid induced 2	X	158,155,559	158,217,425	29465	in gene	6.00
NM_009453, NM_178794	Zrsr2	zinc finger (CCCH type), RNA binding motif and serine/arginine rich 2	X	160,373,375	160,396,530	-14	upstream	7.00
NM_029730	Mospd2	motile sperm domain containing 2	X	161,398,850	161,418,257	33	in gene	6.00
NM_175027	Fancb	Fanconi anemia, complementation group B	X	161,418,645	161,435,204	-421	upstream	6.00
NM_177429	Ofd1	oral-facial-digital syndrome 1 gene homolog (human)	X	162,828,868	162,878,487	-121	upstream	5.00
NM_025432	Trappc2	trafficking protein particle complex 2	X	162,878,733	162,891,070	-125	upstream	5.00
NM_009707, NM_178754	Arhgap6	Rho GTPase activating protein 6	X	165,233,031	165,742,367	241753	in gene	7.00
NM_010797, NM_183151	Mid1	midline 1	X	166,123,179	166,428,730	301813, 304229, 305109, 307253, 310709, 312533	in gene, in gene, in gene, downstream, downstream, downstream	12.83

Table S2. Differentllly expressed genes in the mutant mice

Probe set	Accession No.	Symbol	Gene Title	Fold Change
1415777_at	NM_018874	Pnliprp1	pancreatic lipase related protein 1	104.07
1454866_s_at	NM_172469	Clic6	chloride intracellular channel 6	62.35
1451857_a_at	NM_175263	Notum	notum pectinacetylesterase homolog (Drosophila)	59.36
1457021_x_at	NM_144547	Amhr2	anti-Mullerian hormone type 2 receptor	51.94
1438967_x_at	NM_144547	Amhr2	anti-Mullerian hormone type 2 receptor	47.77
1437438_x_at	NM_011128	Pnliprp2	pancreatic lipase-related protein 2	43.35
1448186_at	NM_011128	Pnliprp2	pancreatic lipase-related protein 2	42.96
1429647_at	XM_487606	1700027L20Rik	RIKEN cDNA 1700027L20 gene	38.92
1449254_at	NM_009263	Spp1	secreted phosphoprotein 1	37.70
1422867_at	NM_010375	Gzmg	granzyme G	37.03
1420344_x_at	NM_010372	Gzmd	granzyme D	36.40
1460465_at	NM_172399	A930038C07Rik	RIKEN cDNA A930038C07 gene	34.12
1427989_at	NM_144547	Amhr2	anti-Mullerian hormone type 2 receptor	32.11
1454159_a_at	NM_008342	Igfbp2	insulin-like growth factor binding protein 2	31.65
1429076_a_at	NM_023608	Gdpd2	glycerophosphodiester phosphodiesterase	28.91
1436503_at	NM_001001179	BC048546	cDNA sequence BC048546	28.03
1420343_at	NM_010372	Gzmd	granzyme D	26.43
1449500_at	NM_027548	Serpinb7	serine (or cysteine) peptidase inhibitor, clade B,	23.91
1419015_at	NM_016873	Wisp2	WNT1 inducible signaling pathway protein 2	23.21
1436869_at	NM_009170	Shh	sonic hedgehog	22.20
1421227_at	NM_010372	Gzmd /// Gzme	granzyme D /// granzyme E	20.38
1424265_at	NM_028749	Npl	N-acetylneuraminate pyruvate lyase	19.78
1448955_s_at	NM_001042617	Cadps	Ca2+-dependent secretion activator	19.30
1425425_a_at	NM_011915	Wif1	Wnt inhibitory factor 1	18.13
1419072_at	NM_026672	Gstm7	glutathione S-transferase, mu 7	15.11
1434165_at	NM_172469	Clic6	chloride intracellular channel 6	14.07
1450171_x_at	NM_010373	Gzme	granzyme E	13.59
1417676_a_at	NM_001164401	Ptpro	protein tyrosine phosphatase, receptor type, O	13.44
1418679_at	NM_010374	Gzmf	granzyme F	12.59
1428987_at	NM_029297	Dynlrb2	dynein light chain roadblock-type 2	12.48
1415938_at	NM_009258	Spink3	serine peptidase inhibitor, Kazal type 3	12.46
1429841_at	NM_001001979	Megf10	multiple EGF-like-domains 10	12.28
1422177_at	NM_008356	Il13ra2	interleukin 13 receptor, alpha 2	12.17
1435999_at	NM_183136	Spink8	serine peptidase inhibitor, Kazal type 8	12.00
1422667_at	NM_008469	Krt15	keratin 15	11.60
1443322_at	AV328597	---		11.53
1436368_at	NM_001114332	Slc16a10	solute carrier family 16 (monocarboxylic acid	11.21
1427247_at	NM_177664	D3Bwg0562e	DNA segment, Chr 3, Brigham & Women's	10.53
1434539_at	NM_010733	Lrrn3	leucine rich repeat protein 3, neuronal	10.47
1453645_at	XM_001002365	2700046A07Rik	RIKEN cDNA 2700046A07 gene	10.42
1423569_at	NM_025961	Gatm	glycine amidinotransferase (L-arginine:glycine	10.08
1443639_at	NM_133237	Apcdd1	Adenomatosis polyposis coli down-regulated 1	9.90
1419332_at	NM_019397	Egfl6	EGF-like-domain, multiple 6	9.09
1450770_at	NM_026142	3632451O06Rik	RIKEN cDNA 3632451O06 gene	8.65

Probe set	Accession No.	Symbol	Gene Title	Fold Change
1419719_at	NM_008069	Gabrb1	gamma-aminobutyric acid (GABA) A receptor,	8.64
1456509_at	NM_001167996	1110032F04Rik	RIKEN cDNA 1110032F04 gene	8.54
1440374_at	NM_001025568	Pde1c	phosphodiesterase 1C	8.18
1427257_at	NM_001081249	Vcan	versican	8.13
1417343_at	NM_022004	Fxyd6	FXYD domain-containing ion transport regulator	8.02
1451796_s_at	NM_008230	Hdc	histidine decarboxylase	7.87
1421262_at	NM_010720	Lipg	lipase, endothelial	7.79
1424556_at	NM_144795	Pycr1	pyrroline-5-carboxylate reductase 1	7.75
1454713_s_at	NM_008230	Hdc	histidine decarboxylase	7.72
1429506_at	NM_001163660	Nkd1	naked cuticle 1 homolog (Drosophila)	7.69
1421679_a_at	NM_001111099	Cdkn1a	cyclin-dependent kinase inhibitor 1A (P21)	7.64
1421694_a_at	NM_001081249	Vcan	versican	7.58
1416200_at	NM_001164724	Il33	interleukin 33	7.51
1436987_at	NM_001126490	Ism1	isthmin 1 homolog (zebrafish)	7.36
1435095_at	BE980202	C030009O12Rik	RIKEN cDNA C030009O12 gene	7.21
1422941_at	NM_053116	Wnt16	wingless-related MMTV integration site 16	7.05
1422592_at	NM_008729	Ctnnd2	catenin (cadherin associated protein), delta 2	7.03
1438602_s_at	NM_008555	Masp1	mannan-binding lectin serine peptidase 1	6.97
1434592_at	NM_001114332	Slc16a10	solute carrier family 16 (monocarboxylic acid	6.96
1457423_at	NM_177393	Nalcn	sodium leak channel, non-selective	6.90
1449451_at	NM_025867	Serpinb11	serine (or cysteine) peptidase inhibitor, clade B	6.87
1417898_a_at	NM_010370	Gzma	granzyme A	6.76
1422914_at	NM_022435	Sp5	trans-acting transcription factor 5	6.63
1430118_at	XM_001002365	2700046A07Rik	RIKEN cDNA 2700046A07 gene	6.60
1431839_a_at	NM_001162979	Ccdc81	coiled-coil domain containing 81	6.57
1425046_at	NM_144890	BC018465	cDNA sequence BC018465	6.46
1438698_at	NM_175432	Tmem132c	transmembrane protein 132C	6.39
1418383_at	NM_133237	Apcdd1	adenomatosis polyposis coli down-regulated 1	6.35
1418457_at	NM_019568	Cxcl14	chemokine (C-X-C motif) ligand 14	6.31
1418003_at	NM_025427	1190002H23Rik	RIKEN cDNA 1190002H23 gene	6.24
1450377_at	NM_011580	Thbs1	thrombospondin 1	6.21
1436845_at	NM_015732	Axin2	axin2	6.11
1438511_a_at	NM_025427	1190002H23Rik	RIKEN cDNA 1190002H23 gene	6.11
1450188_s_at	NM_010720	Lipg	lipase, endothelial	6.06
1416342_at	NM_011607	Tnc	tenascin C	6.03
1450704_at	NM_010544	Ihh	Indian hedgehog	5.98
1431844_at	NM_028231	Kcnmb2	potassium large conductance calcium-activated	5.85
1425985_s_at	NM_008555	Masp1	mannan-binding lectin serine peptidase 1	5.84
1427256_at	NM_001081249	Vcan	versican	5.77
1460302_at	NM_011580	Thbs1	thrombospondin 1	5.72
1427339_at	NM_001039677	Slc30a2	solute carrier family 30 (zinc transporter), member	5.72
1440617_at	NM_177834	Cpa6	carboxypeptidase A6	5.69
1434292_at	NM_175692	Snhg11	small nucleolar RNA host gene 11	5.67
1449286_at	NM_001163348	Ntng1	netrin G1	5.65
1418382_at	NM_133237	Apcdd1	adenomatosis polyposis coli down-regulated 1	5.63

Probe set	Accession No.	Symbol	Gene Title	Fold Change
1417278_a_at	NM_001163660	Nkd1	naked cuticle 1 homolog (Drosophila)	5.61
1424638_at	NM_001111099	Cdkn1a	cyclin-dependent kinase inhibitor 1A (P21)	5.55
1435162_at	NM_008926	Prkg2	protein kinase, cGMP-dependent, type II	5.53
1416468_at	NM_013467	Aldh1a1	aldehyde dehydrogenase family 1, subfamily A1	5.48
1433600_at	NM_007417	Adra2a	adrenergic receptor, alpha 2a	5.46
1436111_at	NM_001164437	Tmem212	transmembrane protein 212	5.36
1425357_a_at	NM_011824	Grem1	gremlin 1	5.21
1419465_at	NM_028186	Nkd2	naked cuticle 2 homolog (Drosophila)	5.21
1435679_at	NM_181848	Optn	optineurin	5.15
1447356_at	NM_001002900	AB099516 ///	cDNA sequence AB099516 /// HIG1 domain	5.12
1454734_at	NM_010703	Lef1	lymphoid enhancer binding factor 1	5.11
1419466_at	NM_028186	Nkd2	naked cuticle 2 homolog (Drosophila)	5.09
1456335_at	NM_001033288	Gm106	predicted gene 106	5.08
1438399_at	NM_001163516	Pex5l	peroxisomal biogenesis factor 5-like	5.02
1448083_at	NM_177393	Nalcn	sodium leak channel, non-selective	5.00
1449478_at	NM_010810	Mmp7	matrix metallopeptidase 7	4.96
1449070_x_at	NM_133237	Apcdd1	adenomatosis polyposis coli down-regulated 1	4.95
1416053_at	NM_008516	Lrrn1	leucine rich repeat protein 1, neuronal	4.92
1451054_at	NM_008768	Orm1	orosomucoid 1	4.87
1438697_at	NM_175432	Tmem132c	transmembrane protein 132C	4.86
1434275_at	NM_028186	Nkd2	naked cuticle 2 homolog (Drosophila)	4.80
1457429_s_at	NM_001033288	Gm106	predicted gene 106	4.77
1433707_at	NM_010251	Gabra4	gamma-aminobutyric acid (GABA) A receptor,	4.75
1423854_a_at	NM_026878	Rasl11b	RAS-like, family 11, member B	4.74
1434172_at	NM_007726	Cnr1	cannabinoid receptor 1 (brain)	4.68
1417867_at	NM_013459	Cfd	complement factor D (adipsin)	4.62
1418601_at	NM_011921	Aldh1a7	aldehyde dehydrogenase family 1, subfamily A7	4.62
1418456_a_at	NM_019568	Cxcl14	chemokine (C-X-C motif) ligand 14	4.60
1429896_at	BE135996	5830408B19Rik	RIKEN cDNA 5830408B19 gene	4.57
1421471_at	NM_010934	Npy1r	neuropeptide Y receptor Y1	4.56
1449133_at	NM_009264	Sprr1a	small proline-rich protein 1A	4.54
1437528_x_at	NM_001167925	A730017C20Rik	RIKEN cDNA A730017C20 gene	4.48
1447669_s_at	NM_010317	Gng4	guanine nucleotide binding protein (G protein),	4.43
1454604_s_at	NM_173007	Tspan12	tetraspanin 12	4.42
1449182_at	NM_022984	Retn	resistin	4.39
1432156_a_at	NM_021470	Rnf32	ring finger protein 32	4.38
1438452_at	NM_028757	Nebl	nebulette	4.33
1417021_a_at	NM_001083959	Spo11	sporulation protein, meiosis-specific, SPO11	4.33
1460256_at	NM_007606	Car3	carbonic anhydrase 3	4.32
1423183_at	NM_020278	Lgi1	leucine-rich repeat LGI family, member 1	4.23
1435026_at	NM_052994	Spock2	sparc/osteonectin, cwcv and kazal-like domains	4.22
1419148_at	NM_009635	Avil	advillin	4.16
1422723_at	NM_001162475	Stra6	stimulated by retinoic acid gene 6	4.16
1423912_at	NM_001164224	Aspscr1	alveolar soft part sarcoma chromosome region,	4.07
1437403_at	NM_177271	Samd5	sterile alpha motif domain containing 5	4.05

Probe set	Accession No.	Symbol	Gene Title	Fold Change
1435460_at	NM_008926	Prkg2	protein kinase, cGMP-dependent, type II	4.02
1430307_a_at	NM_008615	Me1	malic enzyme 1, NADP(+)-dependent, cytosolic	4.01
1455277_at	NM_020259	Hhip	Hedgehog-interacting protein	3.98
1415977_at	NM_023627	Isyna1	myo-inositol 1-phosphate synthase A1	3.96
1454822_x_at	NM_133237	Apcdd1	adenomatosis polyposis coli down-regulated 1	3.95
1449394_at	NM_020495	Slco1b2	solute carrier organic anion transporter family,	3.90
1422596_at	NM_001141933	Nkain4	Na+/K+ transporting ATPase interacting 4	3.89
1425039_at	NM_145467	Itgbl1	integrin, beta-like 1	3.85
1422758_at	NM_018763	Chst2	carbohydrate sulfotransferase 2	3.85
1422604_at	NM_009474	Uox	urate oxidase	3.84
1447272_s_at	NM_009728	Atp10a	ATPase, class V, type 10A	3.77
1434456_at	NM_198620	Rundc3b	RUN domain containing 3B	3.77
1421811_at	NM_011580	Thbs1	thrombospondin 1	3.75
1436853_a_at	NM_001042451	Snca	synuclein, alpha	3.74
1436450_at	NM_001024931	D11Bwg0517e	DNA segment, Chr 11, Brigham & Women's	3.74
1441936_x_at	BB310408	---		3.74
1421671_at	NM_008291	Hsd17b3	hydroxysteroid (17-beta) dehydrogenase 3	3.69
1421404_at	NM_011339	Cxcl15	chemokine (C-X-C motif) ligand 15	3.69
1449382_at	NM_133661	Slc6a12	solute carrier family 6 (neurotransmitter	3.68
1416776_at	NM_016669	Crym	crystallin, mu	3.68
1417828_at	NM_001109045	Aqp8	aquaporin 8	3.66
1418057_at	NM_001145886	Tiam1	T-cell lymphoma invasion and metastasis 1	3.66
1417160_s_at	NM_007969	Expi	extracellular proteinase inhibitor	3.64
1419225_at	NM_009785	Cacna2d3	calcium channel, voltage-dependent, alpha2/delta	3.61
1439109_at	NM_201362	Ccdc68	coiled-coil domain containing 68	3.57
1424221_at	NM_144796	Susd4	sushi domain containing 4	3.51
1428781_at	NM_001166173	Dmkn	dermokine	3.51
1418158_at	NM_001127259	Trp63	transformation related protein 63	3.50
1416967_at	NM_011443	Sox2	SRY-box containing gene 2	3.49
1418599_at	NM_007729	Col11a1	collagen, type XI, alpha 1	3.49
1433577_at	NM_001167925	A730017C20Rik	RIKEN cDNA A730017C20 gene	3.49
1433977_at	NM_018805	Hs3st3b1	heparan sulfate (glucosamine) 3-O-	3.48
1435851_at	NM_020278	Lgi1	leucine-rich repeat LGI family, member 1	3.47
1442769_at	NM_175418	Mybpc1	myosin binding protein C, slow-type	3.47
1438558_x_at	NM_008239	Foxq1	forkhead box Q1	3.47
1451236_at	NM_001164212	Rerg	RAS-like, estrogen-regulated, growth-inhibitor	3.47
1447520_at	NM_008489	Lbp	lipopolysaccharide binding protein	3.45
1456487_at	NM_009622	Adcy1	adenylate cyclase 1	3.45
1448147_at	NM_001164155	Tnfrsf19	tumor necrosis factor receptor superfamily,	3.45
1455645_at	NM_175418	Mybpc1	myosin binding protein C, slow-type	3.44
1429444_at	NM_026864	Rasl11a	RAS-like, family 11, member A	3.44
1456428_at	NM_011339	Cxcl15	chemokine (C-X-C motif) ligand 15	3.43
1437604_x_at	NM_133237	Apcdd1	adenomatosis polyposis coli down-regulated 1	3.42
1439251_at	NM_008325	Idua	Iduronidase, alpha-L-	3.41
1432556_a_at	NM_001162878	Fam183b	family with sequence similarity 183, member B	3.41

Probe set	Accession No.	Symbol	Gene Title	Fold Change
1442166_at	NM_153166	Cpne5	copine V	3.40
1455301_at	NM_001167860	Wipf3	WAS/WASL interacting protein family, member 3	3.37
1433601_at	NM_007417	Adra2a	adrenergic receptor, alpha 2a	3.37
1426640_s_at	NM_144551	Trib2	tribbles homolog 2 (Drosophila)	3.37
1426322_a_at	NM_028231	Kcnmb2	potassium large conductance calcium-activated	3.36
1452260_at	NM_178373	Cidec	cell death-inducing DFFA-like effector c	3.35
1436251_at	NM_001025568	Pde1c	phosphodiesterase 1C	3.34
1451371_at	NM_029844	Mrap	melanocortin 2 receptor accessory protein	3.33
1447851_x_at	NM_009728	Atp10a	ATPase, class V, type 10A	3.32
1435209_at	NM_001081184	BC057079	cDNA sequence BC057079	3.32
1425212_a_at	NM_001164155	Tnfrsf19	tumor necrosis factor receptor superfamily,	3.30
1460230_at	NM_001111015	Syn2	synapsin II	3.29
1439748_at	NM_001136060	Dpp6	dipeptidylpeptidase 6	3.29
1429778_at	NM_181848	Optn	optineurin	3.26
1415936_at	NM_013867	Bcar3	breast cancer anti-estrogen resistance 3	3.25
1424729_at	NM_145635	Adig	adipogenin	3.24
1419613_at	NM_007738	Col7a1	collagen, type VII, alpha 1	3.24
1449581_at	NM_080595	Emid1	EMI domain containing 1	3.22
1435642_at	NM_178774	Prr18	proline rich region 18	3.21
1458104_a_at	NM_001081369	Ccdc153	coiled-coil domain containing 153	3.21
1416211_a_at	NM_008973	Ptn	pleiotrophin	3.21
1417230_at	NM_001159965	Ralgps2	Ral GEF with PH domain and SH3 binding motif 2	3.20
1457273_at	NM_011856	Odz2	odd Oz/ten-m homolog 2 (Drosophila)	3.19
1452016_at	NM_009663	Alox5ap	arachidonate 5-lipoxygenase activating protein	3.16
1439153_at	NM_001170643	Rnf144b	ring finger protein 144B	3.15
1433972_at	NM_001081557	Camta1	calmodulin binding transcription activator 1	3.14
1456379_x_at	NM_152915	Dner	delta/notch-like EGF-related receptor	3.14
1424096_at	NM_027011	Krt5	keratin 5	3.13
1429273_at	NM_028472	Bmper	BMP-binding endothelial regulator	3.12
1423671_at	NM_152915	Dner	delta/notch-like EGF-related receptor	3.11
1423166_at	NM_001159555	Cd36	CD36 antigen	3.11
1433471_at	NM_009331	Tcf7	transcription factor 7, T-cell specific	3.09
1438035_at	NM_201361	Fam82a1	family with sequence similarity 82, member A1	3.08
1443264_at	NM_013516	Ms4a2	membrane-spanning 4-domains, subfamily A,	3.06
1452353_at	NM_001080707	Gpr155	G protein-coupled receptor 155	3.06
1434089_at	NM_001109975	Synpo	synaptopodin	3.04
1452309_at	NM_026599	Cgnl1	cingulin-like 1	3.04
1418191_at	NM_011909	Usp18	ubiquitin specific peptidase 18	3.03
1448595_a_at	NM_009052	Bex1	brain expressed gene 1	3.02
1422866_at	NM_007731	Col13a1	collagen, type XIII, alpha 1	3.01
1460248_at	NM_018867	Cpxm2	carboxypeptidase X 2 (M14 family)	3.01
1455980_a_at	NM_001033331	Gas2l3	growth arrest-specific 2 like 3	3.00
1441165_s_at	NM_022319	Clstn2	calsyntenin 2	2.98
1455280_at	NM_001198811	Frem1	Fras1 related extracellular matrix protein 1	2.96
1437244_at	NM_001033331	Gas2l3	growth arrest-specific 2 like 3	2.95

Probe set	Accession No.	Symbol	Gene Title	Fold Change
1453523_at	NM_001099774	Krtap17-1	keratin associated protein 17-1	2.95
1448249_at	NM_010271	Gpd1	glycerol-3-phosphate dehydrogenase 1 (soluble)	2.95
1422833_at	NM_010446	Foxa2	forkhead box A2	2.94
1420965_a_at	NM_007930	Enc1	ectodermal-neural cortex 1	2.94
1435645_at	NM_026178	Mmd	monocyte to macrophage differentiation-	2.94
1449279_at	NM_030677	Gpx2	glutathione peroxidase 2	2.93
1416022_at	NM_010634	Fabp5	fatty acid binding protein 5, epidermal	2.93
1449048_s_at	NM_009003	Rab4a	RAB4A, member RAS oncogene family	2.93
1418213_at	NM_033373	Krt23	keratin 23	2.93
1437311_at	NM_175692	Snhg11	small nucleolar RNA host gene 11	2.92
1450061_at	NM_007930	Enc1	ectodermal-neural cortex 1	2.91
1438713_at	NM_027760	Rassf8	Ras association (RalGDS/AF-6) domain family (N-	2.91
1436988_at	NM_001126490	Ism1	isthmin 1 homolog (zebrafish)	2.91
1418326_at	NM_011404	Slc7a5	solute carrier family 7 (cationic amino acid	2.91
1418341_at	NM_009003	Rab4a	RAB4A, member RAS oncogene family	2.91
1417788_at	NM_011430	Sncg	synuclein, gamma	2.90
1439106_at	NM_172867	Zfp462	zinc finger protein 462	2.89
1448416_at	NM_008597	Mgp	matrix Gla protein	2.89
1455627_at	NM_007739	Col8a1	collagen, type VIII, alpha 1	2.89
1419748_at	NM_011994	Abcd2	ATP-binding cassette, sub-family D (ALD),	2.89
1434703_at	NM_018788	Extl3	exostoses (multiple)-like 3	2.88
1422870_at	NM_013553	Hoxc4	homeobox C4	2.88
1437967_at	NM_001025576	Ccdc141	coiled-coil domain containing 141	2.88
1436329_at	NM_018781	Egr3	early growth response 3	2.88
1416523_at	NM_011271	Rnase1	ribonuclease, RNase A family, 1 (pancreatic)	2.87
1420719_at	NM_031374	Tex15	testis expressed gene 15	2.87
1419195_at	NM_001045554	Wfdc15b	WAP four-disulfide core domain 15B	2.87
1440510_at	XR_105307	C430002N11Rik	RIKEN cDNA C430002N11 gene	2.86
1424542_at	NM_011311	S100a4	S100 calcium binding protein A4	2.86
1436041_at	NM_010402	Hand2	heart and neural crest derivatives expressed	2.86
1424704_at	NM_001145920	Runx2	runt related transcription factor 2	2.85
1419000_at	NM_018867	Cpxm2	carboxypeptidase X 2 (M14 family)	2.85
1458947_at	AV341977	---		2.85
1422479_at	NM_019811	Acss2	acyl-CoA synthetase short-chain family member 2	2.84
1416021_a_at	NM_010634	Fabp5 ///	fatty acid binding protein 5, epidermal ///	2.83
1435047_at	NM_023852	Rab3c	RAB3C, member RAS oncogene family	2.83
1456789_at	NM_172867	Zfp462	zinc finger protein 462	2.83
1416658_at	NM_011356	Frzb	frizzled-related protein	2.82
1427963_s_at	NM_153133	Rdh9	retinol dehydrogenase 9	2.82
1418744_s_at	NM_021344	Tesc	tescalcin	2.81
1441075_at	NM_181547	Nostrin	nitric oxide synthase trafficker	2.81
1449154_at	NM_007729	Col11a1	collagen, type XI, alpha 1	2.81
1460061_at	AV342748	---		2.80
1455063_at	NM_201361	Fam82a1	family with sequence similarity 82, member A1	2.80
1419254_at	NM_008638	Mthfd2	methylenetetrahydrofolate dehydrogenase (NAD+	2.80

Probe set	Accession No.	Symbol	Gene Title	Fold Change
1443205_at	AU067727	D5Buc30e	DNA segment, Chr 5, Bucan 30 expressed	2.79
1416612_at	NM_009994	Cyp1b1	cytochrome P450, family 1, subfamily b,	2.79
1453102_at	NM_001172160	Flrt3	fibronectin leucine rich transmembrane protein 3	2.79
1425002_at	NM_145373	Sectm1a	secreted and transmembrane 1A	2.79
1449280_at	NM_023612	Esm1	endothelial cell-specific molecule 1	2.78
1436203_a_at	BM730637	1110059G02Rik	RIKEN cDNA 1110059G02 gene	2.78
1426318_at	NM_173052	Serpinb1b	serine (or cysteine) peptidase inhibitor, clade B,	2.78
1438720_at	NM_001162537	9330159F19Rik	RIKEN cDNA 9330159F19 gene	2.77
1437308_s_at	NM_010169	F2r	coagulation factor II (thrombin) receptor	2.77
1428572_at	NM_027395	Basp1	brain abundant, membrane attached signal	2.76
1417541_at	NM_008234	Hells	helicase, lymphoid specific	2.75
1452013_at	NM_009728	Atp10a	ATPase, class V, type 10A	2.75
1455845_at	NM_177618	Wscd1	WSC domain containing 1	2.74
1450643_s_at	NM_007981	Acsl1	acyl-CoA synthetase long-chain family member 1	2.73
1428535_at	NM_001081963	9430020K01Rik	RIKEN cDNA 9430020K01 gene	2.72
1442012_at	AV381755	AU015791	expressed sequence AU015791	2.72
1450852_s_at	NM_010169	F2r	coagulation factor II (thrombin) receptor	2.72
1430964_at	AK009622	2310034O05Rik	RIKEN cDNA 2310034O05 gene	2.71
1434685_at	NM_177664	D3Bwg0562e	DNA segment, Chr 3, Brigham & Women's	2.70
1447807_s_at	NM_181073	Plekhh1	pleckstrin homology domain containing, family H	2.69
1451912_a_at	NM_001164259	Fgfrl1	fibroblast growth factor receptor-like 1	2.66
1453003_at	NM_011436	Sorl1	sortilin-related receptor, LDLR class A repeats-	2.64
1428789_at	NM_001159965	Ralgps2	Ral GEF with PH domain and SH3 binding motif 2	2.63
1436398_at	BB033554	---		2.62
1418425_at	NM_130458	Sp7	Sp7 transcription factor 7	2.62
1425837_a_at	NM_009834	Ccrn4l	CCR4 carbon catabolite repression 4-like (S.	2.60
1422612_at	NM_013820	Hk2	hexokinase 2	2.59
1420796_at	NM_009644	Ahrr	aryl-hydrocarbon receptor repressor	2.59
1427510_at	NM_010305	Gnai1	guanine nucleotide binding protein (G protein),	2.58
1417273_at	NM_013743	Pdk4	pyruvate dehydrogenase kinase, isoenzyme 4	2.58
1417266_at	NM_009139	Ccl6	chemokine (C-C motif) ligand 6	2.58
1417429_at	NM_010231	Fmo1	flavin containing monooxygenase 1	2.57
1428460_at	NM_001111015	Syn2	synapsin II	2.57
1429400_at	NM_016691	Clcn5	chloride channel 5	2.57
1448881_at	NM_017370	Hp	haptoglobin	2.57
1417419_at	NM_007631	Ccnd1	cyclin D1	2.57
1416630_at	NM_008321	Id3	inhibitor of DNA binding 3	2.56
1430697_at	NM_019496	Ammecr1	Alport syndrome, mental retardation, midface	2.55
1416111_at	NM_009856	Cd83	CD83 antigen	2.55
1435917_at	NM_026950	Ociad2	OCIA domain containing 2	2.54
1423571_at	NM_007901	S1pr1	sphingosine-1-phosphate receptor 1	2.54
1439492_at	NM_175467	Sptlc3	serine palmitoyltransferase, long chain base	2.54
1453416_at	NM_001033331	Gas2l3	growth arrest-specific 2 like 3	2.53
1428223_at	NM_029662	Mfsd2a	major facilitator superfamily domain containing	2.52
1450784_at	NM_016678	Reck	reversion-inducing-cysteine-rich protein with	2.52

Probe set	Accession No.	Symbol	Gene Title	Fold Change
1418493_a_at	NM_001042451	Snca	synuclein, alpha	2.50
1448710_at	NM_009911	Cxcr4	chemokine (C-X-C motif) receptor 4	2.49
1450468_at	NM_010865	Myoc	myocilin	2.49
1426225_at	NM_001159487	Rbp4	retinol binding protein 4, plasma	2.49
1427542_at	NM_175407	Sobp	sine oculis-binding protein homolog (Drosophila)	2.49
1452517_at	NM_181073	Plekhh1	pleckstrin homology domain containing, family H	2.49
1449342_at	NM_023587	Ptplb	protein tyrosine phosphatase-like (proline instead	2.49
1434052_at	NM_178906	AI593442	expressed sequence AI593442	2.49
1430584_s_at	NM_007606	Car3	carbonic anhydrase 3	2.48
1438036_x_at	NM_201361	Fam82a1	family with sequence similarity 82, member A1	2.48
1437329_at	NM_023587	Ptplb	protein tyrosine phosphatase-like (proline instead	2.47
1420425_at	NM_007548	Prdm1	PR domain containing 1, with ZNF domain	2.46
1453125_at	NM_009234	Sox11	SRY-box containing gene 11	2.46
1435387_at	NM_001033633	Slc2a13	solute carrier family 2 (facilitated glucose	2.46
1421841_at	NM_001163215	Fgfr3	fibroblast growth factor receptor 3	2.46
1419288_at	NM_023844	Jam2	junction adhesion molecule 2	2.45
1448550_at	NM_008489	Lbp	lipopolysaccharide binding protein	2.45
1424433_at	NM_029619	Msrb2	methionine sulfoxide reductase B2	2.45
1420731_a_at	NM_007792	Csrp2	cysteine and glycine-rich protein 2	2.44
1444341_at	NM_175418	Mybpc1	myosin binding protein C, slow-type	2.44
1417561_at	NM_001110009	Apoc1	apolipoprotein C-I	2.44
1456084_x_at	NM_021355	Fmod	fibromodulin	2.44
1418908_at	NM_013626	Pam	peptidylglycine alpha-amidating monooxygenase	2.44
1418206_at	NM_022324	Sdf2l1	stromal cell-derived factor 2-like 1	2.43
1437992_x_at	NM_010288	Gja1	gap junction protein, alpha 1	2.42
1452514_a_at	NM_001122733	Kit	kit oncogene	2.42
1449880_s_at	NM_001032298	Bglap /// Bglap-	bone gamma carboxyglutamate protein /// bone	2.42
1438012_at	NM_178726	Ppm1l	protein phosphatase 1 (formerly 2C)-like	2.41
1436204_at	BM730637	1110059G02Rik	RIKEN cDNA 1110059G02 gene	2.41
1424266_s_at	NM_144930	Ces1f	carboxylesterase 1F	2.41
1418165_at	NM_010584	Itln1	intelectin 1 (galactofuranose binding)	2.41
1453022_at	NM_026730	Gpihbp1	GPI-anchored HDL-binding protein 1	2.40
1450884_at	NM_001159555	Cd36	CD36 antigen	2.40
1457275_at	NM_183312	Synm	synemin, intermediate filament protein	2.40
1446408_at	NM_001122950	Hoxa10	Homeobox A10	2.40
1432517_a_at	NM_010924	Nnmt	nicotinamide N-methyltransferase	2.40
1421375_a_at	NM_011313	S100a6	S100 calcium binding protein A6 (calcyclin)	2.40
1417673_at	NM_016719	Grb14	growth factor receptor bound protein 14	2.39
1423883_at	NM_007981	Acsl1	acyl-CoA synthetase long-chain family member 1	2.39
1417697_at	NM_009230	Soat1	sterol O-acyltransferase 1	2.39
1426152_a_at	NM_013598	Kitl	kit ligand	2.39
1423258_at	NM_021889	Syt9	synaptotagmin IX	2.39
1417936_at	NM_011338	Ccl9	chemokine (C-C motif) ligand 9	2.39
1421009_at	NM_021384	Rsad2	radical S-adenosyl methionine domain containing	2.39
1440015_at	BB528896	---		2.39

Probe set	Accession No.	Symbol	Gene Title	Fold Change
1429861_at	NM_001081377	Pcdh9	protocadherin 9	2.38
1460458_at	NM_030209	Crispld2	cysteine-rich secretory protein LCCL domain	2.38
1423418_at	NM_134469	Fdps	farnesyl diphosphate synthetase	2.37
1429310_at	NM_001172160	Flrt3	fibronectin leucine rich transmembrane protein 3	2.37
1450387_s_at	NM_001177602	Ak4	adenylate kinase 4	2.36
1436287_at	XM_003084771	Gm10664	predicted gene 10664	2.36
1434719_at	NM_175628	A2m	alpha-2-macroglobulin	2.36
1431422_a_at	NM_019819	Dusp14	dual specificity phosphatase 14	2.35
1460593_at	NM_144796	Susd4	sushi domain containing 4	2.35
1418945_at	NM_010809	Mmp3	matrix metallopeptidase 3	2.35
1429051_s_at	NM_009234	Sox11	SRY-box containing gene 11	2.35
1423371_at	NM_025882	Pole4	polymerase (DNA-directed), epsilon 4 (p12	2.35
1438288_x_at	BB766817	1110059G02Rik	RIKEN cDNA 1110059G02 gene	2.35
1415800_at	NM_010288	Gja1	gap junction protein, alpha 1	2.34
1439117_at	NM_001040682	Clmn	calmin	2.34
1430425_at	NM_172800	Sdk2	sidekick homolog 2 (chicken)	2.34
1429030_at	NM_001135172	C1qtnf7	C1q and tumor necrosis factor related protein 7	2.33
1418743_a_at	NM_021344	Tesc	tescalcin	2.33
1452288_at	NM_172742	Mtmr10	myotubularin related protein 10	2.33
1425099_a_at	NM_007489	Arntl	aryl hydrocarbon receptor nuclear translocator-	2.32
1455610_at	NM_183312	Synm	synemin, intermediate filament protein	2.32
1457851_at	BG066654	---		2.32
1458379_at	NM_001162537	9330159F19Rik	RIKEN cDNA 9330159F19 gene	2.32
1452888_at	NM_028637	1110034G24Rik	RIKEN cDNA 1110034G24 gene	2.32
1455851_at	NM_007555	Bmp5	bone morphogenetic protein 5	2.32
1423311_s_at	NM_001164792	Tpbg	trophoblast glycoprotein	2.32
1457038_at	NM_172862	Frem2	Fras1 related extracellular matrix protein 2	2.31
1449186_at	NM_026121	Bag4	BCL2-associated athanogene 4	2.31
1420249_s_at	NM_009139	Ccl6	chemokine (C-C motif) ligand 6	2.31
1424015_at	NM_021494	Dennd5a	DENN/MADD domain containing 5A	2.30
1417019_a_at	NM_001025779	Cdc6	cell division cycle 6 homolog (S. cerevisiae)	2.30
1433939_at	NM_010678	Aff3	AF4/FMR2 family, member 3	2.30
1415864_at	NM_007563	Bpgm	2,3-bisphosphoglycerate mutase	2.30
1437932_a_at	NM_016674	Cldn1	claudin 1	2.30
1424842_a_at	NM_029270	Arhgap24	Rho GTPase activating protein 24	2.30
1428357_at	NM_173744	2610019F03Rik	RIKEN cDNA 2610019F03 gene	2.29
1437066_at	NM_019778	Zbtb20	zinc finger and BTB domain containing 20	2.29
1420739_at	NM_008779	Cntn3	contactin 3	2.29
1423285_at	NM_001198835	Coch	coagulation factor C homolog (Limulus	2.28
1457248_x_at	NM_010476	Hsd17b7	hydroxysteroid (17-beta) dehydrogenase 7	2.28
1453509_at	NM_026671	Lypd2	Ly6/Plaur domain containing 2	2.28
1436058_at	NM_021384	Rsad2	radical S-adenosyl methionine domain containing	2.27
1453187_at	NM_026950	Ociad2	OCIA domain containing 2	2.27
1433719_at	NM_177909	Slc9a9	solute carrier family 9 (sodium/hydrogen	2.26
1452418_at	BF719154	1200016E24Rik	RIKEN cDNA 1200016E24 gene	2.26

Probe set	Accession No.	Symbol	Gene Title	Fold Change
1423372_at	NM_025882	Pole4	polymerase (DNA-directed), epsilon 4 (p12	2.26
1453622_s_at	NM_027326	Mllt3	myeloid/lymphoid or mixed-lineage leukemia	2.25
1451458_at	NM_001033759	Tmem2	transmembrane protein 2	2.25
1453317_a_at	NM_010158	Khdrbs3	KH domain containing, RNA binding, signal	2.25
1441687_at	NM_009523	LOC100503611	hypothetical protein LOC100503611 /// wingless-	2.25
1417389_at	NM_016696	Gpc1	glypican 1	2.24
1438650_x_at	NM_010288	Gja1	gap junction protein, alpha 1	2.24
1423022_at	NM_007417	Adra2a	adrenergic receptor, alpha 2a	2.24
1434777_at	NM_008506	Mycl1	v-myc myelocytomatosis viral oncogene homolog	2.24
1444905_at	BG069198	D1Ertd705e	DNA segment, Chr 1, ERATO Doi 705, expressed	2.23
1430923_at	AK011946	2610300M13Rik	RIKEN cDNA 2610300M13 gene	2.22
1434758_at	NM_030209	Crispld2	cysteine-rich secretory protein LCCL domain	2.22
1460163_at	NM_001135172	C1qtnf7	C1q and tumor necrosis factor related protein 7	2.22
1422072_a_at	NM_008184	Gstm6	glutathione S-transferase, mu 6	2.22
1429660_s_at	NM_008017	Smc2	structural maintenance of chromosomes 2	2.22
1448021_at	NM_001142952	Fam46c	family with sequence similarity 46, member C	2.21
1437347_at	NM_001136061	Ednrb	endothelin receptor type B	2.21
1420385_at	NM_008137	Gna14	guanine nucleotide binding protein, alpha 14	2.20
1437056_x_at	NM_030209	Crispld2	cysteine-rich secretory protein LCCL domain	2.20
1452257_at	NM_001122683	Bdh1	3-hydroxybutyrate dehydrogenase, type 1	2.19
1424338_at	NM_144512	Slc6a13	solute carrier family 6 (neurotransmitter	2.19
1418440_at	NM_007739	Col8a1	collagen, type VIII, alpha 1	2.19
1431890_a_at	NM_027326	Mllt3	myeloid/lymphoid or mixed-lineage leukemia	2.19
1448700_at	NM_008059	G0s2	G0/G1 switch gene 2	2.19
1420342_at	XM_895068	Gdap10	ganglioside-induced differentiation-associated-	2.19
1438603_x_at	NM_008555	Masp1	mannan-binding lectin serine peptidase 1	2.19
1452283_at	NM_027760	Rassf8	Ras association (RalGDS/AF-6) domain family (N-	2.18
1425863_a_at	NM_001164401	Ptpro	protein tyrosine phosphatase, receptor type, O	2.18
1429471_at	NM_001048005	1110017D15Rik	RIKEN cDNA 1110017D15 gene	2.18
1436790_a_at	NM_009234	Sox11	SRY-box containing gene 11	2.18
1447791_s_at	NM_008137	Gna14	guanine nucleotide binding protein, alpha 14	2.17
1426283_at	NM_172290	Ntm	neurotrimin	2.17
1419251_at	NM_001159964	Eps15	epidermal growth factor receptor pathway	2.16
1422324_a_at	NM_008970	Pthlh	parathyroid hormone-like peptide	2.16
1435551_at	NM_175276	Fhod3	formin homology 2 domain containing 3	2.16
1448789_at	NM_053080	Aldh1a3	aldehyde dehydrogenase family 1, subfamily A3	2.15
1417803_at	NM_001164210	1110032A04Rik	RIKEN cDNA 1110032A04 gene	2.15
1422727_at	NM_080637	Nme5	non-metastatic cells 5, protein expressed in	2.15
1417801_a_at	NM_001163557	Ppfibp2	PTPRF interacting protein, binding protein 2	2.15
1457686_at	NM_001081956	Akap17b	A kinase (PRKA) anchor protein 17B	2.15
1450863_a_at	NM_001111051	Dclk1	doublecortin-like kinase 1	2.15
1437439_at	NM_001162906	2410089E03Rik	RIKEN cDNA 2410089E03 gene	2.15
1423310_at	NM_001164792	Tpbg	trophoblast glycoprotein	2.15
1455717_s_at	NM_001008231	Daam2	dishevelled associated activator of	2.15
1447878_s_at	NM_001164259	Fgfrl1	fibroblast growth factor receptor-like 1	2.15

Probe set	Accession No.	Symbol	Gene Title	Fold Change
1448201_at	NM_009144	Sfrp2	secreted frizzled-related protein 2	2.14
1448613_at	NM_007899	Ecm1	extracellular matrix protein 1	2.14
1429209_at	NM_153393	Col23a1	collagen, type XXIII, alpha 1	2.14
1450451_at	NM_052994	Spock2	sparc/osteonectin, cwcv and kazal-like domains	2.14
1425526_a_at	NM_001025570	Prrx1	paired related homeobox 1	2.14
1453320_at	NM_029604	1700027A23Rik	RIKEN cDNA 1700027A23 gene	2.14
1453238_s_at	NR_002860	3930401B19Rik	RIKEN cDNA 3930401B19 gene /// RIKEN cDNA	2.14
1416490_at	NM_025458	Tmed6	transmembrane emp24 protein transport domain	2.13
1433716_x_at	NM_008115	Gfra2	glial cell line derived neurotrophic factor family	2.13
1428909_at	NR_002860	A130040M12Rik	RIKEN cDNA A130040M12 gene	2.13
1415939_at	NM_021355	Fmod	fibromodulin	2.13
1426981_at	NM_011048	Pcsk6	proprotein convertase subtilisin/kexin type 6	2.13
1428705_at	NM_027040	1700007K13Rik	RIKEN cDNA 1700007K13 gene	2.13
1427231_at	NM_019413	Robo1	roundabout homolog 1 (Drosophila)	2.13
1434129_s_at	NM_172589	Lhfpl2	lipoma HMGIC fusion partner-like 2	2.12
1416939_at	NM_026438	Ppa1	pyrophosphatase (inorganic) 1	2.12
1438945_x_at	NM_010288	Gja1	gap junction protein, alpha 1	2.12
1421341_at	NM_015732	Axin2	axin2	2.11
1437065_at	NM_019778	Zbtb20	zinc finger and BTB domain containing 20	2.11
1438672_at	NM_133167	Parvb	parvin, beta	2.10
1438789_s_at	NM_001136086	Dpysl3	dihydropyrimidinase-like 3	2.10
1451064_a_at	NM_177420	Psat1	phosphoserine aminotransferase 1	2.10
1417355_at	NM_008817	Peg3	paternally expressed 3	2.09
1449166_at	NM_001163525	S100a14	S100 calcium binding protein A14	2.09
1435061_at	NM_001031664	Nudt10	nudix (nucleoside diphosphate linked moiety X)-	2.09
1439015_at	NM_010279	Gfra1	glial cell line derived neurotrophic factor family	2.09
1417529_at	NM_011228	Rab33a	RAB33A, member of RAS oncogene family	2.09
1430139_at	NM_008234	Hells	helicase, lymphoid specific	2.09
1422735_at	NM_008239	Foxq1	forkhead box Q1	2.08
1430435_at	NM_010678	Aff3	AF4/FMR2 family, member 3	2.08
1425518_at	NM_019688	Rapgef4	Rap guanine nucleotide exchange factor (GEF) 4	2.08
1453225_at	NM_172399	A930038C07Rik	RIKEN cDNA A930038C07 gene	2.07
1448898_at	NM_011338	Ccl9	chemokine (C-C motif) ligand 9	2.07
1428731_at	NM_030180	Usp54	ubiquitin specific peptidase 54	2.07
1429205_at	NM_027326	Mllt3	myeloid/lymphoid or mixed-lineage leukemia	2.07
1452837_at	NM_001164885	Lpin2	lipin 2	2.06
1451577_at	NM_019778	Zbtb20	zinc finger and BTB domain containing 20	2.06
1435653_at	NM_018811	Abhd2	abhydrolase domain containing 2	2.06
1435511_at	NM_001111015	Syn2	synapsin II	2.06
1455392_at	NM_178774	Prr18	proline rich region 18	2.06
1449408_at	NM_023844	Jam2	junction adhesion molecule 2	2.06
1449110_at	NM_007483	Rhob	ras homolog gene family, member B	2.06
1439543_at	XM_622231	1110064A23Rik	RIKEN cDNA 1110064A23 gene	2.05
1440446_at	NM_001163660	Nkd1	naked cuticle 1 homolog (Drosophila)	2.05
1452661_at	NM_011638	Tfrc	transferrin receptor	2.05

Probe set	Accession No.	Symbol	Gene Title	Fold Change
1435699_at	NM_178726	Ppm1l	protein phosphatase 1 (formerly 2C)-like	2.05
1441316_at	NM_011720	Wnt8b	wingless related MMTV integration site 8b	2.05
1435089_at	NM_028079	2010111I01Rik	RIKEN cDNA 2010111I01 gene	2.04
1455055_at	NM_001164620	Ccdc157	coiled-coil domain containing 157	2.04
1425546_a_at	NM_133977	Trf	transferrin	2.04
1429166_s_at	NM_001040682	Clmn	calmin	2.04
1434440_at	NM_010305	Gnai1	guanine nucleotide binding protein (G protein),	2.04
1445949_at	BG069486	---		2.04
1423312_at	NM_001164792	Tpbg	trophoblast glycoprotein	2.04
1455466_at	NM_001081342	Gpr133	G protein-coupled receptor 133	2.03
1450782_at	NM_009523	Wnt4	wingless-related MMTV integration site 4	2.03
1419147_at	NM_020002	Rec8	REC8 homolog (yeast)	2.02
1435787_at	NM_178726	Ppm1l	protein phosphatase 1 (formerly 2C)-like	2.02
1427932_s_at	NR_002860	1200003I10Rik	RIKEN cDNA 1200003I10 gene /// RIKEN cDNA	2.02
1424400_a_at	NM_027406	Aldh1l1 ///	aldehyde dehydrogenase 1 family, member L1 ///	2.02
1448961_at	NM_001195084	Plscr2	phospholipid scramblase 2	2.02
1417937_at	NM_001190466	Dact1	dapper homolog 1, antagonist of beta-catenin	2.02
1435123_at	NM_001082483	Efr3b	EFR3 homolog B (S. cerevisiae)	2.02
1420941_at	NM_009063	Rgs5	regulator of G-protein signaling 5	2.01
1430700_a_at	NM_013737	Pla2g7	phospholipase A2, group VII (platelet-activating	2.01
1455320_at	NM_021524	Nampt	nicotinamide phosphoribosyltransferase	2.01
1448029_at	NM_011535	Tbx3	T-box 3	2.01
1435137_s_at	NR_002860	1200015M12Rik	RIKEN cDNA 1200015M12 gene /// RIKEN cDNA	2.01
1452836_at	NM_001164885	Lpin2	lipin 2	2.01
1421641_at	NM_009209	Slc6a2	solute carrier family 6 (neurotransmitter	2.01
1423078_a_at	NM_025436	Sc4mol	sterol-C4-methyl oxidase-like	2.01
1451991_at	NM_001122889	Epha7	Eph receptor A7	2.01
1431429_a_at	NM_001039515	Arl4a	ADP-ribosylation factor-like 4A	2.00
1421908_a_at	NM_011544	Tcf12	transcription factor 12	2.00
1451062_a_at	NM_001163516	Pex5l	peroxisomal biogenesis factor 5-like	2.00
1418203_at	NM_021451	Pmaip1	phorbol-12-myristate-13-acetate-induced protein	2.00
1449848_at	NM_008137	Gna14	guanine nucleotide binding protein, alpha 14	2.00
1426575_at	NM_001168525	Sgms1	sphingomyelin synthase 1	1.99
1435479_at	NM_007557	Bmp7	bone morphogenetic protein 7	1.99
1423804_a_at	NM_145360	Idi1	isopentenyl-diphosphate delta isomerase	1.99
1434820_s_at	NM_001039390	Pkig	protein kinase inhibitor, gamma	1.99
1422598_at	NM_009813	Casq1	calsequestrin 1	1.99
1420377_at	NM_009181	St8sia2	ST8 alpha-N-acetyl-neuraminide alpha-2,8-	1.99
1435110_at	NM_029770	Unc5b	unc-5 homolog B (C. elegans)	1.99
1435053_s_at	NM_181073	Plekhh1	pleckstrin homology domain containing, family H	1.98
1448254_at	NM_008973	Ptn	pleiotrophin	1.98
1435092_at	NM_001039515	Arl4a	ADP-ribosylation factor-like 4A	1.98
1434362_at	BM249454	---		1.98
1423945_a_at	NM_001039390	Pkig	protein kinase inhibitor, gamma	1.98
1416411_at	NM_008183	Gstm2	glutathione S-transferase, mu 2	1.98

Probe set	Accession No.	Symbol	Gene Title	Fold Change
1438329_at	NM_205823	Tlr12	toll-like receptor 12	1.98
1438855_x_at	NM_009396	Tnfaip2	tumor necrosis factor, alpha-induced protein 2	1.98
1460129_at	NM_009209	Slc6a2	solute carrier family 6 (neurotransmitter	1.97
1428136_at	NM_013834	Sfrp1	secreted frizzled-related protein 1	1.97
1438443_at	NM_019778	Zbtb20	zinc finger and BTB domain containing 20	1.97
1437617_x_at	NM_028637	1110034G24Rik	RIKEN cDNA 1110034G24 gene	1.97
1436050_x_at	NM_019479	Hes6	hairy and enhancer of split 6 (Drosophila)	1.96
1418957_at	NM_016853	Stac	src homology three (SH3) and cysteine rich	1.96
1431777_a_at	NM_026122	Hmgn3	high mobility group nucleosomal binding domain	1.96
1434130_at	NM_172589	Lhfpl2	lipoma HMGIC fusion partner-like 2	1.96
1433924_at	NM_008817	Peg3	paternally expressed 3	1.96
1448507_at	NM_028889	Efhd1	EF hand domain containing 1	1.96
1455304_at	NM_001081153	Unc13c	unc-13 homolog C (C. elegans)	1.96
1456545_at	NM_010553	Il18rap	interleukin 18 receptor accessory protein	1.96
1452977_at	NM_177263	Zhx3	zinc fingers and homeoboxes 3	1.96
1453677_a_at	NM_024440	Derl3	Der1-like domain family, member 3	1.95
1424790_at	NM_001007570	Slc25a42	solute carrier family 25, member 42	1.95
1457105_at	NM_181422	Pkd2l1	polycystic kidney disease 2-like 1	1.95
1457265_at	NM_001081956	Akap17b	A kinase (PRKA) anchor protein 17B	1.95
1457279_at	XR_106390	LOC100504040	hypothetical LOC100504040	1.94
1444089_at	NM_009260	Spnb2	spectrin beta 2	1.94
1449452_a_at	NM_025989	Gp2	glycoprotein 2 (zymogen granule membrane)	1.94
1426607_at	NM_001039244	Gm7120	predicted gene 7120	1.94
1426258_at	NM_011436	Sorl1	sortilin-related receptor, LDLR class A repeats-	1.94
1420707_a_at	NM_011634	Traip	TRAF-interacting protein	1.94
1434621_at	NM_001001183	Tmem204	transmembrane protein 204	1.93
1415945_at	NM_008566	Mcm5	minichromosome maintenance deficient 5, cell	1.93
1423860_at	NM_008963	Ptgds	prostaglandin D2 synthase (brain)	1.93
1449846_at	NM_007895	Ear2	eosinophil-associated, ribonuclease A family,	1.93
1435987_x_at	BM570681	1110059G02Rik	RIKEN cDNA 1110059G02 gene	1.93
1451899_a_at	NM_001081462	Gtf2ird1	general transcription factor II I repeat domain-	1.93
1422526_at	NM_007981	Acsl1	acyl-CoA synthetase long-chain family member 1	1.93
1450607_s_at	NM_145077	Ucn2	urocortin 2	1.93
1426530_a_at	NM_175174	Klhl5	kelch-like 5 (Drosophila)	1.93
1423488_at	NM_026178	Mmd	monocyte to macrophage differentiation-	1.93
1439128_at	NM_019778	Zbtb20	zinc finger and BTB domain containing 20	1.93
1424089_a_at	NM_001083967	Tcf4	transcription factor 4	1.92
1450650_at	NM_019472	Myo10	myosin X	1.92
1454086_a_at	NM_001142335	Lmo2	LIM domain only 2	1.92
1446812_at	XM_001474844	Gm2818	Predicted gene 2818	1.92
1448606_at	NM_010336	Lpar1	lysophosphatidic acid receptor 1	1.92
1454959_s_at	NM_010305	Gnai1	guanine nucleotide binding protein (G protein),	1.92
1424767_at	NM_174988	Cdh22	cadherin 22	1.92
1439019_at	NM_175473	Fras1	Fraser syndrome 1 homolog (human)	1.91
1421008_at	NM_021384	Rsad2	radical S-adenosyl methionine domain containing	1.91

Probe set	Accession No.	Symbol	Gene Title	Fold Change
1448117_at	NM_013598	Kitl	kit ligand	1.91
1430143_at	AK015205	4930426D05Rik	RIKEN cDNA 4930426D05 gene	1.91
1417426_at	NM_011157	Srgn	serglycin	1.91
1437393_at	NM_011101	Prkca	protein kinase C, alpha	1.91
1456752_at	XR_104769	LOC100503083	hypothetical LOC100503083	1.91
1450276_a_at	NM_001146196	Scin	scinderin	1.91
1454607_s_at	NM_177420	Psat1	phosphoserine aminotransferase 1	1.91
1434875_a_at	NM_026122	Hmgn3	high mobility group nucleosomal binding domain	1.90
1418707_at	NM_026121	Bag4	BCL2-associated athanogene 4	1.90
1425310_a_at	NM_024474	Emid2	EMI domain containing 2	1.90
1418540_a_at	NM_011212	Ptpre	protein tyrosine phosphatase, receptor type, E	1.90
1448119_at	NM_007563	Bpgm	2,3-bisphosphoglycerate mutase	1.89
1416183_a_at	NM_008492	Ldhb	lactate dehydrogenase B	1.89
1429549_at	NM_025685	Col27a1	collagen, type XXVII, alpha 1	1.89
1424659_at	NM_178804	Slit2	slit homolog 2 (Drosophila)	1.89
1424663_at	NM_145430	BC017647	cDNA sequence BC017647	1.89
1425270_at	NM_008441	Kif1b	kinesin family member 1B	1.89
1440911_at	NM_153393	Col23a1	collagen, type XXIII, alpha 1	1.89
1427600_at	BC025871	---		1.88
1427437_at	BC019134	---		1.88
1433971_at	NM_001081557	Camta1	calmodulin binding transcription activator 1	1.88
1455609_at	NM_007708	Cit	citron	1.88
1416724_x_at	NM_001083967	Tcf4	transcription factor 4	1.88
1420028_s_at	NM_008563	Mcm3	minichromosome maintenance deficient 3 (S.	1.87
1449131_s_at	NM_007639	Cd1d1	CD1d1 antigen	1.87
1416343_a_at	NM_001017959	Lamp2	lysosomal-associated membrane protein 2	1.87
1448052_at	NM_026599	Cgnl1	cingulin-like 1	1.87
1419627_s_at	NM_001190320	Clec4n	C-type lectin domain family 4, member n	1.87
1415865_s_at	NM_007563	Bpgm	2,3-bisphosphoglycerate mutase	1.87
1435608_at	NM_001080924	Znrf3	zinc and ring finger 3	1.87
1419486_at	NM_008592	Foxc1	forkhead box C1	1.87
1427912_at	NM_173047	Cbr3	carbonyl reductase 3	1.87
1418219_at	NM_008357	Il15	interleukin 15	1.87
1431704_a_at	NM_001159965	Ralgps2	Ral GEF with PH domain and SH3 binding motif 2	1.86
1426955_at	NM_001109991	Col18a1	collagen, type XVIII, alpha 1	1.86
1454198_a_at	NM_025769	Efcab1	EF hand calcium binding domain 1	1.86
1436539_at	NM_001040682	Clmn	calmin	1.86
1417749_a_at	NM_001163574	Tjp1	tight junction protein 1	1.86
1447819_x_at	NM_007739	Col8a1	collagen, type VIII, alpha 1	1.86
1448136_at	NM_001136077	Enpp2	ectonucleotide	1.85
1427008_at	NM_172448	Rnf43	ring finger protein 43	1.85
1460742_at	NM_145939	Alg3	asparagine-linked glycosylation 3 homolog (yeast,	1.85
1453269_at	NM_029770	Unc5b	unc-5 homolog B (C. elegans)	1.85
1449823_at	NM_001142570	Dach2	dachshund 2 (Drosophila)	1.85
1455663_at	NM_172907	Olfml1	olfactomedin-like 1	1.85

Probe set	Accession No.	Symbol	Gene Title	Fold Change
1419252_at	NM_001159964	Eps15	epidermal growth factor receptor pathway	1.85
1434709_at	NM_001146031	Nrcam	neuron-glia-CAM-related cell adhesion molecule	1.85
1422411_s_at	NM_001012766	BC151093 ///	cDNA sequence BC151093 /// eosinophil-	1.85
1453009_at	NM_027468	Cpm	carboxypeptidase M	1.84
1417190_at	NM_021524	Nampt	nicotinamide phosphoribosyltransferase	1.84
1422064_a_at	NM_019778	Zbtb20	zinc finger and BTB domain containing 20	1.84
1456901_at	NM_001164785	Adamts20	a disintegrin-like and metallopeptidase (reprolysin	1.84
1421072_at	NM_018826	Irx5	Iroquois related homeobox 5 (Drosophila)	1.84
1428853_at	NM_008957	Ptch1	patched homolog 1	1.84
1448916_at	NM_010756	Mafg	v-maf musculoaponeurotic fibrosarcoma	1.84
1424711_at	NM_001033759	Tmem2	transmembrane protein 2	1.84
1434499_a_at	NM_008492	Ldhb	lactate dehydrogenase B	1.84
1416344_at	NM_001017959	Lamp2	lysosomal-associated membrane protein 2	1.84
1434272_at	NM_001177379	Cpeb2	cytoplasmic polyadenylation element binding	1.84
1428834_at	NM_176933	Dusp4	dual specificity phosphatase 4	1.83
1442021_at	NM_010307	Gnal	guanine nucleotide binding protein, alpha	1.83
1435367_at	NM_172632	Mapk4	mitogen-activated protein kinase 4	1.83
1423640_at	NM_001163032	Synpr	synaptoporin	1.83
1429658_a_at	NM_008017	Smc2	structural maintenance of chromosomes 2	1.83
1456640_at	NM_001146299	Sh3rf2	SH3 domain containing ring finger 2	1.83
1448237_x_at	NM_008492	Ldhb	lactate dehydrogenase B	1.83
1453006_at	NM_028263	Fgfbp3	fibroblast growth factor binding protein 3	1.82
1415993_at	NM_009270	Sqle	squalene epoxidase	1.82
1415877_at	NM_001136086	Dpysl3	dihydropyrimidinase-like 3	1.82
1447836_x_at	XR_105537	4921525O09Rik	RIKEN cDNA 4921525O09 gene	1.82
1455235_x_at	NM_008492	Ldhb	lactate dehydrogenase B	1.82
1454926_at	NM_172430	Sphkap	SPHK1 interactor, AKAP domain containing	1.82
1453840_at	NM_008774	Pabpc1	poly(A) binding protein, cytoplasmic 1	1.82
1416840_at	NM_001166635	Mid1ip1	Mid1 interacting protein 1 (gastrulation specific	1.82
1452951_at	NM_001162906	2410089E03Rik	RIKEN cDNA 2410089E03 gene	1.82
1434484_at	NM_183249	1100001G20Rik	RIKEN cDNA 1100001G20 gene	1.82
1443485_at	NM_001122889	Epha7	Eph receptor A7	1.82
1437718_x_at	NM_021355	Fmod	fibromodulin	1.81
1417420_at	NM_007631	Ccnd1	cyclin D1	1.81
1459713_s_at	NM_178642	Ano1	anoctamin 1, calcium activated chloride channel	1.81
1437685_x_at	NM_021355	Fmod	fibromodulin	1.81
1454890_at	NM_153319	Amot	angiomotin	1.81
1419724_at	NM_010100	Edar	ectodysplasin-A receptor	1.81
1424969_s_at	NM_029692	Upp2	uridine phosphorylase 2	1.80
1460070_at	BB177017	---		1.80
1441111_at	NM_001166030	Mylk4	myosin light chain kinase family, member 4	1.80
1450646_at	NM_020010	Cyp51	cytochrome P450, family 51	1.80
1445597_s_at	NM_139269	Pla2g16	phospholipase A2, group XVI	1.80
1453002_at	NM_009234	Sox11	SRY-box containing gene 11	1.80
1431335_a_at	NM_023395	Wfdc1	WAP four-disulfide core domain 1	1.80

Probe set	Accession No.	Symbol	Gene Title	Fold Change
1451457_at	NM_172769	Sc5d	sterol-C5-desaturase (fungal ERG3, delta-5-	1.80
1420394_s_at	NM_008147	Gp49a /// Lilrb4	glycoprotein 49 A /// leukocyte immunoglobulin-	1.80
1422024_at	NM_008026	Fli1	Friend leukemia integration 1	1.79
1423250_a_at	NM_009367	Tgfb2	transforming growth factor, beta 2	1.79
1443471_at	NM_019778	Zbtb20	zinc finger and BTB domain containing 20	1.79
1434360_s_at	NM_008981	Ptprg	protein tyrosine phosphatase, receptor type, G	1.79
1424097_at	NM_029001	Elovl7	ELOVL family member 7, elongation of long chain	1.79
1415855_at	NM_013598	Kitl	kit ligand	1.79
1436698_x_at	NM_001001183	Tmem204	transmembrane protein 204	1.79
1450080_at	NM_028375	Cxx1c	CAAX box 1 homolog C (human)	1.79
1417625_s_at	NM_007722	Cxcr7	chemokine (C-X-C motif) receptor 7	1.78
1448975_s_at	NM_031192	Ren1 /// Ren2	renin 1 structural /// renin 2 tandem duplication	1.78
1428433_at	NM_001136065	Hipk2	homeodomain interacting protein kinase 2	1.78
1417040_a_at	NM_016778	Bok	BCL2-related ovarian killer protein	1.78
1451611_at	NM_139269	Pla2g16	phospholipase A2, group XVI	1.78
1418926_at	NM_011546	Zeb1	zinc finger E-box binding homeobox 1	1.78
1418261_at	NM_011518	Sykb	spleen tyrosine kinase	1.78
1442659_at	NM_001081377	Pcdh9	protocadherin 9	1.78
1446953_at	BG073799	---		1.78
1448777_at	NM_008564	Mcm2	minichromosome maintenance deficient 2 mitotin	1.77
1429210_at	NM_153393	Col23a1	collagen, type XXIII, alpha 1	1.77
1452226_at	NM_173867	Rcc2	regulator of chromosome condensation 2	1.77
1436593_at	NM_198637	1700016K19Rik	RIKEN cDNA 1700016K19 gene	1.77
1418190_at	NM_011134	Pon1	paraoxonase 1	1.77
1431028_a_at	NM_001114339	Pank1	pantothenate kinase 1	1.77
1422483_a_at	NM_007808	Cycs	cytochrome c, somatic	1.77
1446294_at	NM_011544	Tcf12	Transcription factor 12	1.77
1449198_a_at	NM_001035228	St3gal5	ST3 beta-galactoside alpha-2,3-sialyltransferase 5	1.77
1417047_at	NM_138750	Prom2	prominin 2	1.76
1420056_s_at	NM_033398	Jmjd6	jumonji domain containing 6	1.76
1420549_at	NM_010259	Gbp1	guanylate binding protein 1	1.76
1434411_at	NM_007730	Col12a1	collagen, type XII, alpha 1	1.76
1423594_a_at	NM_001136061	Ednrb	endothelin receptor type B	1.76
1437422_at	NM_009154	Sema5a	sema domain, seven thrombospondin repeats	1.76
1421354_at	NM_008926	Prkg2	protein kinase, cGMP-dependent, type II	1.76
1438966_x_at	NM_021355	Fmod	fibromodulin	1.76
1441170_a_at	NM_001001602	Dab2ip	disabled homolog 2 (Drosophila) interacting	1.76
1418931_at	NM_026328	Reg4	regenerating islet-derived family, member 4	1.76
1460695_a_at	NM_028079	2010111I01Rik	RIKEN cDNA 2010111I01 gene	1.76
1421299_a_at	NM_010703	Lef1	lymphoid enhancer binding factor 1	1.75
1435848_at	NM_001033347	D430041D05Rik	RIKEN cDNA D430041D05 gene	1.75
1439278_at	NM_019778	Zbtb20	zinc finger and BTB domain containing 20	1.75
1449522_at	NM_009472	Unc5c	unc-5 homolog C (C. elegans)	1.75
1436470_at	NM_053271	Rims2	regulating synaptic membrane exocytosis 2	1.75
1450779_at	NM_021272	Fabp7	fatty acid binding protein 7, brain	1.75

Probe set	Accession No.	Symbol	Gene Title	Fold Change
1429552_at	NM_027963	Wdr16	WD repeat domain 16	1.75
1426571_at	NM_178642	Ano1	anoctamin 1, calcium activated chloride channel	1.75
1453128_at	NM_053247	Lyve1	lymphatic vessel endothelial hyaluronan receptor	1.74
1435036_at	NM_001081169	Aspg	asparaginase homolog (S. cerevisiae)	1.74
1439024_at	NM_026121	Bag4	BCL2-associated athanogene 4	1.74
1424761_at	NM_146174	Fam115c	family with sequence similarity 115, member C	1.74
1417871_at	NM_010476	Hsd17b7	hydroxysteroid (17-beta) dehydrogenase 7	1.74
1426044_a_at	NM_008859	Prkcq	protein kinase C, theta	1.74
1417144_at	NM_134024	Tubg1	tubulin, gamma 1	1.74
1417686_at	NM_019516	Lgals12	lectin, galactose binding, soluble 12	1.74
1458375_at	NM_001033531	Klhl32	kelch-like 32 (Drosophila)	1.74
1433443_a_at	NM_145942	Hmgcs1	3-hydroxy-3-methylglutaryl-Coenzyme A synthase	1.74
1443939_at	NM_001085549	Gm12824	predicted gene 12824	1.74
1429651_at	NM_001007154	Phactr3	phosphatase and actin regulator 3	1.74
1422912_at	NM_007554	Bmp4	bone morphogenetic protein 4	1.73
1453623_a_at	NM_009010	Rad23a	RAD23a homolog (S. cerevisiae)	1.73
1429214_at	NM_029981	Adamtsl2	ADAMTS-like 2	1.73
1449130_at	NM_007639	Cd1d1	CD1d1 antigen	1.73
1423478_at	NM_008855	Prkcb	protein kinase C, beta	1.73
1438761_a_at	NM_013614	Odc1	ornithine decarboxylase, structural 1	1.73
1460036_at	NM_026887	Ap1s2	adaptor-related protein complex 1, sigma 2	1.73
1426514_at	NM_029935	Chst15	carbohydrate (N-acetylgalactosamine 4-sulfate 6-	1.73
1440275_at	NM_019732	Runx3	runt related transcription factor 3	1.73
1455542_at	NR_027923	C630043F03Rik	RIKEN cDNA C630043F03 gene	1.73
1418773_at	NM_021890	Fads3	fatty acid desaturase 3	1.73
1437598_at	NM_019778	Zbtb20	zinc finger and BTB domain containing 20	1.73
1448754_at	NM_011254	Rbp1	retinol binding protein 1, cellular	1.73
1436941_at	NM_001134457	Fam55c	family with sequence similarity 55, member C	1.73
1419004_s_at	NM_007534	Bcl2a1a ///	B-cell leukemia/lymphoma 2 related protein A1a	1.73
1422504_at	NM_010298	Glrb	glycine receptor, beta subunit	1.73
1456812_at	NM_011994	Abcd2	ATP-binding cassette, sub-family D (ALD),	1.73
1416178_a_at	NM_001163182	Plekhb1	pleckstrin homology domain containing, family B	1.73
1429413_at	NM_027468	Cpm	carboxypeptidase M	1.72
1427980_at	NM_001163262	4933407C03Rik	RIKEN cDNA 4933407C03 gene	1.72
1418539_a_at	NM_011212	Ptpre	protein tyrosine phosphatase, receptor type, E	1.72
1417732_at	NM_013473	Anxa8	annexin A8	1.72
1448698_at	NM_007631	Ccnd1	cyclin D1	1.72
1418436_at	NM_016797	Stx7	syntaxin 7	1.72
1418331_at	NM_025402	1110031I02Rik	RIKEN cDNA 1110031I02 gene	1.72
1436499_at	NM_001168525	Sgms1	sphingomyelin synthase 1	1.72
1418774_a_at	NM_001109757	Atp7a	ATPase, Cu++ transporting, alpha polypeptide	1.72
1452887_at	NM_028718	Traf3ip1	TRAF3 interacting protein 1	1.72
1450875_at	NM_010338	Gpr37	G protein-coupled receptor 37	1.72
1424886_at	NM_001014288	Ptprd	protein tyrosine phosphatase, receptor type, D	1.72
1421286_a_at	NM_018731	Atp4a	ATPase, H+/K+ exchanging, gastric, alpha	1.72

Probe set	Accession No.	Symbol	Gene Title	Fold Change
1430448_at	XM_001005301	6720418B01Rik	RIKEN cDNA 6720418B01 gene	1.72
1422852_at	NM_019686	Cib2	calcium and integrin binding family member 2	1.72
1427118_at	XM_003085066	5430421N21Rik	RIKEN cDNA 5430421N21 gene	1.71
1422432_at	NM_001037999	Dbi	diazepam binding inhibitor	1.71
1460187_at	NM_013834	Sfrp1	secreted frizzled-related protein 1	1.71
1440564_at	NM_144944	Prokr2	prokineticin receptor 2	1.71
1453129_a_at	NM_001163512	Rgs12	regulator of G-protein signaling 12	1.71
1437324_x_at	NM_021355	Fmod	fibromodulin	1.71
1454969_at	NM_177139	Lypd6	LY6/PLAUR domain containing 6	1.71
1438081_at	NM_001085373	Mcc	mutated in colorectal cancers	1.71
1419665_a_at	NM_019738	Nupr1	nuclear protein 1	1.71
1427364_a_at	NM_013614	Odc1	ornithine decarboxylase, structural 1	1.71
1416835_s_at	NM_009665	Amd1	S-adenosylmethionine decarboxylase 1	1.71
1436808_x_at	NM_008566	Mcm5	minichromosome maintenance deficient 5, cell	1.71
1418260_at	NM_015755	Hunk	hormonally upregulated Neu-associated kinase	1.70
1430762_at	NM_177702	4833427G06Rik	RIKEN cDNA 4833427G06 gene	1.70
1452118_at	NM_001163734	Rrp1b	ribosomal RNA processing 1 homolog B (S.	1.70
1433615_at	NM_178789	Tmem117	transmembrane protein 117	1.70
1431416_a_at	NM_023844	Jam2	junction adhesion molecule 2	1.70
1435021_at	NM_001038701	Gabrb3	gamma-aminobutyric acid (GABA) A receptor,	1.70
1433968_a_at	NM_172694	Megf9	multiple EGF-like-domains 9	1.70
1425282_at	NM_001170643	Rnf144b	ring finger protein 144B	1.70
1450973_s_at	NM_011941	Mapkbp1	mitogen-activated protein kinase binding protein	1.70
1430779_at	NM_001163348	Ntng1	netrin G1	1.70
1448346_at	NM_007687	Cfl1	cofilin 1, non-muscle	1.70
1420855_at	NM_007925	Eln	elastin	1.70
1437996_s_at	NM_175651	Cnpy1	canopy 1 homolog (zebrafish)	1.70
1437479_x_at	NM_011535	Tbx3	T-box 3	1.70
1448769_at	NM_016752	Slc35b1	solute carrier family 35, member B1	1.70
1422699_at	NM_007440	Alox12	arachidonate 12-lipoxygenase	1.69
1422607_at	NM_001163154	Etv1	ets variant gene 1	1.69
1426282_at	NM_172290	Ntm	neurotrimin	1.69
1416723_at	NM_001083967	Tcf4	transcription factor 4	1.69
1427059_at	NM_172608	Tmem184b	transmembrane protein 184b	1.69
1450391_a_at	NM_001166249	Mgll	monoglyceride lipase	1.69
1437417_s_at	NM_001079844	Gpc6	glypican 6	1.69
1430533_a_at	NM_001165902	Ctnnb1	catenin (cadherin associated protein), beta 1	1.68
1426576_at	NM_001168525	Sgms1	sphingomyelin synthase 1	1.68
1433855_at	NM_001170978	Abat	4-aminobutyrate aminotransferase	1.68
1456003_a_at	NM_018861	Slc1a4	solute carrier family 1 (glutamate/neutral amino	1.68
1443601_at	NM_001033791	Gm12888	Predicted gene 12888	1.68
1449455_at	NM_001172117	Hck	hemopoietic cell kinase	1.68
1460419_a_at	NM_008855	Prkcb	protein kinase C, beta	1.68
1423186_at	NM_001122998	Tiam2	T-cell lymphoma invasion and metastasis 2	1.68
1453836_a_at	NM_001166249	Mgll	monoglyceride lipase	1.68

Probe set	Accession No.	Symbol	Gene Title	Fold Change
1436233_at	NM_172793	Btnl9	butyrophilin-like 9	1.68
1426510_at	NM_178653	Sccpdh	saccharopine dehydrogenase (putative)	1.68
1443119_at	NM_177328	Grm7	glutamate receptor, metabotropic 7	1.67
1418237_s_at	NM_001109991	Col18a1	collagen, type XVIII, alpha 1	1.67
1418982_at	NM_007678	Cebpa	CCAAT/enhancer binding protein (C/EBP), alpha	1.67
1434427_a_at	NM_027258	Rnf157	ring finger protein 157	1.67
1459847_x_at	NM_008115	Gfra2	glial cell line derived neurotrophic factor family	1.67
1418835_at	NM_009344	Phlda1	pleckstrin homology-like domain, family A,	1.67
1422820_at	NM_001039507	Lipe	lipase, hormone sensitive	1.67
1434320_at	NM_001166033	Gtf3c4	general transcription factor IIIC, polypeptide 4	1.67
1419814_s_at	NM_011309	S100a1	S100 calcium binding protein A1	1.67
1437933_at	NM_020259	Hhip	Hedgehog-interacting protein	1.67
1437471_at	NM_153545	Lrrc45	leucine rich repeat containing 45	1.67
1452092_at	NM_029935	Chst15	carbohydrate (N-acetylgalactosamine 4-sulfate 6-	1.67
1449089_at	NM_173440	Nrip1	nuclear receptor interacting protein 1	1.67
1426251_at	NM_153107	Cpz	carboxypeptidase Z	1.67
1459838_s_at	NM_001017525	Btbd11	BTB (POZ) domain containing 11	1.67
1449705_x_at	NM_008563	Mcm3	minichromosome maintenance deficient 3 (S.	1.67
1447930_at	NM_013815	Baz1a ///	bromodomain adjacent to zinc finger domain 1A	1.66
1451548_at	NM_029692	Upp2	uridine phosphorylase 2	1.66
1433445_x_at	NM_145942	Hmgcs1	3-hydroxy-3-methylglutaryl-Coenzyme A synthase	1.66
1422545_at	NM_009324	Tbx2	T-box 2	1.66
1427195_at	NM_007885	Slc26a2	solute carrier family 26 (sulfate transporter),	1.66
1419606_a_at	NM_011618	Tnnt1	troponin T1, skeletal, slow	1.66
1455383_at	NM_001033478	Fam47e	family with sequence similarity 47, member E	1.66
1447311_at	NM_009209	Slc6a2	solute carrier family 6 (neurotransmitter	1.66
1426896_at	NM_021559	Zfp191	zinc finger protein 191	1.66
1417695_a_at	NM_009230	Soat1	sterol O-acyltransferase 1	1.66
1437864_at	NM_197985	Adipor2	adiponectin receptor 2	1.66
1434149_at	NM_001083967	Tcf4	transcription factor 4	1.65
1436576_at	NM_175449	Fam26f	family with sequence similarity 26, member F	1.65
1423499_at	NM_026408	Sncaip	synuclein, alpha interacting protein (synphilin)	1.65
1452646_at	NM_178111	Trp53inp2	transformation related protein 53 inducible	1.65
1436514_at	NM_008150	Gpc4	glypican 4	1.65
1429637_at	NM_133187	Fam198b	family with sequence similarity 198, member B	1.65
1436530_at	NM_001081957	Gm11428	predicted gene 11428	1.65
1417094_at	NM_001146057	Acot7	acyl-CoA thioesterase 7	1.65
1430128_a_at	NM_139292	Reep6	receptor accessory protein 6	1.65
1440729_at	NM_001159964	Eps15	epidermal growth factor receptor pathway	1.65
1422484_at	NM_007808	Cycs	cytochrome c, somatic	1.65
1428792_at	NM_001164369	Bcas1	breast carcinoma amplified sequence 1	1.65
1423489_at	NM_026178	Mmd	monocyte to macrophage differentiation-	1.65
1429682_at	NM_001142952	Fam46c	family with sequence similarity 46, member C	1.65
1434196_at	NM_021422	Dnaja4	DnaJ (Hsp40) homolog, subfamily A, member 4	1.65
1451547_at	NM_027391	Iyd	iodotyrosine deiodinase	1.65

Probe set	Accession No.	Symbol	Gene Title	Fold Change
1427167_at	XM_003086849	Armcx4 ///	armadillo repeat containing, X-linked 4 ///	1.65
1423503_at	NM_023277	Jam3	junction adhesion molecule 3	1.65
1454295_at	NM_173440	Nrip1	nuclear receptor interacting protein 1	1.64
1449002_at	NM_013750	Phlda3	pleckstrin homology-like domain, family A,	1.64
1456022_at	NM_001136065	Hipk2	homeodomain interacting protein kinase 2	1.64
1440206_at	XR_106121	A930024E05Rik	RIKEN cDNA A930024E05 gene	1.64
1422788_at	NM_021398	Slc43a3	solute carrier family 43, member 3	1.64
1451344_at	NM_146162	Tmem119	transmembrane protein 119	1.64
1437421_at	BB332988	6330509M05Rik	RIKEN cDNA 6330509M05 gene	1.64
1428026_at	NM_080455	Tshz2	teashirt zinc finger family member 2	1.64
1440759_at	BB321369	Gm10672	predicted gene 10672	1.64
1458492_x_at	NM_172290	Ntm	neurotrimin	1.64
1450922_a_at	NM_009367	Tgfb2	transforming growth factor, beta 2	1.64
1452365_at	NM_172753	Csgalnact1	chondroitin sulfate N-	1.64
1419708_at	NM_009526	Wnt6	wingless-related MMTV integration site 6	1.64
1419282_at	NM_011331	Ccl12	chemokine (C-C motif) ligand 12	1.64
1430152_at	NM_001159964	Eps15	epidermal growth factor receptor pathway	1.64
1443870_at	NM_001033336	Abcc4	ATP-binding cassette, sub-family C (CFTR/MRP),	1.64
1449291_a_at	NM_025705	Dcbld1	discoidin, CUB and LCCL domain containing 1	1.64
1419479_at	NM_026907	Sectm1b	secreted and transmembrane 1B	1.64
1436528_at	NM_178929	Kazald1	Kazal-type serine peptidase inhibitor domain 1	1.64
1423877_at	NM_028083	Chaf1b	chromatin assembly factor 1, subunit B (p60)	1.64
1458512_at	NM_001083927	Tle3	transducin-like enhancer of split 3, homolog of	1.63
1437309_a_at	NM_001164223	Rpa1	replication protein A1	1.63
1436509_at	NM_175403	Mlec	malectin	1.63
1426446_at	NM_001163760	6430548M08Rik	RIKEN cDNA 6430548M08 gene	1.63
1416480_a_at	NM_001112668	Gm9790 ///	predicted gene 9790 /// HIG1 domain family,	1.63
1446923_at	BQ175779	---		1.63
1426968_a_at	NM_133832	Rdh10	retinol dehydrogenase 10 (all-trans)	1.63
1422831_at	NM_010181	Fbn2	fibrillin 2	1.63
1448647_at	NM_008549	Man2a1	mannosidase 2, alpha 1	1.63
1451352_s_at	NM_001171052	Mta3	metastasis associated 3	1.63
1419300_at	NM_010228	Flt1	FMS-like tyrosine kinase 1	1.63
1451529_at	NM_144838	Sgtb	small glutamine-rich tetratricopeptide repeat	1.63
1448443_at	NM_009250	Serpini1	serine (or cysteine) peptidase inhibitor, clade I,	1.63
1423685_at	NM_146217	Aars	alanyl-tRNA synthetase	1.63
1456117_at	NM_001163734	Rrp1b	ribosomal RNA processing 1 homolog B (S.	1.63
1443854_at	NM_010402	Hand2	heart and neural crest derivatives expressed	1.63
1427205_x_at	NM_029586	Ccdc46	coiled-coil domain containing 46	1.62
1455785_at	NM_010595	Kcna1	potassium voltage-gated channel, shaker-related	1.62
1439041_at	NM_172653	Slc39a10	solute carrier family 39 (zinc transporter), member	1.62
1420699_at	NM_020008	Clec7a	C-type lectin domain family 7, member a	1.62
1438431_at	NM_011994	Abcd2	ATP-binding cassette, sub-family D (ALD),	1.62
1460062_at	NM_177606	Plekhh2	pleckstrin homology domain containing, family H	1.62
1448395_at	NM_013834	Sfrp1	secreted frizzled-related protein 1	1.62

Probe set	Accession No.	Symbol	Gene Title	Fold Change
1424762_at	NM_001040631	C1qtnf5 /// Mfrp	C1q and tumor necrosis factor related protein 5	1.62
1448433_a_at	NM_008788	Pcolce	procollagen C-endopeptidase enhancer protein	1.62
1424471_at	NM_001177810	Rapgef3	Rap guanine nucleotide exchange factor (GEF) 3	1.62
1424098_at	NM_029001	Elovl7	ELOVL family member 7, elongation of long chain	1.62
1452771_s_at	NM_001033606	Acsl3	acyl-CoA synthetase long-chain family member 3	1.62
1434856_at	NM_001081433	Ankrd44	ankyrin repeat domain 44	1.62
1453418_at	NM_027770	Col24a1	collagen, type XXIV, alpha 1	1.61
1419666_x_at	NM_019738	Nupr1	nuclear protein 1	1.61
1437284_at	NM_021457	Fzd1	frizzled homolog 1 (Drosophila)	1.61
1423293_at	NM_001164223	Rpa1	replication protein A1	1.61
1460247_a_at	NM_013787	Skp2	S-phase kinase-associated protein 2 (p45)	1.61
1421088_at	NM_008150	Gpc4	glypican 4	1.61
1432176_a_at	NM_001146348	Eng	endoglin	1.61
1453008_at	NM_001081156	Trnp1	TMF1-regulated nuclear protein 1	1.61
1419421_at	NM_001110783	Ank1	ankyrin 1, erythroid	1.61
1451827_a_at	NM_015760	Nox4	NADPH oxidase 4	1.61
1422533_at	NM_020010	Cyp51	cytochrome P450, family 51	1.61
1439849_at	NM_001085549	Gm12824	predicted gene 12824	1.61
1421063_s_at	NM_001082961	Snrpn /// Snurf	small nuclear ribonucleoprotein N /// SNRPN	1.61
1440056_at	BB426260	---		1.61
1418969_at	NM_013787	Skp2	S-phase kinase-associated protein 2 (p45)	1.61
1426657_s_at	NM_016966	Gm8096 ///	3-phosphoglycerate dehydrogenase pseudogene	1.61
1418484_at	NM_011902	Tekt2	tektin 2	1.61
1435323_a_at	NM_153546	Mboat1	membrane bound O-acyltransferase domain	1.61
1439396_x_at	NM_010271	Gpd1	glycerol-3-phosphate dehydrogenase 1 (soluble)	1.61
1421830_at	NM_001177602	Ak4	adenylate kinase 4	1.61
1419112_at	NM_008702	Nlk	nemo like kinase	1.61
1418910_at	NM_007557	Bmp7	bone morphogenetic protein 7	1.61
1420981_a_at	NM_001161769	Lmo4	LIM domain only 4	1.61
1450010_at	NM_019657	Hsd17b12	hydroxysteroid (17-beta) dehydrogenase 12	1.61
1442368_at	NM_175429	Kctd12b	potassium channel tetramerisation domain	1.61
1449991_at	NM_018729	Cd244	CD244 natural killer cell receptor 2B4	1.60
1422864_at	NM_001111021	Runx1	runt related transcription factor 1	1.60
1448949_at	NM_007607	Car4	carbonic anhydrase 4	1.60
1425140_at	NM_145381	Lactb2	lactamase, beta 2	1.60
1451117_a_at	NM_028011	Tom1l1	target of myb1-like 1 (chicken)	1.60
1419599_s_at	NM_026835	Ms4a6d	membrane-spanning 4-domains, subfamily A,	1.60
1456769_at	NM_028207	Dusp3	dual specificity phosphatase 3 (vaccinia virus	1.60
1442144_at	NM_080595	Emid1	EMI domain containing 1	1.60
1434054_at	NM_010756	Mafg	v-maf musculoaponeurotic fibrosarcoma	1.60
1453855_at	NM_026280	Mxra7	matrix-remodelling associated 7	1.60
1418243_at	NM_007995	Fcna	ficolin A	1.60
1417400_at	NM_001166408	Rai14	retinoic acid induced 14	1.60
1442308_at	NM_001102611	Smyd4	SET and MYND domain containing 4	1.60
1426805_at	NM_001174078	Smarca4	SWI/SNF related, matrix associated, actin	1.60

Probe set	Accession No.	Symbol	Gene Title	Fold Change
1421844_at	NM_001159317	Il1rap	interleukin 1 receptor accessory protein	1.60
1435472_at	NM_032396	Kremen1	kringle containing transmembrane protein 1	1.59
1450813_a_at	NM_001112702	Tnni1	troponin I, skeletal, slow 1	1.59
1429830_a_at	NM_001111060	Cd59a	CD59a antigen	1.59
1454930_at	NM_173038	Tbcel	tubulin folding cofactor E-like	1.59
1453419_at	NM_008624	Mras	muscle and microspikes RAS	1.59
1444512_at	NM_172525	Arhgap29	Rho GTPase activating protein 29	1.59
1449372_at	NM_008929	Dnajc3	DnaJ (Hsp40) homolog, subfamily C, member 3	1.59
1456071_a_at	NM_007808	Cycs	cytochrome c, somatic	1.59
1436030_at	NM_198037	Cachd1	cache domain containing 1	1.59
1453191_at	NM_025685	Col27a1	collagen, type XXVII, alpha 1	1.59
1453582_at	NM_001025566	Chka	choline kinase alpha	1.59
1421252_a_at	NM_001033713	Mef2a	myocyte enhancer factor 2A	1.59
1460412_at	NM_024237	Fbln7	fibulin 7	1.58
1433446_at	NM_145942	Hmgcs1	3-hydroxy-3-methylglutaryl-Coenzyme A synthase	1.58
1418248_at	NM_013463	Gla	galactosidase, alpha	1.58
1449590_a_at	NM_008624	Mras	muscle and microspikes RAS	1.58
1456406_at	XR_106194	Gm14964	predicted gene 14964	1.58
1432129_a_at	NM_001025570	Prrx1	paired related homeobox 1	1.58
1437650_at	NM_175537	C730026J16 ///	hypothetical protein C730026J16 /// zinc finger	1.58
1449559_at	NM_013601	Msx2	homeobox, msh-like 2	1.58
1417434_at	NM_001145820	Gpd2	glycerol phosphate dehydrogenase 2,	1.58
1449586_at	NM_019645	Pkp1	plakophilin 1	1.58
1448330_at	NM_010358	Gstm1	glutathione S-transferase, mu 1	1.58
1415854_at	NM_013598	Kitl	kit ligand	1.58
1431179_at	NM_053103	Entpd7	ectonucleoside triphosphate diphosphohydrolase	1.58
1456080_a_at	NM_012032	Serinc3	serine incorporator 3	1.58
1416702_at	NM_009250	Serpini1	serine (or cysteine) peptidase inhibitor, clade I,	1.58
1423627_at	NM_008706	Nqo1	NAD(P)H dehydrogenase, quinone 1	1.58
1444176_at	NM_175406	Atp6v0d2	ATPase, H+ transporting, lysosomal V0 subunit	1.58
1457724_at	NM_009984	Ctsl	cathepsin L	1.58
1438040_a_at	NM_011631	Hsp90b1	heat shock protein 90, beta (Grp94), member 1	1.57
1422631_at	NM_013464	Ahr	aryl-hydrocarbon receptor	1.57
1427527_a_at	NM_008970	Pthlh	parathyroid hormone-like peptide	1.57
1443824_s_at	NM_053070	Car7	carbonic anhydrase 7	1.57
1425225_at	NM_144559	Fcgr4	Fc receptor, IgG, low affinity IV	1.57
1453015_at	NM_001163262	4933407C03Rik	RIKEN cDNA 4933407C03 gene	1.57
1450106_a_at	NM_001163394	Evl	Ena-vasodilator stimulated phosphoprotein	1.57
1421594_a_at	NM_001040085	Sytl2	synaptotagmin-like 2	1.57
1460241_a_at	NM_001035228	St3gal5	ST3 beta-galactoside alpha-2,3-sialyltransferase 5	1.57
1428869_at	NM_001039351	Nolc1	nucleolar and coiled-body phosphoprotein 1	1.57
1436316_at	NM_021366	Klf13	Kruppel-like factor 13	1.57
1424653_at	NM_197996	Tspan15	tetraspanin 15	1.57
1433444_at	NM_145942	Hmgcs1	3-hydroxy-3-methylglutaryl-Coenzyme A synthase	1.57
1426813_at	NM_181470	Ltv1	LTV1 homolog (S. cerevisiae)	1.57

Probe set	Accession No.	Symbol	Gene Title	Fold Change
1433653_at	NM_153782	Fam20a	family with sequence similarity 20, member A	1.56
1448390_a_at	NM_001172424	Dhrs3	dehydrogenase/reductase (SDR family) member 3	1.56
1460330_at	NM_013470	Anxa3	annexin A3	1.56
1427277_at	NM_009189	Six1	sine oculis-related homeobox 1 homolog	1.56
1425784_a_at	NM_001038612	Olfm1	olfactomedin 1	1.56
1424318_at	NM_173752	1110067D22Rik	RIKEN cDNA 1110067D22 gene	1.56
1451394_at	NM_001136060	Dpp6	dipeptidylpeptidase 6	1.56
1452240_at	NM_001146292	Celf4	CUGBP, Elav-like family member 4	1.56
1426110_a_at	NM_010336	Lpar1	lysophosphatidic acid receptor 1	1.56
1444946_at	BG072280	---		1.56
1416199_at	NM_001145831	Kifc3	kinesin family member C3	1.56
1422964_at	NM_009010	Rad23a	RAD23a homolog (S. cerevisiae)	1.56
1422620_s_at	NM_008247	Ppap2a	phosphatidic acid phosphatase type 2A	1.56
1452317_at	NM_008270	Hoxb9	homeobox B9	1.56
1451322_at	NM_181588	Cmbl	carboxymethylenebutenolidase-like	1.56
1447742_at	NM_010686	Laptm5	lysosomal-associated protein transmembrane 5	1.56
1454606_at	NM_178682	4933426M11Rik	RIKEN cDNA 4933426M11 gene	1.56
1419692_a_at	NM_008521	Ltc4s	leukotriene C4 synthase	1.56
1416235_at	NM_133807	Lrrc59	leucine rich repeat containing 59	1.55
1427670_a_at	NM_011544	Tcf12	transcription factor 12	1.55
1424367_a_at	NM_001164086	Homer2	homer homolog 2 (Drosophila)	1.55
1437445_at	NM_001039104	Trpm1	transient receptor potential cation channel,	1.55
1424110_a_at	NM_008704	Nme1	non-metastatic cells 1, protein (NM23A)	1.55
1430781_at	NM_030187	Ak7	adenylate kinase 7	1.55
1415999_at	NM_010423	Hey1	hairy/enhancer-of-split related with YRPW motif 1	1.55
1452474_a_at	NM_181728	Art3	ADP-ribosyltransferase 3	1.55
1448397_at	NM_001010937	Gjb6	gap junction protein, beta 6	1.55
1434046_at	NM_001004174	AA467197	expressed sequence AA467197	1.55
1455976_x_at	NM_001037999	Dbi	diazepam binding inhibitor	1.55
1455689_at	NM_175284	Fzd10	frizzled homolog 10 (Drosophila)	1.55
1455074_at	NM_025769	Efcab1	EF hand calcium binding domain 1	1.55
1442058_s_at	NM_008949	Psmc3ip	proteasome (prosome, macropain) 26S subunit,	1.55
1431591_s_at	NM_015783	Gm9706 ///	predicted gene 9706 /// ISG15 ubiquitin-like	1.55
1423396_at	NM_007428	Agt	angiotensinogen (serpin peptidase inhibitor, clade	1.55
1448729_a_at	NM_011129	41156	septin 4	1.55
1431798_a_at	NM_027875	Syde1	synapse defective 1, Rho GTPase, homolog 1 (C.	1.54
1449030_at	NM_001111015	Syn2	synapsin II	1.54
1422474_at	NM_001177980	Pde4b	phosphodiesterase 4B, cAMP specific	1.54
1452011_a_at	NM_026430	Uxs1	UDP-glucuronate decarboxylase 1	1.54
1428384_at	NM_026821	D4Bwg0951e	DNA segment, Chr 4, Brigham & Women's	1.54
1428821_at	NM_026212	Agpat2	1-acylglycerol-3-phosphate O-acyltransferase 2	1.54
1438030_at	NM_001166493	Rasgrp3	RAS, guanyl releasing protein 3	1.54
1441831_x_at	NM_016691	Clcn5	chloride channel 5	1.54
1423816_at	NM_001018063	Cxx1a /// Cxx1b	CAAX box 1 homolog A (human) /// CAAX box 1	1.54
1426219_at	NM_011327	Scp2	sterol carrier protein 2, liver	1.54

Probe set	Accession No.	Symbol	Gene Title	Fold Change
1429907_at	NM_028567	1700094D03Rik	RIKEN cDNA 1700094D03 gene	1.54
1418061_at	NM_013589	Ltbp2	latent transforming growth factor beta binding	1.54
1421093_at	NM_017394	Slc7a10	solute carrier family 7 (cationic amino acid	1.54
1453084_s_at	NM_027174	Col22a1	collagen, type XXII, alpha 1	1.54
1444242_at	NM_033314	Slco2a1	Solute carrier organic anion transporter family,	1.54
1422619_at	NM_008247	Ppap2a	phosphatidic acid phosphatase type 2A	1.54
1421834_at	NM_008846	Pip5k1b	phosphatidylinositol-4-phosphate 5-kinase, type 1	1.54
1434399_at	NM_001161767	Galnt6	UDP-N-acetyl-alpha-D-galactosamine:polypeptide	1.54
1423047_at	NM_023764	Tollip	toll interacting protein	1.54
1423563_at	NM_030890	Prrt1	proline-rich transmembrane protein 1	1.54
1448564_at	NM_011870	Cib1	calcium and integrin binding 1 (calmyrin)	1.54
1434326_x_at	NM_175484	Coro2b	coronin, actin binding protein, 2B	1.54
1434263_at	NM_028657	F630110N24Rik	RIKEN cDNA F630110N24 gene	1.54
1437012_x_at	NM_001177810	Rapgef3	Rap guanine nucleotide exchange factor (GEF) 3	1.54
1418469_at	NM_173440	Nrip1	nuclear receptor interacting protein 1	1.54
1428354_at	NM_001080932	Foxk2	forkhead box K2	1.54
1416416_x_at	NM_010358	Gstm1	glutathione S-transferase, mu 1	1.53
1420334_at	NM_001083902	Slc12a8	solute carrier family 12 (potassium/chloride	1.53
1451716_at	NM_010658	Mafb	v-maf musculoaponeurotic fibrosarcoma	1.53
1424863_a_at	NM_001136065	Hipk2	homeodomain interacting protein kinase 2	1.53
1456555_at	NM_181816	Ccdc67	coiled-coil domain containing 67	1.53
1453087_at	AK018107	6330403L08Rik	RIKEN cDNA 6330403L08 gene	1.53
1438133_a_at	NM_010516	Cyr61	cysteine rich protein 61	1.53
1417411_at	NM_021432	Nap1l5	nucleosome assembly protein 1-like 5	1.53
1420401_a_at	NM_019511	Ramp3	receptor (calcitonin) activity modifying protein 3	1.53
1435537_at	NM_001014288	Ptprd	protein tyrosine phosphatase, receptor type, D	1.53
1457508_at	BB410003	C430003N24Rik	RIKEN cDNA C430003N24 gene	1.53
1418715_at	NM_001114339	Pank1	pantothenate kinase 1	1.53
1450945_at	NM_011101	Prkca	protein kinase C, alpha	1.53
1416214_at	NM_008565	Mcm4	minichromosome maintenance deficient 4	1.53
1424291_at	NM_172410	Nup93	nucleoporin 93	1.52
1424755_at	NM_146001	Hip1	huntingtin interacting protein 1	1.52
1422544_at	NM_019472	Myo10	myosin X	1.52
1418412_at	NM_009413	Tpd52l1	tumor protein D52-like 1	1.52
1417696_at	NM_009230	Soat1	sterol O-acyltransferase 1	1.52
1422466_at	NM_008750	Nxn	nucleoredoxin	1.52
1448851_a_at	NM_016775	Dnajc5	DnaJ (Hsp40) homolog, subfamily C, member 5	1.52
1428813_a_at	NM_001190385	Caly	calcyon neuron-specific vesicular protein	1.52
1437886_at	NM_183390	Klhl6	kelch-like 6 (Drosophila)	1.52
1419550_a_at	NM_016866	Stk39	serine/threonine kinase 39, STE20/SPS1 homolog	1.52
1437319_at	NM_001081153	Unc13c	unc-13 homolog C (C. elegans)	1.52
1428386_at	NM_001033606	Acsl3	acyl-CoA synthetase long-chain family member 3	1.52
1435908_at	NM_020253	Nrxn2	neurexin II	1.52
1424584_a_at	NM_145824	Ranbp10	RAN binding protein 10	1.52
1438212_at	NM_176972	Usp37	ubiquitin specific peptidase 37	1.52

Probe set	Accession No.	Symbol	Gene Title	Fold Change
1449843_at	NM_009181	St8sia2	ST8 alpha-N-acetyl-neuraminide alpha-2,8-	1.52
1418127_a_at	NM_012019	Aifm1	apoptosis-inducing factor, mitochondrion-	1.51
1456146_at	BI735554	2210411A11Rik	RIKEN cDNA 2210411A11 gene	1.51
1450904_at	NM_025335	Tmem167	transmembrane protein 167	1.51
1451083_s_at	NM_146217	Aars	alanyl-tRNA synthetase	1.51
1458984_at	BI794701	---		1.51
1448024_at	NM_001039181	Npr3	natriuretic peptide receptor 3	1.51
1420295_x_at	NM_016691	Clcn5	chloride channel 5	1.51
1428307_at	NM_028031	Zdhhc13	zinc finger, DHHC domain containing 13	1.51
1416241_at	NM_024206	Sec13	SEC13 homolog (S. cerevisiae)	1.51
1429330_at	NM_010251	Gabra4	gamma-aminobutyric acid (GABA) A receptor,	1.51
1439619_at	NM_011544	Tcf12	transcription factor 12	1.51
1417929_at	NM_016972	Slc7a8	solute carrier family 7 (cationic amino acid	1.51
1437695_at	NM_144944	Prokr2	prokineticin receptor 2	1.51
1456481_at	NM_177775	Esyt3	extended synaptotagmin-like protein 3	1.51
1427229_at	NM_008255	Hmgcr	3-hydroxy-3-methylglutaryl-Coenzyme A	1.51
1441113_at	XR_106375	A430071A18Rik	RIKEN cDNA A430071A18 gene	1.51
1416064_a_at	NM_001163434	Hspa5	heat shock protein 5	1.51
1455951_at	NM_001003913	Mars	methionine-tRNA synthetase	1.50
1421158_at	NM_026599	Cgnl1	cingulin-like 1	1.50
1450923_at	NM_009367	Tgfb2	transforming growth factor, beta 2	1.50
1437397_at	NM_011169	Prlr	prolactin receptor	1.50
1441505_at	BB211484	---		1.50
1419097_a_at	NM_013515	Stom	stomatin	1.50
1418025_at	NM_011498	Bhlhe40	basic helix-loop-helix family, member e40	-1.51
1440343_at	NM_153587	Rps6ka5	ribosomal protein S6 kinase, polypeptide 5	-1.51
1418683_at	NM_011698	Lin7b	lin-7 homolog B (C. elegans)	-1.51
1449579_at	NM_013709	Sh3yl1	Sh3 domain YSC-like 1	-1.51
1438169_a_at	NM_145148	Frmd4b	FERM domain containing 4B	-1.52
1452232_at	NM_001167981	Galnt7	UDP-N-acetyl-alpha-D-galactosamine:	-1.52
1434931_at	NM_001042752	Neo1	neogenin	-1.52
1415975_at	NM_025821	Carhsp1	calcium regulated heat stable protein 1	-1.52
1428176_at	NM_010333	S1pr2	sphingosine-1-phosphate receptor 2	-1.52
1428391_at	NM_144538	Rab3il1	RAB3A interacting protein (rabin3)-like 1	-1.52
1438291_x_at	NM_026069	LOC100502825	60S ribosomal protein L37-like /// ribosomal	-1.52
1417009_at	NM_023143	C1ra	complement component 1, r subcomponent A	-1.52
1417797_a_at	NM_001083916	1810019J16Rik	RIKEN cDNA 1810019J16 gene	-1.52
1419133_at	NM_025276	Evpl	envoplakin	-1.52
1458341_x_at	NM_153422	Pde5a	phosphodiesterase 5A, cGMP-specific	-1.52
1451604_a_at	NM_009612	Acvrl1	activin A receptor, type II-like 1	-1.52
1437190_at	NM_172891	Styk1	serine/threonine/tyrosine kinase 1	-1.52
1423803_s_at	NM_133831	Gltscr2	glioma tumor suppressor candidate region gene	-1.52
1425182_x_at	NM_010114	Klk1b22 ///	kallikrein 1-related peptidase b22 /// kallikrein 1-	-1.52
1418345_at	NM_001034097	BC096441 ///	cDNA sequence BC096441 /// tumor necrosis	-1.52
1424915_s_at	NM_173735	2310044G17Rik	RIKEN cDNA 2310044G17 gene	-1.52

Probe set	Accession No.	Symbol	Gene Title	Fold Change
1423831_at	NM_001170555	Prkag2	protein kinase, AMP-activated, gamma 2 non-	-1.52
1447858_x_at	NM_001008700	Il4ra	interleukin 4 receptor, alpha	-1.52
1417646_a_at	NM_024225	Snx5	sorting nexin 5	-1.52
1417169_at	NM_016808	Usp2	ubiquitin specific peptidase 2	-1.52
1429086_at	NM_026496	Gm16136 ///	predicted gene 16136 /// grainyhead-like 2	-1.52
1417639_at	NM_019687	Slc22a4	solute carrier family 22 (organic cation	-1.52
1416457_at	NM_001190449	Ddah2	dimethylarginine dimethylaminohydrolase 2	-1.52
1423132_a_at	NM_021552	Nsa2	NSA2 ribosome biogenesis homolog (S.	-1.52
1435258_at	NM_001040130	Tmem141	transmembrane protein 141	-1.52
1448694_at	NM_010591	Jun	Jun oncogene	-1.52
1438560_x_at	NM_009837	Cct4	chaperonin containing Tcp1, subunit 4 (delta)	-1.52
1417667_a_at	NM_008961	Pter	phosphotriesterase related	-1.52
1428643_at	NM_145128	Mgat5	mannoside acetylglucosaminyltransferase 5	-1.52
1439962_at	XM_896611	2310010J17Rik	RIKEN cDNA 2310010J17 gene	-1.53
1418259_a_at	NM_009849	Entpd2	ectonucleoside triphosphate diphosphohydrolase	-1.53
1420357_s_at	NM_001081643	LOC630164 ///	x-linked lymphocyte-regulated protein 3B-like ///	-1.53
1418984_at	NM_001005784	Inadl	InaD-like (Drosophila)	-1.53
1435866_s_at	NM_178218	Hist3h2a	histone cluster 3, H2a	-1.53
1454850_at	NM_178650	Tbc1d10c	TBC1 domain family, member 10c	-1.53
1435578_s_at	NM_010014	Dab1	disabled homolog 1 (Drosophila)	-1.53
1452426_x_at	BC004065	---		-1.53
1448735_at	NM_001042611	Cp	ceruloplasmin	-1.53
1438559_x_at	NM_152808	Slc44a2	solute carrier family 44, member 2	-1.53
1440325_at	AV332226	---		-1.53
1451249_at	NM_001164559	Trmt1	TRM1 tRNA methyltransferase 1 homolog (S.	-1.53
1416654_at	NM_025286	Slc31a2	solute carrier family 31, member 2	-1.53
1417409_at	NM_010591	Jun	Jun oncogene	-1.53
1437145_s_at	NM_026415	2310002J15Rik	RIKEN cDNA 2310002J15 gene	-1.53
1418712_at	NM_021454	Cdc42ep5	CDC42 effector protein (Rho GTPase binding) 5	-1.53
1438306_at	NM_027934	Rnf180	ring finger protein 180	-1.53
1423153_x_at	NM_009888	Cfh	complement component factor h	-1.53
1426378_at	NM_145625	Eif4b	eukaryotic translation initiation factor 4B	-1.53
1435293_at	NM_001007220	Adam22	a disintegrin and metallopeptidase domain 22	-1.53
1439011_at	XR_105469	LOC100504475	hypothetical LOC100504475	-1.53
1420388_at	NM_008939	Prss12	protease, serine, 12 neurotrypsin (motopsin)	-1.53
1419040_at	NM_001163472	Cyp2d22	cytochrome P450, family 2, subfamily d,	-1.53
1452657_at	NM_026887	Ap1s2	adaptor-related protein complex 1, sigma 2	-1.53
1456087_at	NM_001122952	Nfia	nuclear factor I/A	-1.53
1435549_at	NM_175130	Trpm4	transient receptor potential cation channel,	-1.53
1419406_a_at	NM_001159289	Bcl11a	B-cell CLL/lymphoma 11A (zinc finger protein)	-1.53
1450612_a_at	NM_008819	Pemt	phosphatidylethanolamine N-methyltransferase	-1.53
1441206_at	NM_080451	Synpo2	synaptopodin 2	-1.53
1456258_at	NM_010132	Emx2	empty spiracles homolog 2 (Drosophila)	-1.53
1441105_at	NR_033508	Gm11110	predicted gene 11110	-1.54
1423100_at	NM_010234	Fos	FBJ osteosarcoma oncogene	-1.54

Probe set	Accession No.	Symbol	Gene Title	Fold Change
1416675_s_at	NM_019676	Plcd1	phospholipase C, delta 1	-1.54
1433843_at	NM_021429	Hs1bp3	HCLS1 binding protein 3	-1.54
1453639_s_at	NM_026714	Ccdc163	coiled-coil domain containing 163	-1.54
1417933_at	NM_008344	Igfbp6	insulin-like growth factor binding protein 6	-1.54
1435389_at	NM_178256	Reps2	RALBP1 associated Eps domain containing	-1.54
1433769_at	NM_001146059	Als2cl	ALS2 C-terminal like	-1.54
1450850_at	NM_009510	Ezr	ezrin	-1.54
1424023_at	NM_145582	Ctu1	cytosolic thiouridylase subunit 1 homolog (S.	-1.54
1433782_at	NM_001193659	Cldn12	claudin 12	-1.54
1417455_at	NM_009368	Tgfb3	transforming growth factor, beta 3	-1.54
1426380_at	NM_145625	Eif4b	eukaryotic translation initiation factor 4B	-1.54
1429399_at	NM_026301	Rnf125	ring finger protein 125	-1.54
1426569_a_at	NM_001159544	Frk	fyn-related kinase	-1.54
1437217_at	NM_001012450	Ankrd6	ankyrin repeat domain 6	-1.54
1448551_a_at	NM_030706	Trim2	tripartite motif-containing 2	-1.54
1460204_at	NM_001113460	Tec	tec protein tyrosine kinase	-1.54
1436363_a_at	NM_001081981	Nfix	nuclear factor I/X	-1.54
1416538_at	NM_011734	Siae	sialic acid acetylesterase	-1.54
1425553_s_at	NM_145070	Hip1r	huntingtin interacting protein 1 related	-1.54
1423738_at	NM_026936	Oxa1l	oxidase assembly 1-like	-1.54
1420930_s_at	NM_018761	Ctnnal1	catenin (cadherin associated protein), alpha-like 1	-1.54
1459348_at	BB454472	---		-1.54
1417661_at	NM_025654	Rdm1	RAD52 motif 1	-1.54
1435261_at	NM_198967	Tmtc1	transmembrane and tetratricopeptide repeat	-1.54
1432263_a_at	NM_001159529	Cox7a2l	cytochrome c oxidase subunit VIIa polypeptide 2-	-1.54
1459512_at	BM123004	---		-1.54
1438718_at	NM_013518	Fgf9	fibroblast growth factor 9	-1.54
1438635_x_at	NM_178699	B930041F14Rik	RIKEN cDNA B930041F14 gene	-1.54
1434828_at	NM_001163567	Fam102b	family with sequence similarity 102, member B	-1.54
1424052_at	NM_025920	Thap4	THAP domain containing 4	-1.54
1438712_at	NM_001093754	Dennd2d	DENN/MADD domain containing 2D	-1.54
1440173_x_at	NM_011347	Selp	selectin, platelet	-1.54
1455322_at	NM_001047604	Ttc21b	tetratricopeptide repeat domain 21B	-1.54
1435893_at	NM_001161420	Vldlr	very low density lipoprotein receptor	-1.55
1415997_at	NM_001009935	Txnip	thioredoxin interacting protein	-1.55
1432264_x_at	NM_001159529	Cox7a2l	cytochrome c oxidase subunit VIIa polypeptide 2-	-1.55
1451431_a_at	NM_001048227	Dbndd2	dysbindin (dystrobrevin binding protein 1)	-1.55
1422658_at	NM_054066	Plcz1	phospholipase C, zeta 1	-1.55
1434963_at	NM_178652	Supt3h	suppressor of Ty 3 homolog (S. cerevisiae)	-1.55
1423222_at	NM_026056	Cap2	CAP, adenylate cyclase-associated protein, 2	-1.55
1427501_at	NM_175176	4922501L14Rik	RIKEN cDNA 4922501L14 gene	-1.55
1434291_a_at	NM_011353	Serf1	small EDRK-rich factor 1	-1.55
1432474_a_at	NM_027221	Krtcap3	keratinocyte associated protein 3	-1.55
1438494_at	NM_008285	Hrh1	histamine receptor H1	-1.55
1419652_s_at	NM_025998	Nkain1	Na+/K+ transporting ATPase interacting 1	-1.55

Probe set	Accession No.	Symbol	Gene Title	Fold Change
1419231_s_at	NM_010661	Krt12	keratin 12	-1.55
1436246_at	AI849504	---		-1.55
1425162_at	NM_001043354	Rorb	RAR-related orphan receptor beta	-1.55
1440359_at	NM_173426	Fam110b	family with sequence similarity 110, member B	-1.55
1416418_at	NM_020590	Gabarapl1	gamma-aminobutyric acid (GABA) A receptor-	-1.56
1420640_at	NM_021310	Jmy	junction-mediating and regulatory protein	-1.56
1441930_x_at	NM_012037	Vat1	Vesicle amine transport protein 1 homolog (T	-1.56
1449799_s_at	NM_026163	Pkp2	plakophilin 2	-1.56
1453321_at	NM_001081416	Fndc1	fibronectin type III domain containing 1	-1.56
1418449_at	NM_133664	Lad1	ladinin	-1.56
1452507_at	NM_010057	Dlx6	distal-less homeobox 6	-1.56
1453168_at	NM_001033148	1700029J07Rik	RIKEN cDNA 1700029J07 gene	-1.56
1430375_a_at	NM_001048179	Ccl27a ///	chemokine (C-C motif) ligand 27A /// chemokine	-1.56
1423891_at	NM_133994	Gstt3	glutathione S-transferase, theta 3	-1.56
1451542_at	NM_024186	Ssbp2	single-stranded DNA binding protein 2	-1.56
1451121_a_at	NM_133831	Gltscr2	glioma tumor suppressor candidate region gene	-1.56
1450404_at	NM_011397	Slc23a1	solute carrier family 23 (nucleobase transporters),	-1.56
1435336_at	NM_001004177	Celsr2	cadherin, EGF LAG seven-pass G-type receptor 2	-1.56
1424468_s_at	NM_153537	Phldb1	pleckstrin homology-like domain, family B,	-1.56
1419291_x_at	NR_002840	Gas5	growth arrest specific 5	-1.56
1436677_at	NR_027819	1810032O08Rik	RIKEN cDNA 1810032O08 gene	-1.56
1454748_at	NM_172607	Naprt1	nicotinate phosphoribosyltransferase domain	-1.56
1419149_at	NM_008871	Serpine1	serine (or cysteine) peptidase inhibitor, clade E,	-1.56
1436689_a_at	NM_019993	Aldh9a1	aldehyde dehydrogenase 9, subfamily A1	-1.56
1415874_at	NM_011896	Spry1	sprouty homolog 1 (Drosophila)	-1.56
1418162_at	NM_021297	Tlr4	toll-like receptor 4	-1.56
1440781_at	BB333095	B830007D08Rik	RIKEN cDNA B830007D08 gene	-1.56
1454666_at	NM_008453	Klf3	Kruppel-like factor 3 (basic)	-1.56
1448690_at	NM_008430	Kcnk1	potassium channel, subfamily K, member 1	-1.56
1423717_at	NM_021299	Ak3	adenylate kinase 3	-1.56
1448400_a_at	NM_001130187	Smarcd2	SWI/SNF related, matrix associated, actin	-1.56
1435763_at	NM_172443	Tbc1d16	TBC1 domain family, member 16	-1.57
1452590_a_at	NM_207229	Gm10393 ///	predicted gene 10393 /// placenta specific 9	-1.57
1444646_at	NM_172870	Bnc2	basonuclin 2	-1.57
1460214_at	NM_008791	Pcp4	Purkinje cell protein 4	-1.57
1428508_at	NM_194334	Tbc1d2b	TBC1 domain family, member 2B	-1.57
1435339_at	NM_146188	Kctd15	potassium channel tetramerisation domain	-1.57
1419638_at	NM_010111	Efnb2	ephrin B2	-1.57
1418791_at	NM_019535	Sh3gl2	SH3-domain GRB2-like 2	-1.57
1423861_at	NM_175175	Plekhf2	pleckstrin homology domain containing, family F	-1.57
1426743_at	NM_145220	Appl2	adaptor protein, phosphotyrosine interaction, PH	-1.57
1428484_at	NM_001163645	Osbpl3	oxysterol binding protein-like 3	-1.57
1448665_at	NM_007868	Dmd	dystrophin, muscular dystrophy	-1.57
1429360_at	NM_008453	Klf3	Kruppel-like factor 3 (basic)	-1.57
1420362_a_at	NM_007546	Bik	BCL2-interacting killer	-1.57

Probe set	Accession No.	Symbol	Gene Title	Fold Change
1427230_at	NM_178699	B930041F14Rik	RIKEN cDNA B930041F14 gene	-1.57
1425179_at	NM_009171	Shmt1	serine hydroxymethyltransferase 1 (soluble)	-1.57
1425792_a_at	NM_011281	Rorc	RAR-related orphan receptor gamma	-1.57
1424942_a_at	NM_001177352	Myc	myelocytomatosis oncogene	-1.57
1447903_x_at	NM_026887	Ap1s2	adaptor-related protein complex 1, sigma 2	-1.57
1424467_at	NM_153537	Phldb1	pleckstrin homology-like domain, family B,	-1.57
1418014_a_at	NM_022305	B4galt1	UDP-Gal:betaGlcNAc beta 1,4-	-1.57
1429089_s_at	NM_172884	2900026A02Rik	RIKEN cDNA 2900026A02 gene	-1.58
1450178_at	NM_001079873	Brdt	bromodomain, testis-specific	-1.58
1427095_at	NM_133974	Cdcp1	CUB domain containing protein 1	-1.58
1450910_at	NM_026056	Cap2	CAP, adenylate cyclase-associated protein, 2	-1.58
1437341_x_at	NM_001146318	Cnp	2',3'-cyclic nucleotide 3' phosphodiesterase	-1.58
1456925_at	NM_001159561	P2rx6	purinergic receptor P2X, ligand-gated ion	-1.58
1419239_at	NM_011760	Zfp54	zinc finger protein 54	-1.58
1435605_at	NM_001004365	Actr3b	ARP3 actin-related protein 3 homolog B (yeast)	-1.58
1443736_at	BG868839	Gm13648	predicted gene 13648	-1.58
1424652_at	NM_145570	Fam176a	family with sequence similarity 176, member A	-1.58
1423996_a_at	NM_001008700	Il4ra	interleukin 4 receptor, alpha	-1.58
1421456_at	NM_008772	P2ry1	purinergic receptor P2Y, G-protein coupled 1	-1.58
1455747_at	NM_011820	Ggt5	gamma-glutamyltransferase 5	-1.58
1433674_a_at	NR_002896	Snhg1	small nucleolar RNA host gene (non-protein	-1.58
1419457_at	NM_012026	Rgnef	Rho-guanine nucleotide exchange factor	-1.58
1455722_at	BB425316	---		-1.58
1438916_x_at	NR_015505	6720401G13Rik	RIKEN cDNA 6720401G13 gene	-1.58
1418294_at	NM_019427	Epb4.1l4b	erythrocyte protein band 4.1-like 4b	-1.58
1436425_at	NM_172872	Kank4	KN motif and ankyrin repeat domains 4	-1.58
1429625_at	BM202541	2900054C01Rik	RIKEN cDNA 2900054C01 gene	-1.58
1417101_at	NM_001002012	Hspa2	heat shock protein 2	-1.58
1440431_at	BB053468	---		-1.58
1424586_at	NM_153078	Ehbp1	EH domain binding protein 1	-1.58
1434432_at	NM_001007465	Rffl	ring finger and FYVE like domain containing	-1.58
1436322_a_at	XR_105685	2810001A02Rik	RIKEN cDNA 2810001A02 gene	-1.58
1439268_x_at	NM_008388	Eif3e	eukaryotic translation initiation factor 3, subunit E	-1.58
1424921_at	NM_198095	Bst2	bone marrow stromal cell antigen 2	-1.58
1435029_at	NM_001164791	B230120H23Rik	RIKEN cDNA B230120H23 gene	-1.58
1417516_at	NM_007837	Ddit3	DNA-damage inducible transcript 3	-1.59
1439439_x_at	NM_023240	Eef1d	eukaryotic translation elongation factor 1 delta	-1.59
1445326_at	NM_177879	Sdk1	sidekick homolog 1 (chicken)	-1.59
1436330_x_at	XR_033816	Gm7072	predicted gene 7072	-1.59
1439078_at	NM_172781	Klhl4	kelch-like 4 (Drosophila)	-1.59
1425619_s_at	NM_007883	Dsg2	desmoglein 2	-1.59
1418216_at	NM_011820	Ggt5	gamma-glutamyltransferase 5	-1.59
1423825_at	NM_026582	Wls	wntless homolog (Drosophila)	-1.59
1437064_at	NM_013476	Ar	androgen receptor	-1.59
1453771_at	NM_028450	Gulp1	GULP, engulfment adaptor PTB domain	-1.59

Probe set	Accession No.	Symbol	Gene Title	Fold Change
1452009_at	NM_027238	Ttc39b	tetratricopeptide repeat domain 39B	-1.59
1424616_s_at	NM_145583	Pgap2	post-GPI attachment to proteins 2	-1.59
1435171_at	XM_001474539	2810416G20Rik	RIKEN cDNA 2810416G20 gene	-1.59
1426566_s_at	NM_001034029	Il17re	interleukin 17 receptor E	-1.59
1452330_a_at	NM_024263	Mxra8	matrix-remodelling associated 8	-1.59
1458406_at	BG144063	---		-1.59
1452905_at	NR_003633	Meg3	maternally expressed 3	-1.59
1426915_at	NM_029653	Dapk1	death associated protein kinase 1	-1.59
1449815_a_at	NM_024186	Ssbp2	single-stranded DNA binding protein 2	-1.59
1454646_at	NM_146008	Tcp11l2	t-complex 11 (mouse) like 2	-1.59
1456365_at	NR_033523	Gm11517	ubiquitin A-52 residue ribosomal protein fusion	-1.59
1428671_at	NM_028179	2200002D01Rik	RIKEN cDNA 2200002D01 gene	-1.59
1460634_at	NM_001145834	Ralgds	ral guanine nucleotide dissociation stimulator	-1.59
1420905_at	NM_008359	Il17ra	interleukin 17 receptor A	-1.60
1418311_at	NM_001038699	Fn3k	fructosamine 3 kinase	-1.60
1417814_at	NM_001122954	Pla2g5	phospholipase A2, group V	-1.60
1448316_at	NM_024217	Cmtm3	CKLF-like MARVEL transmembrane domain	-1.60
1434917_at	NM_172496	Cobl	cordon-bleu	-1.60
1434410_at	NM_174848	Crybg3	beta-gamma crystallin domain containing 3	-1.60
1416686_at	NM_001142916	Plod2	procollagen lysine, 2-oxoglutarate 5-dioxygenase	-1.60
1427313_at	NM_008967	Ptgir	prostaglandin I receptor (IP)	-1.60
1435059_at	BM937495	---		-1.60
1441894_s_at	NM_019518	Grasp	GRP1 (general receptor for phosphoinositides 1)-	-1.60
1423895_a_at	NM_001110228	Celf2	CUGBP, Elav-like family member 2	-1.60
1440888_at	NM_001081147	Oxtr	oxytocin receptor	-1.60
1449410_a_at	NR_002840	Gas5	growth arrest specific 5	-1.60
1449161_at	NM_007902	Edn2	endothelin 2	-1.60
1419442_at	NM_016762	Matn2	matrilin 2	-1.60
1419262_at	NM_025862	Acad8	acyl-Coenzyme A dehydrogenase family, member	-1.60
1436767_at	NM_001170848	Luc7l2	LUC7-like 2 (S. cerevisiae)	-1.60
1450069_a_at	NM_001110228	Celf2	CUGBP, Elav-like family member 2	-1.60
1436520_at	XM_003085583	Ahnak2	AHNAK nucleoprotein 2	-1.60
1424357_at	NM_144936	Tmem45b	transmembrane protein 45b	-1.61
1452195_s_at	NM_030207	Sfi1	Sfi1 homolog, spindle assembly associated (yeast)	-1.61
1436403_at	NM_199200	Fam171a2	family with sequence similarity 171, member A2	-1.61
1417577_at	NM_019510	Trpc3	transient receptor potential cation channel,	-1.61
1441963_at	NM_197945	Prosapip1	ProSAPiP1 protein	-1.61
1431892_a_at	NM_152813	Plcd3	phospholipase C, delta 3	-1.61
1450063_at	NM_019445	Fmn2	formin 2	-1.61
1438915_at	NR_015505	6720401G13Rik	RIKEN cDNA 6720401G13 gene	-1.61
1417168_a_at	NM_016808	Usp2	ubiquitin specific peptidase 2	-1.61
1429269_at	NM_207203	BC068157	cDNA sequence BC068157	-1.61
1424919_at	NM_001003817	Erbb2	v-erb-b2 erythroblastic leukemia viral oncogene	-1.61
1434025_at	BG069607	---		-1.61
1459981_s_at	NM_172684	Rsbn1	rosbin, round spermatid basic protein 1	-1.61

Probe set	Accession No.	Symbol	Gene Title	Fold Change
1433675_at	NR_002896	Snhg1	small nucleolar RNA host gene (non-protein	-1.61
1460092_at	BB082567	---		-1.61
1434045_at	NM_009875	Cdkn1b	cyclin-dependent kinase inhibitor 1B	-1.62
1436933_at	BM211430	---		-1.62
1448265_x_at	NM_007962	Mpzl2	myelin protein zero-like 2	-1.62
1428074_at	NM_001002267	Tmem158	transmembrane protein 158	-1.62
1417738_at	NM_016899	Rab25	RAB25, member RAS oncogene family	-1.62
1417644_at	NM_010656	Sspn	sarcospan	-1.62
1417900_a_at	NM_001161420	Vldlr	very low density lipoprotein receptor	-1.62
1427343_at	NM_029182	Rasd2	RASD family, member 2	-1.62
1427944_at	NM_181541	Caprin2	caprin family member 2	-1.62
1443997_at	NM_183209	Gprin2	G protein regulated inducer of neurite outgrowth	-1.62
1422573_at	NM_009667	Ampd3	adenosine monophosphate deaminase 3	-1.62
1437466_at	NM_009655	Alcam	activated leukocyte cell adhesion molecule	-1.62
1424041_s_at	NM_001097617	C1s	complement component 1, s subcomponent	-1.62
1436413_at	NM_001159544	Frk	fyn-related kinase	-1.62
1422747_at	NM_016681	Chek2	CHK2 checkpoint homolog (S. pombe)	-1.62
1451326_at	NM_029631	Abhd14b	abhydrolase domain containing 14b	-1.62
1452398_at	NM_019588	Plce1	phospholipase C, epsilon 1	-1.62
1448398_s_at	NM_009079	Rpl22	ribosomal protein L22	-1.63
1449740_s_at	NM_007883	Dsg2	desmoglein 2	-1.63
1435044_at	NM_001110513	Ebf4	early B-cell factor 4	-1.63
1438321_x_at	NM_133858	Fam63a	family with sequence similarity 63, member A	-1.63
1419350_at	NM_001167991	Hook2	hook homolog 2 (Drosophila)	-1.63
1438183_x_at	NM_146126	Sord	sorbitol dehydrogenase	-1.63
1425484_at	NM_145711	Tox	thymocyte selection-associated high mobility	-1.63
1418990_at	NM_025658	Ms4a4d	membrane-spanning 4-domains, subfamily A,	-1.63
1427082_at	NM_028725	Sdr42e1	short chain dehydrogenase/reductase family 42E,	-1.63
1437449_at	NM_001013381	Rsad1	radical S-adenosyl methionine domain containing	-1.63
1430780_a_at	NM_013872	Pmm1	phosphomannomutase 1	-1.63
1439566_at	NM_183183	Gprin3	GPRIN family member 3	-1.64
1422438_at	NM_010145	Ephx1	epoxide hydrolase 1, microsomal	-1.64
1437626_at	NM_001001806	Zfp36l2	zinc finger protein 36, C3H type-like 2	-1.64
1433639_at	NM_172543	Fam117a	family with sequence similarity 117, memberA	-1.64
1428622_at	NM_001037937	Depdc6 ///	DEP domain containing 6 /// hypothetical	-1.64
1428431_at	NM_001101433	Zcchc24	zinc finger, CCHC domain containing 24	-1.64
1455896_a_at	NM_008430	Kcnk1	potassium channel, subfamily K, member 1	-1.64
1456559_at	NR_002863	Emx2os	empty spiracles homolog 2 (Drosophila) opposite	-1.64
1423297_at	NM_001164099	Add3	adducin 3 (gamma)	-1.64
1419261_at	NM_025862	Acad8	acyl-Coenzyme A dehydrogenase family, member	-1.64
1435138_at	NM_173446	Fam155a	family with sequence similarity 155, member A	-1.64
1437271_at	NM_019952	Clcf1	cardiotrophin-like cytokine factor 1	-1.64
1450698_at	NM_010090	Dusp2	dual specificity phosphatase 2	-1.64
1426708_at	NM_133738	Antxr2	anthrax toxin receptor 2	-1.64
1433557_at	NM_144811	Cbx7	chromobox homolog 7	-1.64

Probe set	Accession No.	Symbol	Gene Title	Fold Change
1449013_at	NM_007908	Eef2k	eukaryotic elongation factor-2 kinase	-1.64
1417702_a_at	NM_080462	Hnmt	histamine N-methyltransferase	-1.65
1428344_at	NM_028922	Ppapdc2	phosphatidic acid phosphatase type 2 domain	-1.65
1418829_a_at	NM_013509	Eno2	enolase 2, gamma neuronal	-1.65
1455096_at	NM_201518	Flrt2	fibronectin leucine rich transmembrane protein 2	-1.65
1417812_a_at	NM_008484	Lamb3	laminin, beta 3	-1.65
1448901_at	NM_019696	Cpxm1	carboxypeptidase X 1 (M14 family)	-1.65
1418924_at	NM_025886	Rassf7	Ras association (RalGDS/AF-6) domain family (N-	-1.65
1440147_at	NM_144945	Lgi2	leucine-rich repeat LGI family, member 2	-1.65
1418486_at	NM_011704	Vnn1	vanin 1	-1.65
1429390_at	NM_153420	Acpl2	acid phosphatase-like 2	-1.65
1452151_at	NM_172611	Gramd4	GRAM domain containing 4	-1.65
1417492_at	NM_007798	Ctsb	cathepsin B	-1.65
1421350_a_at	NM_028736	Grip1	glutamate receptor interacting protein 1	-1.65
1429727_at	NM_025807	Slc16a9	solute carrier family 16 (monocarboxylic acid	-1.65
1423602_at	NM_009421	Traf1	TNF receptor-associated factor 1	-1.65
1455250_at	NM_133816	Sh3bp4	SH3-domain binding protein 4	-1.65
1438061_at	NM_001162896	4930523C07Rik	RIKEN cDNA 4930523C07 gene	-1.65
1424351_at	NM_026323	Wfdc2	WAP four-disulfide core domain 2	-1.65
1449484_at	NM_011491	Stc2	stanniocalcin 2	-1.65
1438861_at	NM_172870	Bnc2	basonuclin 2	-1.65
1424450_at	NM_001110337	Gprc5c	G protein-coupled receptor, family C, group 5,	-1.66
1419056_at	NM_001025364	Rtn2	reticulon 2 (Z-band associated protein)	-1.66
1423284_at	NM_026345	Mansc1	MANSC domain containing 1	-1.66
1429588_at	NM_026054	2810474O19Rik	RIKEN cDNA 2810474O19 gene	-1.66
1437594_x_at	NM_133779	Pigt	phosphatidylinositol glycan anchor biosynthesis,	-1.66
1451348_at	NM_001037937	Depdc6	DEP domain containing 6	-1.66
1457675_at	NM_001033164	2510002D24Rik	RIKEN cDNA 2510002D24 gene	-1.66
1460386_a_at	NM_009199	Slc1a1	solute carrier family 1 (neuronal/epithelial high	-1.66
1420191_s_at	NM_177473	Tmem191c	transmembrane protein 191C	-1.66
1454824_s_at	NM_001005863	Mtus1	mitochondrial tumor suppressor 1	-1.66
1450971_at	NM_008655	Gadd45b	growth arrest and DNA-damage-inducible 45	-1.66
1421852_at	NM_021542	Kcnk5	potassium channel, subfamily K, member 5	-1.66
1419905_s_at	NM_008278	Hpgd	hydroxyprostaglandin dehydrogenase 15 (NAD)	-1.66
1437744_at	NM_178740	Slitrk4	SLIT and NTRK-like family, member 4	-1.67
1423493_a_at	NM_001081981	Nfix	nuclear factor I/X	-1.67
1417848_at	NM_133218	Zfp704	zinc finger protein 704	-1.67
1417027_at	NM_030706	Trim2	tripartite motif-containing 2	-1.67
1420664_s_at	NM_011171	Procr	protein C receptor, endothelial	-1.67
1438667_at	NM_001032727	Sybu	syntabulin (syntaxin-interacting)	-1.67
1420678_a_at	NM_019583	Il17rb	interleukin 17 receptor B	-1.67
1425396_a_at	NM_001162432	Lck	lymphocyte protein tyrosine kinase	-1.67
1421172_at	NM_007400	Adam12	a disintegrin and metallopeptidase domain 12	-1.67
1435657_at	NM_175367	Ston2	stonin 2	-1.68
1436591_at	NM_001033311	Vsig10	V-set and immunoglobulin domain containing 10	-1.68

Probe set	Accession No.	Symbol	Gene Title	Fold Change
1437661_at	NM_001033220	AU021092	expressed sequence AU021092	-1.68
1425514_at	NM_001024955	Pik3r1	phosphatidylinositol 3-kinase, regulatory subunit,	-1.68
1423718_at	NM_021299	Ak3	adenylate kinase 3	-1.68
1426383_at	NM_001113333	Cry2	cryptochrome 2 (photolyase-like)	-1.68
1424615_at	NM_145583	Pgap2	post-GPI attachment to proteins 2	-1.68
1418406_at	NM_008803	Pde8a	phosphodiesterase 8A	-1.68
1426379_at	NM_145625	Eif4b	eukaryotic translation initiation factor 4B	-1.68
1431109_at	NM_001081224	Prr16	proline rich 16	-1.68
1455908_a_at	NM_029023	Scpep1	serine carboxypeptidase 1	-1.68
1423131_at	NM_021552	Nsa2	NSA2 ribosome biogenesis homolog (S.	-1.68
1457964_at	XR_105000	1810044D09Rik	RIKEN cDNA 1810044D09 gene	-1.68
1425715_at	NM_027163	Il1f8	interleukin 1 family, member 8	-1.68
1437442_at	NM_001122758	Pcdh7	Protocadherin 7	-1.68
1419075_s_at	NM_009117	Saa1	serum amyloid A 1	-1.68
1459749_s_at	NM_183221	Fat4	FAT tumor suppressor homolog 4 (Drosophila)	-1.68
1417883_at	NM_010361	Gstt2	glutathione S-transferase, theta 2	-1.68
1434489_at	NM_172760	Elmo3	engulfment and cell motility 3, ced-12 homolog	-1.68
1426937_at	NM_027519	6330406I15Rik	RIKEN cDNA 6330406I15 gene	-1.69
1449475_at	NM_138652	Atp12a	ATPase, H+/K+ transporting, nongastric, alpha	-1.69
1448724_at	NM_009895	Cish	cytokine inducible SH2-containing protein	-1.69
1429796_at	NM_001164268	Kalrn	kalirin, RhoGEF kinase	-1.69
1436101_at	NM_178607	Rnf24	ring finger protein 24	-1.69
1418581_a_at	NM_001034030	Limk2	LIM motif-containing protein kinase 2	-1.69
1426606_at	NM_145123	Crtac1	cartilage acidic protein 1	-1.69
1427149_at	NM_001160268	Plekha6	pleckstrin homology domain containing, family A	-1.69
1456984_at	NM_133194	Scml2	sex comb on midleg-like 2 (Drosophila)	-1.69
1423679_at	NM_025944	2810432L12Rik	RIKEN cDNA 2810432L12 gene	-1.69
1455426_at	NM_010140	Epha3	Eph receptor A3	-1.69
1448471_a_at	NM_001145799	Ctla2a	cytotoxic T lymphocyte-associated protein 2	-1.69
1419475_a_at	NM_007914	Ehf	ets homologous factor	-1.70
1439428_x_at	NM_146041	Gmds	GDP-mannose 4, 6-dehydratase	-1.70
1421604_a_at	NM_008453	Klf3	Kruppel-like factor 3 (basic)	-1.70
1451574_at	NM_029933	Bcl9	B-cell CLL/lymphoma 9	-1.70
1447583_x_at	NM_029771	Gpr30	G protein-coupled receptor 30	-1.70
1448227_at	NM_010346	Grb7	growth factor receptor bound protein 7	-1.70
1427982_s_at	NM_001005510	Syne2	synaptic nuclear envelope 2	-1.70
1426784_at	NM_172570	Trim47	tripartite motif-containing 47	-1.70
1439571_at	BB820889	E230008J23Rik	RIKEN cDNA E230008J23 gene	-1.70
1452678_a_at	NM_172404	Ccbl1	cysteine conjugate-beta lyase 1	-1.70
1426642_at	NM_010233	Fn1	fibronectin 1	-1.70
1460453_at	NM_145968	Tagap ///	T-cell activation Rho GTPase-activating protein	-1.71
1449403_at	NM_001163748	Pde9a	phosphodiesterase 9A	-1.71
1455144_s_at	NM_001099288	AU040829	expressed sequence AU040829	-1.71
1437173_at	NM_010101	S1pr3	sphingosine-1-phosphate receptor 3	-1.71
1418172_at	NM_013546	Hebp1	heme binding protein 1	-1.71

Probe set	Accession No.	Symbol	Gene Title	Fold Change
1418086_at	NM_026731	Ppp1r14a	protein phosphatase 1, regulatory (inhibitor)	-1.71
1441197_at	NR_015610	9530059O14Rik	RIKEN cDNA 9530059O14 gene	-1.71
1423824_at	NM_026582	Wls	wntless homolog (Drosophila)	-1.71
1460732_a_at	NM_008909	Ppl	periplakin	-1.71
1448530_at	NM_025508	Gmpr	guanosine monophosphate reductase	-1.71
1416996_at	NM_018775	Tbc1d8	TBC1 domain family, member 8	-1.71
1418320_at	NM_133351	Prss8	protease, serine, 8 (prostasin)	-1.71
1453498_x_at	NM_001085409	Steap3	STEAP family member 3	-1.71
1429146_at	NM_001160345	Svip	small VCP/p97-interacting protein	-1.71
1454788_at	NM_177305	Arl4c	ADP-ribosylation factor-like 4C	-1.71
1441065_at	NM_009167	Shc3	Src homology 2 domain-containing transforming	-1.71
1418105_at	NM_019675	Stmn4	stathmin-like 4	-1.71
1440454_at	NM_175437	Pion	pigeon homolog (Drosophila)	-1.71
1443998_at	NM_175445	Rassf2	Ras association (RalGDS/AF-6) domain family	-1.71
1449510_at	NM_001085415	Zfp467	zinc finger protein 467	-1.71
1437784_at	NM_001111026	Runx1t1	runt-related transcription factor 1; translocated	-1.71
1427298_at	NR_002870	Dnm3os	dynamin 3, opposite strand	-1.72
1424067_at	NM_010493	Icam1	intercellular adhesion molecule 1	-1.72
1450931_at	NM_001081039	Dock9	dedicator of cytokinesis 9	-1.72
1438054_x_at	NM_172994	Ppp2r2c	protein phosphatase 2 (formerly 2A), regulatory	-1.72
1419066_at	NM_030244	Ier5l	immediate early response 5-like	-1.72
1451154_a_at	NM_001110228	Celf2	CUGBP, Elav-like family member 2	-1.72
1427482_a_at	NM_007592	Car8	carbonic anhydrase 8	-1.72
1456392_at	NM_001039094	Negr1	neuronal growth regulator 1	-1.72
1441081_a_at	NR_015536	1110038B12Rik	RIKEN cDNA 1110038B12 gene	-1.72
1419836_at	AU040583	---		-1.72
1422818_at	NM_001111324	Nedd9	neural precursor cell expressed, developmentally	-1.72
1417155_at	NM_008709	Mycn	v-myc myelocytomatosis viral related oncogene,	-1.72
1425543_s_at	NM_144920	Plekha5	pleckstrin homology domain containing, family A	-1.72
1460365_a_at	NM_010065	Dnm1	dynamin 1	-1.72
1420904_at	NM_008359	Il17ra	interleukin 17 receptor A	-1.72
1442024_at	NM_001167908	Ppp1r3e	Protein phosphatase 1, regulatory (inhibitor)	-1.72
1433725_at	NM_007395	Acvr1b	activin A receptor, type 1B	-1.73
1422640_at	NM_053134	Pcdhb9	protocadherin beta 9	-1.73
1455337_at	NM_139232	Fgd4	FYVE, RhoGEF and PH domain containing 4	-1.73
1435084_at	BB200607	C730049O14Rik	RIKEN cDNA C730049O14 gene	-1.73
1430886_at	NM_028275	1700112E06Rik	RIKEN cDNA 1700112E06 gene	-1.73
1420349_at	NM_008966	Ptgfr	prostaglandin F receptor	-1.73
1422476_at	NM_023065	Ifi30	interferon gamma inducible protein 30	-1.73
1425104_at	NM_001142731	Kctd1	potassium channel tetramerisation domain	-1.73
1417481_at	NM_001168392	Ramp1	receptor (calcitonin) activity modifying protein 1	-1.73
1418301_at	NM_016851	Irf6	interferon regulatory factor 6	-1.73
1448169_at	NM_010664	Krt18	keratin 18	-1.74
1424833_at	NM_010586	Itpr2	inositol 1,4,5-triphosphate receptor 2	-1.74
1439537_at	BG071024	---		-1.74

Probe set	Accession No.	Symbol	Gene Title	Fold Change
1452308_a_at	NM_178405	Atp1a2	ATPase, Na+/K+ transporting, alpha 2	-1.74
1443260_at	NM_001193271	Meis1	Meis homeobox 1	-1.74
1421066_at	NM_001048177	Jak2	Janus kinase 2	-1.74
1437829_s_at	NM_007908	Eef2k	eukaryotic elongation factor-2 kinase	-1.74
1430211_at	XM_128114	4930415O20Rik	RIKEN cDNA 4930415O20 gene	-1.74
1431805_a_at	NM_027897	Rhpn2	rhophilin, Rho GTPase binding protein 2	-1.74
1440527_at	BI440542	---		-1.74
1424167_a_at	NM_013872	Pmm1	phosphomannomutase 1	-1.75
1427042_at	NM_178920	Mal2	mal, T-cell differentiation protein 2	-1.75
1438972_x_at	NR_030682	2810410L24Rik	RIKEN cDNA 2810410L24 gene	-1.75
1432591_at	NM_021362	Pappa	pregnancy-associated plasma protein A	-1.75
1435695_a_at	NM_026637	Ggct	gamma-glutamyl cyclotransferase	-1.75
1448785_at	NM_001111026	Runx1t1	runt-related transcription factor 1; translocated	-1.75
1438662_at	NM_001099299	Ajap1	adherens junction associated protein 1	-1.75
1437089_at	NM_029011	Pyroxd2	pyridine nucleotide-disulphide oxidoreductase	-1.75
1425016_at	NM_010142	Ephb2	Eph receptor B2	-1.75
1448656_at	NM_001044741	Cacnb3	calcium channel, voltage-dependent, beta 3	-1.75
1449271_a_at	NM_019487	Hebp2	heme binding protein 2	-1.75
1451363_a_at	NM_001093754	Dennd2d	DENN/MADD domain containing 2D	-1.76
1449248_at	NM_009900	Clcn2	chloride channel 2	-1.76
1417483_at	NM_001159394	Nfkbiz	nuclear factor of kappa light polypeptide gene	-1.76
1451848_a_at	NM_009968	Cryz	crystallin, zeta	-1.76
1451323_at	NM_145916	Zfp7	zinc finger protein 7	-1.76
1426865_a_at	NM_001081445	Ncam1	neural cell adhesion molecule 1	-1.76
1451047_at	NM_008409	Itm2a	integral membrane protein 2A	-1.76
1452703_at	NM_001171000	Ahcyl2	S-adenosylhomocysteine hydrolase-like 2	-1.76
1451375_at	NM_007914	Ehf	ets homologous factor	-1.76
1455418_at	BM119433	---		-1.76
1453344_at	NR_027819	1810032O08Rik	RIKEN cDNA 1810032O08 gene	-1.76
1450177_at	NM_033217	Ngfr	nerve growth factor receptor (TNFR superfamily,	-1.76
1422335_at	NM_007418	Adra2c	adrenergic receptor, alpha 2c	-1.77
1418672_at	NM_013778	Akr1c13	aldo-keto reductase family 1, member C13	-1.77
1444632_at	NR_015455	BC064078	cDNA sequence BC064078	-1.77
1443579_s_at	NM_001037937	Depdc6 ///	DEP domain containing 6 /// hypothetical	-1.77
1438241_at	NM_177740	Rgma	RGM domain family, member A	-1.77
1435933_at	NM_001099298	Scn2a1	sodium channel, voltage-gated, type II, alpha 1	-1.77
1439909_at	NM_145492	Zfp521	zinc finger protein 521	-1.77
1431829_a_at	NM_023622	Rgl3	ral guanine nucleotide dissociation stimulator-like	-1.77
1437132_x_at	NM_001111324	Nedd9	neural precursor cell expressed, developmentally	-1.77
1428915_at	NM_178848	Sirt5	sirtuin 5 (silent mating type information	-1.77
1438325_at	NM_007963	Mecom	MDS1 and EVI1 complex locus	-1.77
1447693_s_at	NM_001042752	Neo1	neogenin	-1.77
1424834_s_at	NM_010586	Itpr2	inositol 1,4,5-triphosphate receptor 2	-1.77
1426626_at	NM_026816	Gtf2f2	general transcription factor IIF, polypeptide 2	-1.77
1451446_at	NM_054041	Antxr1	anthrax toxin receptor 1	-1.77

Probe set	Accession No.	Symbol	Gene Title	Fold Change
1439852_at	BB036443	---		-1.77
1437392_at	NM_001085521	Tmem90b	transmembrane protein 90B	-1.78
1434007_at	NM_001166633	Gyltl1b	glycosyltransferase-like 1B	-1.78
1427386_at	NM_001112744	Arhgef16	Rho guanine nucleotide exchange factor (GEF) 16	-1.78
1421078_at	NM_053085	Tcf23	transcription factor 23	-1.78
1428394_at	NM_172267	Phyhd1	phytanoyl-CoA dioxygenase domain containing 1	-1.78
1454752_at	NM_001081425	Rbm24	RNA binding motif protein 24	-1.78
1449019_at	NM_001042541	Akap1	A kinase (PRKA) anchor protein 1	-1.78
1420838_at	NM_001025074	Ntrk2	neurotrophic tyrosine kinase, receptor, type 2	-1.78
1416958_at	NM_011584	Nr1d2	nuclear receptor subfamily 1, group D, member 2	-1.78
1429022_at	NM_001025372	Adcyap1r1	adenylate cyclase activating polypeptide 1	-1.78
1450992_a_at	NM_001193271	Meis1	Meis homeobox 1	-1.78
1448861_at	NM_011633	Traf5	TNF receptor-associated factor 5	-1.79
1436040_at	NR_029468	Snhg12	small nucleolar RNA host gene 12	-1.79
1421424_a_at	NM_008486	Anpep	alanyl (membrane) aminopeptidase	-1.79
1441506_at	NM_001190451	Dcn	decorin	-1.79
1435115_at	NM_027402	Fndc5	fibronectin type III domain containing 5	-1.79
1455374_at	NM_008426	Kcnj3	potassium inwardly-rectifying channel, subfamily	-1.79
1455607_at	NM_028351	Rspo3	R-spondin 3 homolog (Xenopus laevis)	-1.79
1434158_at	NM_146041	Gmds	GDP-mannose 4, 6-dehydratase	-1.79
1437302_at	NM_007420	Adrb2	adrenergic receptor, beta 2	-1.79
1443964_at	NM_146260	Tmie	transmembrane inner ear	-1.79
1422567_at	NM_022018	Fam129a	family with sequence similarity 129, member A	-1.79
1449553_at	NM_025998	Nkain1	Na+/K+ transporting ATPase interacting 1	-1.79
1448765_at	NM_001122892	Fyn	Fyn proto-oncogene	-1.79
1439476_at	NM_007883	Dsg2	desmoglein 2	-1.79
1450350_a_at	NM_030887	Jdp2	Jun dimerization protein 2	-1.79
1452244_at	NM_027519	6330406I15Rik	RIKEN cDNA 6330406I15 gene	-1.80
1416236_a_at	NM_007962	Mpzl2	myelin protein zero-like 2	-1.80
1455260_at	NM_001163073	Lcorl	ligand dependent nuclear receptor corepressor-	-1.80
1438745_at	NR_027829	Gm10638	predicted gene 10638	-1.80
1429029_at	NM_028943	Sgms2	sphingomyelin synthase 2	-1.80
1417896_at	NM_013769	Tjp3	tight junction protein 3	-1.80
1424175_at	NM_017376	Tef	thyrotroph embryonic factor	-1.80
1427182_s_at	NM_172631	D18Ertd653e	DNA segment, Chr 18, ERATO Doi 653, expressed	-1.80
1438877_at	NM_153417	Trpm6	transient receptor potential cation channel,	-1.80
1429274_at	NM_027990	Lypd6b	LY6/PLAUR domain containing 6B	-1.80
1439406_x_at	NM_001039189	Fars2	phenylalanine-tRNA synthetase 2 (mitochondrial)	-1.80
1418788_at	NM_013690	Tek	endothelial-specific receptor tyrosine kinase	-1.80
1435951_at	NM_028736	Grip1	glutamate receptor interacting protein 1	-1.81
1422018_at	NM_010437	Hivep2	human immunodeficiency virus type I enhancer	-1.81
1444615_x_at	NM_001111026	Runx1t1	runt-related transcription factor 1; translocated	-1.81
1419201_at	NM_001177373	Klk11	kallikrein related-peptidase 11	-1.81
1425108_a_at	NM_001033872	Smagp	small cell adhesion glycoprotein	-1.81
1422721_at	NM_031880	Tnk1	tyrosine kinase, non-receptor, 1	-1.81

Probe set	Accession No.	Symbol	Gene Title	Fold Change
1435603_at	NM_172463	Sned1	sushi, nidogen and EGF-like domains 1	-1.81
1419276_at	NM_008813	Enpp1	ectonucleotide	-1.81
1420512_at	NM_020265	Dkk2	dickkopf homolog 2 (Xenopus laevis)	-1.81
1439098_at	XR_105187	E230013L22Rik	RIKEN cDNA E230013L22 gene	-1.81
1421937_at	NM_011932	Dapp1	dual adaptor for phosphotyrosine and 3-	-1.81
1428809_at	NM_001163473	1810010H24Rik	RIKEN cDNA 1810010H24 gene	-1.81
1460574_at	NM_183221	Fat4	FAT tumor suppressor homolog 4 (Drosophila)	-1.81
1446591_at	NM_001005422	Gm1574	predicted gene 1574	-1.82
1456133_x_at	NM_001145884	Itgb5	integrin beta 5	-1.82
1460178_at	NM_001168591	Lonp2	lon peptidase 2, peroxisomal	-1.82
1426818_at	NM_001042592	Arrdc4	arrestin domain containing 4	-1.82
1453141_at	XR_106377	0610009L18Rik	RIKEN cDNA 0610009L18 gene	-1.82
1424245_at	NM_145603	Ces2c /// Ces2d-	carboxylesterase 2C /// carboxylesterase 2D,	-1.82
1416302_at	NM_007897	Ebf1	early B-cell factor 1	-1.82
1422632_at	NM_009985	Ctsw	cathepsin W	-1.82
1416072_at	NM_001111059	Cd34	CD34 antigen	-1.82
1459546_s_at	NM_008813	Enpp1	ectonucleotide	-1.82
1454617_at	NM_001042591	Arrdc3	arrestin domain containing 3	-1.82
1451502_at	NM_011987	Pla2g10	phospholipase A2, group X	-1.83
1423954_at	NM_009778	C3	complement component 3	-1.83
1448110_at	NM_001163489	Sema4a	sema domain, immunoglobulin domain (Ig),	-1.83
1429206_at	NM_001081347	Rhobtb1	Rho-related BTB domain containing 1	-1.83
1455565_at	NM_029933	Bcl9	B-cell CLL/lymphoma 9	-1.83
1434628_a_at	NM_027897	Rhpn2	rhophilin, Rho GTPase binding protein 2	-1.83
1416401_at	NM_001136055	Cd82	CD82 antigen	-1.84
1418472_at	NM_023113	Aspa	aspartoacylase	-1.84
1456233_at	BB080923	---		-1.84
1421129_a_at	NM_001163336	Atp2a3	ATPase, Ca++ transporting, ubiquitous	-1.84
1419427_at	NM_009971	Csf3	colony stimulating factor 3 (granulocyte)	-1.84
1416101_a_at	NM_015786	Hist1h1c	histone cluster 1, H1c	-1.84
1440879_at	NM_147220	Abca9	ATP-binding cassette, sub-family A (ABC1),	-1.84
1455604_at	NM_001042659	Fzd5	frizzled homolog 5 (Drosophila)	-1.84
1456397_at	NM_009867	Cdh4	cadherin 4	-1.85
1455963_at	NM_177013	Tmem229a	transmembrane protein 229A	-1.85
1416916_at	NM_001163131	Elf3	E74-like factor 3	-1.85
1455799_at	NM_001043354	Rorb	RAR-related orphan receptor beta	-1.85
1455010_at	NM_001081005	1500012F01Rik	RIKEN cDNA 1500012F01 gene	-1.86
1433687_at	NM_001166474	Vmac	vimentin-type intermediate filament associated	-1.86
1418374_at	NM_008557	Fxyd3	FXYD domain-containing ion transport regulator	-1.86
1426864_a_at	NM_001081445	Ncam1	neural cell adhesion molecule 1	-1.87
1449062_at	NM_008439	Khk	ketohexokinase	-1.87
1416029_at	NM_013692	Klf10	Kruppel-like factor 10	-1.87
1434572_at	NM_024124	Hdac9	histone deacetylase 9	-1.87
1449773_s_at	NM_008655	Gadd45b	growth arrest and DNA-damage-inducible 45	-1.87
1417590_at	NM_024264	Cyp27a1	cytochrome P450, family 27, subfamily a,	-1.87

Probe set	Accession No.	Symbol	Gene Title	Fold Change
1439087_a_at	NM_178149	Pik3ip1	phosphoinositide-3-kinase interacting protein 1	-1.87
1416473_a_at	NM_020043	Igdcc4	immunoglobulin superfamily, DCC subclass,	-1.87
1460459_at	NM_028748	Paqr5	progestin and adipoQ receptor family member V	-1.87
1417558_at	NM_001122892	Fyn	Fyn proto-oncogene	-1.87
1454733_at	NM_001171007	Nod1	nucleotide-binding oligomerization domain	-1.88
1418400_at	NM_026235	Larp6	La ribonucleoprotein domain family, member 6	-1.88
1437900_at	NM_001162896	4930523C07Rik	RIKEN cDNA 4930523C07 gene	-1.88
1428066_at	NM_207202	Ccdc120	coiled-coil domain containing 120	-1.88
1416713_at	NM_026481	Tppp3	tubulin polymerization-promoting protein family	-1.88
1438934_x_at	NM_001163489	Sema4a	sema domain, immunoglobulin domain (Ig),	-1.88
1424647_at	NM_146017	Gabrp	gamma-aminobutyric acid (GABA) A receptor, pi	-1.88
1438724_at	NM_001163645	Osbpl3	oxysterol binding protein-like 3	-1.88
1456735_x_at	NM_153420	Acpl2	acid phosphatase-like 2	-1.88
1424127_at	NM_010165	Eya2	eyes absent 2 homolog (Drosophila)	-1.89
1418780_at	NM_018887	Cyp39a1	cytochrome P450, family 39, subfamily a,	-1.89
1423608_at	NM_008409	Itm2a	integral membrane protein 2A	-1.89
1417533_a_at	NM_001145884	Itgb5	integrin beta 5	-1.89
1439827_at	NM_175501	Adamts12	a disintegrin-like and metallopeptidase (reprolysin	-1.89
1452861_at	NM_028096	2010300C02Rik	RIKEN cDNA 2010300C02 gene	-1.89
1425841_at	NM_145947	Slc26a7	solute carrier family 26, member 7	-1.89
1438411_at	NM_175520	Gpr81	G protein-coupled receptor 81	-1.89
1422734_a_at	NM_010848	Myb	myeloblastosis oncogene	-1.90
1417534_at	NM_001145884	Itgb5	integrin beta 5	-1.90
1435196_at	NM_001025074	Ntrk2	neurotrophic tyrosine kinase, receptor, type 2	-1.90
1439764_s_at	NM_183029	Igf2bp2	insulin-like growth factor 2 mRNA binding	-1.91
1428988_at	NM_029600	Abcc3	ATP-binding cassette, sub-family C (CFTR/MRP),	-1.91
1435069_at	NR_015455	BC064078	cDNA sequence BC064078	-1.91
1417050_at	NM_026161	C1qtnf4	C1q and tumor necrosis factor related protein 4	-1.92
1416892_s_at	NM_025626	Fam107b	family with sequence similarity 107, member B	-1.92
1436275_at	NM_030716	Kcnip2	Kv channel-interacting protein 2	-1.92
1449098_a_at	NM_001136090	Poli	polymerase (DNA directed), iota	-1.92
1425248_a_at	NM_019392	Tyro3	TYRO3 protein tyrosine kinase 3	-1.92
1425436_x_at	NM_010648	Klra3 /// Klra9	killer cell lectin-like receptor, subfamily A,	-1.92
1460185_at	NM_016879	Krt85	keratin 85	-1.92
1440973_at	NM_012028	LOC552874 ///	hypothetical LOC552874 /// ST6 (alpha-N-acetyl-	-1.93
1428064_at	NM_001040111	Arap1	ArfGAP with RhoGAP domain, ankyrin repeat and	-1.93
1448293_at	NM_007897	Ebf1	early B-cell factor 1	-1.93
1452893_s_at	NM_027147	Enho	energy homeostasis associated	-1.93
1418511_at	NM_019759	Dpt	dermatopontin	-1.93
1451033_a_at	NM_016984	Trpc4	transient receptor potential cation channel,	-1.93
1437494_at	NM_178907	Mapkapk3	mitogen-activated protein kinase-activated	-1.93
1436665_a_at	NM_001113549	Ltbp4	latent transforming growth factor beta binding	-1.93
1442187_at	NM_009747	Bdkrb2	Bradykinin receptor, beta 2	-1.94
1437434_a_at	NM_026582	Wls	wntless homolog (Drosophila)	-1.94
1428392_at	NM_175445	Rassf2	Ras association (RalGDS/AF-6) domain family	-1.94

Probe set	Accession No.	Symbol	Gene Title	Fold Change
1435687_at	NM_001109753	Sv2b	synaptic vesicle glycoprotein 2 b	-1.95
1429891_at	NM_029341	Capsl	calcyphosine-like	-1.95
1439887_at	NM_001160368	Rnf152	ring finger protein 152	-1.95
1427508_at	NM_001038499	Arsi	arylsulfatase i	-1.95
1428811_at	NM_181819	Wfikkn2	WAP, follistatin/kazal, immunoglobulin, kunitz	-1.95
1416121_at	NM_010728	Lox	lysyl oxidase	-1.95
1450455_s_at	NM_013777	Akr1c12 ///	aldo-keto reductase family 1, member C12 ///	-1.95
1427640_a_at	NM_001111026	Runx1t1	runt-related transcription factor 1; translocated	-1.95
1460259_s_at	NM_009899	Clca1 /// Clca2	chloride channel calcium activated 1 /// chloride	-1.95
1439527_at	NM_008829	Pgr	progesterone receptor	-1.96
1455227_at	NM_178772	Nceh1	arylacetamide deacetylase-like 1	-1.96
1436501_at	NM_001005863	Mtus1	mitochondrial tumor suppressor 1	-1.96
1416623_at	NM_013691	Thbs3	thrombospondin 3	-1.96
1452382_at	NR_002870	Dnm3os	dynamin 3, opposite strand	-1.96
1452213_at	NM_198292	Tex2	testis expressed gene 2	-1.96
1418318_at	NM_023270	Rnf128	ring finger protein 128	-1.96
1449205_at	NM_026924	Ovol2	ovo-like 2 (Drosophila)	-1.96
1420630_at	NM_028982	8430419L09Rik	RIKEN cDNA 8430419L09 gene	-1.96
1434193_at	NM_001099319	Gm12942	predicted gene 12942	-1.97
1427673_a_at	NM_011348	Sema3e	sema domain, immunoglobulin domain (Ig), short	-1.97
1458176_at	NM_011067	Per3	Period homolog 3 (Drosophila)	-1.97
1433626_at	NM_178711	Plscr4	phospholipid scramblase 4	-1.97
1420558_at	NM_011347	Selp	selectin, platelet	-1.97
1424632_a_at	NM_011264	Rev3l	REV3-like, catalytic subunit of DNA polymerase	-1.97
1438114_x_at	NM_010112	Efs	Embryonal Fyn-associated substrate	-1.97
1418186_at	NM_008185	Gstt1	glutathione S-transferase, theta 1	-1.97
1428960_at	NM_027728	Enkur	enkurin, TRPC channel interacting protein	-1.97
1455970_at	NM_153422	Pde5a	phosphodiesterase 5A, cGMP-specific	-1.97
1456060_at	NM_001025577	Maf	avian musculoaponeurotic fibrosarcoma (v-maf)	-1.98
1449563_at	NM_001159647	Cntn1	contactin 1	-1.98
1440156_s_at	NM_001098799	Tox2	TOX high mobility group box family member 2	-1.98
1451601_a_at	NM_153060	Spns2	spinster homolog 2 (Drosophila)	-1.98
1420484_a_at	NM_011707	Vtn	vitronectin	-1.99
1456197_x_at	NM_001099299	Ajap1	Adherens junction associated protein 1	-1.99
1447166_at	AI891479	---		-1.99
1420683_at	NM_001168356	Bnipl	BCL2/adenovirus E1B 19kD interacting protein	-1.99
1439557_s_at	NM_001077398	Ldb2	LIM domain binding 2	-1.99
1440085_at	NM_001161432	Eda2r	ectodysplasin A2 receptor	-1.99
1449591_at	NM_007609	Casp4	caspase 4, apoptosis-related cysteine peptidase	-1.99
1453152_at	NM_174857	Mamdc2	MAM domain containing 2	-1.99
1448509_at	NM_025626	Fam107b	family with sequence similarity 107, member B	-1.99
1419608_a_at	NM_019394	Mia1	melanoma inhibitory activity 1	-2.00
1456786_at	NM_001077398	Ldb2	LIM domain binding 2	-2.00
1416635_at	NM_020561	Smpdl3a	sphingomyelin phosphodiesterase, acid-like 3A	-2.00
1449265_at	NM_009807	Casp1	caspase 1	-2.00

Probe set	Accession No.	Symbol	Gene Title	Fold Change
1452384_at	NM_134005	Enpp3	ectonucleotide	-2.00
1449167_at	NM_013512	Epb4.1l4a	erythrocyte protein band 4.1-like 4a	-2.01
1451335_at	NM_139198	Plac8	placenta-specific 8	-2.01
1418252_at	NM_008812	Padi2	peptidyl arginine deiminase, type II	-2.01
1438658_a_at	NM_010101	S1pr3	sphingosine-1-phosphate receptor 3	-2.01
1419647_a_at	NM_133662	Ier3	immediate early response 3	-2.01
1433965_at	NM_001038999	Atp8a1	ATPase, aminophospholipid transporter (APLT),	-2.01
1417395_at	NM_010637	Klf4	Kruppel-like factor 4 (gut)	-2.01
1436294_at	NM_001190371	Ankrd29	ankyrin repeat domain 29	-2.01
1436260_at	BB131085	---		-2.01
1439990_at	BB460243	---		-2.01
1449315_at	NM_001145937	Odz3	odd Oz/ten-m homolog 3 (Drosophila)	-2.01
1427711_a_at	NM_001039185	Ceacam1	carcinoembryonic antigen-related cell adhesion	-2.02
1427183_at	NM_146015	Efemp1	epidermal growth factor-containing fibulin-like	-2.02
1451453_at	NM_010019	Dapk2	death-associated protein kinase 2	-2.02
1434528_at	NM_175503	Aard	alanine and arginine rich domain containing	-2.02
1427371_at	NM_153145	Abca8a	ATP-binding cassette, sub-family A (ABC1),	-2.02
1418424_at	NM_009398	Tnfaip6	tumor necrosis factor alpha induced protein 6	-2.02
1429938_at	BB125806	A930009E05Rik	RIKEN cDNA A930009E05 gene	-2.02
1453587_at	NM_027819	Ggt6	gamma-glutamyltransferase 6	-2.03
1417071_s_at	NM_133969	Cyp4v3	cytochrome P450, family 4, subfamily v,	-2.03
1453072_at	NM_001134385	Gpr160	G protein-coupled receptor 160	-2.03
1441412_s_at	NM_001165952	Trim45	tripartite motif-containing 45	-2.03
1457321_at	XM_001474094	D130037M23Rik	RIKEN cDNA D130037M23 gene	-2.04
1428696_at	NM_181397	Rftn1	raftlin lipid raft linker 1	-2.04
1426268_at	NM_181323	Cwh43	cell wall biogenesis 43 C-terminal homolog (S.	-2.04
1435895_at	NM_175548	Lsamp	limbic system-associated membrane protein	-2.04
1451527_at	NM_029620	Pcolce2	procollagen C-endopeptidase enhancer 2	-2.04
1439260_a_at	NM_134005	Enpp3	ectonucleotide	-2.04
1458040_at	BM213832	D7Wsu130e	DNA segment, Chr 7, Wayne State University 130,	-2.04
1434423_at	NM_028450	Gulp1	GULP, engulfment adaptor PTB domain	-2.04
1452766_at	NM_182839	Tppp	tubulin polymerization promoting protein	-2.04
1438610_a_at	NM_009968	Cryz	crystallin, zeta	-2.05
1419323_at	NM_011059	Padi1	peptidyl arginine deiminase, type I	-2.05
1454254_s_at	NM_029639	1600029D21Rik	RIKEN cDNA 1600029D21 gene	-2.05
1453135_at	NM_027402	Fndc5	fibronectin type III domain containing 5	-2.05
1444178_at	XR_106016	Gm9895 ///	predicted gene 9895 /// hypothetical	-2.05
1436584_at	NM_011897	Spry2	sprouty homolog 2 (Drosophila)	-2.05
1416474_at	NM_020043	Igdcc4	immunoglobulin superfamily, DCC subclass,	-2.05
1423933_a_at	NM_029639	1600029D21Rik	RIKEN cDNA 1600029D21 gene	-2.06
1436345_at	NM_028872	5730559C18Rik	RIKEN cDNA 5730559C18 gene	-2.06
1421444_at	NM_008829	Pgr	progesterone receptor	-2.06
1417852_x_at	NM_009899	Clca1	chloride channel calcium activated 1	-2.06
1422851_at	NM_010441	Hmga2	high mobility group AT-hook 2	-2.06
1458659_at	NM_207229	Gm10393 ///	predicted gene 10393 /// placenta specific 9	-2.06

Probe set	Accession No.	Symbol	Gene Title	Fold Change
1421002_at	NM_011923	Angptl2	angiopoietin-like 2	-2.07
1459971_at	NM_001081027	Kcnt2	potassium channel, subfamily T, member 2	-2.07
1430111_a_at	NM_001024468	Bcat1	branched chain aminotransferase 1, cytosolic	-2.07
1454078_a_at	NM_001177691	Gal3st1	galactose-3-O-sulfotransferase 1	-2.08
1434815_a_at	NM_178907	Mapkapk3	mitogen-activated protein kinase-activated	-2.08
1429178_at	NM_001145937	Odz3	odd Oz/ten-m homolog 3 (Drosophila)	-2.08
1422637_at	NM_018750	Rassf5	Ras association (RalGDS/AF-6) domain family	-2.08
1417066_at	NM_001163290	Adck3	aarF domain containing kinase 3	-2.08
1454048_a_at	NM_027627	4931408A02Rik	RIKEN cDNA 4931408A02 gene	-2.08
1448263_a_at	NM_023149	Cndp2	CNDP dipeptidase 2 (metallopeptidase M20	-2.09
1417070_at	NM_133969	Cyp4v3	cytochrome P450, family 4, subfamily v,	-2.09
1439616_at	BB167055	---		-2.09
1447808_s_at	NM_001145899	Slc15a2	solute carrier family 15 (H+/peptide transporter),	-2.09
1424966_at	NM_001168256	Tmem40	transmembrane protein 40	-2.09
1435828_at	NM_001025577	Maf	avian musculoaponeurotic fibrosarcoma (v-maf)	-2.10
1456130_at	NM_001164805	Thsd7a	thrombospondin, type I, domain containing 7A	-2.10
1430355_a_at	NM_001085409	Steap3	STEAP family member 3	-2.10
1455257_at	NM_016780	Itgb3	integrin beta 3	-2.10
1421040_a_at	NM_008182	Gsta2	glutathione S-transferase, alpha 2 (Yc2)	-2.10
1427020_at	NM_172604	Scara3	scavenger receptor class A, member 3	-2.11
1419564_at	NM_001085415	Zfp467	zinc finger protein 467	-2.11
1449310_at	NM_008964	Ptger2	prostaglandin E receptor 2 (subtype EP2)	-2.11
1456008_at	NM_028175	Lrrc8e	leucine rich repeat containing 8 family, member E	-2.11
1450871_a_at	NM_001024468	Bcat1	branched chain aminotransferase 1, cytosolic	-2.11
1441572_at	NM_007831	Dcc	deleted in colorectal carcinoma	-2.11
1455697_at	XM_127791	Fam124a	family with sequence similarity 124, member A	-2.12
1416950_at	NM_001177759	Tnfaip8	tumor necrosis factor, alpha-induced protein 8	-2.12
1454880_s_at	NM_138313	Bmf	BCL2 modifying factor	-2.12
1424730_a_at	NM_001145899	Slc15a2	solute carrier family 15 (H+/peptide transporter),	-2.13
1455636_at	NM_175548	Lsamp	limbic system-associated membrane protein	-2.13
1438799_at	NR_015388	Dlx6os1	Dlx6 opposite strand transcript 1	-2.13
1429308_at	NM_001177995	Prdm16	PR domain containing 16	-2.14
1447901_x_at	NM_030207	Sfi1	Sfi1 homolog, spindle assembly associated (yeast)	-2.14
1449036_at	NM_023270	Rnf128	ring finger protein 128	-2.14
1435596_at	NM_175437	Pion	pigeon homolog (Drosophila)	-2.14
1424507_at	NM_145495	Rin1	Ras and Rab interactor 1	-2.14
1438445_at	NM_028175	Lrrc8e	leucine rich repeat containing 8 family, member E	-2.14
1439862_at	NM_001043354	Rorb	RAR-related orphan receptor beta	-2.14
1458591_at	NM_001017427	Rasef	RAS and EF hand domain containing	-2.14
1437361_at	NM_001105561	Gm11545	predicted gene 11545	-2.15
1426231_at	NM_001197028	Vit	vitrin	-2.15
1418492_at	NM_011825	Grem2	gremlin 2 homolog, cysteine knot superfamily	-2.15
1427287_s_at	NM_010586	Itpr2	inositol 1,4,5-triphosphate receptor 2	-2.15
1460177_at	NM_023149	Cndp2	CNDP dipeptidase 2 (metallopeptidase M20	-2.15
1456195_x_at	NM_001145884	Itgb5	integrin beta 5	-2.15

Probe set	Accession No.	Symbol	Gene Title	Fold Change
1449519_at	NM_007836	Gadd45a	growth arrest and DNA-damage-inducible 45	-2.16
1416271_at	NM_022032	Perp	PERP, TP53 apoptosis effector	-2.16
1439680_at	NM_009425	Tnfsf10	tumor necrosis factor (ligand) superfamily,	-2.16
1460242_at	NM_010016	Cd55	CD55 antigen	-2.16
1436566_at	NM_139147	Rab40b	Rab40b, member RAS oncogene family	-2.16
1444697_at	XR_106364	4732490B19Rik	RIKEN cDNA 4732490B19 gene	-2.17
1434606_at	NM_010153	Erbb3	v-erb-b2 erythroblastic leukemia viral oncogene	-2.17
1451260_at	NM_028270	Aldh1b1	aldehyde dehydrogenase 1 family, member B1	-2.17
1455435_s_at	NM_001136240	Chdh	choline dehydrogenase	-2.17
1428812_at	NM_028491	1700040L02Rik	RIKEN cDNA 1700040L02 gene	-2.17
1448321_at	NM_001146217	Smoc1	SPARC related modular calcium binding 1	-2.18
1435667_at	NM_001012623	Rims1	regulating synaptic membrane exocytosis 1	-2.18
1428859_at	NM_153783	Paox	polyamine oxidase (exo-N4-amino)	-2.19
1424220_a_at	NM_016913	Porcn	porcupine homolog (Drosophila)	-2.19
1441917_s_at	NM_001168256	Tmem40	transmembrane protein 40	-2.19
1431461_at	AK016843	4933417E11Rik	RIKEN cDNA 4933417E11 gene	-2.19
1416564_at	NM_011446	Sox7	SRY-box containing gene 7	-2.20
1426938_at	NM_021361	Nova1	neuro-oncological ventral antigen 1	-2.20
1421034_a_at	NM_001008700	Il4ra	interleukin 4 receptor, alpha	-2.21
1426787_at	NM_030207	Sfi1	Sfi1 homolog, spindle assembly associated (yeast)	-2.21
1448964_at	NM_009789	S100g	S100 calcium binding protein G	-2.21
1418925_at	NM_009886	Celsr1	cadherin, EGF LAG seven-pass G-type receptor 1	-2.21
1415857_at	NM_010330	Emb	embigin	-2.21
1434881_s_at	NM_177715	Kctd12	potassium channel tetramerisation domain	-2.22
1436532_at	NM_172928	Dclk3	doublecortin-like kinase 3	-2.22
1451332_at	NM_145492	Zfp521	zinc finger protein 521	-2.22
1422293_a_at	NM_001142731	Kctd1	potassium channel tetramerisation domain	-2.22
1455665_at	NM_001081150	Lonrf1	LON peptidase N-terminal domain and ring	-2.23
1421761_a_at	NM_013800	Barx2	BarH-like homeobox 2	-2.24
1447849_s_at	NM_001025577	Maf	avian musculoaponeurotic fibrosarcoma (v-maf)	-2.24
1450085_at	NM_011923	Angptl2	angiopoietin-like 2	-2.24
1438423_at	NM_024186	Ssbp2	single-stranded DNA binding protein 2	-2.26
1457915_at	NM_177101	4833442J19Rik	RIKEN cDNA 4833442J19 gene	-2.26
1440084_at	AV380966	---		-2.26
1438165_x_at	NM_012037	Vat1	vesicle amine transport protein 1 homolog (T	-2.26
1417602_at	NM_011066	Per2	period homolog 2 (Drosophila)	-2.27
1424208_at	NM_001136079	Ptger4	prostaglandin E receptor 4 (subtype EP4)	-2.27
1459913_at	NM_009425	Tnfsf10	tumor necrosis factor (ligand) superfamily,	-2.27
1427630_x_at	NM_001039185	Ceacam1	carcinoembryonic antigen-related cell adhesion	-2.27
1421065_at	NM_001048177	Jak2	Janus kinase 2	-2.27
1420403_at	NM_001036684	Atp2b2	ATPase, Ca++ transporting, plasma membrane 2	-2.27
1434581_at	NM_026629	2410066E13Rik	RIKEN cDNA 2410066E13 gene	-2.27
1436600_at	NM_172913	Tox3	TOX high mobility group box family member 3	-2.28
1452114_s_at	NM_010518	Igfbp5	insulin-like growth factor binding protein 5	-2.28
1441727_s_at	NM_001085415	Zfp467	zinc finger protein 467	-2.28

Probe set	Accession No.	Symbol	Gene Title	Fold Change
1417394_at	NM_010637	Klf4	Kruppel-like factor 4 (gut)	-2.28
1429844_at	NM_027158	2310043J07Rik	RIKEN cDNA 2310043J07 gene	-2.29
1417835_at	NM_008645	Mug1	murinoglobulin 1	-2.29
1434800_at	NM_001109753	Sv2b	synaptic vesicle glycoprotein 2 b	-2.29
1438305_at	NM_001012623	Rims1	regulating synaptic membrane exocytosis 1	-2.29
1428332_at	NM_178149	Pik3ip1	phosphoinositide-3-kinase interacting protein 1	-2.29
1419456_at	NM_026428	Dcxr	dicarbonyl L-xylulose reductase	-2.30
1423323_at	NM_020047	Tacstd2	tumor-associated calcium signal transducer 2	-2.30
1427053_at	NM_001014399	Abi3bp	ABI gene family, member 3 (NESH) binding	-2.30
1421317_x_at	NM_010848	Myb	myeloblastosis oncogene	-2.30
1415856_at	NM_010330	Emb	embigin	-2.31
1427945_at	NM_170778	Dpyd	dihydropyrimidine dehydrogenase	-2.31
1450781_at	NM_010441	Hmga2	high mobility group AT-hook 2	-2.32
1450194_a_at	NM_010848	Myb	myeloblastosis oncogene	-2.32
1427054_s_at	NM_001014399	Abi3bp	ABI gene family, member 3 (NESH) binding	-2.32
1427512_a_at	NM_010680	Lama3	laminin, alpha 3	-2.32
1427387_a_at	NM_001005608	Itgb4	integrin beta 4	-2.32
1437152_at	NM_175366	Mex3b	mex3 homolog B (C. elegans)	-2.32
1436994_a_at	NM_015786	Hist1h1c	histone cluster 1, H1c	-2.34
1438512_at	NM_001193274	BC048679	cDNA sequence BC048679	-2.34
1452626_a_at	NM_026928	1810014F10Rik	RIKEN cDNA 1810014F10 gene	-2.34
1418091_at	NM_023755	Tcfcp2l1	transcription factor CP2-like 1	-2.35
1455147_at	BB177828	---		-2.36
1425538_x_at	NM_001039185	Ceacam1	carcinoembryonic antigen-related cell adhesion	-2.36
1447936_at	NR_030738	2410006H16Rik	RIKEN cDNA 2410006H16 gene	-2.37
1439699_at	BB525237	---		-2.37
1435033_at	NM_183019	Arhgef4	Rho guanine nucleotide exchange factor (GEF) 4	-2.37
1418930_at	NM_021274	Cxcl10	chemokine (C-X-C motif) ligand 10	-2.37
1453231_at	NM_183294	Cdkl1	cyclin-dependent kinase-like 1 (CDC2-related	-2.37
1440785_at	NM_212452	Rxfp1	relaxin/insulin-like family peptide receptor 1	-2.38
1444262_at	NM_001162998	1110017F19Rik	RIKEN cDNA 1110017F19 gene	-2.38
1427521_a_at	NM_001164263	9930023K05Rik	RIKEN cDNA 9930023K05 gene	-2.39
1438097_at	NM_011227	Rab20	RAB20, member RAS oncogene family	-2.39
1434295_at	NM_011246	Rasgrp1	RAS guanyl releasing protein 1	-2.39
1427344_s_at	NM_029182	Rasd2	RASD family, member 2	-2.39
1456510_x_at	NM_001002900	Higd1c ///	HIG1 domain family, member 1C ///	-2.40
1419468_at	NM_025809	Clec14a	C-type lectin domain family 14, member a	-2.40
1435337_at	NM_172298	Tshz3	teashirt zinc finger family member 3	-2.40
1435296_at	NM_007418	Adra2c	adrenergic receptor, alpha 2c	-2.40
1450780_s_at	NM_010441	Hmga2	high mobility group AT-hook 2	-2.41
1440870_at	NM_001177995	Prdm16	PR domain containing 16	-2.41
1433827_at	NM_001038999	Atp8a1	ATPase, aminophospholipid transporter (APLT),	-2.41
1418417_at	NM_010827	Msc	musculin	-2.42
1422638_s_at	NM_018750	Rassf5	Ras association (RalGDS/AF-6) domain family	-2.42
1419326_at	NM_026188	1700028P14Rik	RIKEN cDNA 1700028P14 gene	-2.42

Probe set	Accession No.	Symbol	Gene Title	Fold Change
1427302_at	NM_134005	Enpp3	ectonucleotide	-2.43
1443906_at	NM_010016	Cd55	CD55 antigen	-2.43
1455772_at	NM_008829	Pgr	progesterone receptor	-2.44
1421912_at	NM_011397	Slc23a1	solute carrier family 23 (nucleobase transporters),	-2.45
1417884_at	NM_001029842	Slc16a6	solute carrier family 16 (monocarboxylic acid	-2.45
1428663_at	NM_028943	Sgms2	sphingomyelin synthase 2	-2.45
1451424_at	NM_146017	Gabrp	gamma-aminobutyric acid (GABA) A receptor, pi	-2.45
1438756_at	NM_001190371	Ankrd29	ankyrin repeat domain 29	-2.45
1444740_at	NM_001164201	Lass3	LAG1 homolog, ceramide synthase 3	-2.46
1452473_at	NM_030024	Prr15	proline rich 15	-2.46
1417756_a_at	NM_001136071	Lsp1	lymphocyte specific 1	-2.46
1455477_s_at	NM_001164557	Pdzk1ip1	PDZK1 interacting protein 1	-2.47
1440339_at	NM_008813	Enpp1	ectonucleotide	-2.47
1434151_at	NM_027334	Mettl7a1	methyltransferase like 7A1	-2.48
1448600_s_at	NM_020505	Vav3	vav 3 oncogene	-2.48
1429313_at	NM_013845	Ror1	receptor tyrosine kinase-like orphan receptor 1	-2.48
1429679_at	NM_028977	Lrrc17	leucine rich repeat containing 17	-2.48
1429918_at	NM_175535	Arhgap20	Rho GTPase activating protein 20	-2.48
1454942_at	NM_022018	Fam129a	family with sequence similarity 129, member A	-2.49
1448640_at	NM_001171010	Slc14a1	solute carrier family 14 (urea transporter),	-2.49
1448566_at	NM_016917	Slc40a1	solute carrier family 40 (iron-regulated	-2.51
1454858_x_at	NM_027334	Mettl7a1	methyltransferase like 7A1	-2.51
1448299_at	NM_009199	Slc1a1	solute carrier family 1 (neuronal/epithelial high	-2.52
1427946_s_at	NM_170778	Dpyd	dihydropyrimidine dehydrogenase	-2.52
1437876_at	NM_001033543	Il20rb	interleukin 20 receptor beta	-2.53
1419467_at	NM_025809	Clec14a	C-type lectin domain family 14, member a	-2.55
1417061_at	NM_016917	Slc40a1	solute carrier family 40 (iron-regulated	-2.55
1417575_at	NM_001177841	Otub2	OTU domain, ubiquitin aldehyde binding 2	-2.55
1437270_a_at	NM_019952	Clcf1	cardiotrophin-like cytokine factor 1	-2.55
1437292_at	NM_175448	Clvs2	clavesin 2	-2.56
1416617_at	NM_080575	Acss1	acyl-CoA synthetase short-chain family member 1	-2.56
1456036_x_at	NM_010362	Gsto1	glutathione S-transferase omega 1	-2.57
1440000_at	NR_026942	E330013P04Rik	RIKEN cDNA E330013P04 gene	-2.58
1417122_at	NM_020505	Vav3	vav 3 oncogene	-2.58
1423619_at	NM_009026	Rasd1	RAS, dexamethasone-induced 1	-2.59
1440487_at	NM_007831	Dcc	deleted in colorectal carcinoma	-2.59
1424007_at	NM_145741	Gdf10	growth differentiation factor 10	-2.59
1429664_at	NM_183294	Cdkl1	cyclin-dependent kinase-like 1 (CDC2-related	-2.60
1451596_a_at	NM_001172472	Sphk1	sphingosine kinase 1	-2.60
1434210_s_at	NM_008377	Lrig1	leucine-rich repeats and immunoglobulin-like	-2.60
1420378_at	NM_009160	Sftpd	surfactant associated protein D	-2.62
1419420_at	NM_012028	St6galnac5	ST6 (alpha-N-acetyl-neuraminyl-2,3-beta-	-2.62
1455699_at	NM_001024468	Bcat1	branched chain aminotransferase 1, cytosolic	-2.62
1452981_at	NM_001159647	Cntn1	contactin 1	-2.63
1424010_at	NM_029568	Mfap4	microfibrillar-associated protein 4	-2.64

Probe set	Accession No.	Symbol	Gene Title	Fold Change
1417689_a_at	NM_001164557	Pdzk1ip1	PDZK1 interacting protein 1	-2.65
1456929_at	BM235852	BC042782	cDNA sequence BC042782	-2.65
1452358_at	NM_001103367	Rai2	retinoic acid induced 2	-2.66
1444524_at	NR_028589	Gm14005	predicted gene 14005	-2.66
1434526_at	NM_001001804	Ephx4	epoxide hydrolase 4	-2.66
1439301_at	BM240022	---		-2.67
1447173_at	XR_002334	Lrrc31	leucine rich repeat containing 31	-2.67
1449082_at	NM_015776	Mfap5	microfibrillar associated protein 5	-2.68
1452065_at	NM_145967	Vstm2a	V-set and transmembrane domain containing 2A	-2.68
1420402_at	NM_001036684	Atp2b2	ATPase, Ca++ transporting, plasma membrane 2	-2.70
1441926_x_at	NM_146260	Tmie	transmembrane inner ear	-2.71
1449893_a_at	NM_008377	Lrig1	leucine-rich repeats and immunoglobulin-like	-2.71
1428114_at	NM_001171010	Slc14a1	solute carrier family 14 (urea transporter),	-2.71
1423468_at	NM_001085409	Steap3	STEAP family member 3	-2.72
1450798_at	NM_031176	Tnxb	tenascin XB	-2.73
1418762_at	NM_010016	Cd55	CD55 antigen	-2.77
1439066_at	NM_009640	Angpt1	angiopoietin 1	-2.77
1434194_at	NM_001039934	Mtap2	microtubule-associated protein 2	-2.77
1458324_x_at	BB208251	---		-2.77
1418174_at	NM_016974	Dbp	D site albumin promoter binding protein	-2.78
1425092_at	NM_009865	Cdh10	cadherin 10	-2.78
1418126_at	NM_013653	Ccl5	chemokine (C-C motif) ligand 5	-2.79
1416531_at	NM_010362	Gsto1	glutathione S-transferase omega 1	-2.79
1455090_at	NM_011923	Angptl2	angiopoietin-like 2	-2.79
1426147_s_at	NM_001160096	Cldn10	claudin 10	-2.81
1454728_s_at	NM_001038999	Atp8a1	ATPase, aminophospholipid transporter (APLT),	-2.81
1438211_s_at	NM_016974	Dbp	D site albumin promoter binding protein	-2.82
1447227_at	AI504711	---		-2.84
1457619_at	NM_198171	Ces2b	carboxyesterase 2B	-2.85
1434150_a_at	NM_001002900	Higd1c ///	HIG1 domain family, member 1C ///	-2.89
1421073_a_at	NM_001136079	Ptger4	prostaglandin E receptor 4 (subtype EP4)	-2.91
1452716_at	NM_027464	5730469M10Rik	RIKEN cDNA 5730469M10 gene	-2.92
1424902_at	NM_001163608	Plxdc1	plexin domain containing 1	-2.95
1427063_at	NM_001033304	5330417C22Rik	RIKEN cDNA 5330417C22 gene	-2.97
1428547_at	NM_011851	Nt5e	5' nucleotidase, ecto	-2.97
1429072_at	NM_026763	Col6a4	collagen, type VI, alpha 4	-2.98
1449319_at	NM_138683	Rspo1	R-spondin homolog (Xenopus laevis)	-3.02
1442226_at	NM_011348	Sema3e	sema domain, immunoglobulin domain (Ig), short	-3.06
1450112_a_at	NM_008087	Gas2	growth arrest specific 2	-3.08
1425415_a_at	NM_009199	Slc1a1	solute carrier family 1 (neuronal/epithelial high	-3.09
1417995_at	NM_008979	Ptpn22	protein tyrosine phosphatase, non-receptor type	-3.12
1423367_at	NM_009527	Wnt7a	wingless-related MMTV integration site 7A	-3.13
1418979_at	NM_134072	Akr1c14	aldo-keto reductase family 1, member C14	-3.14
1455396_at	NM_001001488	Atp8b1	ATPase, class I, type 8B, member 1	-3.15
1424409_at	NM_027998	Cldn23	claudin 23	-3.15

Probe set	Accession No.	Symbol	Gene Title	Fold Change
1419571_at	NM_022317	Slc28a3	solute carrier family 28 (sodium-coupled	-3.16
1426047_a_at	NM_001161838	Ptprr	protein tyrosine phosphatase, receptor type, R	-3.16
1419070_at	NM_001004455	Cys1	cystin 1	-3.18
1432418_a_at	NM_009897	Ckmt1	creatine kinase, mitochondrial 1, ubiquitous	-3.21
1433434_at	NM_178737	AW551984	expressed sequence AW551984	-3.22
1439528_at	NM_001081664	4833423E24Rik	RIKEN cDNA 4833423E24 gene	-3.25
1422178_a_at	NM_001159725	Rab17	RAB17, member RAS oncogene family	-3.25
1418454_at	NM_015776	Mfap5	microfibrillar associated protein 5	-3.25
1419615_at	NM_022413	Trpv6	transient receptor potential cation channel,	-3.27
1455561_at	NM_177450	Cndp1	carnosine dipeptidase 1 (metallopeptidase M20	-3.29
1455528_at	AI315686	---		-3.29
1419717_at	NM_011348	Sema3e	sema domain, immunoglobulin domain (Ig), short	-3.31
1422945_a_at	NM_008449	Kif5c	kinesin family member 5C	-3.36
1444687_at	NM_207233	C1ql2	complement component 1, q subcomponent-like	-3.46
1455516_at	NM_153409	Csrnp3	cysteine-serine-rich nuclear protein 3	-3.48
1421087_at	NM_011067	Per3	period homolog 3 (Drosophila)	-3.49
1428485_at	NM_178396	Car12	carbonic anyhydrase 12	-3.56
1447774_x_at	NM_027464	5730469M10Rik	RIKEN cDNA 5730469M10 gene	-3.61
1429381_x_at	AK007826	Igh-2 /// Igh-VJ558 /// LOC544903	immunoglobulin heavy chain 2 (serum IgA) /// immunoglobulin heavy chain (J558 family) /// similar to immunoglobulin mu-chain	-3.63
1448973_at	NM_016771	Sult1d1	sulfotransferase family 1D, member 1	-3.71
1418724_at	NM_007686	Cfi	complement component factor i	-3.72
1448107_x_at	NM_010639	Klk1	kallikrein 1	-3.73
1433888_at	NM_001036684	Atp2b2	ATPase, Ca++ transporting, plasma membrane 2	-3.74
1434136_at	NM_177013	Tmem229a	transmembrane protein 229A	-3.76
1418858_at	NM_023617	Aox3	aldehyde oxidase 3	-3.79
1436223_at	NM_177290	Itgb8	integrin beta 8	-3.81
1451204_at	NM_001168318	Scara5	scavenger receptor class A, member 5 (putative)	-3.86
1418138_at	NM_016771	Sult1d1	sulfotransferase family 1D, member 1	-3.91
1417089_a_at	NM_009897	Ckmt1	creatine kinase, mitochondrial 1, ubiquitous	-3.93
1417408_at	NM_010171	F3	coagulation factor III	-3.93
1455186_a_at	NM_029821	1190003J15Rik	RIKEN cDNA 1190003J15 gene	-3.96
1418697_at	NM_009349	Inmt	indolethylamine N-methyltransferase	-3.98
1453442_at	AK009785	2310043M15Rik	RIKEN cDNA 2310043M15 gene	-4.02
1427660_x_at	BC013496	Gm10883 ///	predicted gene 10883 /// predicted gene 1420 ///	-4.13
1431786_s_at	NM_029821	1190003J15Rik	RIKEN cDNA 1190003J15 gene	-4.18
1418207_at	NM_001173372	Fxyd4	FXYD domain-containing ion transport regulator	-4.18
1454903_at	NM_033217	Ngfr	nerve growth factor receptor (TNFR superfamily,	-4.38
1455266_at	NM_008449	Kif5c	kinesin family member 5C	-4.39
1451440_at	NM_139134	Chodl	chondrolectin	-4.50
1442113_at	NM_001033304	5330417C22Rik	RIKEN cDNA 5330417C22 gene	-4.69
1422789_at	NM_009022	Aldh1a2	aldehyde dehydrogenase family 1, subfamily A2	-4.80
1420539_a_at	NM_133709	Chrdl2	chordin-like 2	-4.88
1415837_at	NM_010639	Klk1	kallikrein 1	-4.95

Probe set	Accession No.	Symbol	Gene Title	Fold Change
1427044_a_at	NM_175007	Amph	amphiphysin	-5.11
1438671_at	NM_172994	Ppp2r2c	protein phosphatase 2 (formerly 2A), regulatory	-5.41
1425763_x_at	BC019425	Igh-2 /// Igh-	immunoglobulin heavy chain 2 (serum IgA) ///	-5.57
1417785_at	NM_134102	Pla1a	phospholipase A1 member A	-5.59
1433529_at	NM_173749	Pamr1	peptidase domain containing associated with	-5.88
1419476_at	NM_021475	Adamdec1	ADAM-like, decysin 1	-6.11
1426858_at	NM_008381	Inhbb	inhibin beta-B	-6.18
1442143_at	NM_178773	Ano4	anoctamin 4	-6.81
1416761_at	NM_008289	Hsd11b2	hydroxysteroid 11-beta dehydrogenase 2	-6.92
1417789_at	NM_011330	Ccl11	chemokine (C-C motif) ligand 11	-7.13
1421653_a_at	NM_134051	Igh-2 /// Igh-	immunoglobulin heavy chain 2 (serum IgA) ///	-7.60
1436279_at	NM_145947	Slc26a7	solute carrier family 26, member 7	-11.80

Table S3. Direct target genes of β-catenin

Probe Set	Accession No.	Symbol	Gene Title	Fold Change	Chr	Gene Start	Gene End	Interval Dists to Start	Interval Pos	Avg Peak
1454866_s_at	NM_172469	Clic6	chloride intracellular channel 6	62.35	16	92498392	92541486	84243208	in gene, in gene, in gene	5.33
1425425_a_at	NM_011915	Wif1	Wnt inhibitory factor 1	18.13	10	120471286	120537698	-326	upstream	4.00
1434165_at	NM_172469	Clic6	chloride intracellular channel 6	14.07	16	92498392	92541486	84243208	in gene, in gene, in gene	5.33
1417676_a_at	NM_011216	Ptpro	protein tyrosine phosphatase, receptor type, O	13.44	6	137200986	137411754	110174	in gene	4.00
1453645_at	XM_001002365, XM_984603	2700046A07Rik	RIKEN cDNA 2700046A07 gene	10.42	18	62911328	62915969	321	in gene	5.00
1421262_at	NM_010720	Lipg	lipase, endothelial	7.79	18	75099017	75120760	-9064	upstream	6.00
1424556_at	NM_144795	Pycr1	pyrroline-5-carboxylate reductase 1	7.75	11	120497026	120504984	9992	downstream	6.00
1429506_at	NM_027280	Nkd1	naked cuticle 1 homolog (Drosophila)	7.69	8	91045269	91116225	1.69393E+19	in gene, in gene, in gene, in gene	8.00
1421679_a_at	NM_007669	Cdkn1a	cyclin-dependent kinase inhibitor 1A (P21)	7.64	17	29230720	29237667	-10208	upstream	5.00
1422941_at	NM_053116	Wnt16	wingless-related MMTV integration site 16	7.05	6	22238230	22248522	1162	in gene	5.00
1422914_at	NM_022435	Sp5	trans-acting transcription factor 5	6.63	2	70312980	70315783	-3721756	upstream, in gene	5.50
1430118_at	XM_001002365, XM_984603	2700046A07Rik	RIKEN cDNA 2700046A07 gene	6.60	18	62911328	62915969	321	in gene	5.00
1418457_at	NM_019568	Cxcl14	chemokine (C-X-C motif) ligand 14	6.31	13	56390004	56397912	8872	downstream	6.00
1418003_at	NM_025427	1190002H23Rik	RIKEN cDNA 1190002H23 gene	6.24	14	79688557	79701442	130	in gene	5.00
1436845_at	NM_015732	Axin2	axin2	6.11	11	108781680	108812097	776	in gene	5.00
1438511_a_at	NM_025427	1190002H23Rik	RIKEN cDNA 1190002H23 gene	6.11	14	79688557	79701442	130	in gene	5.00
1450188_s_at	NM_010720	Lipg	lipase, endothelial	6.06	18	75099017	75120760	-9064	upstream	6.00
1440617_at	NM_177834	Cpa6	carboxypeptidase A6	5.69	1	10314808	10710024	8808	in gene	6.00
1449286_at	NM_030699	Ntng1	netrin G1	5.65	3	109584024	109946146	264130	in gene	5.00
1417278_a_at	NM_027280	Nkd1	naked cuticle 1 homolog (Drosophila)	5.61	8	91045269	91116225	1.69393E+19	in gene, in gene, in gene, in gene	8.00
1424638_at	NM_007669	Cdkn1a	cyclin-dependent kinase inhibitor 1A (P21)	5.55	17	29230720	29237667	-10208	upstream	5.00
1419465_at	NM_028186	Nkd2	naked cuticle 2 homolog (Drosophila)	5.21	13	73957581	73985060	2660	in gene	6.00
1419466_at	NM_028186	Nkd2	naked cuticle 2 homolog (Drosophila)	5.09	13	73957581	73985060	2660	in gene	6.00
1451054_at	NM_008768	Orm1	orosomucoid 1	4.87	4	63005600	63009196	6304	downstream	6.00
1434275_at	NM_028186	Nkd2	naked cuticle 2 homolog (Drosophila)	4.80	13	73957581	73985060	2660	in gene	6.00
1423854_a_at	NM_026878	Rasl11b	RAS-like, family 11, member B	4.74	5	74591351	74595502	6409	downstream	5.00
1418456_a_at	NM_019568	Cxcl14	chemokine (C-X-C motif) ligand 14	4.60	13	56390004	56397912	8872	downstream	6.00
1435026_at	NM_052994	Spock2	sparc/osteonectin, cwcv and kazal-like domains proteoglycan 2	4.22	10	59569005	59596662	-7293	upstream	5.00
1422758_at	NM_018763	Chst2	carbohydrate sulfotransferase 2	3.85	9	95304920	95307141	48	in gene	5.00
1448147_at	NM_013869	Tnfrsf19	tumor necrosis factor receptor superfamily, member 19	3.45	14	61582873	61665306	5017533946	in gene, in gene	5.50
1439251_at	NM_008325	Idua	Iduronidase, alpha-L-	3.41	5	109098384	109113573	5360	in gene	7.00
1452260_at	NM_178373	Cidec	cell death-inducing DFFA-like effector c	3.35	6	113374630	113385749	17760	downstream	6.00
1425212_a_at	NM_013869	Tnfrsf19	tumor necrosis factor receptor superfamily, member 19	3.30	14	61582873	61665306	5017533946	in gene, in gene	5.50
1415936_at	NM_013867	Bcar3	breast cancer anti-estrogen resistance 3	3.25	3	122122745	122233101	42791	in gene	5.00
1417230_at	NM_023884	Ralgps2	Ral GEF with PH domain and SH3 binding motif 2	3.20	1	158738253	158869711	751	in gene	5.00
1433972_at	NM_001081557	Camta1	calmodulin binding transcription activator 1	3.14	4	150433635	151235855	5.71599E+23	in gene, in gene, in gene, in gene	7.00
1424096_at	NM_027011	Krt5	keratin 5	3.13	15	101537501	101543322	-5750	upstream	6.00
1429273_at	NM_028472	Bmper	BMP-binding endothelial regulator	3.12	9	23027520	23289646	177008	in gene	7.00
1434089_at	XM_001479956, XM_001479958, XM_981156	Synpo	synaptopodin	3.04	18	60753627	60784227	2933128387	in gene, in gene	6.50
1452309_at	NM_026599	Cgnl1	cingulin-like 1	3.04	9	71474316	71619409	1.24337E+21	in gene, in gene, in gene, in gene	6.75
1438713_at	NM_027760	Rassf8	Ras association (RalGDS/AF-6) domain family (N-terminal) member 8	2.91	6	145756903	145766104	-3927	upstream	7.00
1418326_at	NM_011404	Slc7a5	solute carrier family 7 (cationic amino acid transporter, y+ system), member 5	2.91	8	124405050	124431571	3496332467	downstream, downstream	8.00
1419748_at	NM_011994	Abcd2	ATP-binding cassette, sub-family D (ALD), member 2	2.89	15	90976302	91022238	1550	in gene	6.00
1424704_at	NM_009820	Runx2	runt related transcription factor 2	2.85	17	44740950	44951576	1.66472E+16	in gene, in gene, in gene	5.33
1422479_at	NM_019811	Acss2	acyl-CoA synthetase short-chain family member 2	2.84	2	155343779	155388479	77	in gene	6.00
1416612_at	NM_009994	Cyp1b1	cytochrome P450, family 1, subfamily b, polypeptide 1	2.79	17	80106293	80114381	-1747	upstream	5.00
1428789_at	NM_023884	Ralgps2	Ral GEF with PH domain and SH3 binding motif 2	2.63	1	158738253	158869711	751	in gene	5.00
1420796_at	NM_009644	Ahrr	aryl-hydrocarbon receptor repressor	2.59	13	74348566	74429757	46557	in gene	8.00
1449342_at	NM_023587	Ptplb	protein tyrosine phosphatase-like (proline instead of catalytic arginine), member b	2.49	16	35022507	35109261	-59	upstream	7.00
1437329_at	NM_023587	Ptplb	protein tyrosine phosphatase-like (proline instead of catalytic arginine), member b	2.47	16	35022507	35109261	-59	upstream	7.00
1420425_at	NM_007548	Prdm1	PR domain containing 1, with ZNF domain	2.46	10	44156983	44178554	18282	in gene	4.00
1421841_at	NM_008010	Fgfr3	fibroblast growth factor receptor 3	2.46	5	34064373	34079712	-5061	upstream	6.00
1420731_a_at	NM_007792	Csrp2	cysteine and glycine-rich protein 2	2.44	10	110357235	110376678	-115	upstream	8.00
1418908_at	NM_013626	Pam	peptidylglycine alpha-amidating monooxygenase	2.44	1	99717671	99873836	61228	in gene	5.00
1452514_a_at	NM_021099	Kit	kit oncogene	2.42	5	75971049	76052747	11847	in gene	5.00
1438012_at	NM_178726	Ppm1l	protein phosphatase 1 (formerly 2C)-like	2.41	3	69120840	69359326	22792	in gene	6.00
1453022_at	NM_026730	Gpihbp1	GPI-anchored HDL-binding protein 1	2.40	15	75427088	75428643	2245	downstream	5.00
1417697_at	NM_009230	Soat1	sterol O-acyltransferase 1	2.39	1	158358239	158404459	-117	upstream	5.00
1423258_at	NM_021889	Syt9	synaptotagmin IX	2.39	7	114514304	114692169	16	in gene	6.00
1430425_at	NM_172800	Sdk2	sidekick homolog 2 (chicken)	2.34	11	113642104	113927265	1.1925E+11	in gene, in gene, in gene	6.00
1457038_at	NM_172862	Frem2	Fras1 related extracellular matrix protein 2	2.31	3	53317860	53461277	57965	in gene	5.00
1423285_at	NM_007728	Coch	coagulation factor C homolog (Limulus polyphemus)	2.28	12	52694442	52706760	374	in gene	5.00
1433719_at	NM_177909	Slc9a9	solute carrier family 9 (sodium/hydrogen exchanger), member 9	2.26	9	94570337	95130864	-10089199359	upstream, in gene	5.50
1451458_at	NM_001033759, NM_031997	Tmem2	transmembrane protein 2	2.25	19	21852832	21932817	52256	in gene	5.00
1452283_at	NM_027760	Rassf8	Ras association (RalGDS/AF-6) domain family (N-terminal) member 8	2.18	6	145756903	145766104	-3927	upstream	7.00
1425863_a_at	NM_011216	Ptpro	protein tyrosine phosphatase, receptor type, O	2.18	6	137200986	137411754	110174	in gene	4.00
1435551_at	NM_175276	Fhod3	formin homology 2 domain containing 3	2.16	18	24867946	25292002	172950	in gene	5.00
1417801_a_at	NM_008905	Ppfibp2	PTPRF interacting protein, binding protein 2 (liprin beta 2)	2.15	7	114738780	114888399	79876	in gene	5.00
1450863_a_at	NM_019978	Dclk1	doublecortin-like kinase 1	2.15	3	55046550	55340600	153802	in gene	4.00
1429209_at	NM_153393	Col23a1	collagen, type XXIII, alpha 1	2.14	11	51103422	51394612	1.3293E+11	in gene, in gene	6.50
1450451_at	NM_052994	Spock2	sparc/osteonectin, cwcv and kazal-like domains proteoglycan 2	2.14	10	59569005	59596662	-7293	upstream	5.00
1426981_at	XM_355911, XM_886136, XM_905687, XM_919493	Pcsk6	proprotein convertase subtilisin/kexin type 6	2.13	7	73007022	73195272	110738	in gene	6.00
1428705_at	NM_027040	1700007K13Rik	RIKEN cDNA 1700007K13 gene	2.13	2	28317521	28321844	-1980	upstream	5.00
1416939_at	NM_026438	Ppa1	pyrophosphatase (inorganic) 1	2.12	10	61111369	61136913	11031	in gene	5.00
1421341_at	NM_015732	Axin2	axin2	2.11	11	108781680	108812097	776	in gene	5.00
1438789_s_at	NM_009468	Dpysl3	dihydropyrimidinase-like 3	2.10	18	43483936	43552985	-1415	upstream	6.00
1435653_at	NM_018811	Abhd2	abhydrolase domain containing 2	2.06	7	86418152	86506487	243112	in gene, in gene	6.50

Probe Set	Accession No.	Symbol	Gene Title	Fold Change	Chr	Gene Start	Gene End	Interval Dists to Start	Interval Pos	Avg Peak
1440446_at	NM_027280	Nkd1	naked cuticle 1 homolog (Drosophila)	2.05	8	91045269	91116225	1.69393E+19	in gene, in gene, in gene, in gene	8.00
1435699_at	NM_178726	Ppm1l	protein phosphatase 1 (formerly 2C)-like	2.05	3	69120840	69359326	22792	in gene	6.00
1455466_at	NM_001081342	Gpr133	G protein-coupled receptor 133	2.03	5	129602625	129710474	110191	downstream	6.00
1450782_at	NM_009523	Wnt4	wingless-related MMTV integration site 4	2.03	4	136833550	136852694	35709056	in gene, in gene	4.50
1435787_at	NM_178726	Ppm1l	protein phosphatase 1 (formerly 2C)-like	2.02	3	69120840	69359326	22792	in gene	6.00
1417937_at	NM_021532	Dact1	dapper homolog 1, antagonist of beta-catenin (xenopus)	2.02	12	72410971	72419881	-171	upstream	5.00
1448029_at	NM_011535, NM_198052	Tbx3	T-box 3	2.01	5	120120678	120134610	-1382902122	upstream, upstream, in gene	6.00
1438329_at	NM_205823	Tlr12	toll-like receptor 12	1.98	4	128292891	128295733	8373139	downstream, upstream	5.50
1456545_at	NM_010553	Il18rap	interleukin 18 receptor accessory protein	1.96	1	40572208	40606150	20165	in gene	5.00
1452977_at	NM_177263	Zhx3	zinc fingers and homeoboxes 3	1.96	2	160596183	160698726	-9482	upstream	4.00
1424790_at	NM_001007570	Slc25a42	solute carrier family 25, member 42	1.95	8	72708239	72736155	20299101	in gene, upstream	6.00
1415945_at	NM_008566	Mcm5	minichromosome maintenance deficient 5, cell division cycle 46 (S. cerevisiae)	1.93	8	77633427	77652338	15181	in gene	5.00
1451899_a_at	NM_001081462, NM_001081463, NM_001081464, NM_001081465, NM_001081466, NM_001081467, NM_001081468, NM_001081469, NM_001081470, NM_020331	Gtf2ird1	general transcription factor II I repeat domain-containing 1	1.93	5	134833531	134932581	68997	in gene	7.00
1424089_a_at	NM_013685	Tcf4	transcription factor 4	1.92	18	69504175	69843998	317841	in gene	6.00
1450650_at	NM_019472	Myo10	myosin X	1.92	15	25552305	25743426	55583161823	in gene, in gene	5.00
1424767_at	NM_174988	Cdh22	cadherin 22	1.92	2	164937009	165060219	2011	in gene	6.00
1437393_at	NM_011101	Prkca	protein kinase C, alpha	1.91	11	107799562	108205242	7215418938	in gene, in gene	6.00
1425310_a_at	NM_024474	Emid2	EMI domain containing 2	1.90	5	137217634	137358977	7243328513	in gene, in gene	6.00
1429549_at	NM_025685	Col27a1	collagen, type XXVII, alpha 1	1.89	4	62876446	62996025	32770	in gene	6.00
1440911_at	NM_153393	Col23a1	collagen, type XXIII, alpha 1	1.89	11	51103422	51394612	1.3293E+11	in gene, in gene	6.50
1433971_at	NM_001081557	Camta1	calmodulin binding transcription activator 1	1.88	4	150433635	151235855	5.71599E+23	in gene, in gene, in gene, in gene	7.00
1455609_at	NM_007708	Cit	citron	1.88	5	116295665	116456352	13007169039	in gene, downstream	5.50
1416724_x_at	NM_013685	Tcf4	transcription factor 4	1.88	18	69504175	69843998	317841	in gene	6.00
1448052_at	NM_026599	Cgnl1	cingulin-like 1	1.87	9	71474316	71619409	1.24337E+21	in gene, in gene, in gene, in gene	6.75
1431704_a_at	NM_023884	Ralgps2	Ral GEF with PH domain and SH3 binding motif 2	1.86	1	158738253	158869711	751	in gene	5.00
1426955_at	NM_009929	Col18a1	collagen, type XVIII, alpha 1	1.86	10	76514924	76629246	343024478	in gene, in gene	8.00
1424711_at	NM_001033759, NM_031997	Tmem2	transmembrane protein 2	1.84	19	21852832	21932817	52256	in gene	5.00
1442021_at	NM_010307, NM_177137	Gnal	guanine nucleotide binding protein, alpha stimulating, olfactory type	1.83	18	67247990	67386446	116906	in gene	8.00
1456640_at	NM_172966	Sh3rf2	SH3 domain containing ring finger 2	1.83	18	42213364	42318105	1404	in gene	5.00
1415877_at	NM_009468	Dpysl3	dihydropyrimidinase-like 3	1.82	18	43483936	43552985	-1415	upstream	6.00
1419724_at	NM_010100	Edar	ectodysplasin-A receptor	1.81	10	58063537	58138444	3884	in gene	7.00
1431335_a_at	NM_023395	Wfdc1	WAP four-disulfide core domain 1	1.80	8	122190265	122211920	31063	downstream	6.00
1423250_a_at	NM_009367	Tgfb2	transforming growth factor, beta 2	1.79	1	188447521	188529868	-2580	upstream	6.00
1417040_a_at	NM_016778	Bok	BCL2-related ovarian killer protein	1.78	1	95582271	95592339	1.21712E+13	in gene, downstream, downstream	6.67
1429210_at	NM_153393	Col23a1	collagen, type XXIII, alpha 1	1.77	11	51103422	51394612	1.3293E+11	in gene, in gene	6.50
1418190_at	NM_011134	Pon1	paraoxonase 1	1.77	6	5118105	5143824	4208	in gene	5.00
1422912_at	NM_007554	Bmp4	bone morphogenetic protein 4	1.73	14	47003195	47010274	3682	in gene	8.00
1429214_at	NM_029981	Adamtsl2	ADAMTS-like 2	1.73	2	26934901	26964133	13723	in gene	5.00
1440275_at	NM_019732	Runx3	runt related transcription factor 3	1.73	4	134676560	134733905	43675	in gene	6.00
1456812_at	NM_011994	Abcd2	ATP-binding cassette, sub-family D (ALD), member 2	1.73	15	90976302	91022238	1550	in gene	6.00
1427980_at	XM_001004724, XM_924798	4933407C03Rik	RIKEN cDNA 4933407C03 gene	1.72	8	119780993	119985406	7.54551E+16	in gene, in gene, in gene	6.00
1418331_at	NM_025402	1110031I02Rik	RIKEN cDNA 1110031I02 gene	1.72	11	121038907	121065962	5210	in gene	7.00
1440564_at	NM_144944	Prokr2	prokineticin receptor 2	1.71	2	132196901	132211136	-48	upstream	8.00
1454969_at	NM_177139	Lypd6	LY6/PLAUR domain containing 6	1.71	2	49983890	50049084	-7986	upstream	6.00
1436808_x_at	NM_008566	Mcm5	minichromosome maintenance deficient 5, cell division cycle 46 (S. cerevisiae)	1.71	8	77633427	77652338	15181	in gene	5.00
1418260_at	NM_015755	Hunk	hormonally upregulated Neu-associated kinase	1.70	16	90386642	90499798	38574	in gene	6.00
1452118_at	NM_028244	Rrp1b	ribosomal RNA processing 1 homolog B (S. cerevisiae)	1.70	17	32173107	32197550	26765	downstream	8.00
1435021_at	NM_001038701, NM_008071	Gabrb3	gamma-aminobutyric acid (GABA) A receptor, subunit beta 3	1.70	7	64845904	65084172	592	in gene	6.00
1430779_at	NM_030699	Ntng1	netrin G1	1.70	3	109584024	109946146	264130	in gene	5.00
1448346_at	NM_007687	Cfl1	cofilin 1, non-muscle	1.70	19	5490525	5495201	-1437	upstream	7.00
1437479_x_at	NM_011535, NM_198052	Tbx3	T-box 3	1.70	5	120120678	120134610	-1382902122	upstream, upstream, in gene	6.00
1422699_at	NM_007440	Alox12	arachidonate 12-lipoxygenase	1.69	11	70054957	70068843	747	in gene	5.00
1422607_at	NM_007960	Etv1	ets variant gene 1	1.69	12	39506897	39594790	-1569	upstream	6.00
1416723_at	NM_013685	Tcf4	transcription factor 4	1.69	18	69504175	69843998	317841	in gene	6.00
1430533_a_at	NM_007614	Ctnnb1	catenin (cadherin associated protein), beta 1	1.68	9	120842593	120869625	-4919679	upstream, in gene	7.00
1418237_s_at	NM_009929	Col18a1	collagen, type XVIII, alpha 1	1.67	10	76514924	76629246	343024478	in gene, in gene	8.00
1434320_at	NM_172977	Gtf3c4	general transcription factor IIIC, polypeptide 4	1.67	2	28681095	28695806	3582	in gene	6.00
1449089_at	NM_173440	Nrip1	nuclear receptor interacting protein 1	1.67	16	76291107	76373294	58654	in gene	6.00
1459838_s_at	NM_001017525, NM_028709	Btbd11	BTB (POZ) domain containing 11	1.67	10	84850564	85123037	6.17561E+16	in gene, in gene, in gene	5.33
1417695_a_at	NM_009230	Soat1	sterol O-acyltransferase 1	1.66	1	158358239	158404459	-117	upstream	5.00
1434149_at	NM_013685	Tcf4	transcription factor 4	1.65	18	69504175	69843998	317841	in gene	6.00
1423499_at	NM_026408	Sncaip	synuclein, alpha interacting protein (synphilin)	1.65	18	52927363	53075584	38621	in gene	6.00
1428792_at	NM_029815	Bcas1	breast carcinoma amplified sequence 1	1.65	2	170172648	170253328	26224	in gene	4.00
1454295_at	NM_173440	Nrip1	nuclear receptor interacting protein 1	1.64	16	76291107	76373294	58654	in gene	6.00
1451344_at	NM_146162	Tmem119	transmembrane protein 119	1.64	5	114243738	114250367	-65	upstream	7.00
1450922_a_at	NM_009367	Tgfb2	transforming growth factor, beta 2	1.64	1	188447521	188529868	-2580	upstream	6.00
1443870_at	NM_001033336	Abcc4	ATP-binding cassette, sub-family C (CFTR/MRP), member 4	1.64	14	118881914	119105427	11635	in gene	7.00
1436528_at	NM_178929	Kazald1	Kazal-type serine peptidase inhibitor domain 1	1.64	19	45150629	45153772	-1114	upstream	5.00
1458512_at	NM_009389	Tle3	transducin-like enhancer of split 3, homolog of Drosophila E(spl)	1.63	9	61221479	61264791	569	in gene	6.00
1419300_at	NM_010228	Flt1	FMS-like tyrosine kinase 1	1.63	5	148373772	148537564	160364	in gene	6.00
1448443_at	NM_009250	Serpini1	serine (or cysteine) peptidase inhibitor, clade I, member 1	1.63	3	75361495	75446276	73049	in gene	7.00
1456117_at	NM_028244	Rrp1b	ribosomal RNA processing 1 homolog B (S. cerevisiae)	1.63	17	32173107	32197550	26765	downstream	8.00
1455785_at	NM_010595	Kcna1	potassium voltage-gated channel, shaker-related subfamily, member 1	1.62	6	126586481	126595819	-1541	upstream	5.00
1438431_at	NM_011994	Abcd2	ATP-binding cassette, sub-family D (ALD), member 2	1.62	15	90976302	91022238	1550	in gene	6.00

Probe Set	Accession No.	Symbol	Gene Title	Fold Change	Chr	Gene Start	Gene End	Interval Dists to Start	Interval Pos	Avg Peak
1424471_at	NM_144850	Rapgef3	Rap guanine nucleotide exchange factor (GEF) 3	1.62	15	97575384	97598103	20695	in gene	5.00
1434856_at	NM_001081433	Ankrd44	ankyrin repeat domain 44	1.62	1	54702184	54983202	171618	in gene	7.00
1432176_a_at	NM_007932	Eng	endoglin	1.61	2	32502115	32537638	3339389	in gene, in gene	6.50
1449991_at	NM_018729	Cd244	CD244 natural killer cell receptor 2B4	1.60	1	173489324	173515447	-364	upstream	5.00
1451117_a_at	NM_028011	Tom1l1	target of myb1-like 1 (chicken)	1.60	11	90507005	90548915	23619	in gene	6.00
1426805_at	NM_011417	Smarca4	SWI/SNF related, matrix associated, actin dependent regulator of chromatin, subfamily a, member 4	1.60	9	21420613	21507811	59	in gene	6.00
1454930_at	NM_173038	Tbcel	tubulin folding cofactor E-like	1.59	9	42220400	42280309	13941	in gene	6.00
1453191_at	NM_025685	Col27a1	collagen, type XXVII, alpha 1	1.59	4	62876446	62996025	32770	in gene	6.00
1449559_at	NM_013601	Msx2	homeobox, msh-like 2	1.58	13	53562250	53568149	5333	in gene	6.00
1449586_at	NM_019645	Pkp1	plakophilin 1	1.58	1	137767972	137815601	56913	downstream	5.00
1416702_at	NM_009250	Serpini1	serine (or cysteine) peptidase inhibitor, clade I, member 1	1.58	3	75361495	75446276	73049	in gene	7.00
1422631_at	NM_013464	Ahr	aryl-hydrocarbon receptor	1.57	12	36182651	36219661	-19	upstream	7.00
1453015_at	XM_001004724, XM_924798	4933407C03Rik	RIKEN cDNA 4933407C03 gene	1.57	8	119780993	119985406	7.54551E+16	in gene, in gene, in gene	6.00
1450106_a_at	NM_007965	Evl	Ena-vasodilator stimulated phosphoprotein	1.57	12	109792930	109926726	-7714	upstream	6.00
1460330_at	NM_013470	Anxa3	annexin A3	1.56	5	97222434	97274987	1228612670	in gene, in gene	4.50
1452317_at	NM_008270	Hoxb9	homeobox B9	1.56	11	96132644	96137909	764	in gene	5.00
1437445_at	NM_001039104, NM_018752	Trpm1	transient receptor potential cation channel, subfamily M, member 1	1.55	7	71298814	71414658	55938	in gene	7.00
1455689_at	NM_175284	Fzd10	frizzled homolog 10 (Drosophila)	1.55	5	129106981	129109968	-2661	upstream	5.00
1418061_at	NM_013589	Ltbp2	latent transforming growth factor beta binding protein 2	1.54	12	86124162	86217445	68949	in gene	5.00
1453084_s_at	XM_907370, XM_981889	Col22a1	collagen, type XXII, alpha 1	1.54	15	71628906	71864639	239	in gene	5.00
1444242_at	NM_033314	Slco2a1	Solute carrier organic anion transporter family, member 2a1	1.54	9	102910819	102990179	70125	in gene	6.00
1423563_at	NM_030890	Prrt1	proline-rich transmembrane protein 1	1.54	17	34766631	34769205	-20232793	upstream, downstream	6.50
1434263_at	NM_028657	F630110N24Rik	RIKEN cDNA F630110N24 gene	1.54	10	80820236	80828967	-55007332	upstream, in gene	5.50
1437012_x_at	NM_144850	Rapgef3	Rap guanine nucleotide exchange factor (GEF) 3	1.54	15	97575384	97598103	20695	in gene	5.00
1418469_at	NM_173440	Nrip1	nuclear receptor interacting protein 1	1.54	16	76291107	76373294	58654	in gene	6.00
1428354_at	NM_001080932	Foxk2	forkhead box K2	1.54	11	121116808	121171210	4248	in gene	7.00
1438133_a_at	NM_010516	Cyr61	cysteine rich protein 61	1.53	3	145309940	145312945	2241	in gene	6.00
1420401_a_at	NM_019511	Ramp3	receptor (calcitonin) activity modifying protein 3	1.53	11	6558536	6577478	2232	in gene	5.00
1450945_at	NM_011101	Prkca	protein kinase C, alpha	1.53	11	107799562	108205242	7215418938	in gene, in gene	6.00
1424755_at	NM_146001	Hip1	huntingtin interacting protein 1	1.52	5	135883896	136020985	5554547001	in gene, in gene	7.00
1422544_at	NM_019472	Myo10	myosin X	1.52	15	25552305	25743426	55583161823	in gene, in gene	5.00
1417696_at	NM_009230	Soat1	sterol O-acyltransferase 1	1.52	1	158358239	158404459	-117	upstream	5.00
1422466_at	NM_008750	Nxn	nucleoredoxin	1.52	11	76070738	76212626	136191	in gene	4.00
1435908_at	NM_020253	Nrxn2	neurexin II	1.52	19	6428016	6533217	81872	in gene	5.00
1437695_at	NM_144944	Prokr2	prokineticin receptor 2	1.51	2	132196901	132211136	-48	upstream	8.00
1455951_at	NM_001003913	Mars	methionine-tRNA synthetase	1.50	10	126733282	126748695	21015	downstream	7.00
1421158_at	NM_026599	Cgnl1	cingulin-like 1	1.50	9	71474316	71619409	1.24337E+21	in gene, in gene, in gene, in gene	6.75
1450923_at	NM_009367	Tgfb2	transforming growth factor, beta 2	1.50	1	188447521	188529868	-2580	upstream	6.00
1452232_at	NM_144731	Galnt7	UDP-N-acetyl-alpha-D-galactosamine: polypeptide N-acetylgalactosaminyltransferase 7	-1.52	8	60003366	60131800	6834	in gene	4.00
1415975_at	NM_025821	Carhsp1	calcium regulated heat stable protein 1	-1.52	16	8658680	8672246	1814	in gene	8.00
1419133_at	NM_025276	Evpl	envoplakin	-1.52	11	116081873	116099405	165575299	in gene, upstream	5.00
1417169_at	NM_016808, NM_198091, NM_198092	Usp2	ubiquitin specific peptidase 2	-1.52	9	43875104	43903710	-6.41222E+14	upstream, in gene, in gene, in gene	6.00
1448694_at	NM_010591	Jun	Jun oncogene	-1.52	4	94715746	94718878	-482	upstream	8.00
1438560_x_at	NM_009837	Cct4	chaperonin containing Tcp1, subunit 4 (delta)	-1.52	11	22890593	22903336	84	in gene	6.00
1428643_at	NM_145128	Mgat5	mannoside acetylglucosaminyltransferase 5	-1.52	1	129101563	129379549	22960	in gene	6.00
1439962_at	XM_896611, XM_922997	2310010J17Rik	RIKEN cDNA 2310010J17 gene	-1.53	7	97273368	97278775	599	in gene	6.00
1418259_a_at	NM_009849	Entpd2	ectonucleoside triphosphate diphosphohydrolase 2	-1.53	2	25251478	25256838	234	in gene	6.00
1417409_at	NM_010591	Jun	Jun oncogene	-1.53	4	94715746	94718878	-482	upstream	8.00
1435549_at	NM_175130	Trpm4	transient receptor potential cation channel, subfamily M, member 4	-1.53	7	52558002	52589112	33581	downstream	4.00
1417933_at	NM_008344	Igfbp6	insulin-like growth factor binding protein 6	-1.54	15	101974793	101979943	6759	downstream	6.00
1433782_at	NM_022890	Cldn12	claudin 12	-1.54	5	5505109	5514873	366	in gene	6.00
1437217_at	NM_001012450, NM_001012451, NM_080471	Ankrd6	ankyrin repeat domain 6	-1.54	4	32891010	33037801	12274586169	in gene, in gene	6.00
1425553_s_at	NM_145070	Hip1r	huntingtin interacting protein 1 related	-1.54	5	124423648	124453209	2668829376	in gene, in gene	7.00
1435261_at	NM_198967	Tmtc1	transmembrane and tetratricopeptide repeat containing 1	-1.54	6	148185194	148392874	196085	in gene	5.00
1424052_at	NM_025920	Thap4	THAP domain containing 4	-1.54	1	95601968	95651415	5725549911	downstream, downstream	7.00
1451431_a_at	NM_001048227, NM_001048228, NM_001048229, NM_026797	Dbndd2	dysbindin (dystrobrevin binding protein 1) domain containing 2	-1.55	2	164311640	164318823	11368	downstream	6.00
1434963_at	NM_178652	Supt3h	suppressor of Ty 3 homolog (S. cerevisiae)	-1.55	17	44914120	45256233	-232	upstream	5.00
1420640_at	NM_021310	Jmy	junction-mediating and regulatory protein	-1.56	13	94204157	94269644	172	in gene	7.00
1423891_at	NM_133994	Gstt3	glutathione S-transferase, theta 3	-1.56	10	75236867	75244159	7791	downstream	6.00
1424468_s_at	NM_153537	Phldb1	pleckstrin homology-like domain, family B, member 1	-1.56	9	44494391	44543277	-2131	upstream	8.00
1423717_at	NM_021299	Ak3	adenylate kinase 3	-1.56	19	29095322	29122392	-5267	upstream	5.00
1424467_at	NM_153537	Phldb1	pleckstrin homology-like domain, family B, member 1	-1.57	9	44494391	44543277	-2131	upstream	8.00
1419457_at	NM_012026	Rgnef	Rho-guanine nucleotide exchange factor	-1.58	13	98668550	98976120	190104	in gene	6.00
1418294_at	XM_001476161, XM_001476174, XM_980440	Epb4.1l4b	erythrocyte protein band 4.1-like 4b	-1.58	4	57008606	57156520	132856	in gene	5.00
1417516_at	NM_007837	Ddit3	DNA-damage inducible transcript 3	-1.59	10	126727866	126733342	-186	upstream	7.00
1460634_at	NM_009058	Ralgds	ral guanine nucleotide dissociation stimulator	-1.59	2	28368851	28408590	1250438685	in gene, in gene	6.50
1419442_at	NM_016762	Matn2	matrilin 2	-1.60	15	34236436	34365997	193	in gene	5.00
1452195_s_at	NM_030207	Sfi1	Sfi1 homolog, spindle assembly associated (yeast)	-1.61	11	3031853	3093466	69626	downstream	13.00
1450063_at	NM_019445	Fmn2	formin 2	-1.61	1	176419303	176752208	35673	in gene	7.00
1417168_a_at	NM_016808, NM_198091, NM_198092	Usp2	ubiquitin specific peptidase 2	-1.61	9	43875104	43903710	-6.41222E+14	upstream, in gene, in gene, in gene	6.00
1424919_at	NM_001003817	Erbb2	v-erb-b2 erythroblastic leukemia viral oncogene homolog 2, neuro/glioblastoma derived oncogene homolog (avian)	-1.61	11	98273798	98299030	22442	in gene	5.00
1428074_at	NM_001002267	Tmem158	transmembrane protein 158	-1.62	9	123168175	123169907	8275	downstream	6.00
1422747_at	NM_016681	Chek2	CHK2 checkpoint homolog (S. pombe)	-1.62	5	111269036	111303152	29588	in gene	10.00
1437449_at	NM_001013381	Rsad1	radical S-adenosyl methionine domain containing 1	-1.63	11	94401112	94410513	-15	upstream	6.00
1456559_at	NR_002863	Emx2os	empty spiracles homolog 2 (Drosophila) opposite strand	-1.64	19	59499594	59533125	22517	in gene	7.00
1455096_at	NM_201518	Flrt2	fibronectin leucine rich transmembrane protein 2	-1.65	12	96930464	97019235	2816	in gene	6.00
1429727_at	NM_025807	Slc16a9	solute carrier family 16 (monocarboxylic acid transporters), member 9	-1.65	10	69708024	69748699	14056	in gene	7.00

Probe Set	Accession No.	Symbol	Gene Title	Fold Change	Chr	Gene Start	Gene End	Interval Dists to Start	Interval Pos	Avg Peak
1419056_at	NM_001025364, NM_013648	Rtn2	reticulon 2 (Z-band associated protein)	-1.66	7	19868016	19881509	13360	in gene	6.00
1423284_at	NM_026345	Mansc1	MANSC domain containing 1	-1.66	6	134559228	134582506	-8806	upstream	4.00
1437594_x_at	NM_133779	Pigt	phosphatidylinositol glycan anchor biosynthesis, class T	-1.66	2	164323025	164333801	-17	upstream	6.00
1417848_at	NM_133218	Zfp704	zinc finger protein 704	-1.67	3	9427023	9610085	85429	in gene	6.00
1425396_a_at	NM_010693	Lck	lymphocyte protein tyrosine kinase	-1.67	4	129225602	129235616	-10048	upstream	5.00
1423718_at	NM_021299	Ak3	adenylate kinase 3	-1.68	19	29095322	29122392	-5267	upstream	5.00
1426383_at	NM_009963	Cry2	cryptochrome 2 (photolyase-like)	-1.68	2	92243803	92274185	10089	in gene	7.00
1426937_at	NM_027519	6330406I15Rik	RIKEN cDNA 6330406I15 gene	-1.69	5	150214381	150234278	16675	in gene	5.00
1426606_at	NM_145123	Crtac1	cartilage acidic protein 1	-1.69	19	42357527	42506273	133153	in gene	5.00
1439428_x_at	NM_146041	Gmds	GDP-mannose 4, 6-dehydratase	-1.70	13	31911455	32430413	69357	in gene	6.00
1448530_at	NM_025508	Gmpr	guanosine monophosphate reductase	-1.71	13	45602838	45641750	15914	in gene	7.00
1453498_x_at	NM_133186	Steap3	STEAP family member 3	-1.71	1	122123783	122161834	3322616778	in gene, in gene	7.00
1449510_at	NM_020589	Zfp467	zinc finger protein 467	-1.71	6	48387585	48395606	1590	in gene	5.00
1437784_at	NM_009822	Runx1t1	runt-related transcription factor 1; translocated to, 1 (cyclin D-related)	-1.71	4	13711929	13818264	48295	in gene	6.00
1420349_at	NM_008966	Ptgfr	prostaglandin F receptor	-1.73	3	151461574	151500492	-7444	upstream	5.00
1443260_at	NM_010789	Meis1	Meis homeobox 1	-1.74	11	18780431	18918683	482591877	in gene, upstream	8.00
1448785_at	NM_009822	Runx1t1	runt-related transcription factor 1; translocated to, 1 (cyclin D-related)	-1.75	4	13711929	13818264	48295	in gene	6.00
1425016_at	NM_010142	Ephb2	Eph receptor B2	-1.75	4	136209523	136391850	10469886650	in gene, in gene	5.00
1448656_at	NM_001044741, NM_007581	Cacnb3	calcium channel, voltage-dependent, beta 3 subunit	-1.75	15	98462651	98474961	229813829	in gene, downstream	5.50
1451323_at	NM_145916	Zfp7	zinc finger protein 7	-1.76	15	76709689	76722825	20615	downstream	7.00
1434007_at	NM_172670	Gyltl1b	glycosyltransferase-like 1B	-1.78	2	92205209	92211168	-9552	upstream	5.00
1449019_at	NM_001042541, NM_009648	Akap1	A kinase (PRKA) anchor protein 1	-1.78	11	88692106	88725900	76	in gene	7.00
1450992_a_at	NM_010789	Meis1	Meis homeobox 1	-1.78	11	18780431	18918683	482591877	in gene, upstream	8.00
1421424_a_at	NM_008486	Anpep	alanyl (membrane) aminopeptidase	-1.79	7	86966689	86987170	49305854	in gene, upstream	6.50
1434158_at	NM_146041	Gmds	GDP-mannose 4, 6-dehydratase	-1.79	13	31911455	32430413	69357	in gene	6.00
1452244_at	NM_027519	6330406I15Rik	RIKEN cDNA 6330406I15 gene	-1.80	5	150214381	150234278	16675	in gene	5.00
1429029_at	NM_028943	Sgms2	sphingomyelin synthase 2	-1.80	3	131025656	131047853	24157	downstream	5.00
1424175_at	NM_017376, NM_153484	Tef	thyrotroph embryonic factor	-1.80	15	81633247	81657293	8673	in gene	8.00
1444615_x_at	NM_009822	Runx1t1	runt-related transcription factor 1; translocated to, 1 (cyclin D-related)	-1.81	4	13711929	13818264	48295	in gene	6.00
1456133_x_at	NM_010580	Itgb5	integrin beta 5	-1.82	16	33829845	33949232	-7205	upstream	6.00
1429206_at	NM_001081347	Rhobtb1	Rho-related BTB domain containing 1	-1.83	10	68675405	68754539	65971	in gene	7.00
1418472_at	NM_023113	Aspa	aspartoacylase	-1.84	11	73118490	73138136	22680	downstream	5.00
1454733_at	NM_172729	Nod1	nucleotide-binding oligomerization domain containing 1	-1.88	6	54873943	54922405	-59	upstream	7.00
1424127_at	NM_010165	Eya2	eyes absent 2 homolog (Drosophila)	-1.89	2	165480798	165597131	-414	upstream	7.00
1417533_a_at	NM_010580	Itgb5	integrin beta 5	-1.89	16	33829845	33949232	-7205	upstream	6.00
1438411_at	NM_175520	Gpr81	G protein-coupled receptor 81	-1.89	5	124326987	124330012	-8612	upstream	5.00
1417534_at	NM_010580	Itgb5	integrin beta 5	-1.90	16	33829845	33949232	-7205	upstream	6.00
1439764_s_at	NM_183029	Igf2bp2	insulin-like growth factor 2 mRNA binding protein 2	-1.91	16	22059128	22163092	40788	in gene	4.00
1425248_a_at	NM_019392	Tyro3	TYRO3 protein tyrosine kinase 3	-1.92	2	119625250	119643840	19182	downstream	6.00
1429891_at	NM_029341	Capsl	calcyphosine-like	-1.95	15	9365783	9395790	30841	downstream	6.00
1428811_at	NM_181819	Wfikkn2	WAP, follistatin/kazal, immunoglobulin, kunitz and netrin domain containing 2	-1.95	11	94098912	94103863	6135361	downstream, upstream	8.50
1427640_a_at	NM_009822	Runx1t1	runt-related transcription factor 1; translocated to, 1 (cyclin D-related)	-1.95	4	13711929	13818264	48295	in gene	6.00
1439527_at	NM_008829	Pgr	progesterone receptor	-1.96	9	8899833	8968611	70567	downstream	8.00
1452213_at	NM_198292	Tex2	testis expressed gene 2	-1.96	11	106363453	106474244	111908	downstream	7.00
1427673_a_at	NM_011348	Sema3e	sema domain, immunoglobulin domain (Ig), short basic domain, secreted, (semaphorin) 3E	-1.97	5	14025276	14256689	148676	in gene	6.00
1418186_at	NM_008185	Gstt1	glutathione S-transferase, theta 1	-1.97	10	75246560	75261329	24961	downstream	6.00
1420484_a_at	NM_011707	Vtn	vitronectin	-1.99	11	78312788	78315826	1548	in gene	4.00
1419647_a_at	NM_133662	Ier3	immediate early response 3	-2.01	17	35958658	35959856	3341726	in gene, downstream	6.00
1449315_at	NM_011857	Odz3	odd Oz/ten-m homolog 3 (Drosophila)	-2.01	8	49313038	49760044	431436	in gene	6.00
1451453_at	NM_010019	Dapk2	death-associated protein kinase 2	-2.02	9	66006072	66120049	-88	upstream	6.00
1453587_at	NM_027819	Ggt6	gamma-glutamyltransferase 6	-2.03	11	72249028	72251909	5788	downstream	7.00
1436584_at	NM_011897	Spry2	sprouty homolog 2 (Drosophila)	-2.05	14	106291732	106296036	-604	upstream	6.00
1421444_at	NM_008829	Pgr	progesterone receptor	-2.06	9	8899833	8968611	70567	downstream	8.00
1429178_at	NM_011857	Odz3	odd Oz/ten-m homolog 3 (Drosophila)	-2.08	8	49313038	49760044	431436	in gene	6.00
1454048_a_at	NM_027627	4931408A02Rik	RIKEN cDNA 4931408A02 gene	-2.08	16	90831358	90904940	51682	in gene	9.00
1448263_a_at	NM_023149	Cndp2	CNDP dipeptidase 2 (metallopeptidase M20 family)	-2.09	18	84836861	84855025	-175	upstream	4.00
1456130_at	XM_287555, XM_983072	Thsd7a	thrombospondin, type I, domain containing 7A	-2.10	6	12265952	12699188	146788	in gene	6.00
1430355_a_at	NM_133186	Steap3	STEAP family member 3	-2.10	1	122123783	122161834	3322616778	in gene, in gene	7.00
1419564_at	NM_020589	Zfp467	zinc finger protein 467	-2.11	6	48387585	48395606	1590	in gene	5.00
1441572_at	NM_007831	Dcc	deleted in colorectal carcinoma	-2.11	18	71413286	72510723	1052915	in gene	5.00
1447901_x_at	NM_030207	Sfi1	Sfi1 homolog, spindle assembly associated (yeast)	-2.14	11	3031853	3093466	69626	downstream	13.00
1460177_at	NM_023149	Cndp2	CNDP dipeptidase 2 (metallopeptidase M20 family)	-2.15	18	84836861	84855025	-175	upstream	4.00
1456195_x_at	NM_010580	Itgb5	integrin beta 5	-2.15	16	33829845	33949232	-7205	upstream	6.00
1460242_at	NM_010016	Cd55	CD55 antigen	-2.16	1	132335606	132359317	28341	downstream	5.00
1434606_at	NM_010153	Erbb3	v-erb-b2 erythroblastic leukemia viral oncogene homolog 3 (avian)	-2.17	10	128006424	128026557	23181	downstream	7.00
1448321_at	NM_022316	Smoc1	SPARC related modular calcium binding 1	-2.18	12	82127818	82287387	110694	in gene	6.00
1435667_at	NM_001012623, NM_001012624, NM_001012625, NM_053270, NM_183018	Rims1	regulating synaptic membrane exocytosis 1	-2.18	1	22278503	22422924	147180	downstream	5.00
1426787_at	NM_030207	Sfi1	Sfi1 homolog, spindle assembly associated (yeast)	-2.21	11	3031853	3093466	69626	downstream	13.00
1434881_s_at	NM_177715	Kctd12	potassium channel tetramerisation domain containing 12	-2.22	14	103375798	103381854	30	in gene	8.00
1421761_a_at	NM_013800	Barx2	BarH-like homeobox 2	-2.24	9	31653865	31720658	63618	in gene	6.00
1417602_at	NM_011066	Per2	period homolog 2 (Drosophila)	-2.27	1	93312559	93355873	-1183	upstream	6.00
1434581_at	NM_026629	2410066E13Rik	RIKEN cDNA 2410066E13 gene	-2.27	6	54631766	54650400	23738	downstream	7.00
1441727_s_at	NM_020589	Zfp467	zinc finger protein 467	-2.28	6	48387585	48395606	1590	in gene	5.00
1438305_at	NM_001012623, NM_001012624, NM_001012625, NM_053270, NM_183018	Rims1	regulating synaptic membrane exocytosis 1	-2.29	1	22278503	22422924	147180	downstream	5.00
1443906_at	NM_010016	Cd55	CD55 antigen	-2.43	1	132335606	132359317	28341	downstream	5.00
1455772_at	NM_008829	Pgr	progesterone receptor	-2.44	9	8899833	8968611	70567	downstream	8.00
1428663_at	NM_028943	Sgms2	sphingomyelin synthase 2	-2.45	3	131025656	131047853	24157	downstream	5.00
1444740_at	XM_620510, XM_907730	Lass3	LAG1 homolog, ceramide synthase 3	-2.46	7	73888390	73966728	14602	in gene	5.00
1429918_at	NM_175535	Arhgap20	Rho GTPase activating protein 20	-2.48	9	51573457	51661164	7343	in gene	4.00
1440487_at	NM_007831	Dcc	deleted in colorectal carcinoma	-2.59	18	71413286	72510723	1052915	in gene	5.00

Probe Set	Accession No.	Symbol	Gene Title	Fold Change	Chr	Gene Start	Gene End	Interval Dists to Start	Interval Pos	Avg Peak
1434210_s_at	NM_008377	Lrig1	leucine-rich repeats and immunoglobulin-like domains 1	-2.60	6	94554523	94650139	72179	in gene	5.00
1419420_at	NM_012028	St6galnac5	ST6 (alpha-N-acetyl-neuraminyl-2,3-beta-galactosyl-1,3)-N-acetylgalactosaminide alpha-2,6-sialyltransferase 5	-2.62	3	152482850	152645174	169350	downstream	7.00
1452358_at	NM_198409	Rai2	retinoic acid induced 2	-2.66	0	158155559	158217425	29465	in gene	6.00
1449893_a_at	NM_008377	Lrig1	leucine-rich repeats and immunoglobulin-like domains 1	-2.71	6	94554523	94650139	72179	in gene	5.00
1423468_at	NM_133186	Steap3	STEAP family member 3	-2.72	1	122123783	122161834	3322616778	in gene, in gene	7.00
1418762_at	NM_010016	Cd55	CD55 antigen	-2.77	1	132335606	132359317	28341	downstream	5.00
1424902_at	NM_028199	Plxdc1	plexin domain containing 1	-2.95	11	97784552	97847694	-8178	upstream	5.00
1442226_at	NM_011348	Sema3e	sema domain, immunoglobulin domain (Ig), short basic domain, secreted, (semaphorin) 3E	-3.06	5	14025276	14256689	148676	in gene	6.00
1419717_at	NM_011348	Sema3e	sema domain, immunoglobulin domain (Ig), short basic domain, secreted, (semaphorin) 3E	-3.31	5	14025276	14256689	148676	in gene	6.00
1418207_at	NM_033648	Fxyd4	FXYD domain-containing ion transport regulator 4	-4.18	6	117883577	117887353	-1975	upstream	6.00
